# Cucurbitacin B Promotes Tumor Necrosis Factor Receptor 1 Ectodomain Shedding by Selectively Activating the Extracellular Signal-Regulated Kinase Signaling Pathway

**DOI:** 10.3390/ijms27115011

**Published:** 2026-06-01

**Authors:** Piimwara Yarangsee, Itsuki Fukai, Sophany Phol, Kosei Kinugawa, Eiichi Kusagawa, Yasunobu Miyake, Takao Kataoka

**Affiliations:** 1 Department of Applied Biology, Kyoto Institute of Technology, Kyoto 606-8585, Japan; 2 Division of Molecular and Cellular Immunoscience, Department of Biomolecular Sciences, Faculty of Medicine, Saga University, Saga 849-8501, Japan; 3 Center for Social and Biomedical Engineering, Kyoto Institute of Technology, Kyoto 606-8585, Japan

**Keywords:** cucurbitacin B, triterpenoid, TNF receptor 1, shedding, ADAM, ERK, RAF1, MAPK, NF-κB

## Abstract

Cucurbitacin B belongs to a group of tetracyclic triterpenoids and exerts a number of biological effects, including anti-inflammatory and anticancer activities. We previously demonstrated that cucurbitacin B down-regulated tumor necrosis factor (TNF) receptor 1 (TNF-R1) expression and prevented activation of the transcription factor nuclear factor κB in response to a TNF-α stimulation. The present study shows that cucurbitacin B promoted the ectodomain shedding of TNF-R1 by generating a soluble form that accumulated in the culture medium of human lung adenocarcinoma A549 cells. Of the eight tetracyclic and pentacyclic triterpenoids consisting of an *α*,*β*-unsaturated carbonyl group that were examined, only cucurbitacin B promoted TNF-R1 ectodomain shedding. Cucurbitacin B-induced TNF-R1 shedding was attenuated by TNF-α protease inhibitor 2 and the mitogen-activated protein kinase (MAPK)/extracellular signal-regulated kinase (ERK) kinase (MEK) inhibitor U0126, but not by the p38 MAPK inhibitor SB203580 or the c-Jun *N*-terminal kinase (JNK) inhibitor SP600125. Consistent with these results, cucurbitacin B promoted the rapid phosphorylation of rapidly accelerated fibrosarcoma 1 (RAF1) and ERK, but exerted minimal effects on the phosphorylation of p38 MAPK and JNK. Collectively, these results demonstrate that cucurbitacin B selectively activated the RAF1-MEK-ERK pathway, which was essential for TNF-R1 ectodomain shedding.

## 1. Introduction

Tumor necrosis factor (TNF)-α controls immune and inflammatory responses and, thus, is involved in the pathogenesis of inflammatory diseases and cancer [1,2]. TNF-α specifically interacts with two cell surface receptors: TNF receptor 1 (TNF-R1) and TNF-R2 [3,4,5]. TNF-R1 is broadly expressed in the majority of cells, while TNF-R2 is expressed in limited cell types [3,4,5]. In response to a TNF-α stimulation, TNF-R1 forms a membrane-bound signaling complex that mainly activates the transcription factor nuclear factor κB (NF-κB) signaling pathway as well as the mitogen-activated protein (MAP) kinase (MAPK) signaling pathway, which regulate the expression of numerous target genes, such as proinflammatory and prosurvival genes [6]. TNF-R1 may also form secondary cytosolic signaling complexes that activate the cell death pathway, leading to apoptosis and necroptosis [6]. In contrast, TNF-R2 forms a distinct signaling complex that induces the NF-κB and MAPK signaling pathways [4,5].

The MAPK family mainly consists of three major signaling pathways: extracellular signal-regulated kinase (ERK), p38 MAPK, and c-Jun *N*-terminal kinase (JNK) [7]. In response to extracellular stimuli, these kinases are activated by stepwise phosphorylation involving upstream MAP kinase kinase (MAP2K) and MAP kinase kinase kinase (MAP3K) in kinase cascades [7]. Classical ERK1 and ERK2 are phosphorylated by MAPK/ERK kinase (MEK) 1 and MEK2, which belong to MAP2K [8]. MEK1 and MEK2 are phosphorylated by multiple MAP3Ks, including rapidly accelerated fibrosarcoma (RAF) kinases [8]. The RAF kinase family comprises ARAF, BRAF, and CRAF (also known as RAF1), which undergo dimerization after binding to the rat sarcoma (RAS) GTPase at the plasma membrane and become activated in the process [9]. The RAS-RAF-MEK-ERK axis plays a crucial role in controlling various cellular processes, including cell cycle, proliferation, and differentiation [10].

Ectodomain shedding is a post-translational process that cleaves membrane-bound proteins and regulates their expression, function, and localization [11]. The a disintegrin and metalloprotease (ADAM) family is ubiquitously expressed in mammalian tissues and comprises more than 20 members in humans [12,13]. ADAM17, also referred to as the TNF-α-converting enzyme, has an *N*-terminal extracellular region with a catalytic metalloprotease domain, a transmembrane region, and a C-terminal cytoplasmic region [14,15]. TNF-α is synthesized and transported to the plasma membrane in a membrane-bound form, which is cleaved by ADAM17 into its soluble form [16]. In addition to TNF-α, ADAM17 is capable of cleaving TNF-R1 and TNF-R2 [14,15,17]. ADAM17 activity is regulated by the phosphorylation of its cytoplasmic domain via ERK and p38 MAPK [18,19,20,21]. Consistent with this finding, we demonstrated that TNF-R1 was processed into its soluble form via the activation of ERK and p38 MAPK in response to ribotoxic stress induced by translation inhibitors [22,23,24,25,26,27].

**Figure 1:**
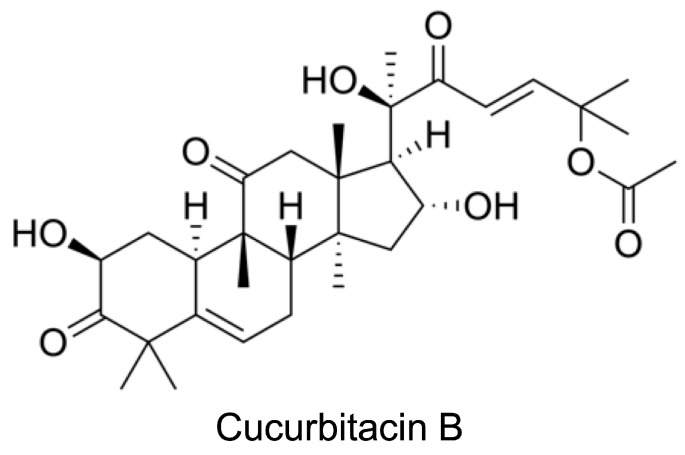
Structure of cucurbitacin B.

Cucurbitacins are natural products belonging to the class of triterpenes characterized by a tetracyclic structure and are found in plants from the Cucurbitaceae family [28,29]. Cucurbitacins, including cucurbitacin B (Figure 1), exert a number of biological effects, particularly anti-inflammatory and anticancer activities [30,31,32,33,34,35]. Previous studies demonstrated that cucurbitacin B prevented the activation of NF-κB induced by TNF-α in human cervical carcinoma HeLa cells [36] and reduced NF-κB reporter activity upon a TNF-α stimulation in human embryonic kidney 293 cells [37], while cucurbitacin E inhibited NF-κB activation induced by TNF-α in human synoviocyte MH7A cells [38]. These findings suggest that cucurbitacins are capable of inhibiting TNF-α-dependent NF-κB signaling; however, the underlying mechanisms remain unclear. We recently reported that cucurbitacin B down-regulated TNF-R1 expression and inhibited TNF-α-dependent NF-κB activation in human lung adenocarcinoma A549 cells [39]. In the present study, we investigated the mechanisms by which cucurbitacin B down-regulates TNF-R1 expression.

## 2. Results

### 2.1. Cucurbitacin B Increased Soluble TNF-R1 Levels in A549 Cells

**Figure 2:**
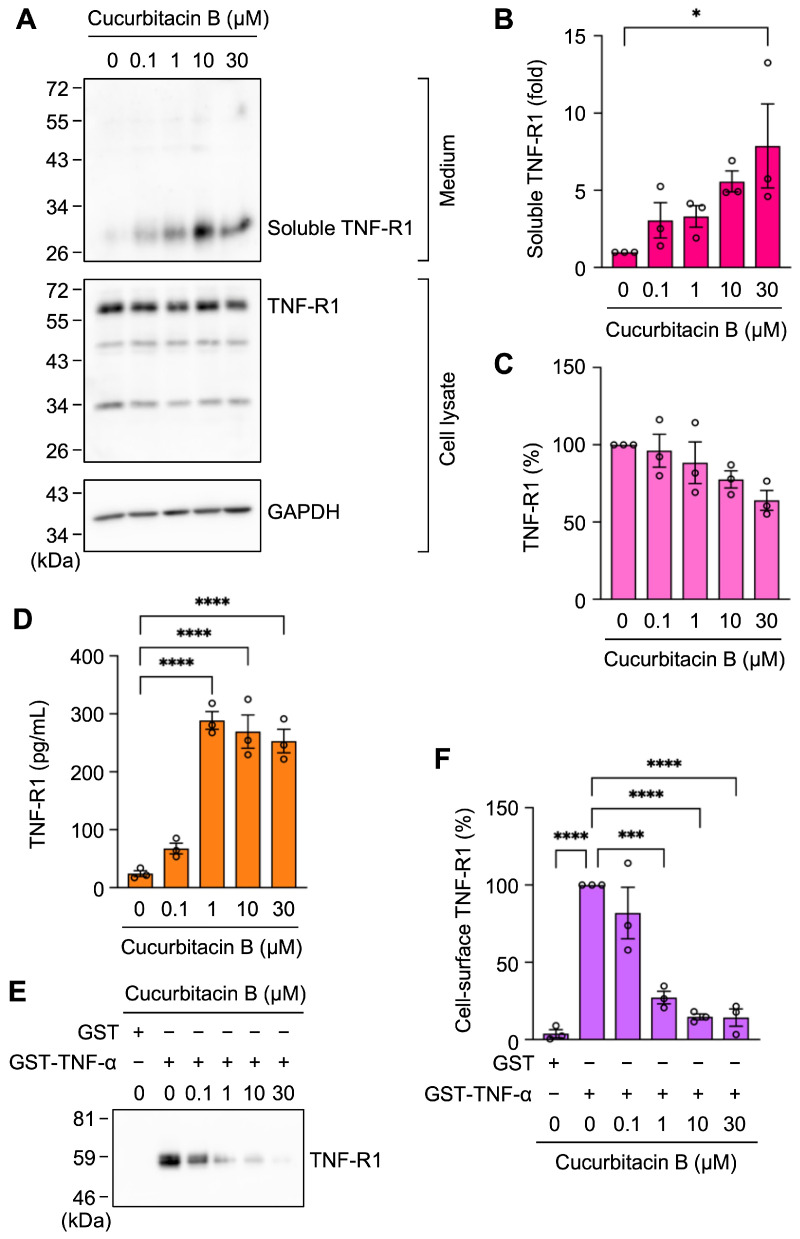
Cucurbitacin B increased soluble TNF-R1 levels in the culture medium. (**A**–**D**) A549 cells were incubated with the indicated concentrations of cucurbitacin B for 1 h. The culture medium and whole-cell lysates were subjected to a Western blot analysis using anti-TNF-R1 antibodies (H-5 and C25C1, respectively). Panel (**A**) shows representative blots from three independent experiments. Soluble TNF-R1 (fold) (**B**) and TNF-R1 (%) (**C**) are expressed as the mean ± standard error (*n* = 3). The culture medium was also subjected to ELISA. TNF-R1 (pg/mL) (**D**) is expressed as the mean ± standard error (*n* = 3). Soluble TNF-R1 (fold) in the control group and TNF-R1 (%) in the control group were assigned values of 1-fold and 100%, respectively. (**E**,**F**) A549 cells were preincubated with the indicated concentrations of cucurbitacin B for 1 h, and were then incubated with (+) or without (−) GST (2 µg/mL) or GST-TNF-α (2 µg/mL) for 5 min on ice. Cell lysates were treated with Glutathione Sepharose 4B. Pull-down products were analyzed by Western blotting. Cell-surface TNF-R1 (%) is expressed as the mean ± standard error (*n* = 3). Individual data points are shown as circles. * *p* < 0.05, *** *p* < 0.001, and **** *p* < 0.0001. Original blots are shown in Figures S1–S12.

We previously showed that cucurbitacin B down-regulated TNF-R1 expression without reducing its mRNA level in A549 cells [39]. To investigate whether cucurbitacin B induced the ectodomain shedding of TNF-R1, we treated A549 cells with cucurbitacin B for 1 h, after which Western blotting was used to analyze the culture medium as well as whole-cell lysates. The anti-TNF-R1 antibody H-5 was raised against amino acids 30–301 within the extracellular domain of human TNF-R1. On the other hand, the anti-TNF-R1 C25C1 was raised against amino acids within the intracellular region and surrounding Ser331 of human TNF-R1. H-5 was used for Western blotting of the culture medium, and C25C1 for that of whole-cell lysates. Cucurbitacin B increased the amount of TNF-R1 bands migrating between the 26 kDa and 34 kDa markers in a dose-dependent manner (Figure 2A,B). We previously detected soluble TNF-R1, resulting from the cleavage of full-length TNF-R1, in the culture medium of A549 cells exposed to translation inhibitors [22,24,25,26,27].

Previous studies reported the cleavage of TNF-R1 between Asn201 and Val202 near the transmembrane domain, generating an extracellular domain containing 30–201 amino acids with three potential *N*-glycosylation sites [40]. Recombinant soluble TNF-R1 consisting of the extracellular domain has been shown to exhibit molecular weights of 28–32 kDa in Chinese hamster ovary cells [41] and 24–28 kDa in High Five insect cells [42]. We previously demonstrated that FLAG-tagged TNF-R1 (1–232), containing approximately 30 additional amino acids, migrated between 28 and 37 kDa when expressed in human embryonic kidney 293T cells [43]. Collectively, these findings support the TNF-R1 bands detected between 26 and 34 kDa in the present study corresponding to soluble extracellular forms generated by ectodomain shedding.

In whole-cell lysates, cucurbitacin B appeared to decrease the amount of full-length TNF-R1 detectable near the 55 kDa marker in a dose-dependent manner (Figure 2A,C). However, in cucurbitacin B-treated cells, the cleaved membrane-bound form of TNF-R1 migrating near a 34 kDa marker was not increased by Western blotting using the anti-TNF-R1 antibody C25C1 (Figure 2A,C), suggesting that the cleaved intracellular region of TNF-R1 undergoes degradation.

The reduction in full-length TNF-R1 by cucurbitacin B was less obvious than that observed in our previous study [39]. This may be due to differences in the experimental conditions employed because the present study used serum-free medium to avoid the interference of a large amount of serum albumin with protein separation during electrophoresis. Since cellular TNF-R1 levels are affected by multiple cellular processes, including transcription, translation, degradation, and shedding, the assessment of soluble TNF-R1 in the culture medium is a more suitable approach for monitoring the selective cleavage of TNF-R1 at the cell surface.

To complement Western blotting results, soluble TNF-R1 was also measured using an enzyme-linked immunosorbent assay (ELISA). In the culture medium of control A549 cells, the amount of soluble TNF-R1 was approximately 24 pg/mL (Figure 2D). Cucurbitacin B at concentrations of 1–30 µM markedly increased soluble TNF-R1 levels to higher than 250 pg/mL (Figure 2D).

Cell-surface TNF-R1 expression was evaluated by a pull-down assay using recombinant human TNF-α fused to glutathione *S*-transferase (GST). A549 cells were treated with cucurbitacin B for 1 h, after which they were incubated with GST or GST-TNF-α for 5 min on ice. GST-TNF-α, but not GST, efficiently captured cell-surface TNF-R1 (Figure 2E,F). Cucurbitacin B decreased the amount of TNF-R1 in GST-TNF-α pull-down products (Figure 2E,F). These results show that cell-surface TNF-R1 was down-regulated by cucurbitacin B.

### 2.2. Cucurbitacin B Promoted TNF-R1 Ectodomain Shedding in A549 Cells

**Figure 3:**
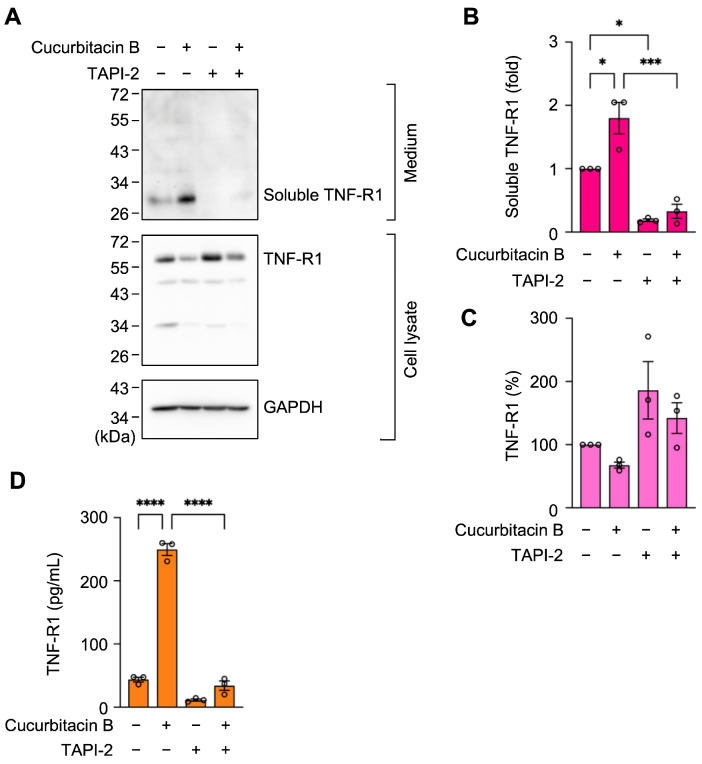
Cucurbitacin B promoted TNF-R1 ectodomain shedding. (**A**–**D**) A549 cells were pretreated with TAPI-2 for 1 h or left untreated, and were then treated for 1 h in the presence (+) or absence (−) of cucurbitacin B (10 µM) or TAPI-2 (25 µM). The culture medium and whole-cell lysates were subjected to a Western blot analysis using anti-TNF-R1 antibodies (H-5 and C25C1, respectively). Panel A presents representative blots from three independent experiments. Soluble TNF-R1 (fold) (**B**) and TNF-R1 (%) (**C**) are expressed as the mean ± standard error (*n* = 3). The culture medium was also subjected to ELISA. TNF-R1 (pg/mL) is expressed as the mean ± standard error (*n* = 3) (**D**). Soluble TNF-R1 (fold) in the control group and TNF-R1 (%) in the control group were assigned values of 1-fold and 100%, respectively. Individual data points are shown as circles. * *p* < 0.05, *** *p* < 0.001 and **** *p* < 0.0001. Original blots are shown in Figures S13–S20.

ADAM17 has been shown to cleave full-length TNF-R1 into its soluble form [14,15,17]. Furthermore, ADAM8 and ADAM10 have been reported to cleave TNF-R1 [44,45,46,47]. TNF-α protease inhibitor 2 (TAPI-2) has been used to investigate whether these ADAM proteases are responsible for increases in soluble TNF-R1 because it suppresses the activities of ADAMs and matrix metalloproteases [48,49,50]. Among these metalloproteases, TAPI-2 was found to exhibit greater selectivity for ADAM17 [49]. In the present study, the pretreatment of A549 cells with TAPI-2 attenuated the increase in soluble TNF-R1 in the culture medium of cucurbitacin B-treated cells (Figure 3A,B). Moreover, TAPI-2 reduced soluble TNF-R1 levels in the absence of cucurbitacin B (Figure 3A,B), suggesting the constitutive processing of TNF-R1 by metalloproteases. Under these conditions, TAPI-2 appeared to increase total TNF-R1 levels in cell lysates (Figure 3A,C), while decreasing cleaved TNF-R1 fragments (Figure 3A). The results of ELISA confirmed that TAPI-2 markedly reduced soluble TNF-R1 levels in both control and cucurbitacin B-treated A549 cells (Figure 3D). These results show that cucurbitacin B promoted TNF-R1 ectodomain shedding.

### 2.3. Cucurbitacin B Increased Soluble TNF-R1 Levels in 293T Cells

**Figure 4:**
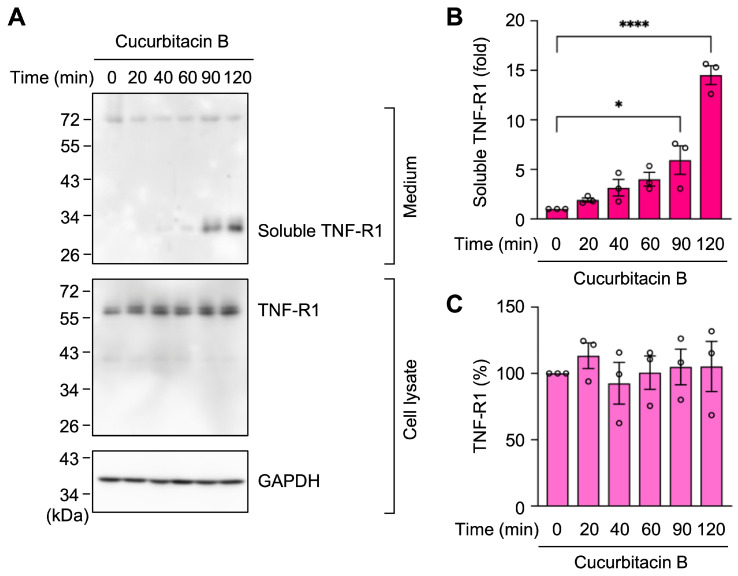
Cucurbitacin B increased soluble TNF-R1 levels in 293T cells. (**A**–**C**) 293T cells were incubated with cucurbitacin B (10 µM) for the indicated times. The culture medium and whole-cell lysates were subjected to a Western blot analysis using an anti-TNF-R1 antibody (H-5). Panel A shows representative blots from three independent experiments. Soluble TNF-R1 (fold) (**B**) and TNF-R1 (%) (**C**) are expressed as the mean ± standard error (*n* = 3). Soluble TNF-R1 (fold) in the control group and TNF-R1 (%) in the control group were assigned values of 1-fold and 100%, respectively. Individual data points are shown as circles. * *p* < 0.05 and **** *p* < 0.0001. Original blots are shown in Figures S21–S28.

We used an additional cell line to investigate whether cucurbitacin B induces the ectodomain shedding of TNF-R1. 293T cells were incubated with cucurbitacin B for different time periods. Cucurbitacin B markedly increased soluble TNF-R1 levels during an incubation for 90–120 min (Figure 4A,B). In contrast, TNF-R1 levels in cell lysates were not affected by the cucurbitacin B treatment (Figure 4A,C). These results indicate that cucurbitacin B induced the ectodomain shedding of TNF-R1 in at least two cell lines, thereby providing support for this phenomenon not being restricted to A549 cells.

### 2.4. Structure-Activity Relationship Between Triterpenoids and TNF-R1 Ectodomain Shedding in A549 Cells

**Figure 5:**
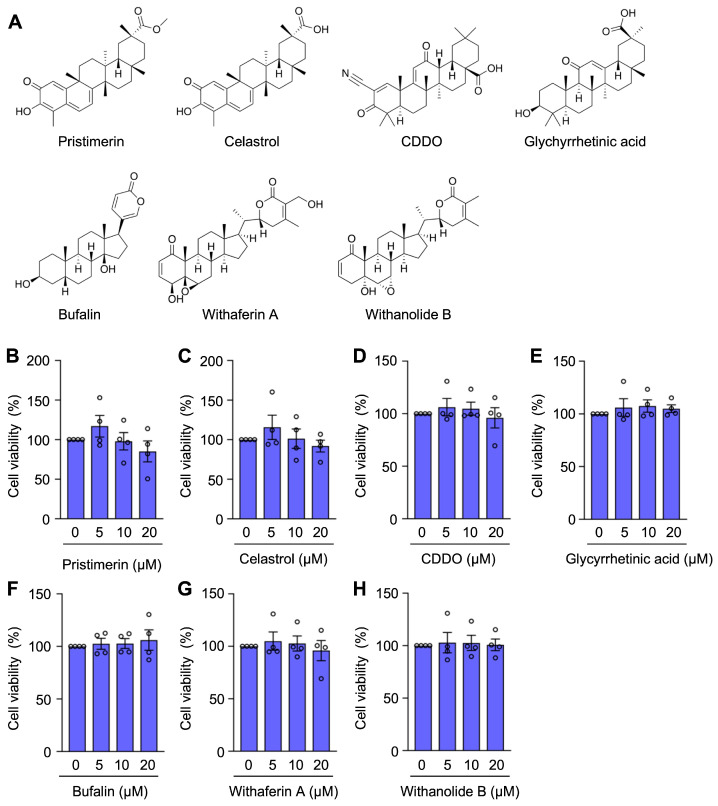
Structures of triterpenoids and their biological effects on cell viability. (**A**) Structures of tetracyclic and pentacyclic triterpenoids. (**B**–**H**) A549 cells were treated with the indicated concentrations of pristimerin (**B**), celastrol (**C**), CDDO (**D**), glycyrrhetinic acid (**E**), bufalin (**F**), withaferin A (**G**), and withanolide B (**H**) for 2 h, after which crystal violet staining was conducted. Cell viability (%) is expressed as the mean ± standard error (*n* = 4). Individual data points are shown as circles. There were no significant differences.

Triterpenoids may be subdivided primarily into those possessing pentacyclic and tetracyclic structures [51]. Cucurbitacin B contains an *α*,*β*-unsaturated carbonyl group [28,29]. This group interacts with the cysteine residues of cellular target proteins via the Michael reaction [52]. We previously reported that glutathione, *N*-acetylcysteine (NAC), and cysteine suppressed the inhibitory effects of cucurbitacin B on gene expression induced by TNF-α [39], suggesting that an *α*,*β*-unsaturated carbonyl group is critical for the biological activity of cucurbitacin B. Pentacyclic and tetracyclic triterpenoids possessing an *α*,*β*-unsaturated carbonyl group have been shown to inhibit NF-κB activation in response to TNF-α [53,54,55,56,57,58]. However, it remains unclear whether triterpenoids containing an *α*,*β*-unsaturated carbonyl group are also capable of promoting TNF-R1 shedding. To investigate the structure-activity relationship of TNF-R1 shedding, we selected seven structurally related triterpenoids (Figure 5A) and examined their effects on TNF-R1 shedding. Crystal violet staining showed that pristimerin, celastrol, 2-cyano-3,12-dioxooleana-1,9-dien-28-oic acid (CDDO), glycyrrhetinic acid, bufalin, withaferin A, and withanolide B at concentrations up to 20 µM did not affect A549 cell viability during a 2 h incubation (Figure 5B–H).

**Figure 6:**
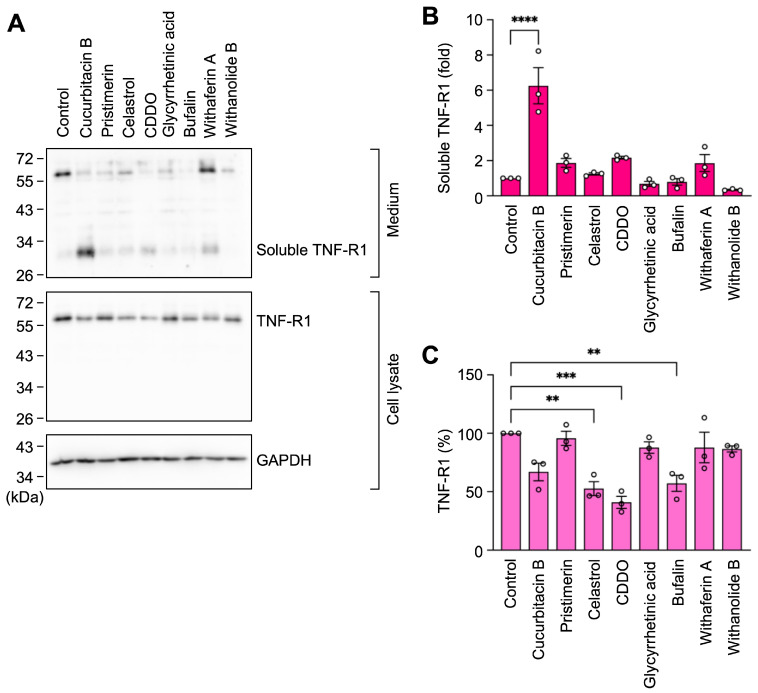
Cucurbitacin B, but not other tetracyclic or pentacyclic triterpenoids, selectively promoted TNF-R1 ectodomain shedding. (**A**–**C**) A549 cells were incubated with cucurbitacin B (10 µM), pristimerin (5 µM), celastrol (5 µM), CDDO (10 µM), glycyrrhetinic acid (10 µM), bufalin (10 µM), withaferin A (10 µM), or withanolide B (10 µM) for 2 h or left untreated (control). The culture medium and whole-cell lysates were subjected to a Western blot analysis using the anti-TNF-R1 antibody H-5. Panel A shows representative blots from three independent experiments. Soluble TNF-R1 (fold) (**B**) and TNF-R1 (%) (**C**) are expressed as the mean ± standard error (*n* = 3). The control group was assigned values of 1-fold and 100%, respectively. Individual data points are shown as circles. ** *p* < 0.01, *** *p* < 0.001, and **** *p* < 0.0001. Original blots are shown in Figures S29–S36.

A549 cells were exposed to eight triterpenoids for 2 h. Cucurbitacin B markedly increased the amount of soluble TNF-R1, whereas the seven other triterpenoids exerted minimal effects on soluble TNF-R1 levels (Figure 6A,B). These results demonstrate that cucurbitacin B selectively promoted TNF-R1 ectodomain shedding among the pentacyclic and tetracyclic triterpenoids tested, all of which possessed at least one *α*,*β*-unsaturated carbonyl group. In contrast, celastrol, CDDO, and bufalin reduced the amount of full-length TNF-R1 in cell lysates without increasing soluble TNF-R1 levels (Figure 6A,C).

**Figure 7:**
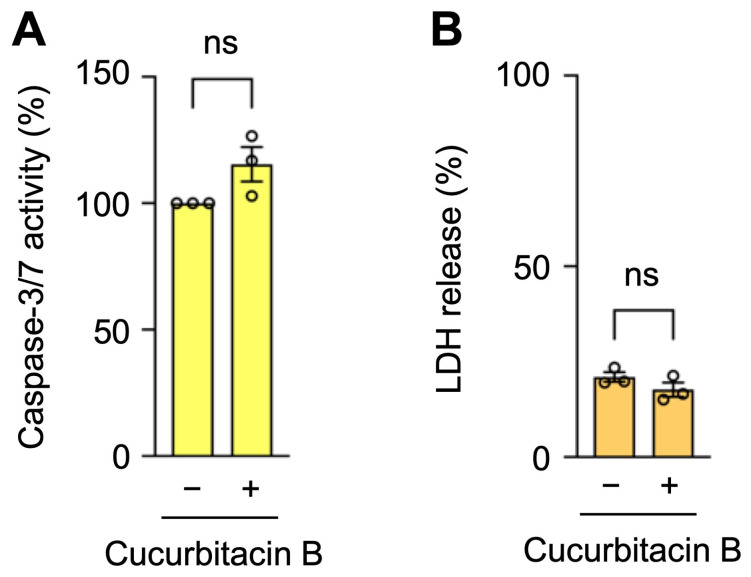
Cucurbitacin B did not markedly affect caspase-3/7 activity or LDH release. (**A**,**B**) A549 cells were incubated with (+) or without (−) cucurbitacin B (10 µM) for 2 h. Caspase-3/7 activity in cell lysates was measured. Caspase-3/7 activity (%) (**A**) is expressed as the mean ± standard error (*n* = 3). LDH activity in the culture medium and total cell lysates was measured. LDH release (%) (**B**) is expressed as the mean ± standard error (*n* = 3). Caspase-3/7 activity and LDH release in the control group were assigned values of 100%. Individual data points are shown as circles. ns, not significant.

We previously showed that cucurbitacin B at concentrations up to 10 µM did not markedly affect cell viability [39]. To investigate whether cucurbitacin B induces apoptosis or necrosis and thereby promotes TNF-R1 shedding, we measured caspase-3/7 activity as an apoptosis marker and lactate dehydrogenase (LDH) release as a necrosis marker during a 2 h incubation. Cucurbitacin B did not markedly affect caspase-3/7 activity or LDH release (Figure 7A,B), suggesting that an apoptosis- or necrosis-associated membrane perturbation is unlikely to account for TNF-R1 shedding in cucurbitacin B-treated cells.

### 2.5. U0126 Attenuated TNF-R1 Ectodomain Shedding Induced by Cucurbitacin B in A549 Cells

**Figure 8:**
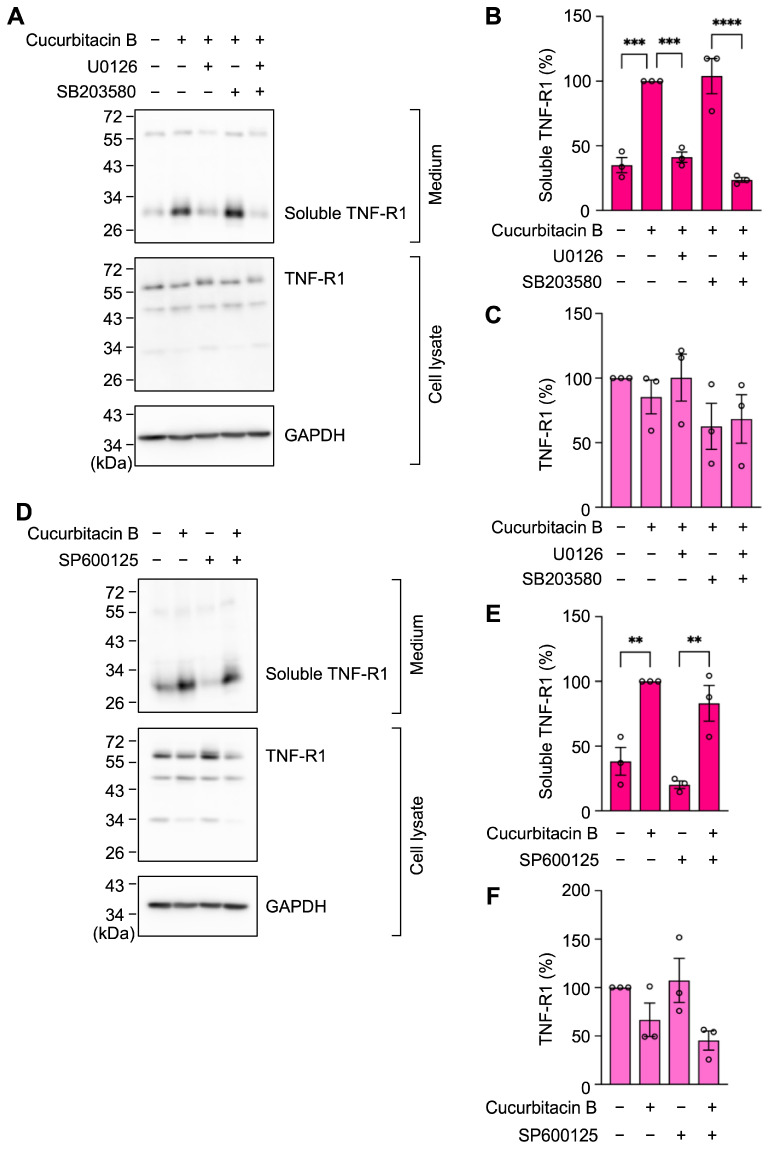
The MEK inhibitor U0126 attenuated TNF-R1 ectodomain shedding induced by cucurbitacin B. (**A**–**F**) A549 cells were pretreated with (+) or without (−) U0126 or SB203580 for 1 h and were then treated for 2 h in the presence (+) or absence (−) of cucurbitacin B (10 µM), U0126 (10 µM), or SB203580 (10 µM) (**A**–**C**). A549 cells were pretreated with (+) SP600125 for 1 h or left untreated (−), and were then treated for 2 h in the presence (+) or absence (−) of cucurbitacin B (10 µM) or SP600125 (10 µM) (**D**–**F**). The culture medium and whole-cell lysates were subjected to a Western blot analysis using anti-TNF-R1 antibodies (H-5 and C25C1, respectively). Panels (**A**,**D**) show representative blots from three independent experiments. Soluble TNF-R1 (%) (**B**,**E**) and TNF-R1 (%) (**C**,**F**) are expressed as the mean ± standard error (*n* = 3). Soluble TNF-R1 levels in the cucurbitacin B-treated group and TNF-R1 levels in the control group were assigned values of 100%. Individual data points are shown as circles. ** p < 0.01, *** *p* < 0.001, and **** *p* < 0.0001. Original blots are shown in Figures S37–S52.

ERK and p38 MAPK have been shown to phosphorylate and thereby activate ADAM17 [18,19,20,21]. To clarify whether ERK and p38 MAPK are involved in TNF-R1 ectodomain shedding, specific protein kinase inhibitors were used: the MEK inhibitor U0126, the p38 MAPK inhibitor SB203580, and the JNK inhibitor SP600125. A549 cells were pretreated with these kinase inhibitors for 1 h and then exposed to cucurbitacin B for 2 h. Cucurbitacin B-induced increases in soluble TNF-R1 levels were attenuated by U0126 alone and U0126 plus SB203580, but not by SB203580 alone (Figure 8A,B). SP600125 did not affect cucurbitacin B-induced increases in soluble TNF-R1 levels (Figure 8D,E). The total levels of full-length TNF-R1 in cell lysates were not markedly affected by these compounds (Figure 8A,C,D,F). These results suggest that the MEK-ERK pathway contributed to TNF-R1 ectodomain shedding induced by cucurbitacin B.

### 2.6. Cucurbitacin B Selectively Induced ERK Phosphorylation in A549 Cells

**Figure 9:**
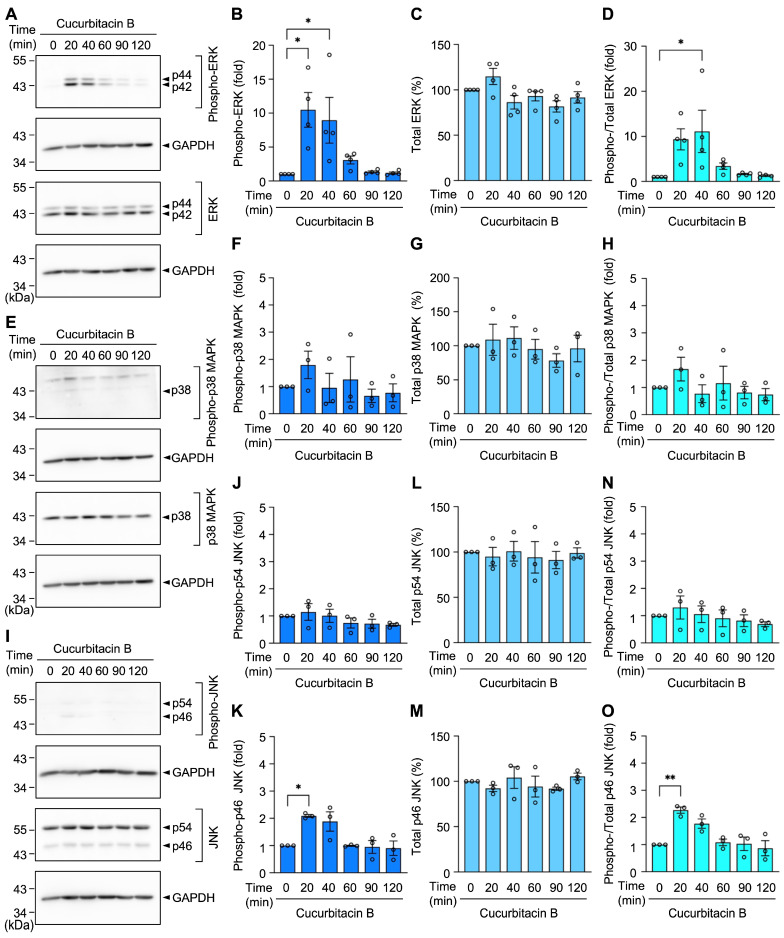
Cucurbitacin B selectively induced ERK phosphorylation. (**A**–**O**) A549 cells were treated with cucurbitacin B (10 µM) for the indicated time periods. Whole-cell lysates were subjected to a Western blot analysis. Panel (**A**) shows representative blots from four independent experiments. Panels (**E**) and (**I**) show representative blots from three independent experiments. Phospho-ERK (fold) (**B**), total ERK (%) (**C**), phospho-ERK/total ERK (fold) (**D**), phospho-p38 MAPK (fold) (**F**), total p38 MAPK (%) (**G**), phospho-p38 MAPK/total p38 MAPK (fold) (**H**), phospho-p54 JNK (fold) (**J**), phospho-p46 JNK (fold) (**K**), total p54 JNK (%) (**L**), total p46 JNK (%) (**M**), phospho-p54 JNK/total p54 JNK (fold) (**N**), and phospho-p46 JNK/total p46 JNK (fold) (**O**) are expressed as the mean ± standard error of four independent experiments (**B**–**D**) and three independent experiments (**F**–**H**,**J**–**O**). Individual data points are shown as circles. * p < 0.05 and ** p < 0.01. Original blots are shown in Figures S53–S78.

The MAPK superfamily members ERK, p38 MAPK, and JNK are activated through phosphorylation by upstream kinases [7]. To investigate whether cucurbitacin B activates MAPK superfamily members, A549 cells were treated with cucurbitacin B for different time periods. The phosphorylated state of each member was then analyzed by Western blotting. Cucurbitacin B induced ERK phosphorylation at 20–40 min, which returned to near-background levels at 90–120 min (Figure 9A,B). Total ERK levels did not markedly change during the incubation with cucurbitacin B (Figure 9A,C). In contrast, cucurbitacin B did not markedly affect the phosphorylation or total amount of p38 MAPK during the 120 min incubation period (Figure 9E–G). Similarly, cucurbitacin B exerted minimal effects on JNK phosphorylation or total JNK levels during the same period (Figure 9I–M). We also evaluated the ratios of phosphorylated relative to total ERK, p38 MAPK, p54 JNK, and p46 JNK. ERK phosphorylation increased by approximately 10-fold, while the phosphorylation of p38 MAPK, p54 JNK, and p46 JNK increased by less than approximately 2-fold (Figure 9D,H,N,O). Collectively, these results suggest that cucurbitacin B selectively induced ERK phosphorylation.

### 2.7. Cucurbitacin B Induced RAF1 Phosphorylation in A549 Cells

**Figure 10:**
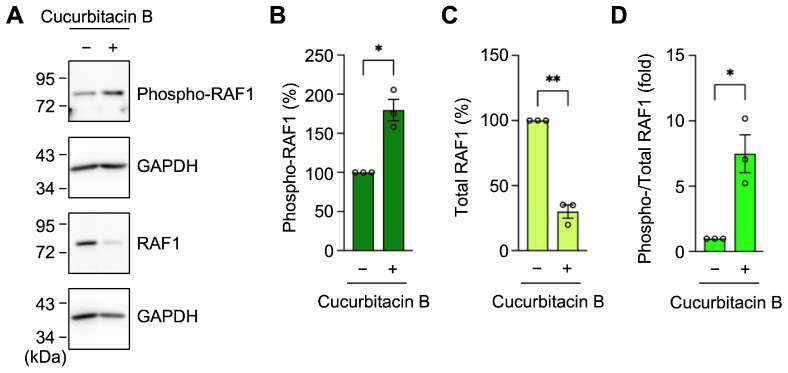
Cucurbitacin B induced RAF1 phosphorylation. (**A**–**D**) A549 cells were incubated with (+) cucurbitacin B (10 µM) for 20 min or left untreated (−). Whole-cell lysates were subjected to a Western blot analysis. Panel (**A**) shows representative blots from three independent experiments. Phospho-RAF1 (%) (**B**), total RAF1 (%) (**C**), and phospho-RAF1/total RAF1 ratios (fold) (**D**) are expressed as the mean ± standard error (*n* = 3). Individual data points are shown as circles. * *p* < 0.05 and ** *p* < 0.01. Original blots are shown in Figures S79–S86.

In the ERK pathway cascade, RAF family members phosphorylate and activate MEK1/2, which, in turn, phosphorylate and activate ERK1/2 [8]. RAF1 is expressed in A549 cells and plays a critical role in regulating proliferation, metastasis, and invasion capabilities [59,60]. In the present study, A549 cells were exposed to cucurbitacin B for 20 min, after which the phosphorylation state of RAF1 was analyzed by Western blotting. Cucurbitacin B increased phospho-RAF1 levels by approximately 2-fold (Figure 10A,B), while total RAF1 levels decreased by more than 50% (Figure 10A,C). The phospho-RAF1/total RAF1 ratio markedly increased following the cucurbitacin B treatment (Figure 10D). These results demonstrate that cucurbitacin B induced RAF1 phosphorylation upstream of ERK phosphorylation.

## 3. Discussion

**Figure 11:**
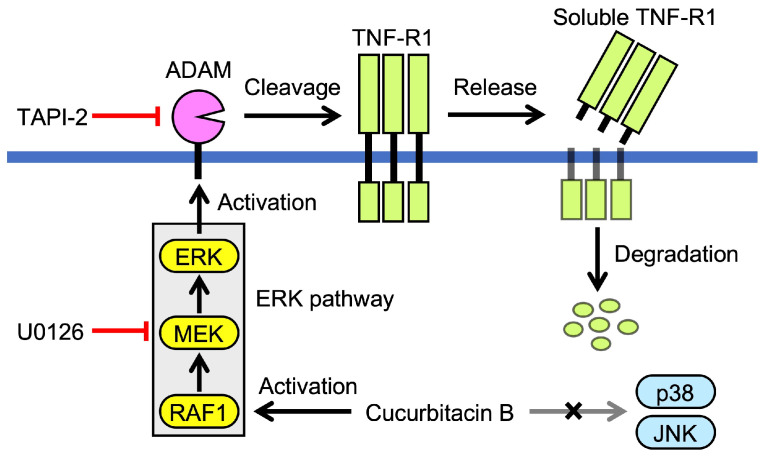
Schematic of cucurbitacin B-induced TNF-R1 ectodomain shedding. Multiple ADAM proteases cleave full-length TNF-R1 at a membrane-proximal site. The resultant soluble TNF-R1, consisting of the extracellular domain, is released into the culture medium. The membrane-bound intracellular region of TNF-R1 then appears to undergo lysosomal degradation. Cucurbitacin B selectively activates the ERK pathway via the RAF1–MEK–ERK axis, while exerting minimal effects on the activation of p38 MAPK and JNK. ERK may contribute to the activation of ADAM proteases involved in TNF-R1 shedding. TNF-R1 ectodomain shedding induced by cucurbitacin B is attenuated by the metalloprotease inhibitor TAPI-2 and the MEK inhibitor U0126.

We previously reported that cucurbitacin B down-regulated TNF-R1 expression with no discernible reduction in the adaptor proteins associated with TNF-R1, namely, TNF receptor-associated death domain protein, TNF receptor-associated factor 2, and receptor-interacting kinase 1 [39]. TAPI-2 reversed the cucurbitacin B-induced decrease in TNF-R1 expression [39]. The present results revealed that cucurbitacin B increased the levels of soluble TNF-R1 in the culture medium of human lung adenocarcinoma A549 cells. Similar effects were observed in 293T cells, which suggests that cucurbitacin B-induced TNF-R1 shedding is not limited to A549 cells. Moreover, TAPI-2 reduced the increase in soluble TNF-R1 in cucurbitacin B-treated cells. TAPI-2 has been shown to inhibit the activities of multiple ADAM and matrix metalloproteases [48,49,50]. TNF-R1 was found to be cleaved by ADAM8, ADAM10, and ADAM17 [44,45,46,47,61,62], with ADAM17 being regarded as a major sheddase in several cell types [61,62]. Taken together, these findings and the present results suggest that cucurbitacin B promotes the ectodomain shedding of cell-surface TNF-R1, presumably mediated by ADAM proteases (Figure 11). Meanwhile, the membrane-bound intracellular region of TNF-R1 did not increase in cucurbitacin B-treated cells (Figure 2A and Figure 3A). We previously demonstrated that a cleaved membrane-bound form of TNF-R1 increased in control and cucurbitacin B-treated cells exposed to bafilomycin A_1_, which specifically inhibits vacuolar-type H^+^-ATPase, thereby preventing lysosomal acidification and protein degradation [39]. Therefore, the cleaved membrane-bound form of TNF-R1 appears to undergo rapid lysosomal degradation (Figure 11).

Proinflammatory cytokines, Toll-like receptor (TLR) ligands, and T-cell receptor (TCR) stimulation have been shown to activate the NF-κB signaling pathway [63,64]. A TCR stimulation may be mimicked by phorbol 12-myristate 13-acetate (PMA, a protein kinase C activator) and ionomycin (a calcium ionophore), which results in the activation of NF-κB [65]. Cucurbitacins B and E were previously shown to inhibit NF-κB activation induced by TNF-α [36,38] and lipopolysaccharide (a TLR4 ligand) [66,67,68], whereas cucurbitacin E blocked NF-κB activation in response to PMA plus ionomycin [69,70]. Cucurbitacin D also inhibited the constitutive activation of the NF-κB signaling pathway in cancer cell lines [71,72,73]. These findings suggest that cucurbitacins interfere with multiple target proteins involved in the NF-κB signaling pathway or its upstream processes that indirectly activate NF-κB. Cucurbitacins may bind directly to multiple cellular proteins via the covalent binding of their *α*,*β*-unsaturated carbonyl group [33]. In the NF-κB signaling pathway, previous studies demonstrated that cucurbitacins B, D, and E inhibited the nuclear translocation and/or phosphorylation of the NF-κB subunit RelA [38,66,67,68,69,70,71,72,73]. Meanwhile, cucurbitacin B was found to have no effect on the nuclear translocation of RelA, but inhibited its transactivation activity in response to TNF-α [36]. Based on these findings, cucurbitacin B may directly inhibit the NF-κB signaling pathway independently of TNF-R1 shedding.

Cucurbitacins are triterpenoids composed of a tetracyclic cucurbitane structure with multiple oxidation groups [29,31]. The *α*,*β*-unsaturated carbonyl group is a bioactive nucleophile that reacts covalently with the cysteine residues of cellular proteins [52]. Many cucurbitacins, including B, D, and E, possess this group and, thus, are biologically active compounds [29,31]. Triterpenoids with an *α*,*β*-unsaturated carbonyl group are among those that have been reported to prevent TNF-α-dependent NF-κB activation [53,54,55,56,57,58]. Unlike cucurbitacin B, these triterpenoids did not markedly affect soluble TNF-R1 levels in the culture medium. These findings indicate that cucurbitacin B selectively induces TNF-R1 ectodomain shedding. The *α*,*β*-unsaturated carbonyl group positioned in the side chain outside the tetracyclic ring appears to be unique to cucurbitacins and may be critical for this biological activity.

Unlike cucurbitacin B, celastrol, CDDO, and bufalin did not promote TNF-R1 shedding; they reduced TNF-R1 levels. We previously showed that the translation inhibitor puromycin decreased the amount of TNF-R1 [27]. In addition, bafilomycin A_1_, which inhibits lysosomal degradation, increased TNF-R1 levels, suggesting that TNF-R1 undergoes lysosomal turnover under basal conditions, whereas the proteasome inhibitor MG-132 reduced TNF-R1 levels [39]. Therefore, TNF-R1 appears to be regulated by multiple biosynthetic and degradation pathways. TNF-R1 has also been shown to undergo ubiquitination, glycosylation, and palmitoylation, which regulate its internalization, proteolysis, and expression [74,75,76]. Collectively, these findings suggest that celastrol, CDDO, and bufalin reduce TNF-R1 expression through intracellular processes, such as translation inhibition, lysosomal degradation, and the ubiquitin-proteasome-dependent regulation of TNF-R1 stability.

We previously showed that ribotoxic stress caused by several translation inhibitors induced the activation of ERK, p38 MAPK, or JNK [22,23,24,25,26,27]. ERK and p38 MAPK have been shown to phosphorylate ADAM17 at a threonine residue in its cytoplasmic region and promote the ectodomain shedding of various receptors [18,19,20,21]. Consistent with these findings, we reported that the pharmacological inhibition of the ERK pathway and p38 MAPK prevented the ectodomain shedding of TNF-R1 in response to translation inhibitors [22,23,24,25]. The present study demonstrated that U0126, an inhibitor of MEK, but not SB203580 or SP600125, inhibitors of p38 MAPK and JNK, respectively, suppressed TNF-R1 ectodomain shedding in cucurbitacin B-treated cells. This is consistent with our additional results showing that cucurbitacin B induced the rapid and potent activation of ERK, while exerting minimal effects on p38 MAPK or JNK. In addition to ADAM17 [14,15,17], TNF-R1 has been identified as a substrate of ADAM8 and ADAM10 [44,45,46,47]. ADAM10 and ADAM17 are reportedly activated through the dissociation of tissue inhibitor of metalloprotease 3 following the phosphorylation of intracellular threonine residues [50,77]. Taken together, the present results suggest a mechanism by which cucurbitacin B selectively activates ERK and thereby promotes TNF-R1 shedding through the activation of ADAM proteases (Figure 11). However, additional studies will be required to identify the specific metalloproteases involved and to clarify the mechanisms underlying ERK-dependent metalloprotease activation.

In various cancer cells, the constitutive activation of the ERK pathway contributes to cell growth and survival [10]. Previous studies demonstrated that cucurbitacin B suppressed ERK activation in human glioblastoma, leukemia, neuroblastoma, osteosarcoma, and non-small cell lung cancer cells [78,79,80,81,82]. In addition, stimulation-dependent ERK activation was reduced by cucurbitacin B in phorbol ester-treated human hepatocellular cancer HepG2 cells and TNF-α-treated human cervical carcinoma HeLa cells [83,84]. In A549 cells, cucurbitacins directly interact with epidermal growth factor receptor (EGFR), thereby suppressing EGFR-dependent ERK activation [85,86,87]. These findings indicate that cucurbitacins frequently inhibit ERK signaling in cancer cells.

In contrast, other studies reported that cucurbitacin B increased phospho-ERK levels in human lung carcinoma, human hepatocellular carcinoma, human pancreatic cancer, and rat pheochromocytoma [88,89,90,91]. In the present study, cucurbitacin B at 10 µM rapidly increased phospho-ERK1/2 levels in A549 cells, reaching maximal levels within 20–40 min, followed by a decline after 60–90 min. In contrast, phospho-p38 MAPK and phospho-JNK levels were minimally affected during the same time period. Consistent with these results, RAF1 phosphorylation markedly increased 20 min after exposure to cucurbitacin B. Collectively, these results suggest that cucurbitacin B induced the rapid and transient activation of the RAF1-MEK-ERK pathway in A549 cells, which may contribute to TNF-R1 ectodomain shedding. Previous studies also showed that lower concentrations of cucurbitacin B (0.3 µM) delayed ERK activation to later time points [90,91]. Therefore, the effects of cucurbitacins on ERK signaling may vary depending on their concentration, exposure time, and cellular context.

The excessive production of reactive oxygen species (ROS) causes oxidative stress and activates the ERK signaling pathway [92]. Cucurbitacins B, E, and I have been reported to increase intracellular ROS production [93,94,95,96,97,98]. Moreover, NAC was shown to attenuate cell cycle arrest, apoptosis, autophagy, and metastasis induced by cucurbitacin B [94,95,97,98]. NAC also reversed cucurbitacin I-induced autophagy and ERK activation [93], as well as cucurbitacin E-induced apoptosis and autophagy [96]. In addition, we previously demonstrated that NAC prevented the inhibitory effects of cucurbitacin B on TNF-α-induced gene expression [39]. Taken together, these findings suggest that ROS production contributes to ERK activation induced by cucurbitacin B.

## 4. Materials and Methods

### 4.1. Cells

Human lung adenocarcinoma A549 cells (JCRB0076) were obtained from the JCRB Cell Bank at the National Institutes of Biomedical Innovation, Health and Nutrition (Osaka, Japan). Human embryonic kidney 293T cells (RCB2202) were obtained from the RIKEN BioResource Research Center, Tsukuba, Japan. A549 cells and 293T cells were routinely subcultured two to three times per week in Roswell Park Memorial Institute (RPMI) 1640 medium (Thermo Fisher Scientific, Grand Island, NY, USA) and Dulbecco’s Modified Eagle Medium (DMEM) (Thermo Fisher Scientific), respectively, supplemented with Penicillin-Streptomycin Mixed Solution (Stabilized) (Nacalai Tesque, Kyoto, Japan) and inactivated fetal bovine serum (Nichirei Bioscience, Tokyo, Japan).

### 4.2. Reagents

Small-molecule compounds were obtained from commercial suppliers: bufalin (029-16981; Wako Pure Chemical Industries, Osaka, Japan), CDDO (Bardoxolone) (81035; Cayman Chemical Company, Ann Arbor, MI, USA), celastrol (70950; Cayman Chemical Company), cucurbitacin B (C3442; Tokyo Chemical Industry Co., Ltd., Tokyo, Japan), glycyrrhetinic acid (also referred to as glychyrrhetic acid) (G0149; Tokyo Chemical Industry Co., Ltd.), pristimerin (13621; Cayman Chemical Company), SB203580 (13067; Cayman Chemical Company), SP600125 (197-16591; Fujifilm Wako Pure Chemical Corporation, Osaka, Japan), U0126 (211-01051; Wako Pure Chemical Industries), TAPI-2 (INH-3852-PI; Peptide Institute Inc., Osaka, Japan), withaferin A (WT; Fermentek, Jerusalem, Israel), and withanolide B (11798; Cayman Chemical Company). Recombinant GST and GST-TNF-α were prepared in our previous study [43].

### 4.3. Antibodies

The primary antibodies used for Western blotting were as follows: an anti-ERK antibody (137F5; Cell Signaling Technology, Danvers, MA, USA), anti-phospho-ERK antibody (Thr202/Tyr204) (#9101; Cell Signaling Technology), anti-JNK antibody (#9252; Cell Signaling Technology), anti-phospho-JNK antibody (Thr183/Tyr185) (#9251; Cell Signaling Technology), anti-p38 MAPK antibody (#9212; Cell Signaling Technology), anti-phospho-p38 MAPK antibody (Thr180/Tyr182) (D3F9; Cell Signaling Technology), anti-RAF1 antibody (D5X6R; Cell Signaling Technology), anti-phospho-RAF1 antibody (Ser289/296/301) (#9431; Cell Signaling Technology), anti-TNF-R1 antibody (C25C1; Cell Signaling Technology), anti-TNF-R1 antibody (H-5; Santa Cruz Biotechnology, Dallas, TX, USA), and anti-glyceraldehyde-3-phosphate dehydrogenase (GAPDH) antibody (sc-32233; Santa Cruz Biotechnology). The secondary antibodies used were as follows: peroxidase-conjugated goat anti-mouse IgG (H+L) (115-035-146; Jackson ImmunoResearch Laboratories, West Grove, PA, USA) and peroxidase-conjugated goat anti-rabbit IgG (H+L) (111-035-144; Jackson ImmunoResearch Laboratories).

### 4.4. Cell Viability

A549 cells were seeded on 96-well plates, cultured overnight, and then treated with compounds in RPMI 1640 medium. Cell viability was evaluated by crystal violet staining as described in our previous studies [99,100]. Absorbance at 570 nm was measured using the iMark microplate reader (Bio-Rad Laboratories, Hercules, CA, USA).

### 4.5. Preparation of Protein Samples

A549 cells and 293T cells were seeded on 35 mm dishes, cultured overnight, and then treated with compounds in serum-free RPMI 1640 medium or serum-free DMEM. Protein samples from the culture medium were prepared as previously described [101]. Briefly, the culture medium was collected, mixed with methanol and chloroform, and centrifuged to obtain protein precipitates. Whole-cell lysates were prepared as previously described [102]. Cell pellets were rinsed and lyzed with Triton X-100 lysis buffer consisting of the phosphatase inhibitor cocktail (Nacalai Tesque) and the protease inhibitor cocktail cOmplete (Merck, Darmstadt, Germany). Lysed cells were subjected to repeated sonication and centrifugation to prepare whole-cell lysates.

### 4.6. Western Blotting

Western blotting was performed on the protocols described in our previous studies [99,100,101]. Protein samples were separated by sodium dodecyl sulfate-polyacrylamide gel electrophoresis (SDS-PAGE) and transferred onto nitrocellulose membranes. Membranes were blocked overnight in skim milk and then incubated with primary antibodies, followed by peroxidase-conjugated secondary antibodies. Immunoreactive bands were detected by chemiluminescent reactions. Blot images were acquired using an Amersham Imager 680 (GE Healthcare Japan, Tokyo, Japan). Membranes were then stripped using Stripping Solution (Fujifilm Wako Chemical Corporation, Osaka, Japan) for normalization and reprobed with loading control antibodies. Protein bands were quantified using ImageQuant TL software version 7.0.1.0 (GE Healthcare Japan).

### 4.7. ELISA

The Duoset^TM^ ELISA Development System for Human TNF RI/TNFRSF1A (DY225-05, R&D Systems, Inc., Minneapolis, MN, USA) was used. The culture medium was collected after centrifugation. The amount of TNF-R1 was measured according to the manufacturer’s protocol. *o*-Phenylenediamine and hydrogen peroxide were used as substrates for peroxidases. Absorbance at 450 nm was measured using the iMark microplate reader. Standard curves were used to assess TNF-R1 concentrations.

### 4.8. GST Pull-Down Assay

GST pull-down assays were conducted as previously described [43], with some modifications. Cells were incubated with or without compounds, and then incubated with GST or GST-TNF-α fusion proteins on ice for 5 min. Cells were washed with phosphate-buffered saline and lysed using DISC lysis buffer for 15 min, followed by centrifugation. The resultant supernatants were incubated with Glutathione Sepharose 4B for 1 h. Pull-down products were washed three times with DISC lysis buffer and analyzed by SDS-PAGE and Western blotting.

### 4.9. Caspase-3/7 Activity Assay

The Caspase-Glo^®^ 3/7 Assay System (G8090, Promega Corporation, Madison, WI, USA) was used to assess caspase-3/7 activity. Cells were lysed using digitonin lysis buffer and centrifuged to prepare cytosolic cell lysates. Caspase-3/7 activity was measured according to the manufacturer’s protocol. Relative light units were measured using Lumitester C-110 (Kikkoman Biochemifa, Tokyo, Japan).

### 4.10. LDH Release Assay

The LDH Cytotoxicity Assay Kit (18250-64, Nacalai Tesque) was used to assess LDH activity. Cells were incubated with compounds for 2 h, after which the cell medium and total cell lysates were collected and assayed for LDH activity. Absorbance at 490 nm was measured using the iMark microplate reader.

### 4.11. Data and Statistical Analyses

Experiments were independently performed at least three times. Data were analyzed using Welch’s *t*-tests or a one-way analysis of variance followed by Tukey’s multiple comparisons using GraphPad Prism version 11.0.0 (GraphPad Software, Boston, MA, USA).

## 5. Conclusions

TNF-R1 exhibits a ubiquitous distribution and plays an essential role in TNF-α-dependent cellular responses. Multiple ADAM proteases are capable of cleaving TNF-R1. We herein demonstrated that cucurbitacin B selectively activated the ERK signaling pathway and promoted TNF-R1 ectodomain shedding in A549 cells. Therefore, cucurbitacin B may be useful for blocking TNF-α-dependent cellular responses. However, cucurbitacin B is capable of inducing ERK activation, which regulates essential cellular processes, such as cell proliferation, survival, growth, differentiation, and metabolism. Taken together, cucurbitacin B-induced ERK activation may cause side effects in addition to its therapeutic anti-inflammatory and anticancer effects. Future investigations are needed to clarify the mechanisms by which cucurbitacin B induces ERK activation and promotes the activation of ADAM proteases involved in TNF-R1 shedding.

## Data Availability

The data presented in this study are available upon reasonable request from the corresponding author.

## Supplementary Materials

The following supporting information can be downloaded at: https://www.mdpi.com/article/10.3390/ijms27115011/s1.

## Abbreviations

The following abbreviations are used in this manuscript:

ADAM	a disintegrin and metalloprotease
CDDO	2-cyano-3,12-dioxooleana-1,9-dien-28-oic acid
DMEM	Dulbecco’s modified Eagle medium
EGFR	epidermal growth factor receptor
ELISA	enzyme-linked immunosorbent assay
ERK	extracellular signal-regulated kinase
GAPDH	glyceraldehyde-3-phosphate dehydrogenase
GST	glutathione *S*-transferase
JNK	c-Jun *N*-terminal kinase
LDH	lactate dehydrogenase
MAP	mitogen-activated protein
MAPK	MAP kinase
MAP2K	MAP kinase kinase
MAP3K	MAP kinase kinase kinase
MEK	MAPK/ERK kinase
NAC	*N*-acetylcysteine
NF-κB	nuclear factor κB
PMA	phorbol 12-myristate 13-acetate
RAF	rapidly accelerated fibrosarcoma
RAF1	rapidly accelerated fibrosarcoma 1
RAS	rat sarcoma
ROS	reactive oxygen species
RPMI	Roswell Park Memorial Institute
SDS-PAGE	sodium dodecyl sulfate-polyacrylamide gel electrophoresis
TAPI-2	TNF protease inhibitor 2
TCR	T-cell receptor
TLR	Toll-like receptor
TNF	tumor necrosis factor
TNF-α	tumor necrosis factor α
TNF-R1	TNF receptor 1
TNF-R2	TNF receptor 2
