# Supplementary material for: Cucurbitacin B Promotes Tumor Necrosis Factor Receptor 1 Ectodomain Shedding by Selectively Activating the Extracellular Signal-Regulated Kinase Signaling Pathway
Source: Int J Mol Sci. 2026 Jun 1;27(11):5011. doi: 10.3390/ijms27115011 (PMC13257231; doi:10.3390/ijms27115011)

# Figure S1: Original blots in Figure 2A (medium)

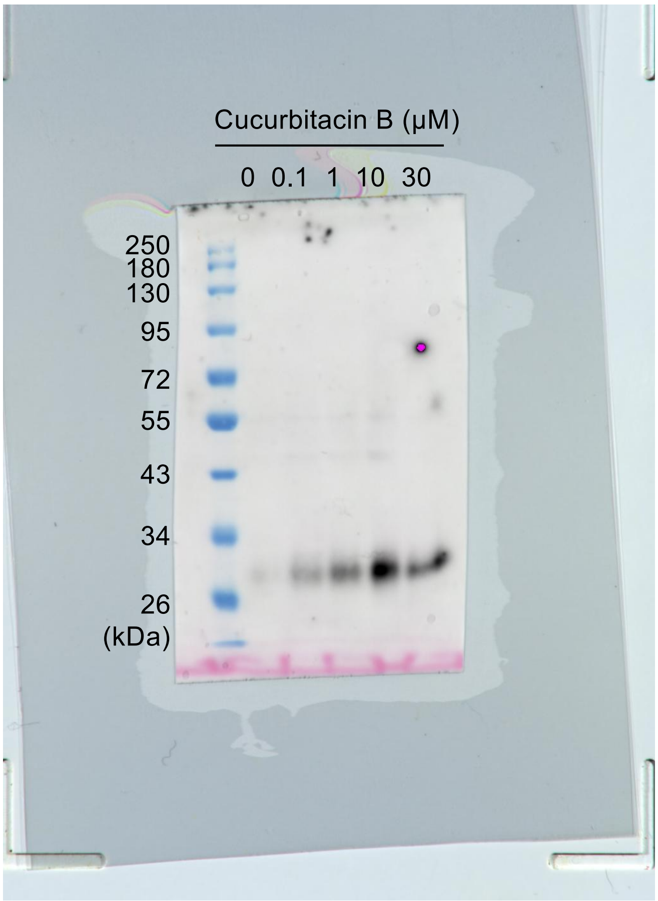


WB: TNF-R1

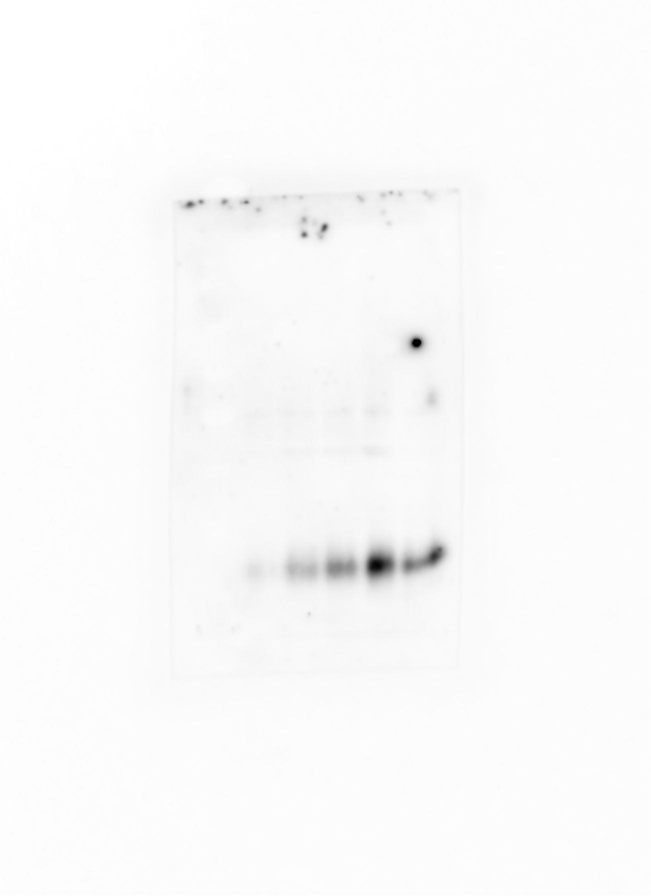

## Figure S2: Original bots in Figure 2A (cell lysate)

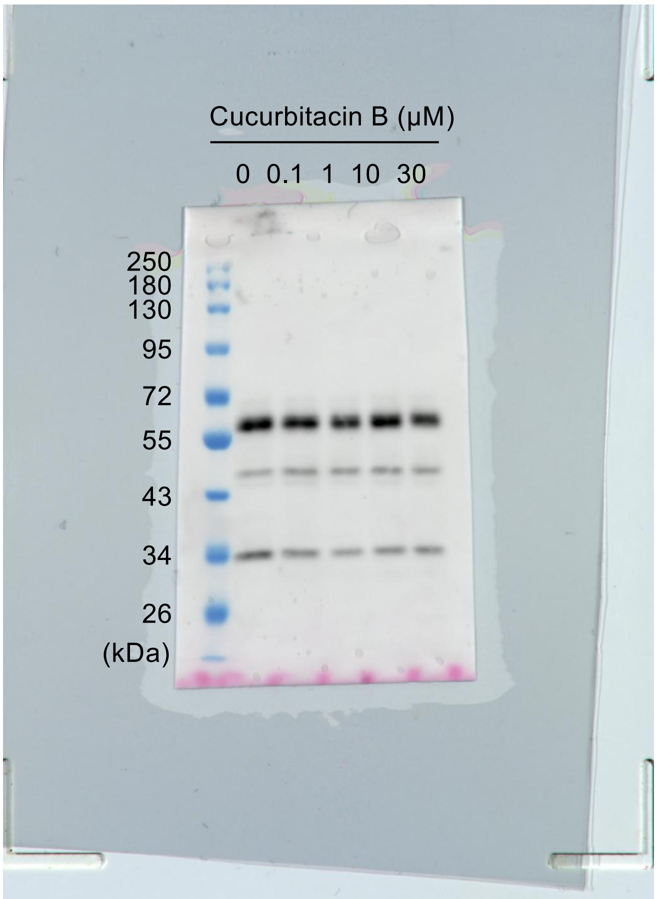


WB: TNF-R1

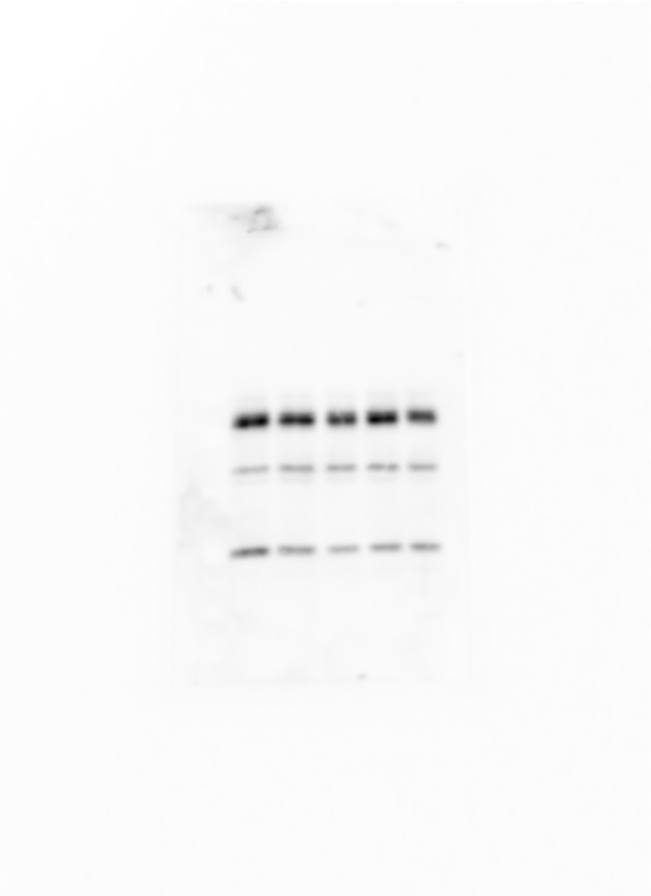

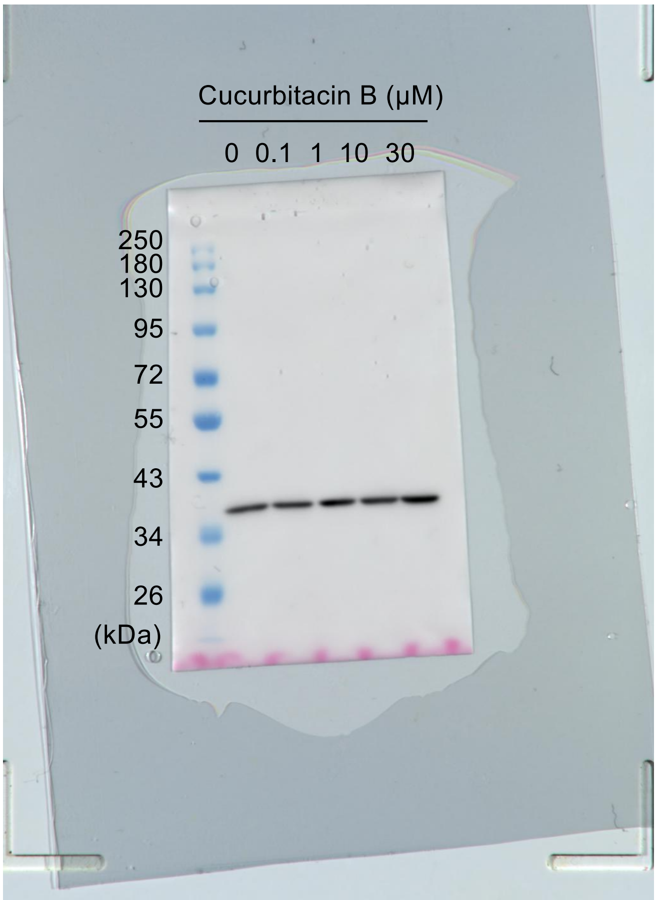


WB: GAPDH (reprobed)

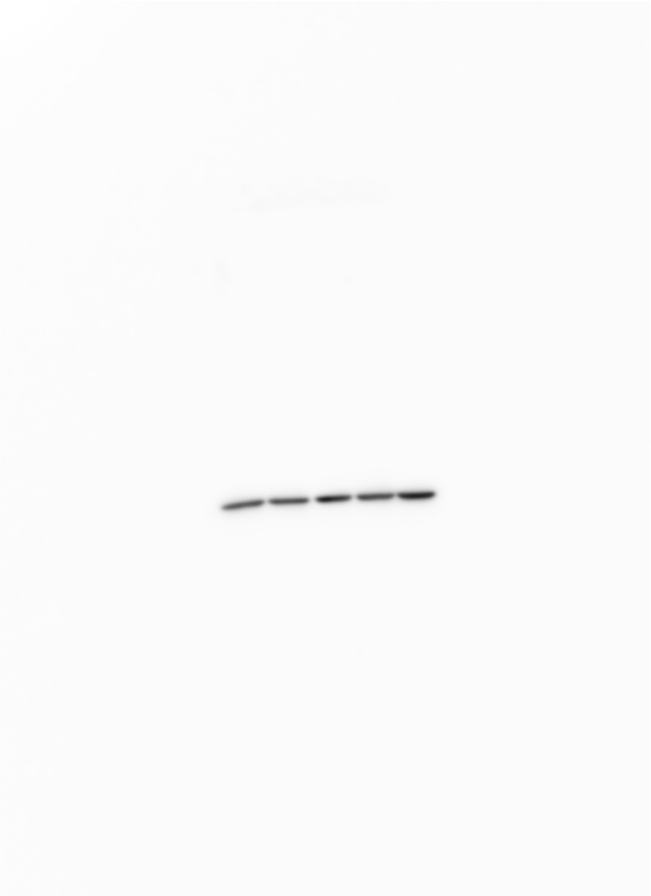

# Figure S3: Original blots (1) in Figure 2B

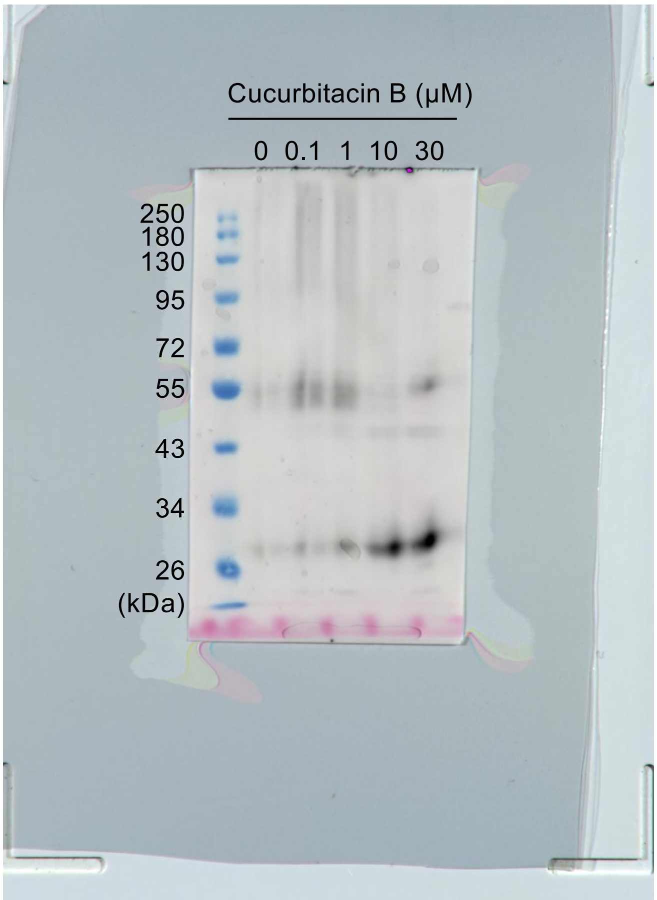


WB: TNF-R1

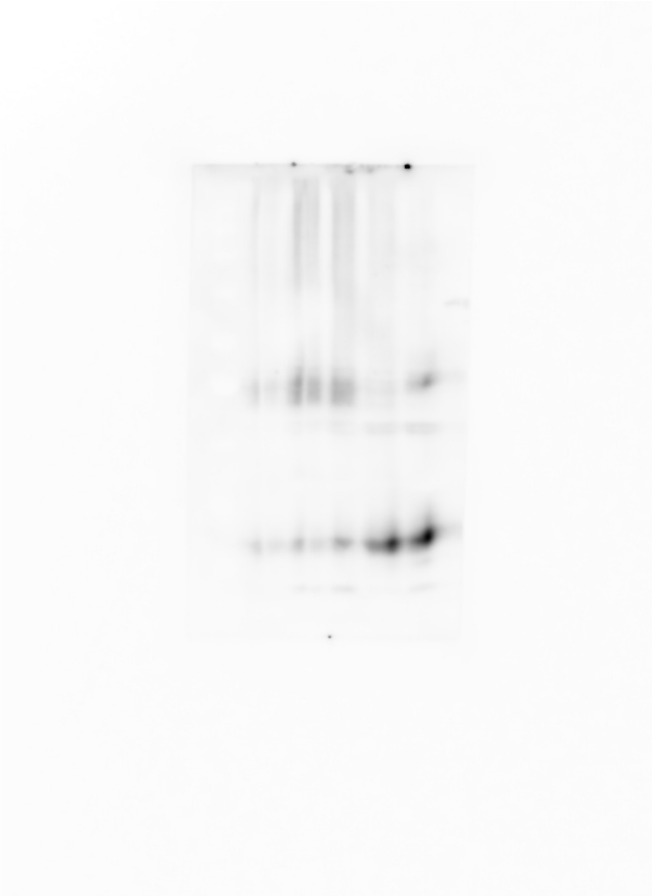

# Figure S4: Original blots (2) in Figure 2B

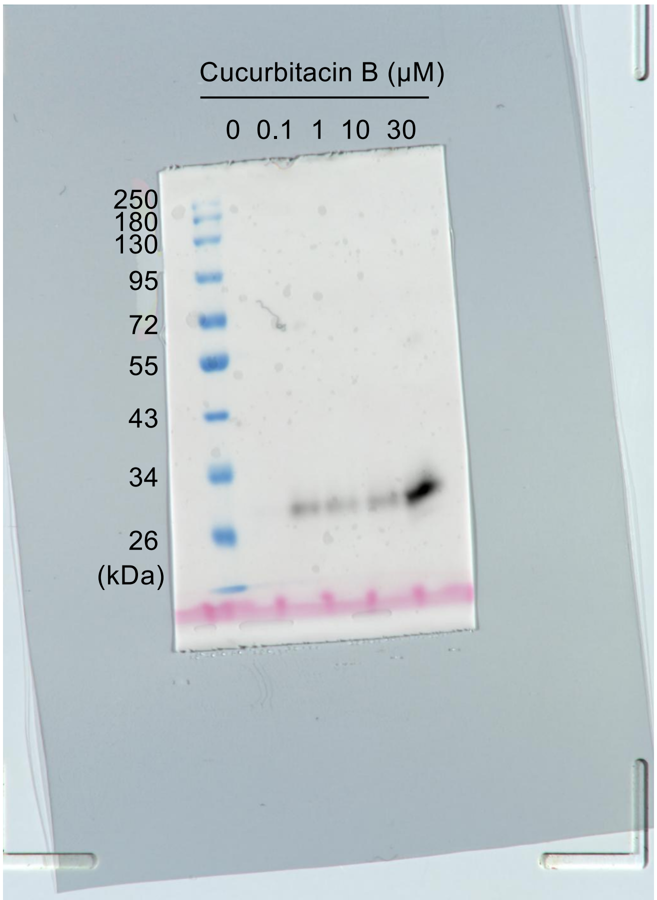


WB: TNF-R1

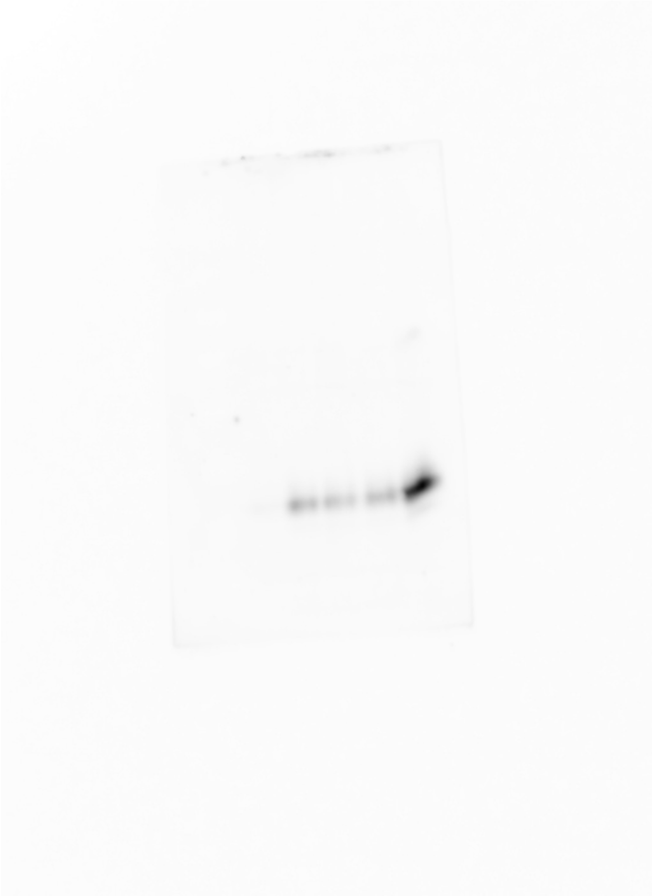

# Figure S5: Original blots (3) in Figure 2B

WB: TNF-R1

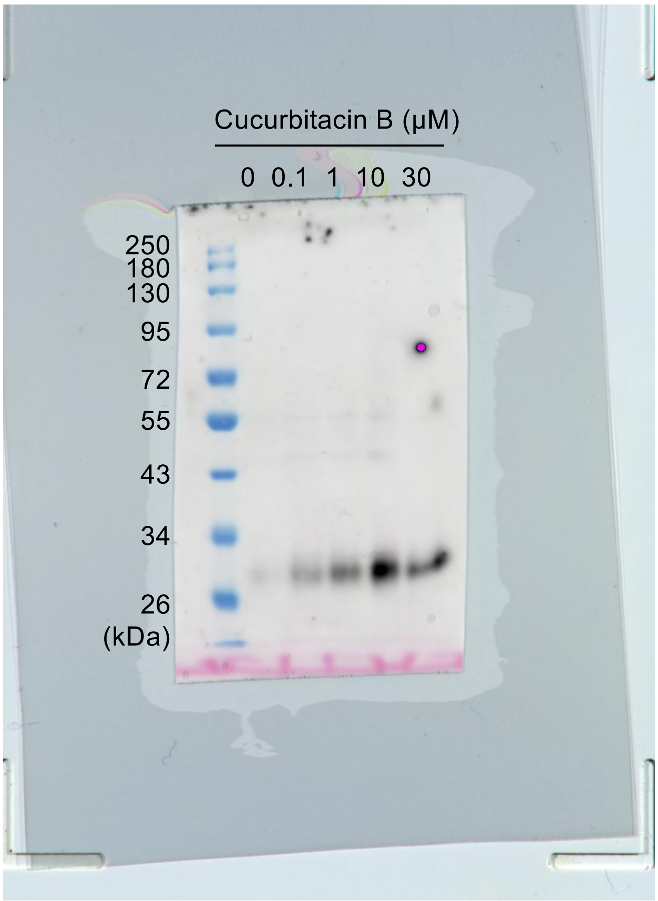


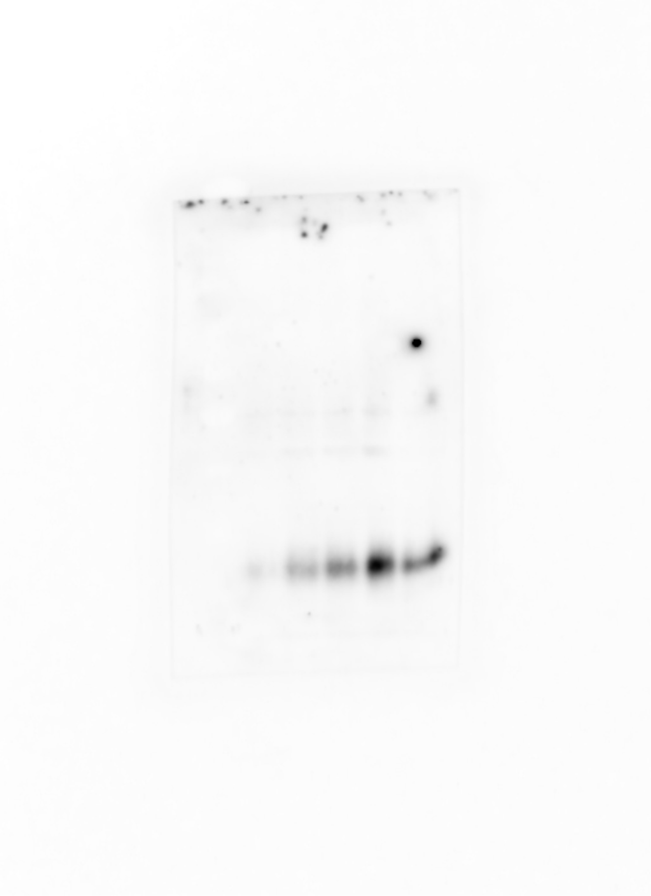

# Figure S6: Original blots (1) in Figure 2C

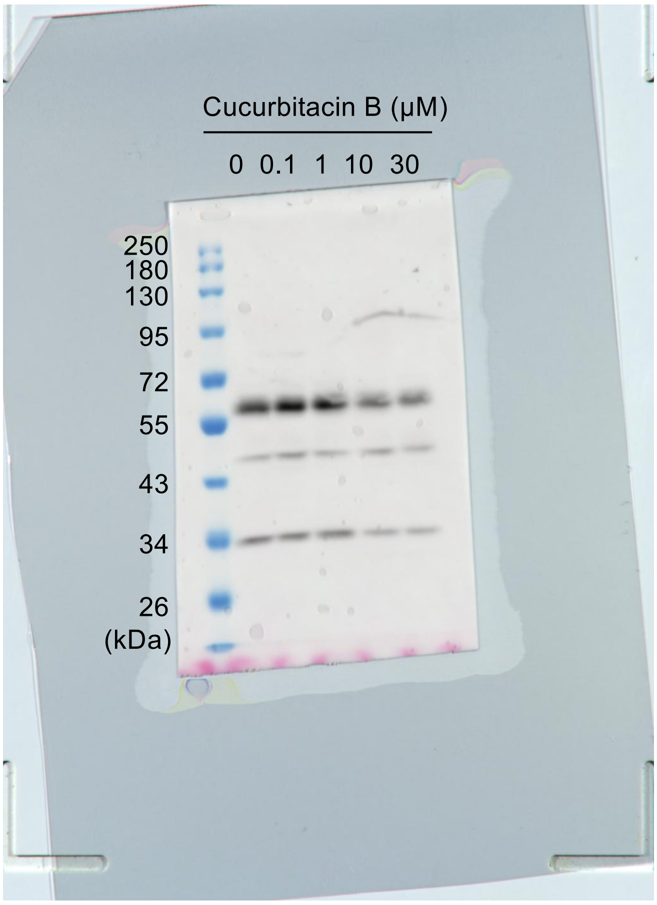


WB: TNF-R1

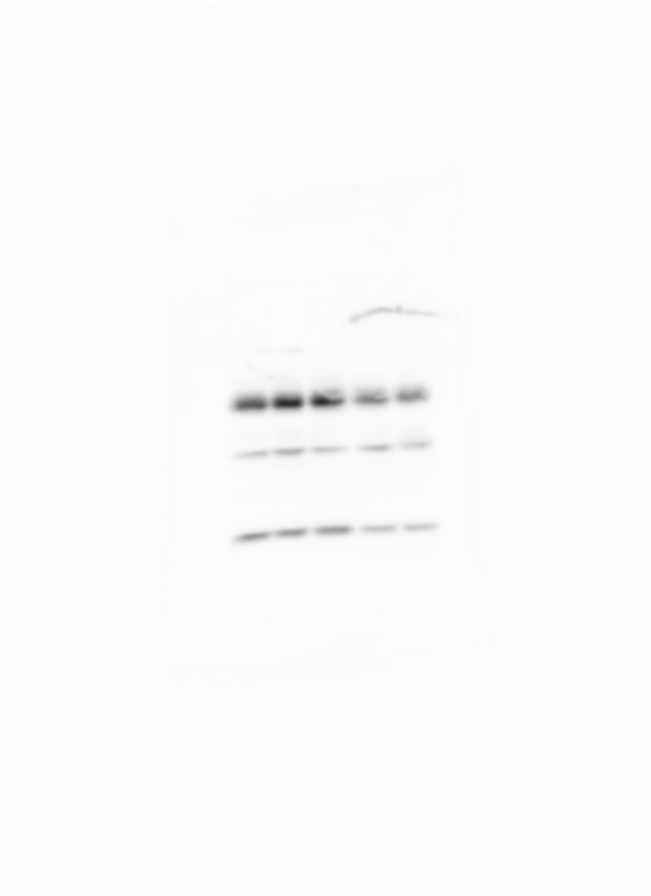

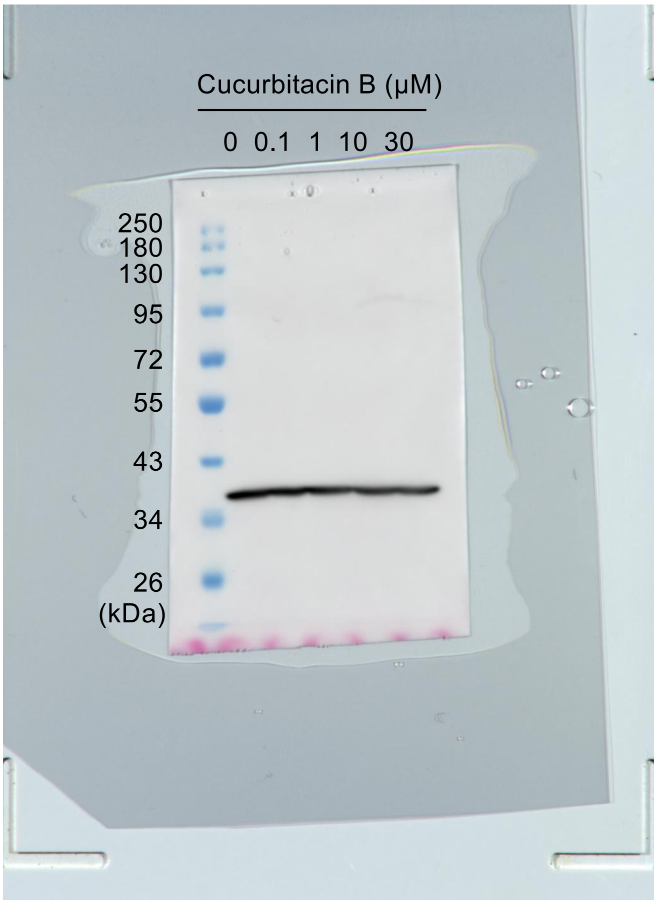


WB: GAPDH (reprobed)

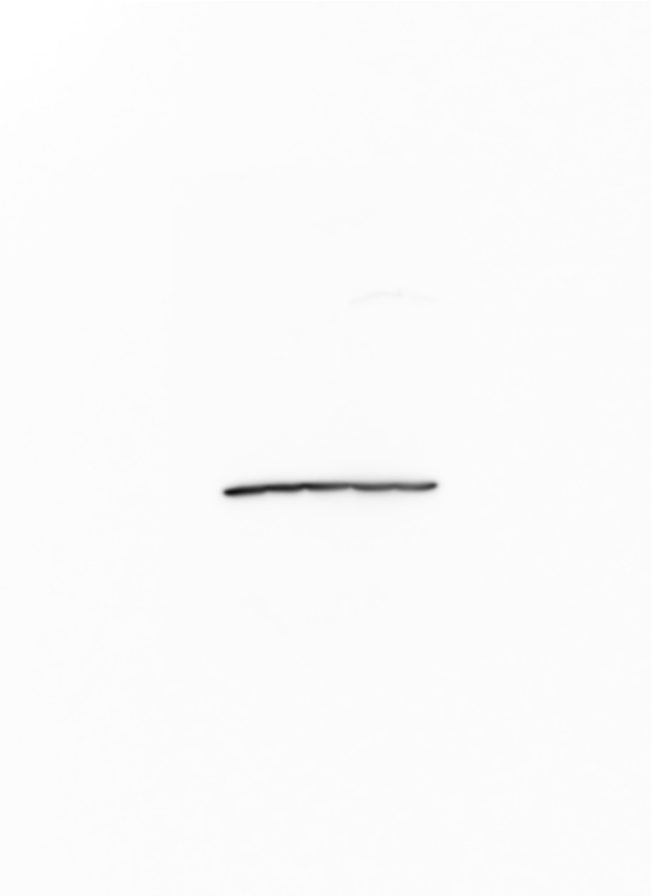

# Figure S7: Original blots (2) in Figure 2C

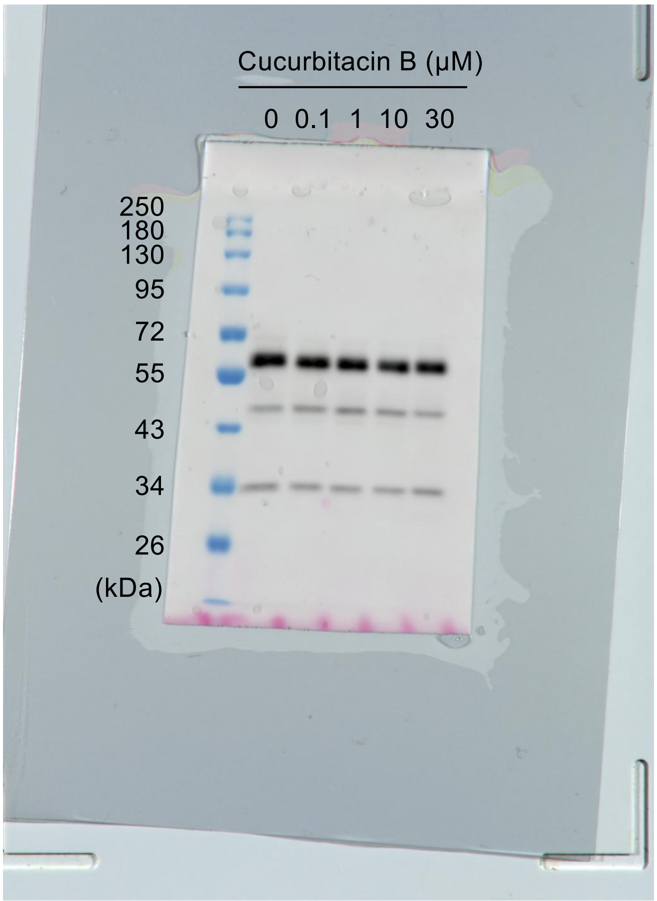


WB: TNF-R1

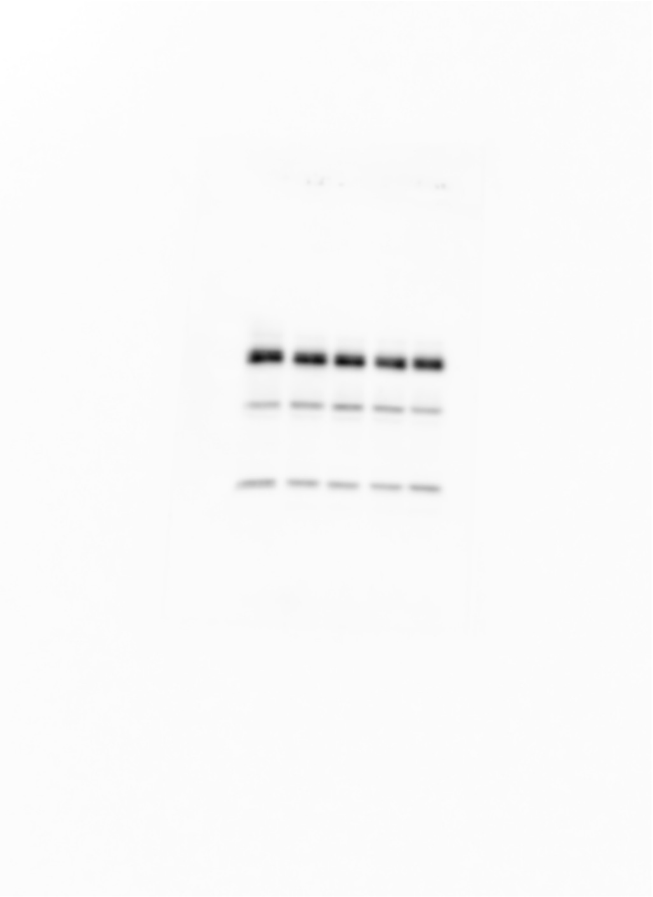

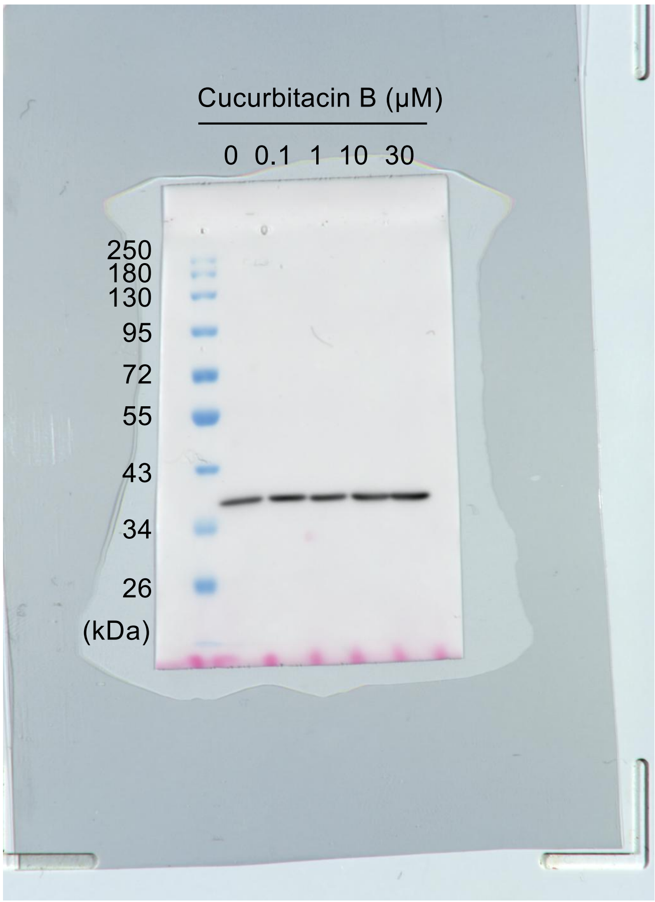


WB: GAPDH (reprobed)

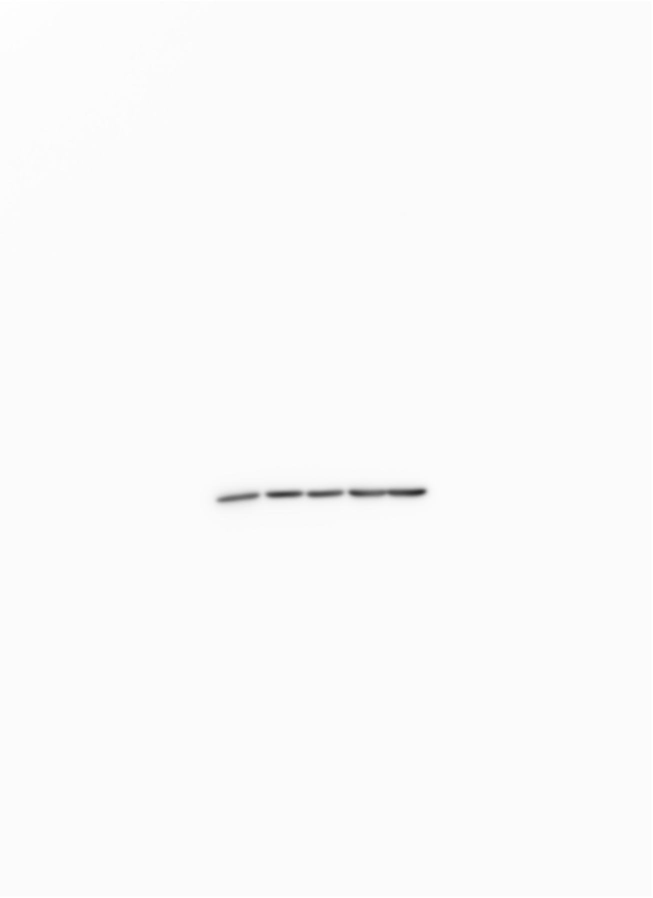

## Figure S8: Original blots (3) in Figure 2C

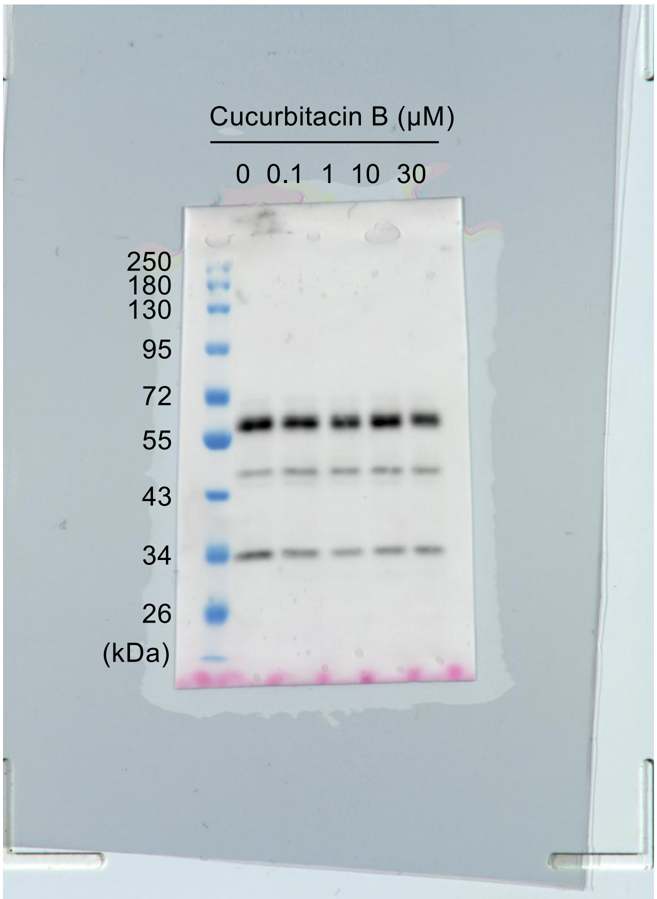


WB: TNF-R1

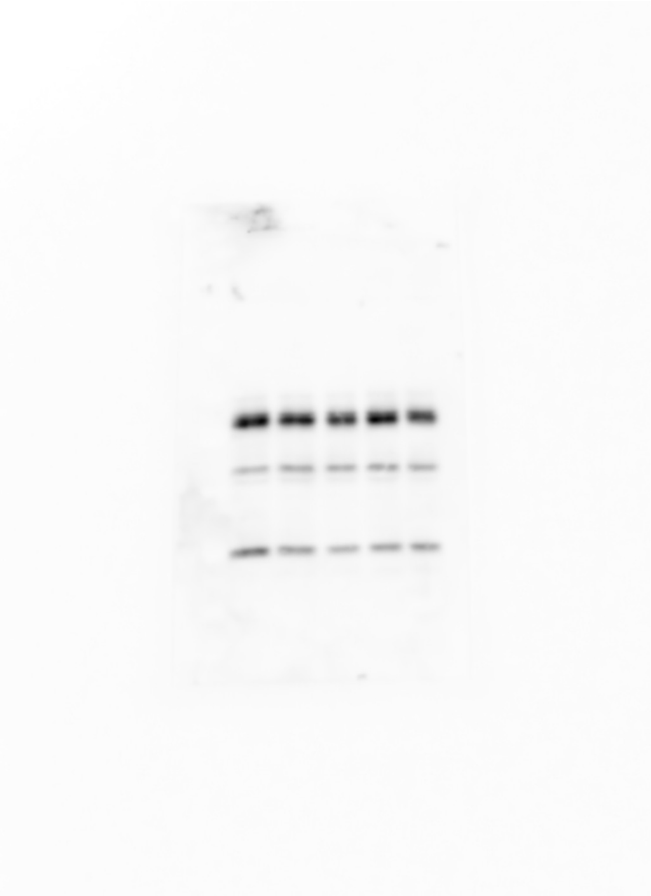

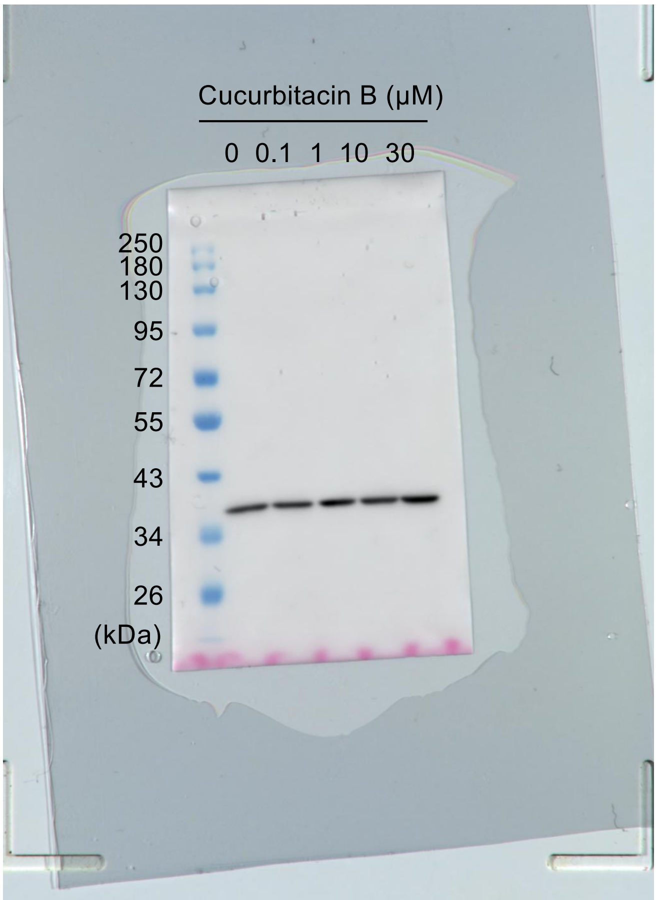


WB: GAPDH (reprobed)

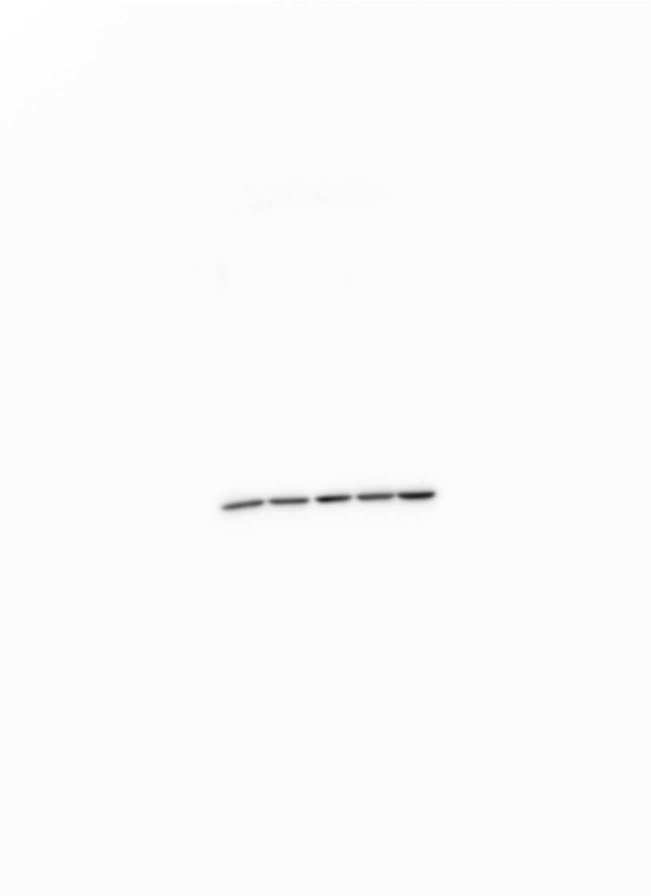

## Figure S9: Original blots in Figure 2E

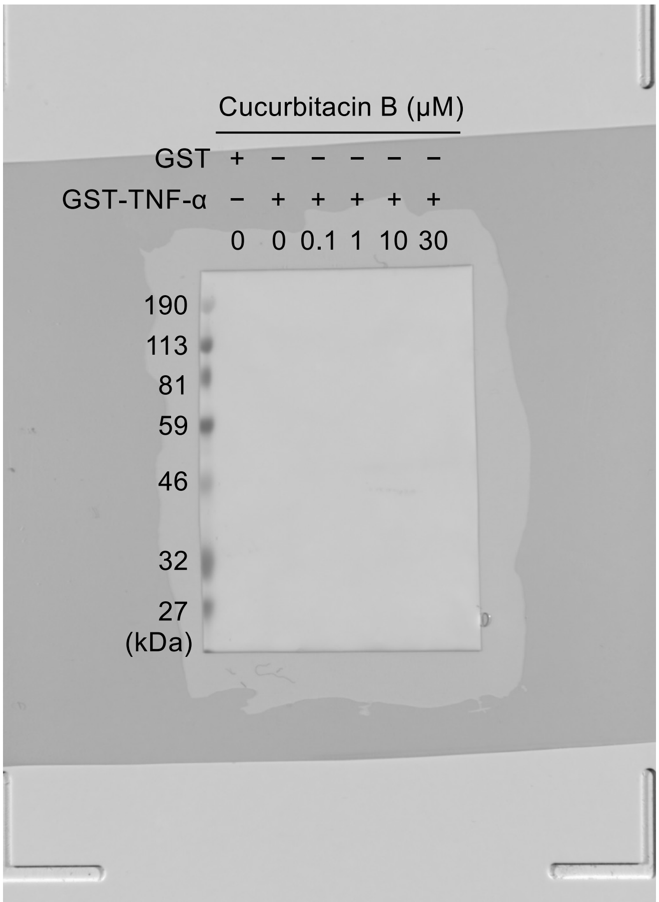


WB: TNF-R1

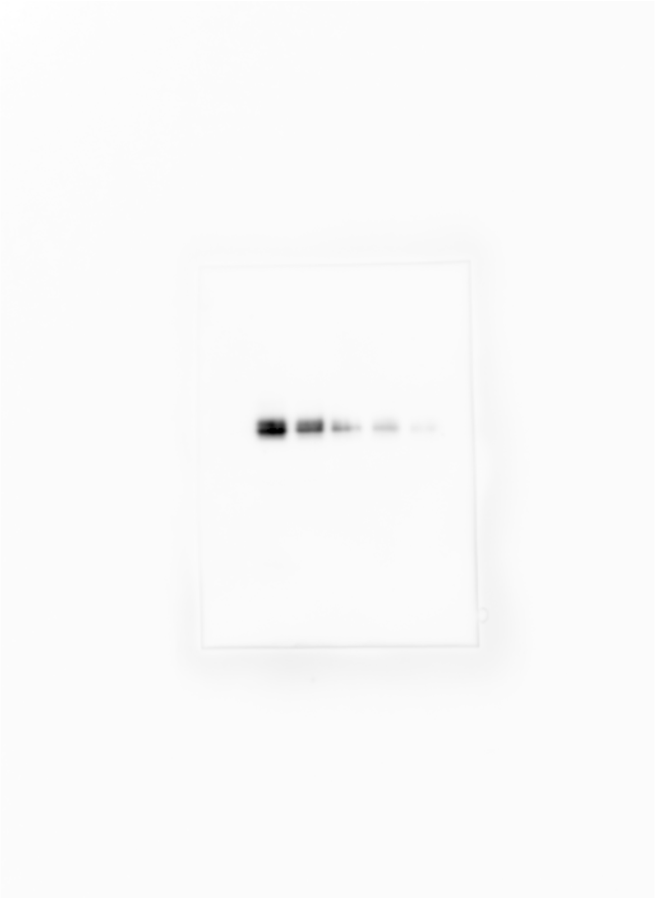

## Figure S10: Original blots (1) in Figure 2F

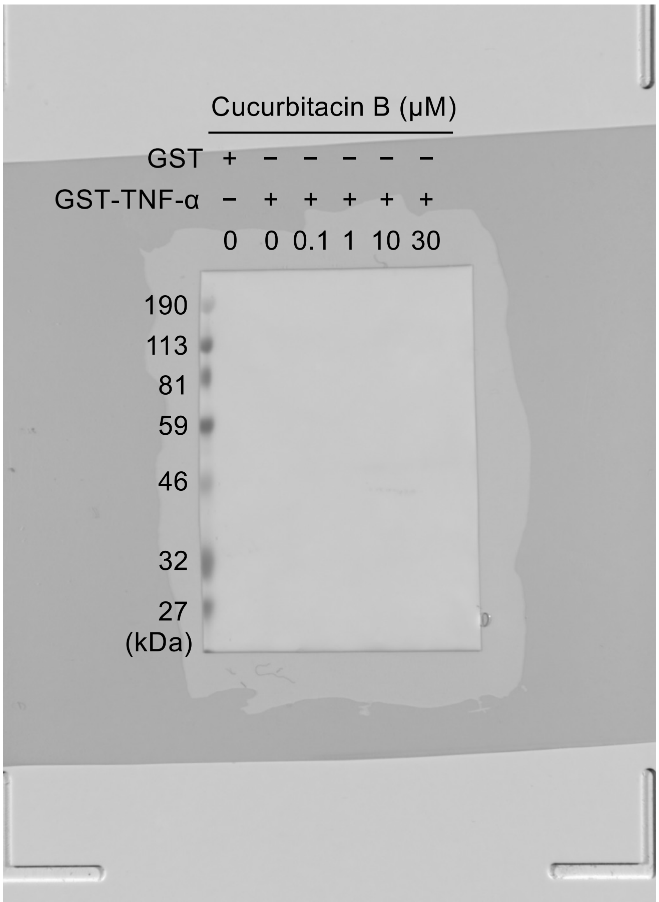


WB: TNF-R1

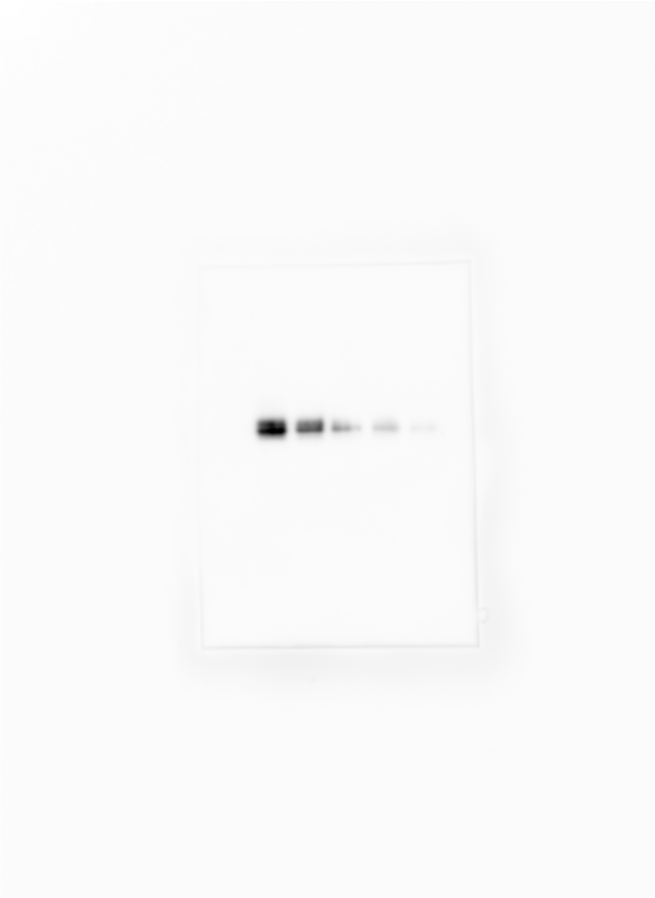

# Figure S11: Original blots (2) in Figure 2F

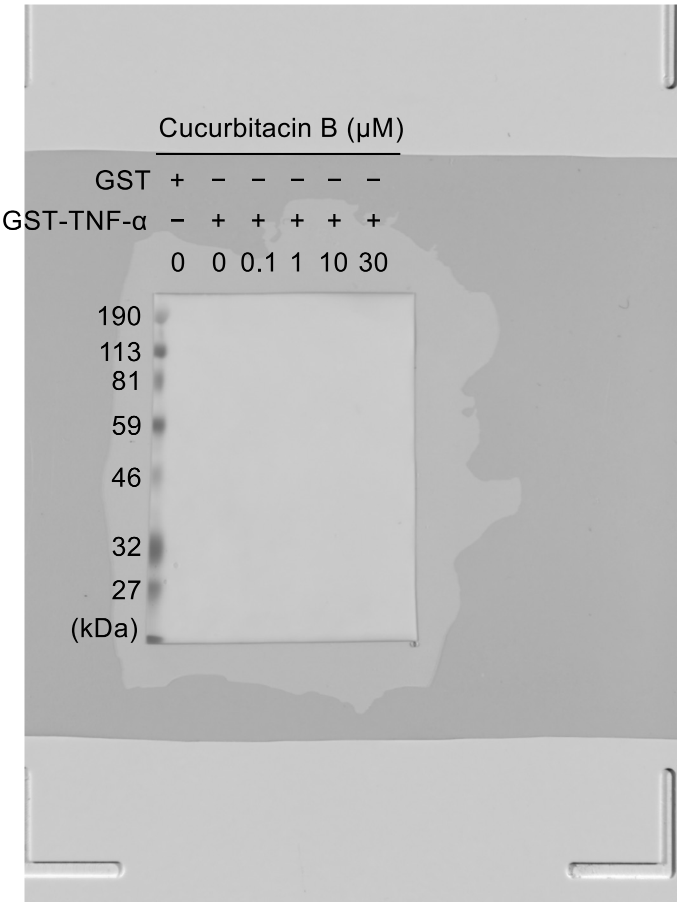


WB: TNF-R1

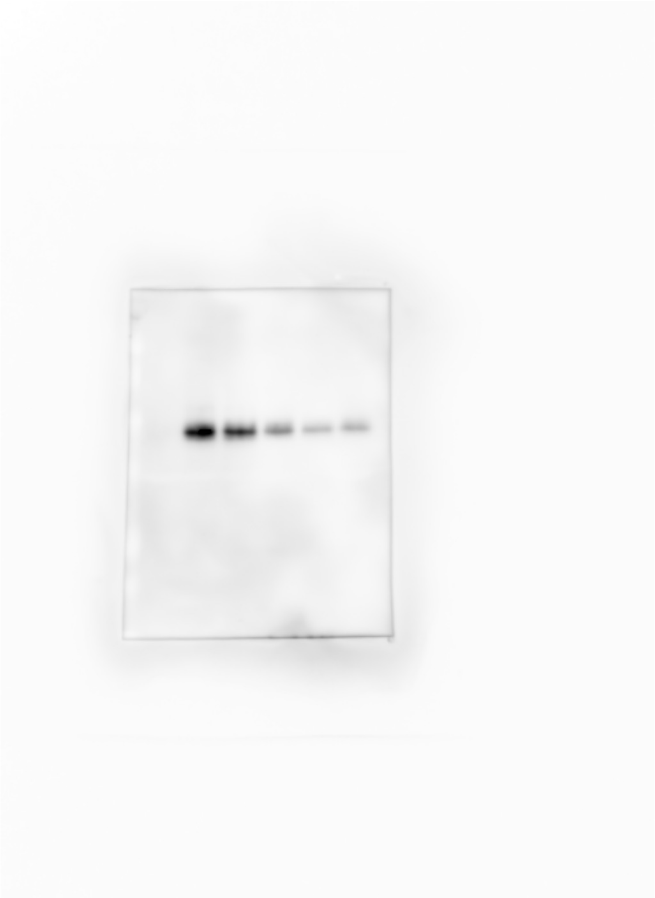

# Figure S12: Original blots (3) in Figure 2F

WB: TNF-R1

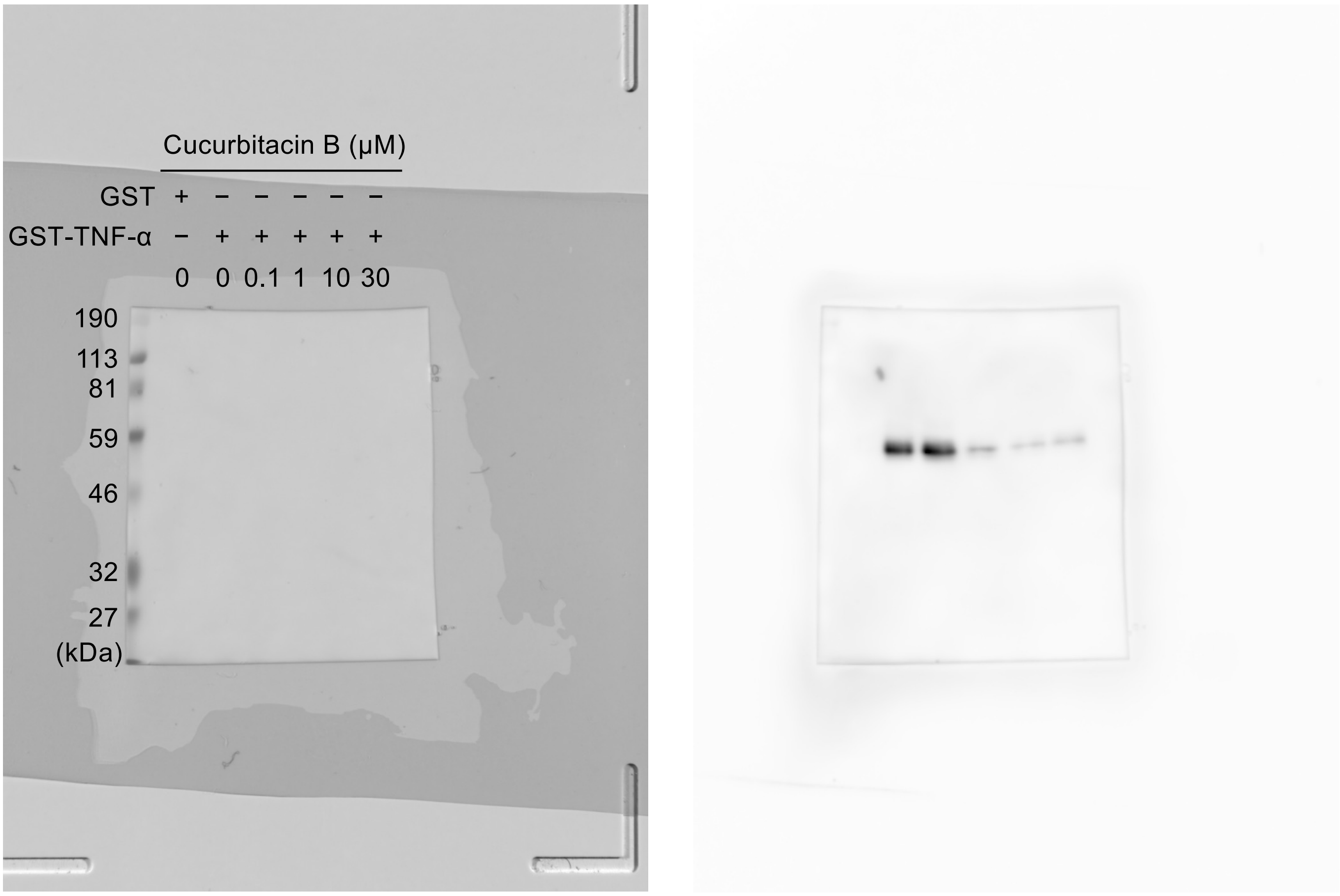

# Figure S13: Original blots in Figure 3A (medium)

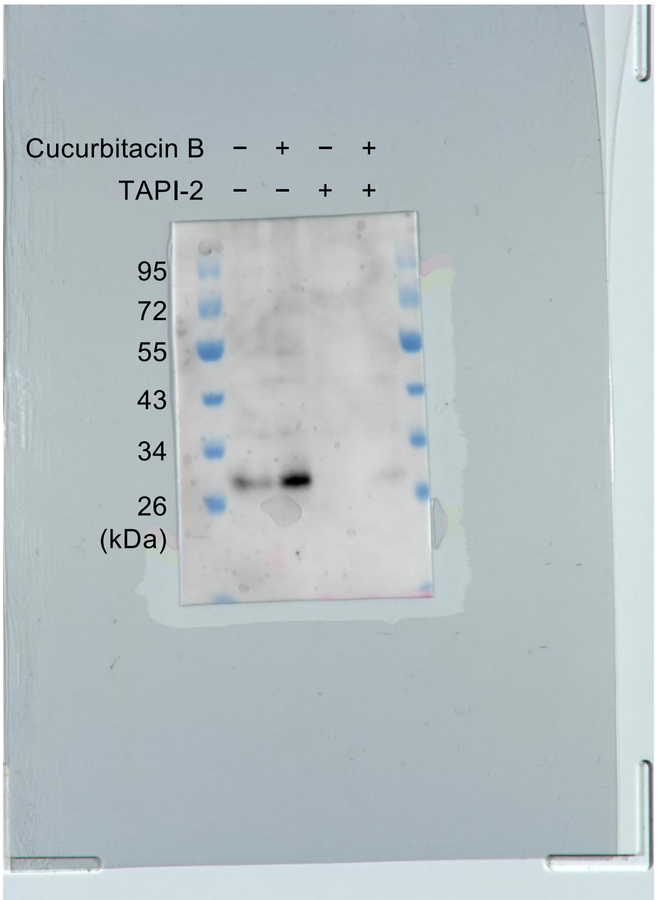


WB: TNF-R1

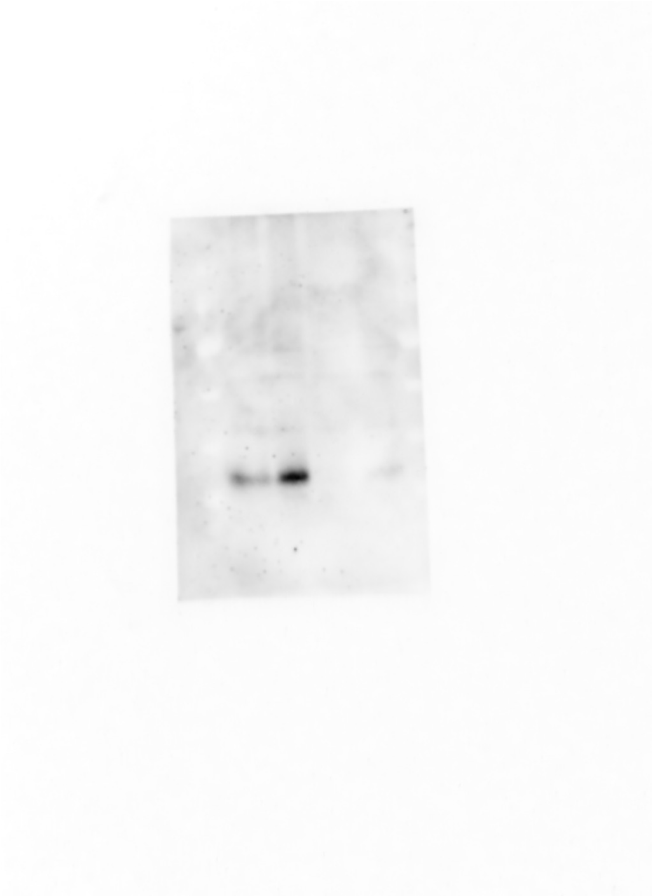

# Figure S14: Original blots in Figure 3A (cell lysate)

WB: TNF-R1

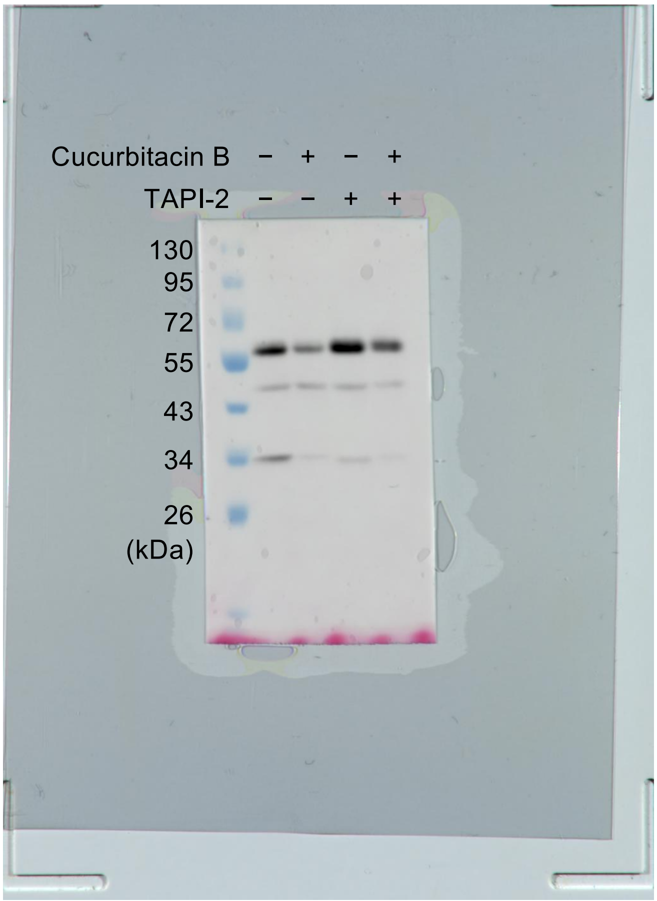


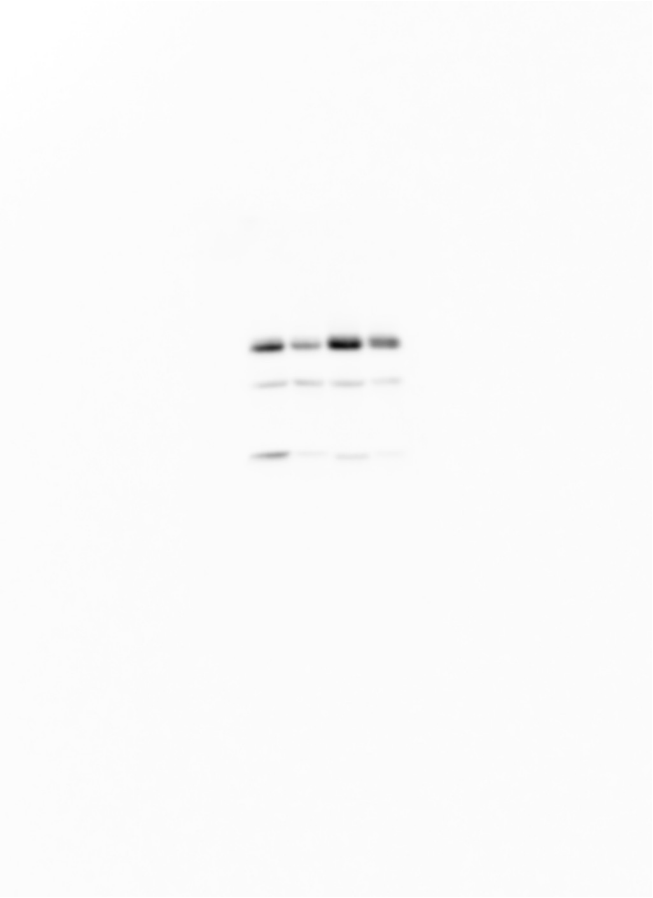

WB: GAPDH (reprobed)

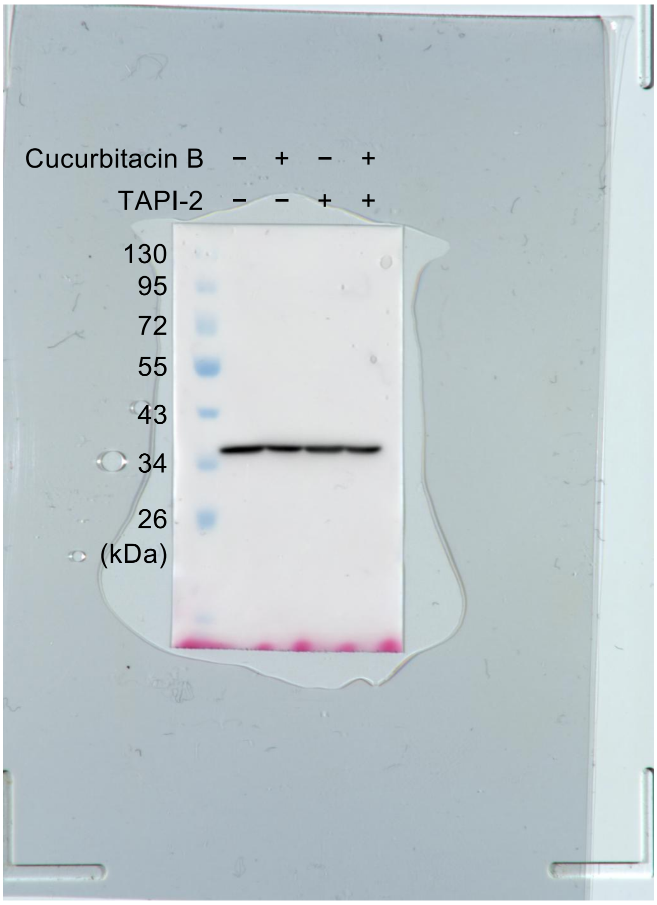


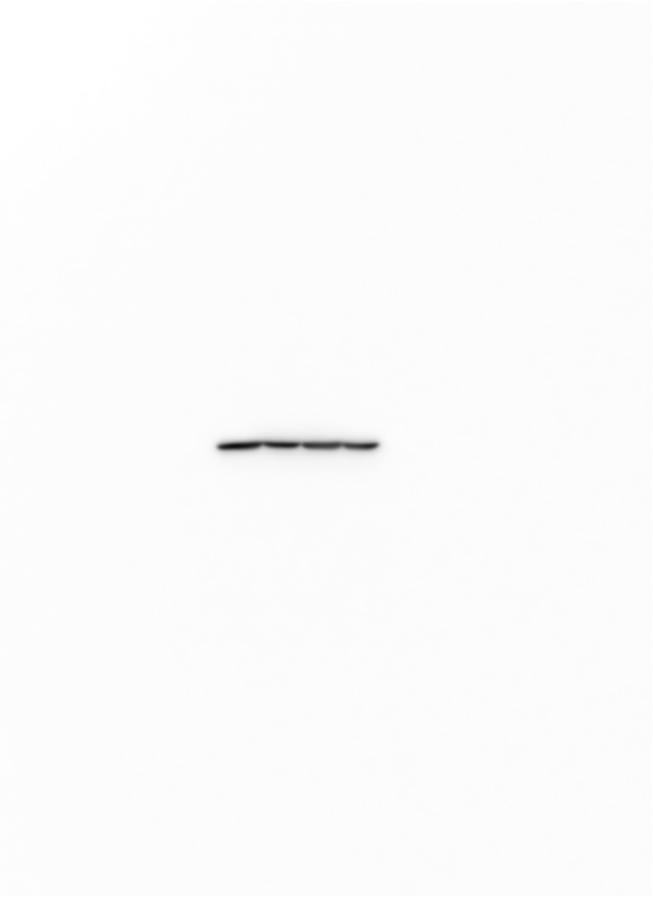

# Figure S15: Original blots (1) in Figure 3B

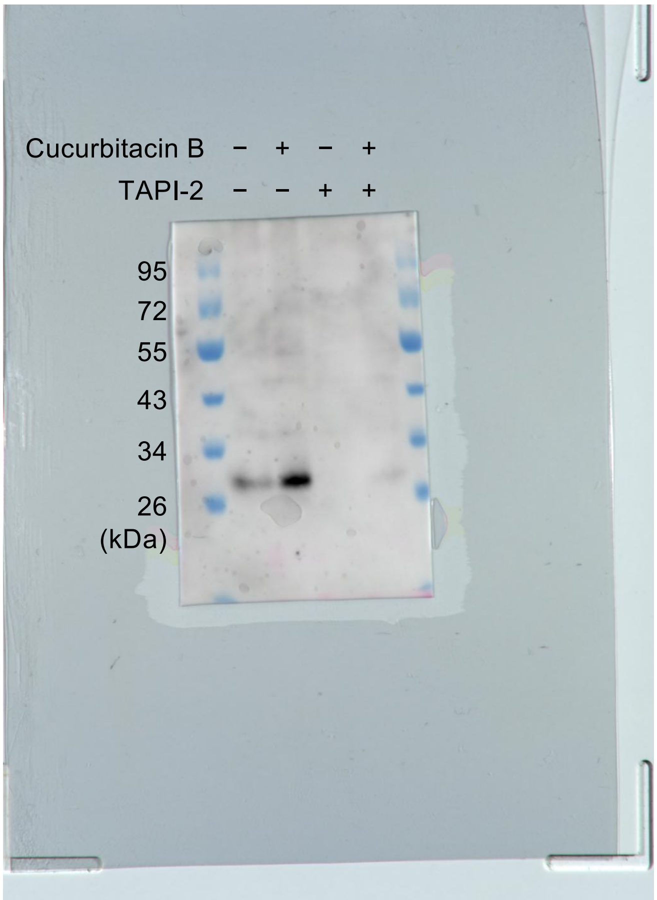


WB: TNF-R1

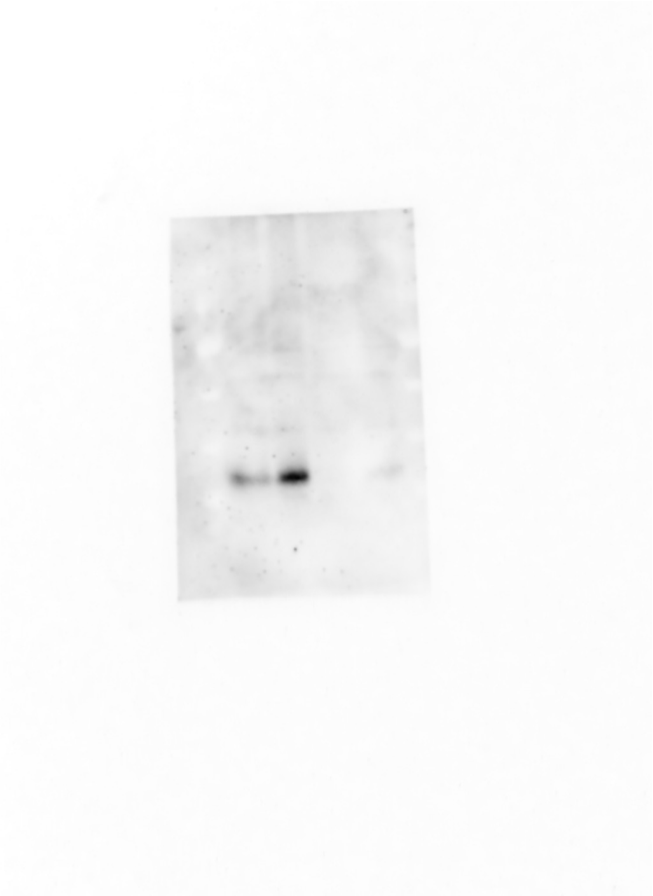

## Figure S16: Original blots (2) in Figure 3B

WB: TNF-R1

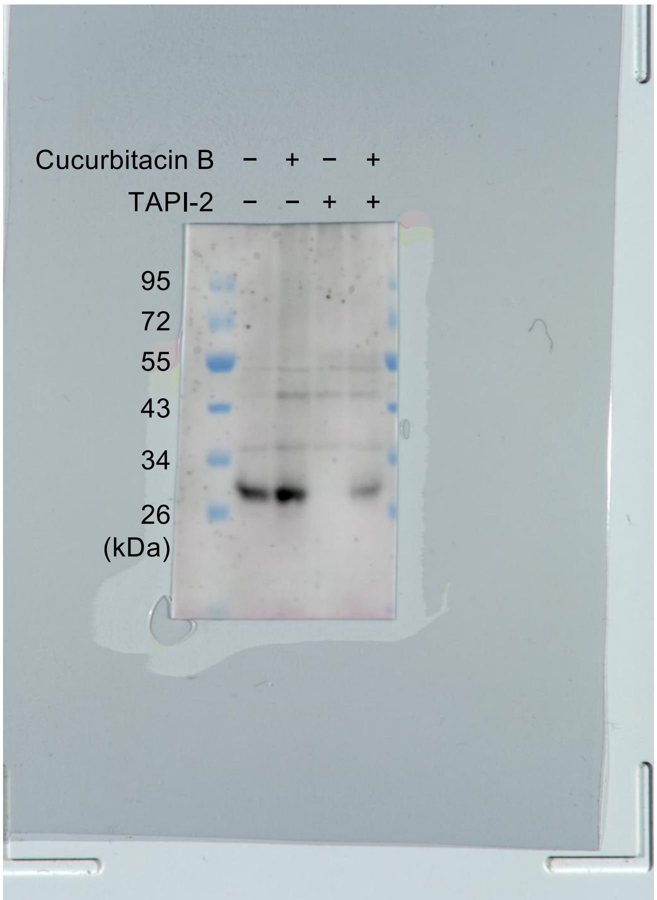


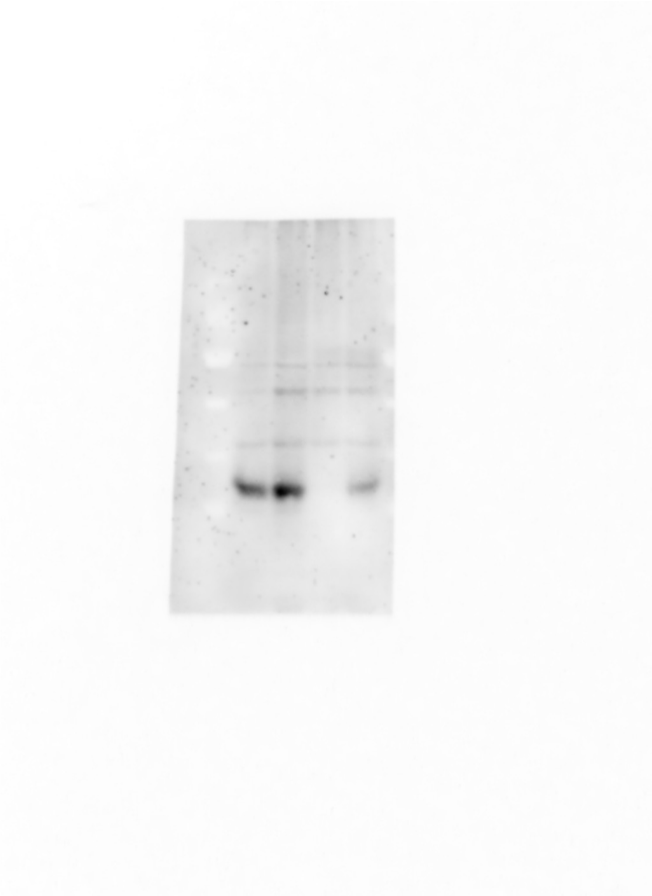

## Figure S17: Original blots (3) in Figure 3B

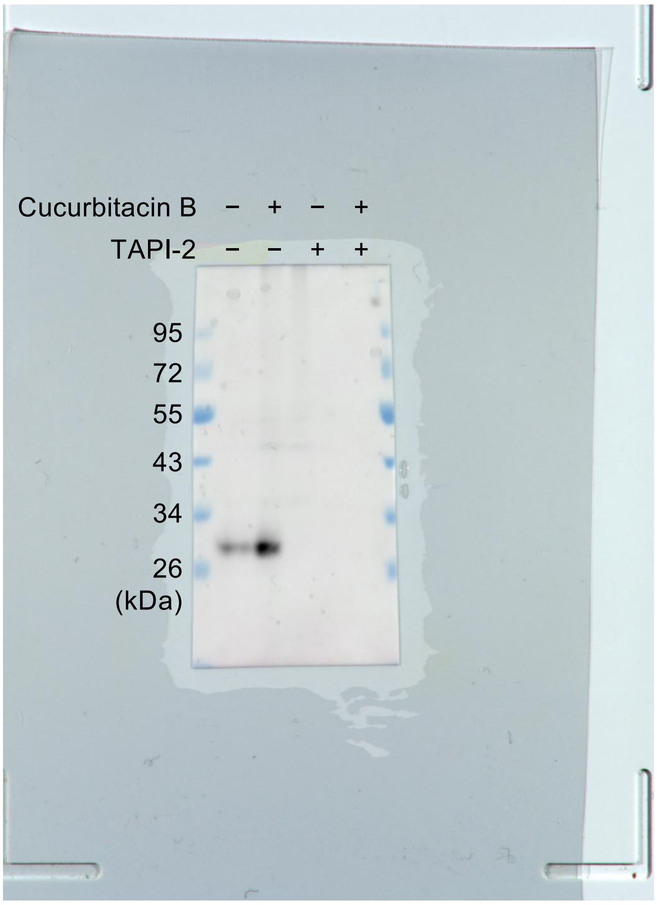


WB: TNF-R1

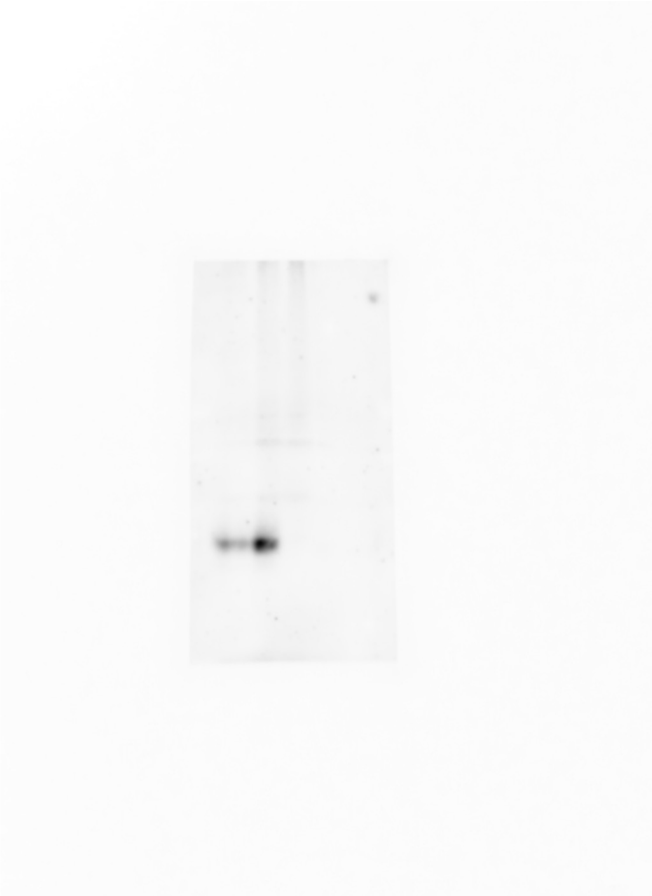

# Figure S18: Original blots (1) in Figure 3C

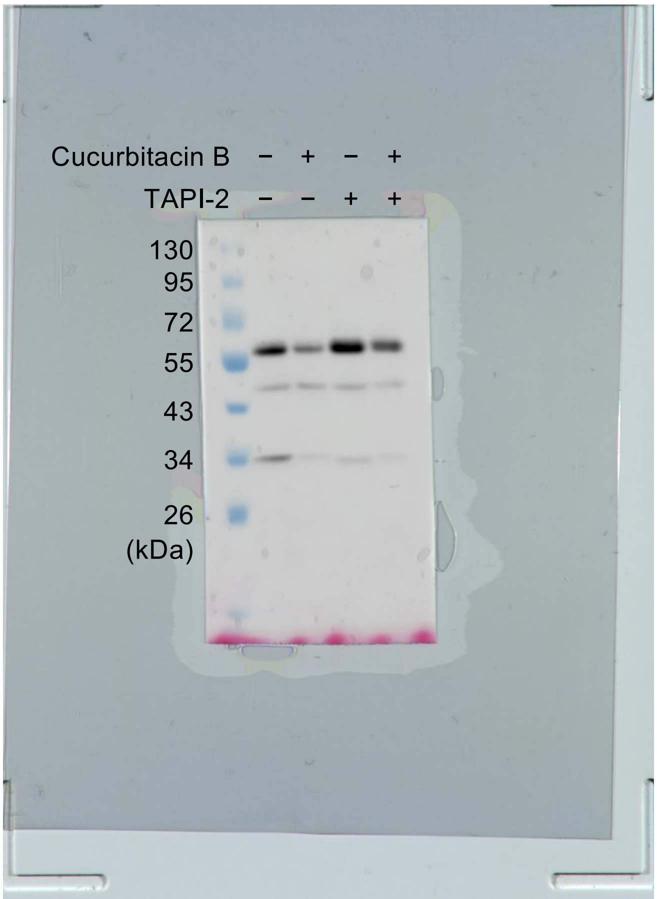


WB: TNF-R1

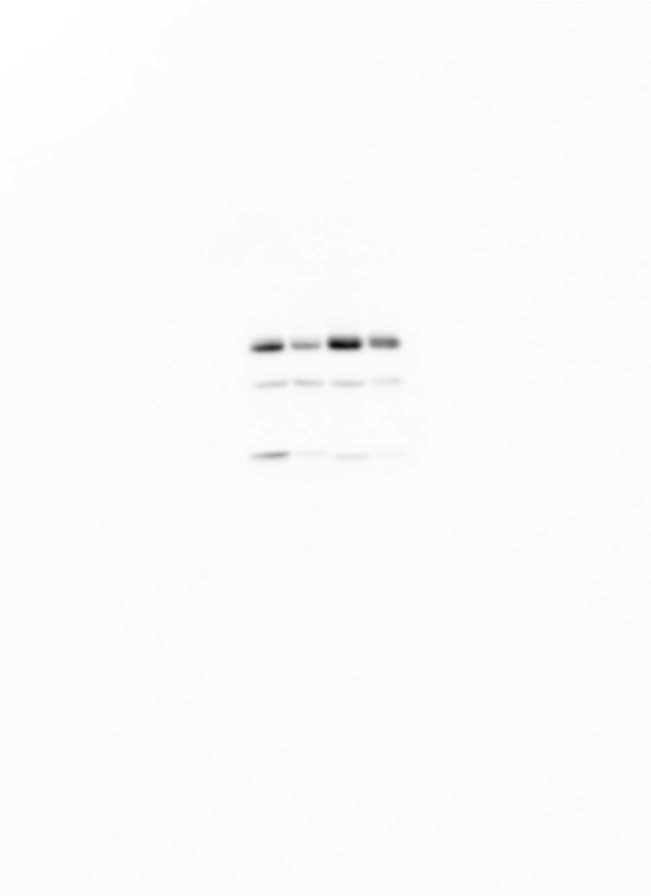

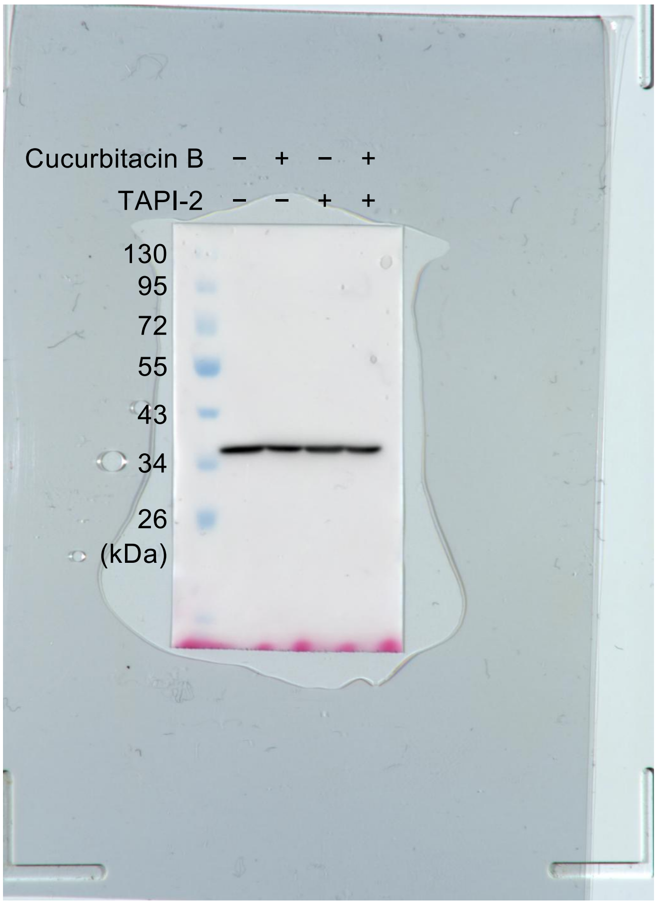


WB: GAPDH (reprobed)

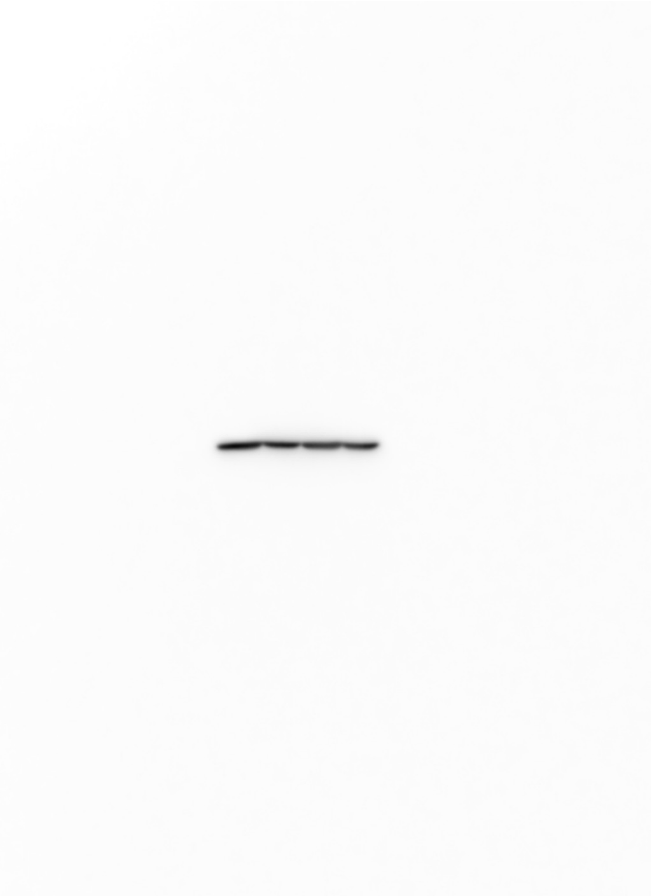

# Figure S19: Original blots (2) in Figure 3C

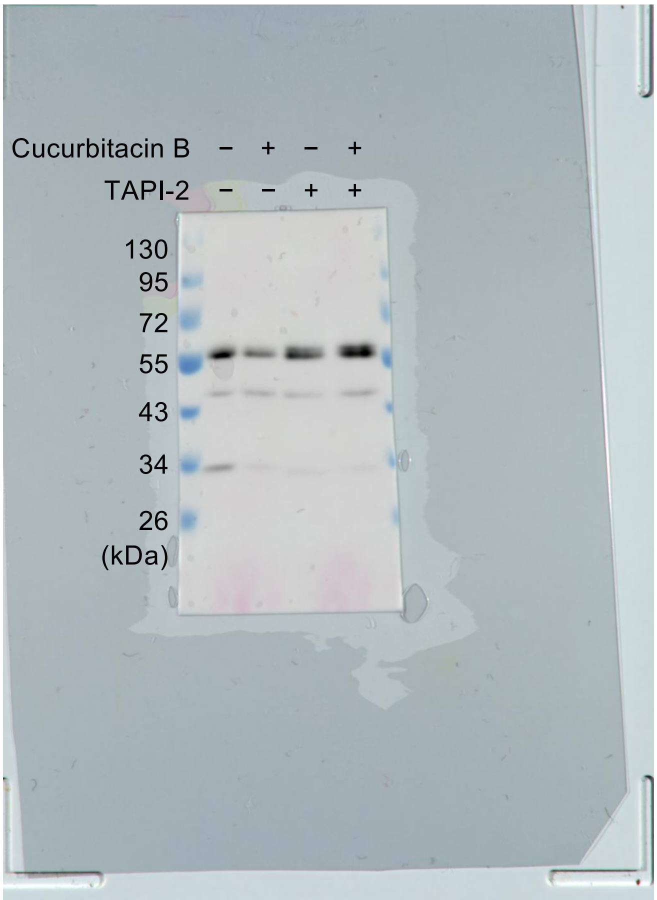


WB: TNF-R1

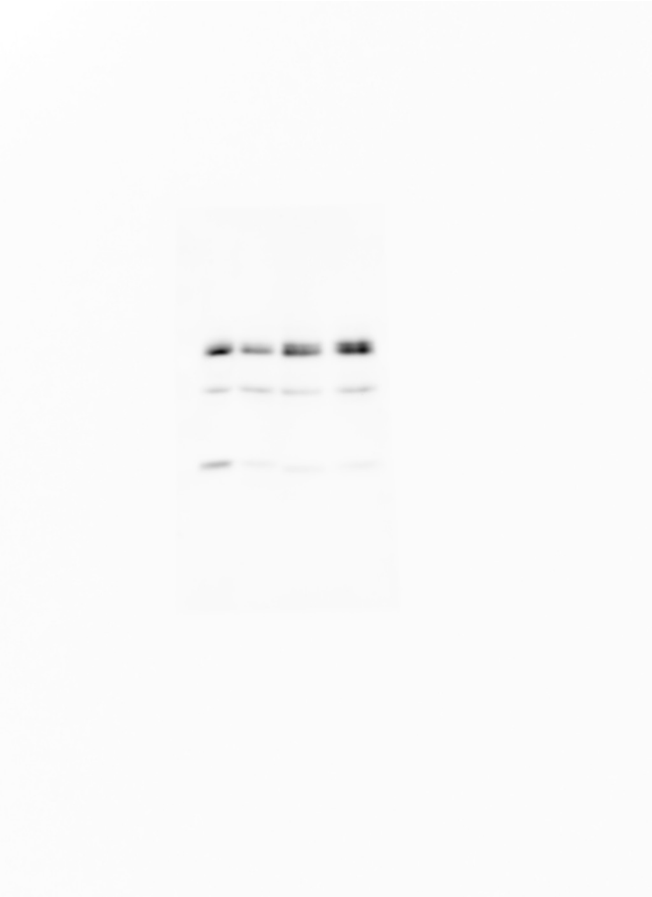

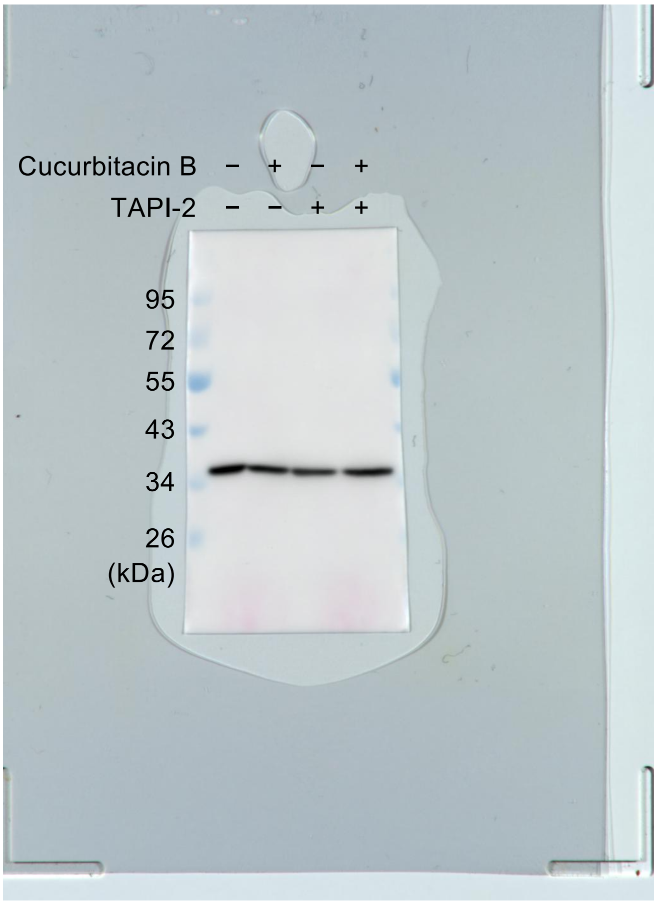


WB: GAPDH (reprobed)

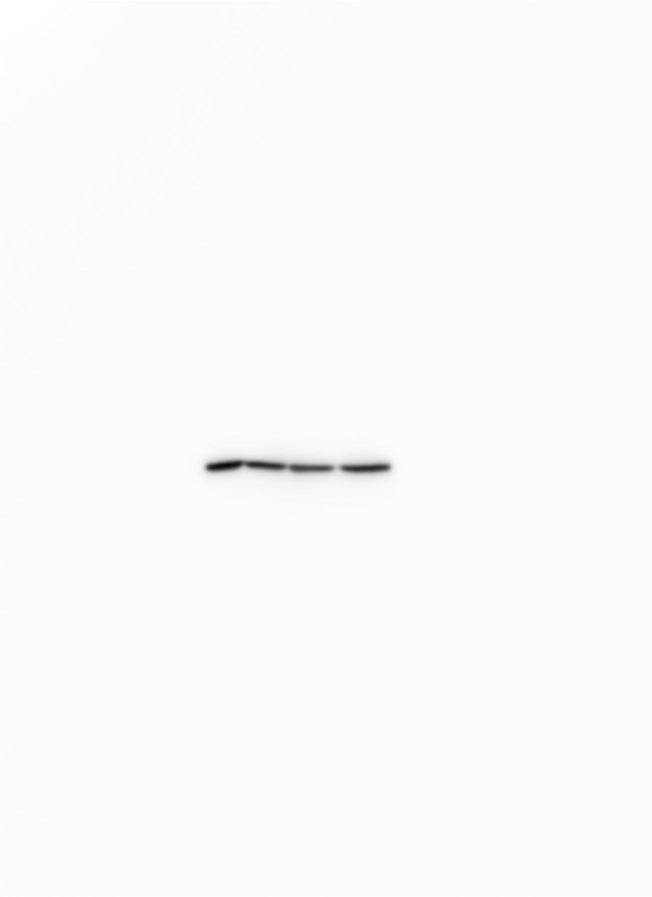

## Figure S20: Original blots (3) in Figure 3C

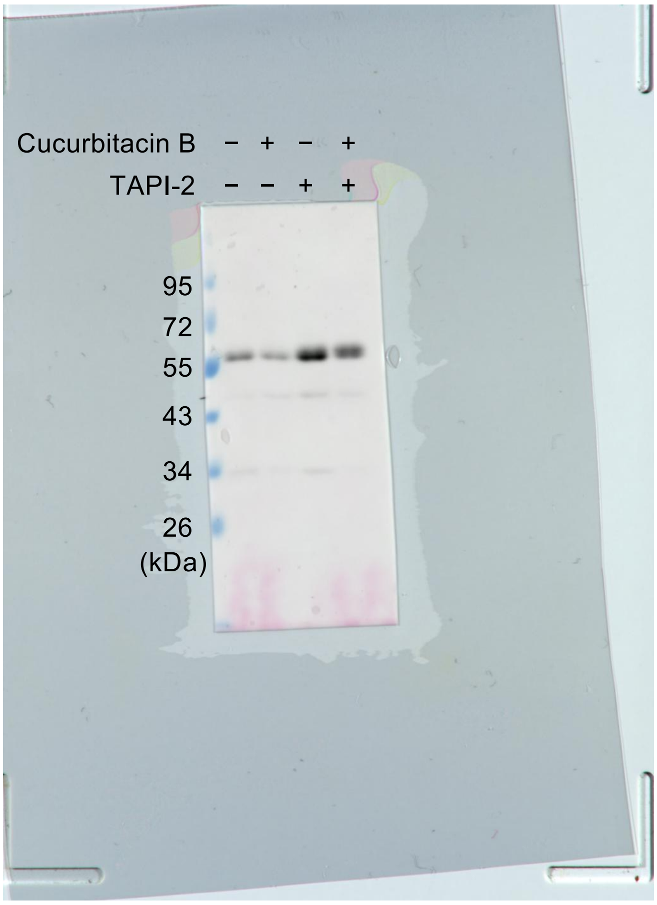


WB: TNF-R1

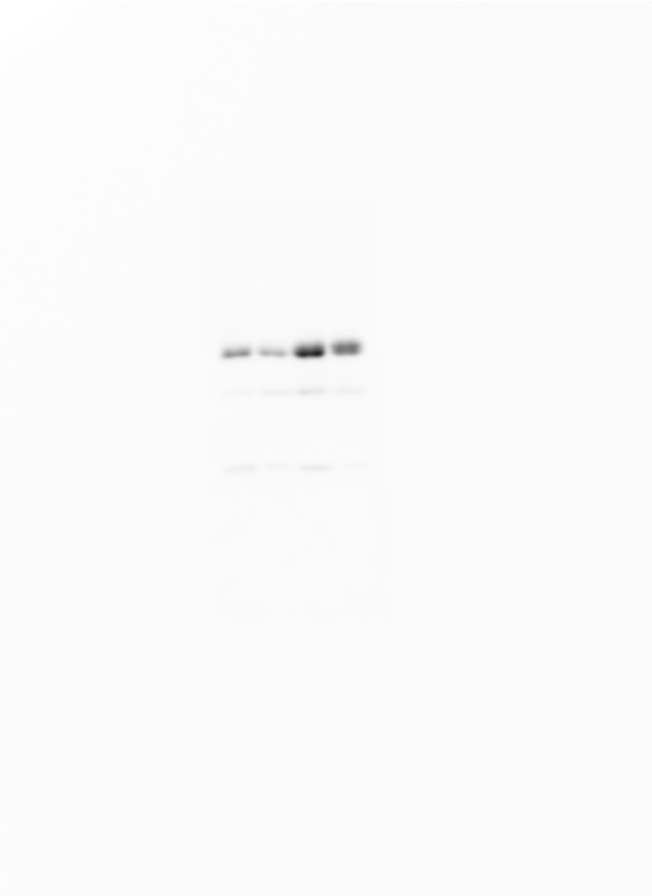

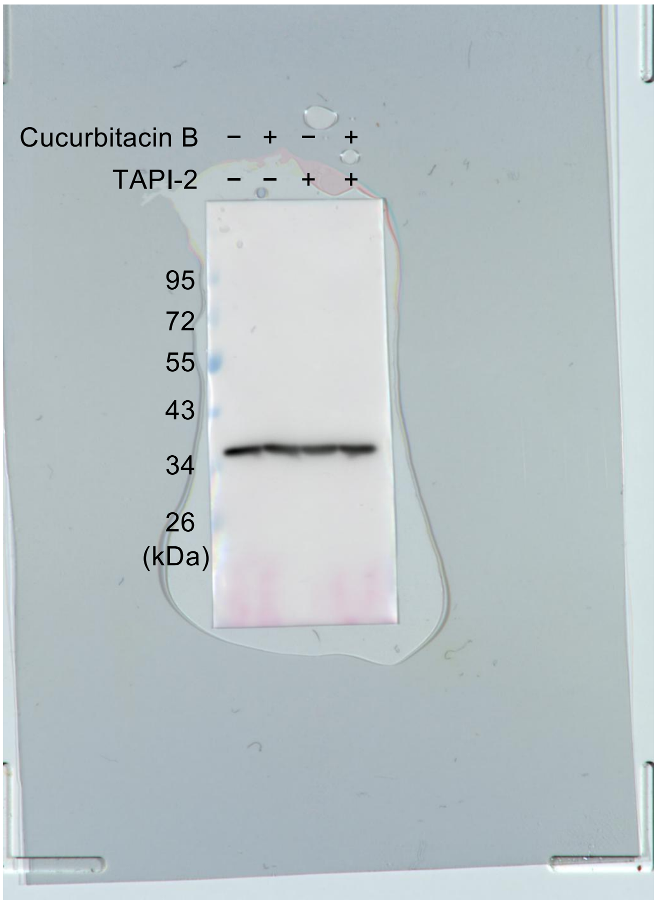


WB: GAPDH (reprobed)

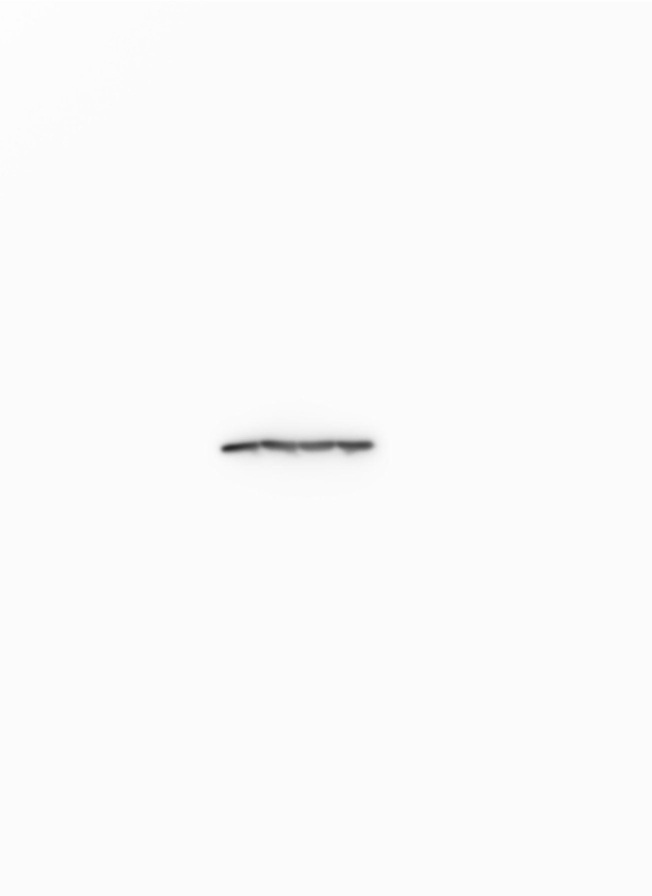

## Figure S21: Original blots in Figure 4A (medium)

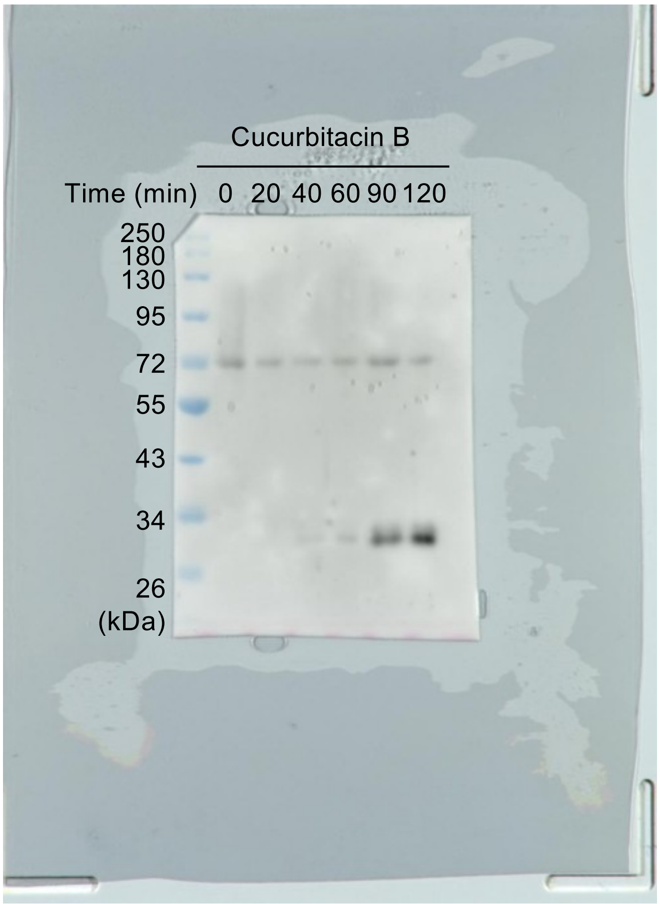


WB: TNF-R1

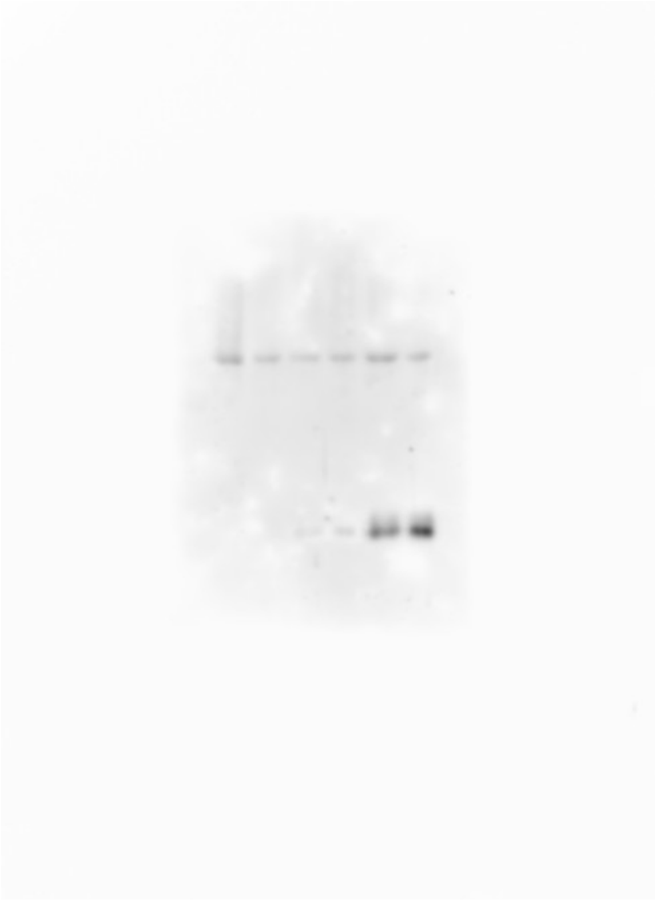

## Figure S22: Original blots in Figure 4A (cell lysate)

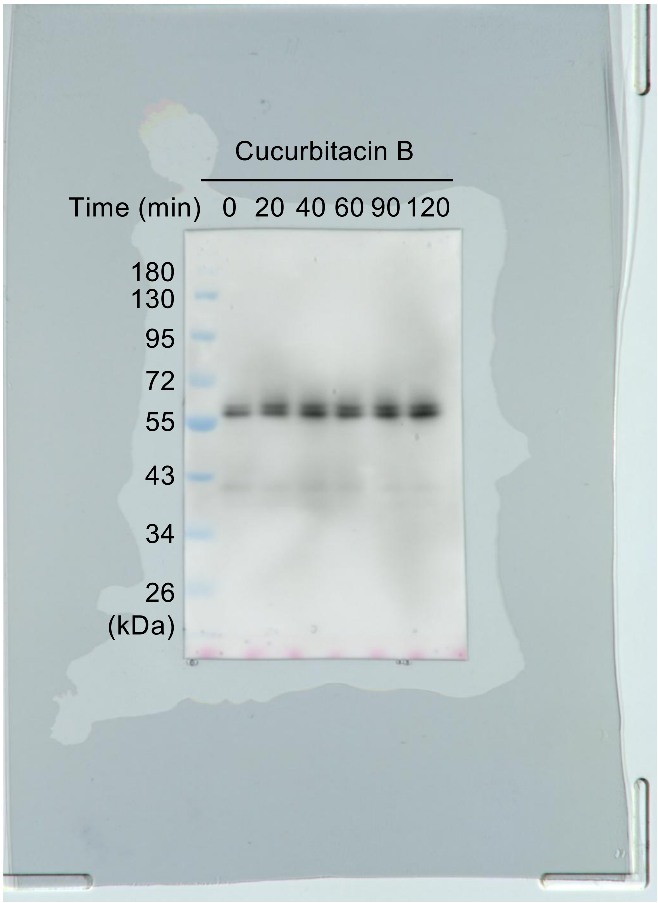


WB: TNF-R1

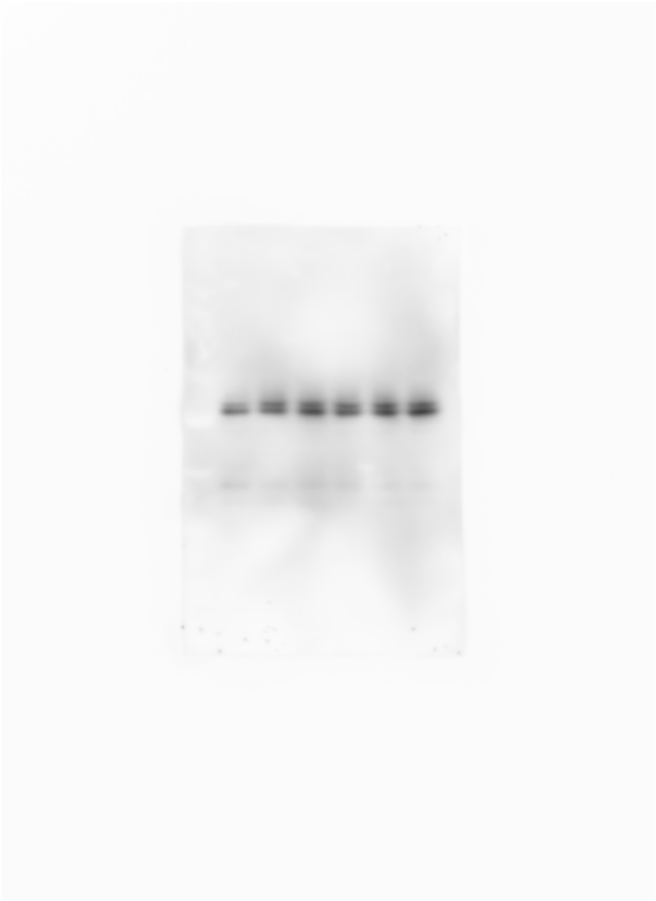

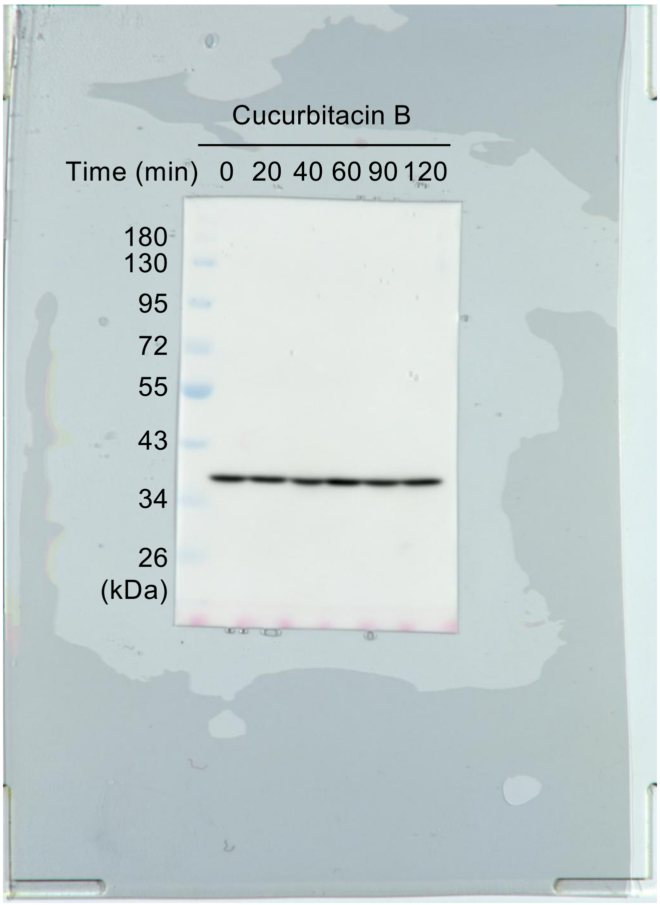


WB: GAPDH (reprobed)

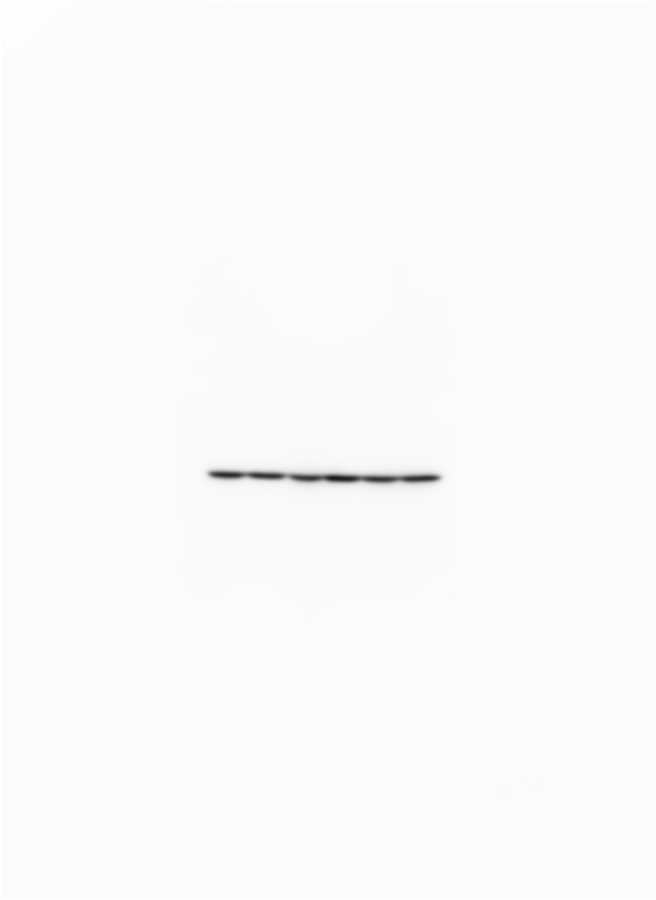

# Figure S23: Original blots (1) in Figure 4B

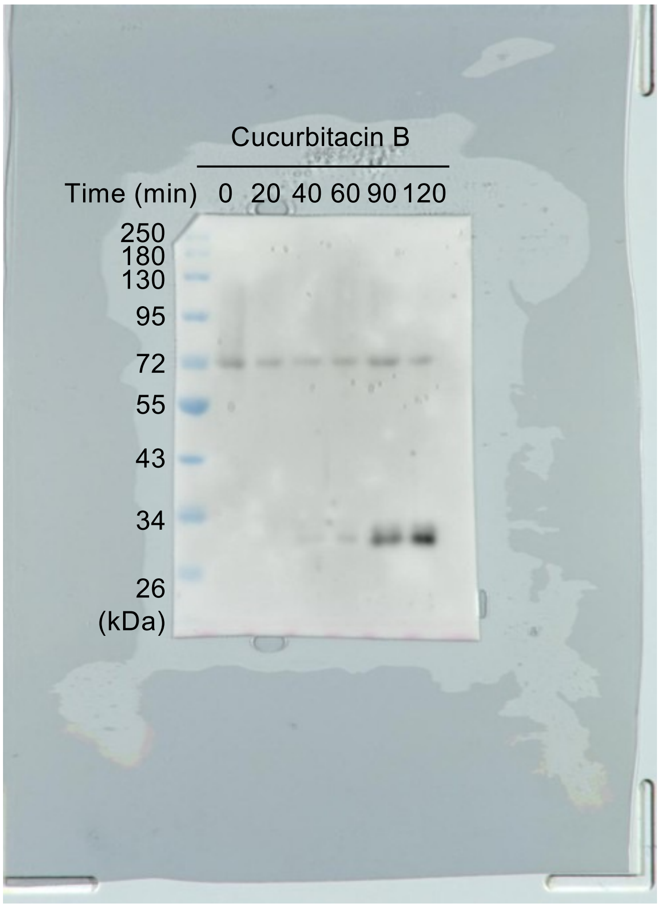


WB: TNF-R1

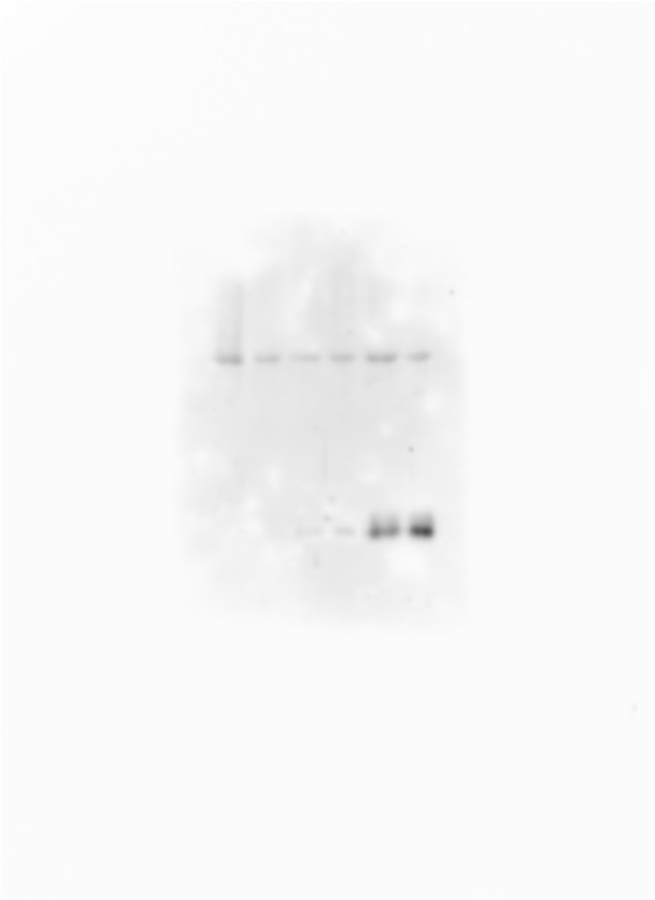

# Figure S24: Original blots (2) in Figure 4B

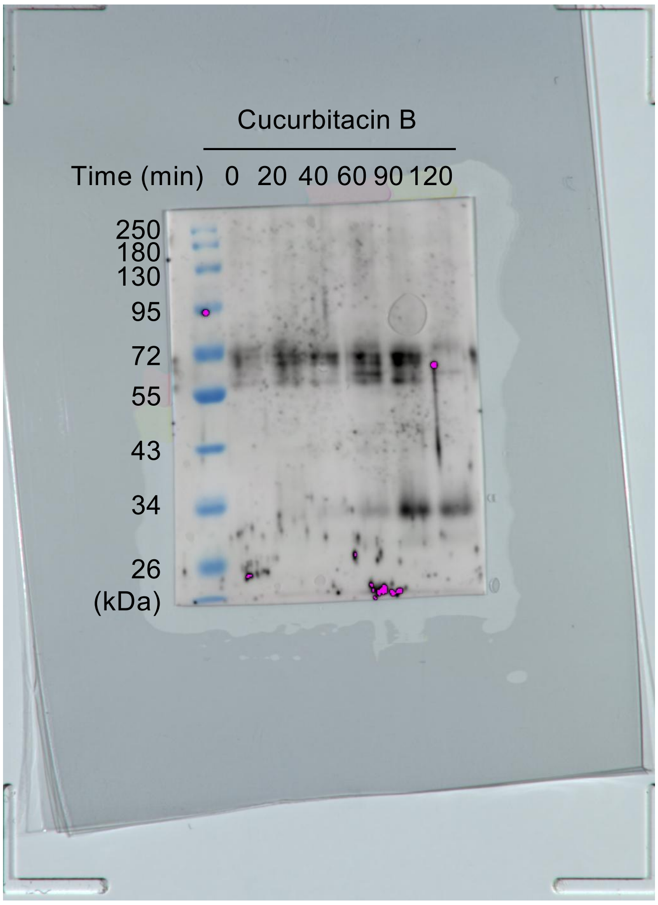


WB: TNF-R1

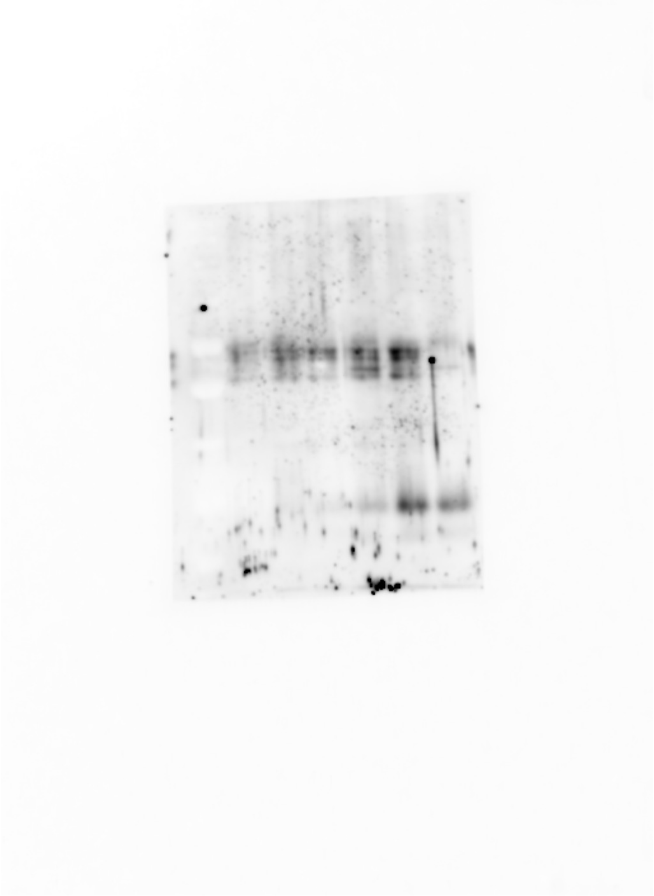

# Figure S25: Original blots (3) in Figure 4B

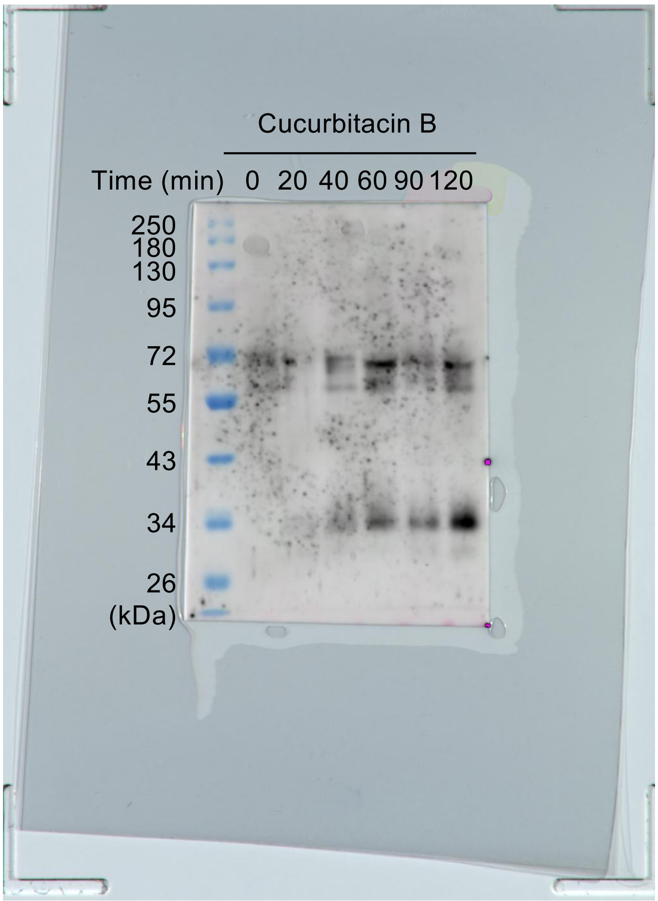


WB: TNF-R1

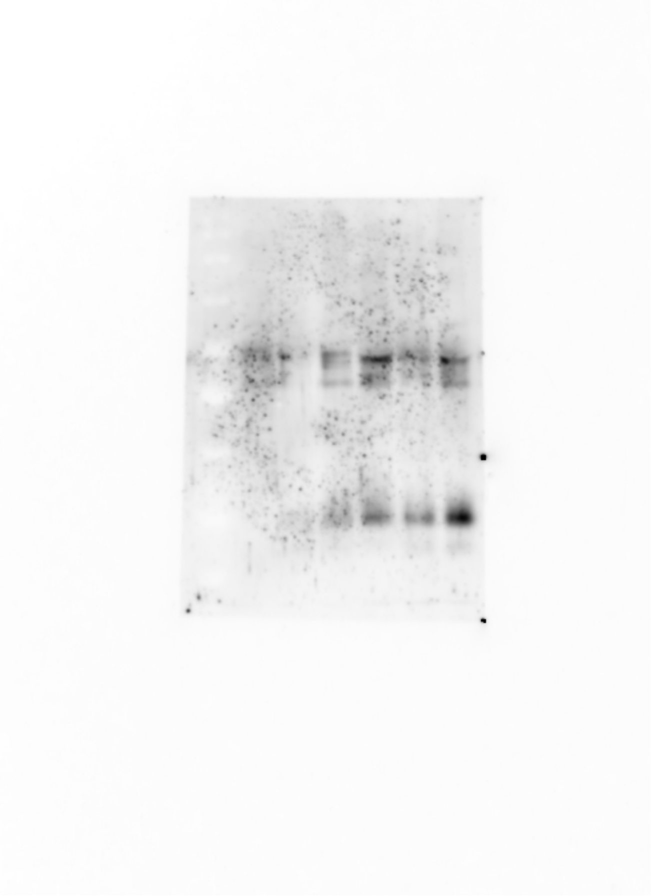

# Figure S26: Original blots (1) in Figure 4C

WB: TNF-R1

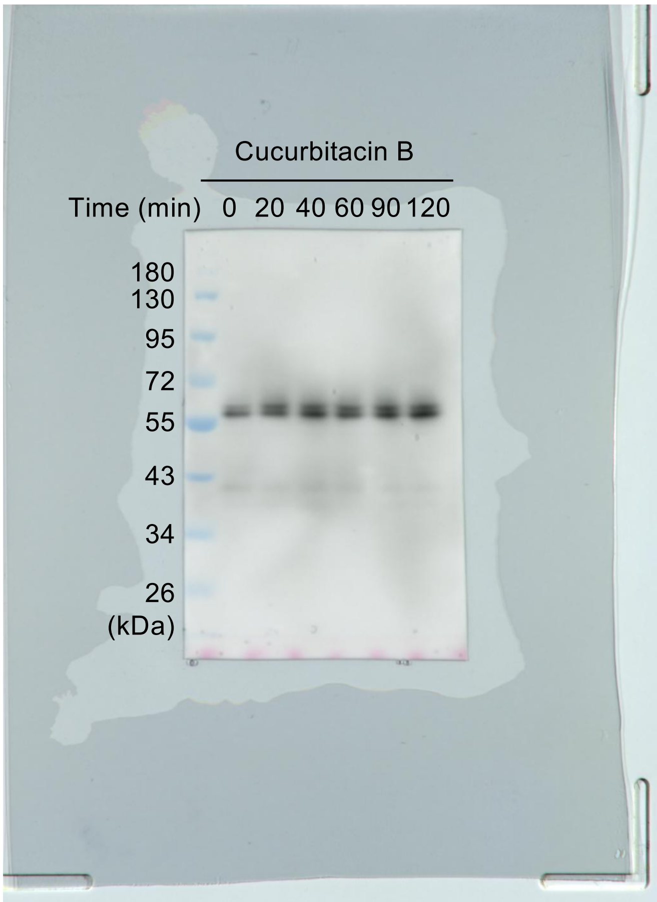


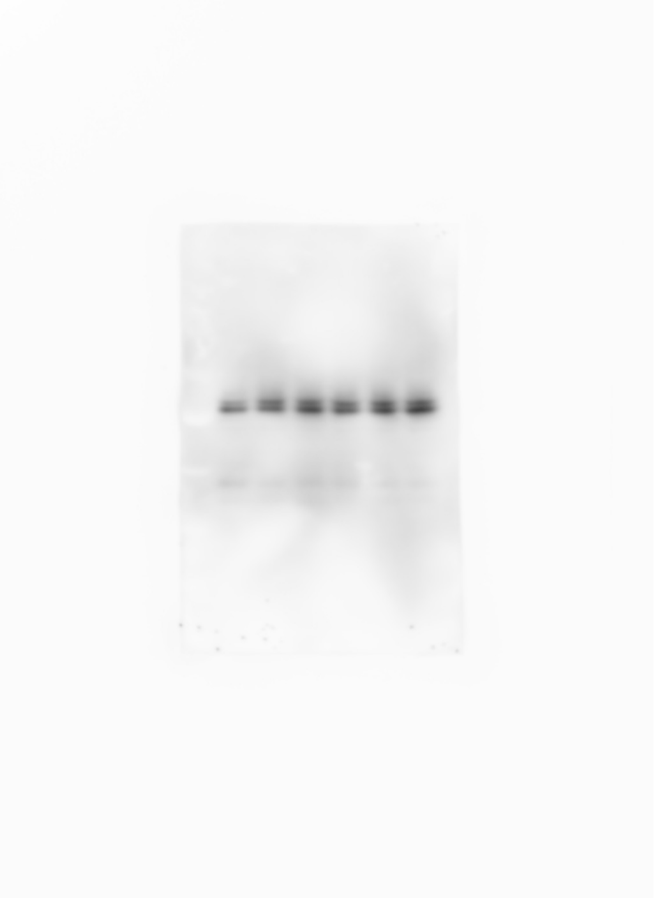

WB: GAPDH (reprobed)

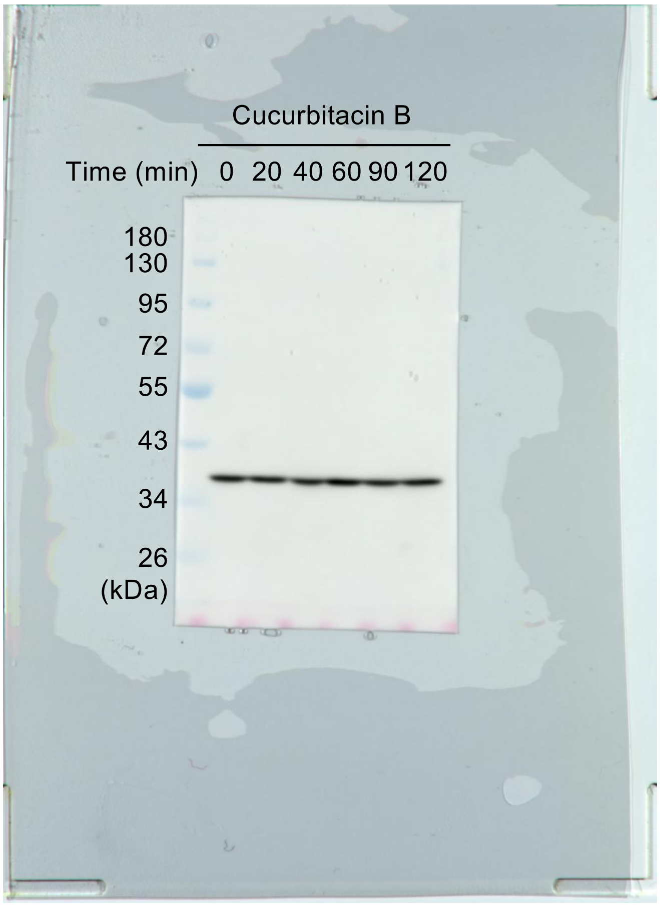


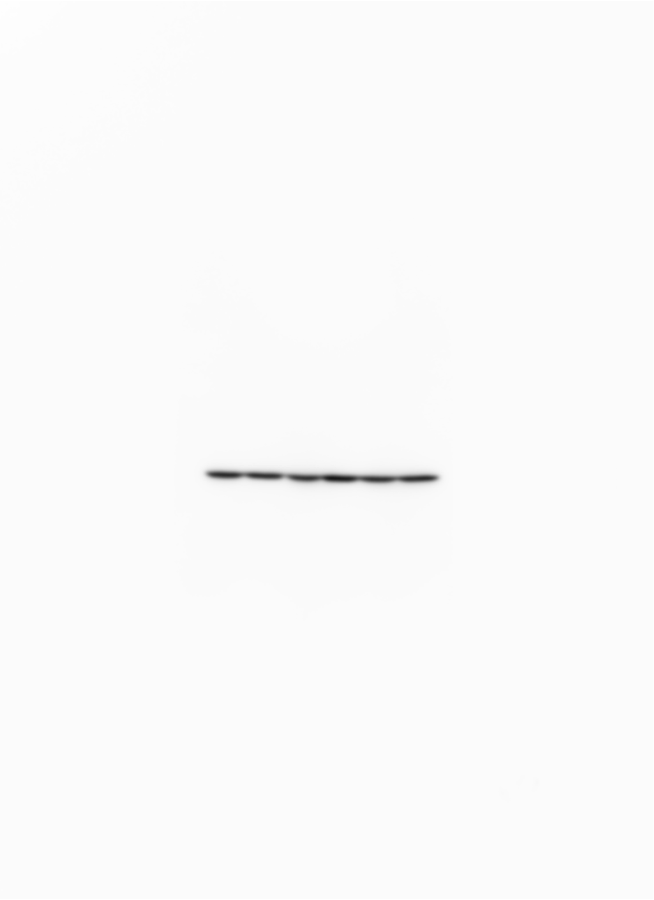

# Figure S27: Original blots (2) in Figure 4C

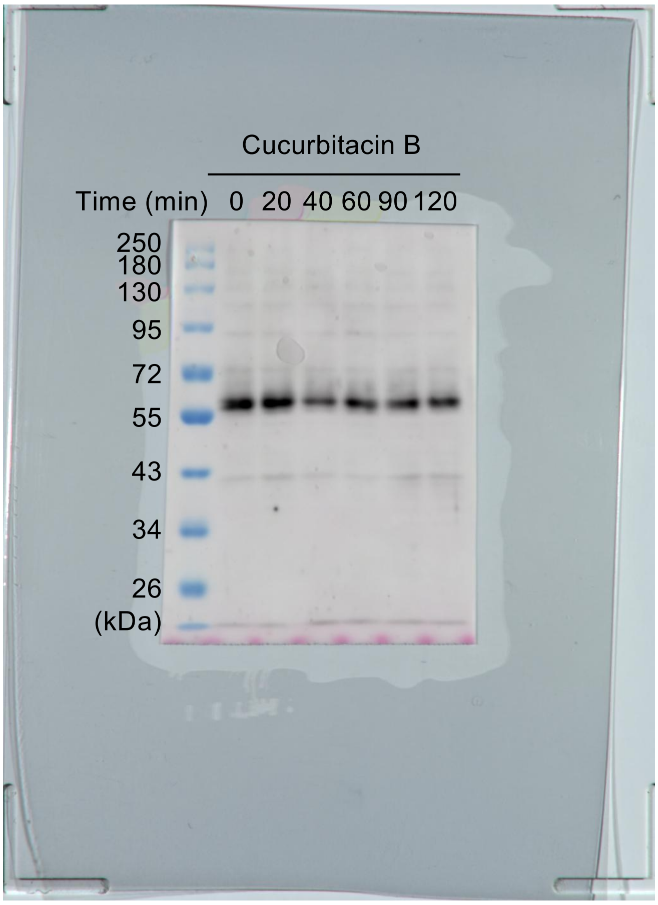


WB: TNF-R1

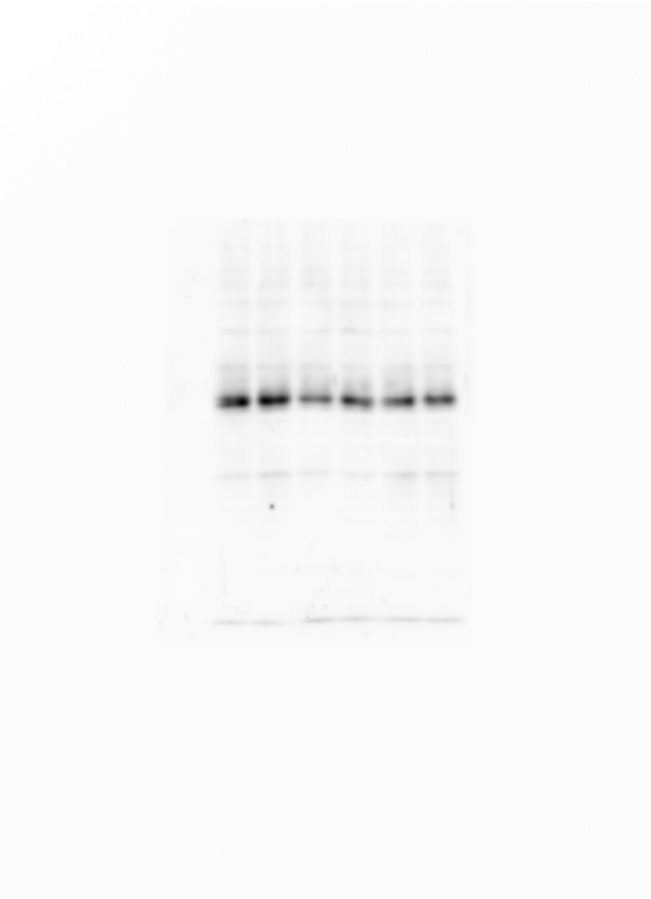

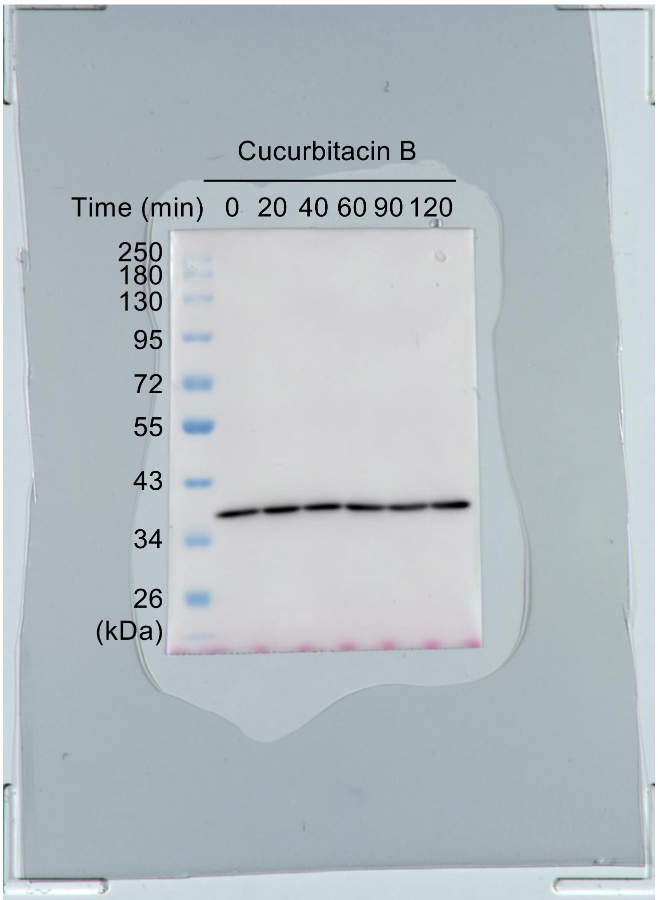


WB: GAPDH (reprobed)

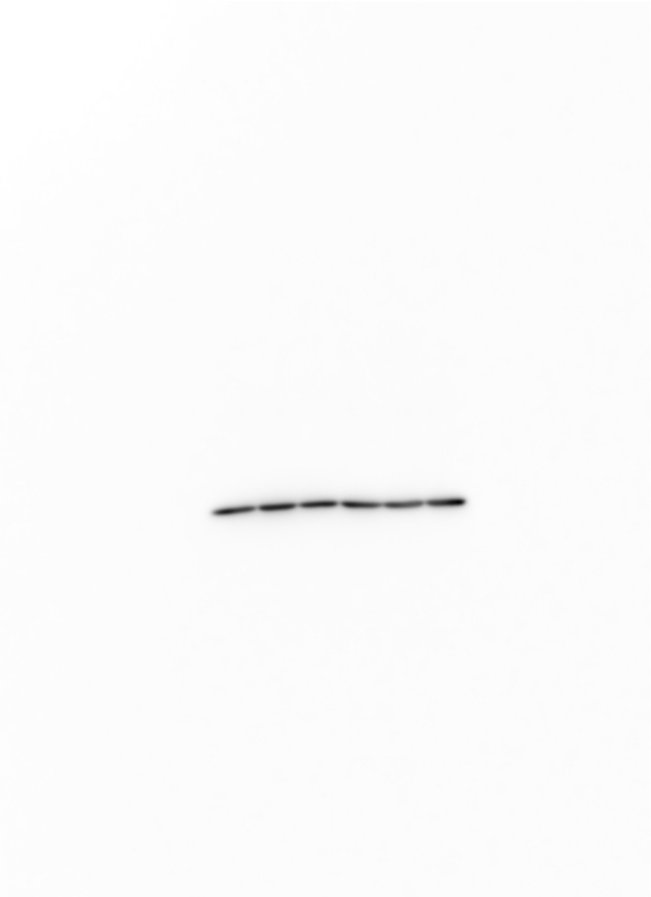

# Figure S28: Original blots (3) in Figure 4C

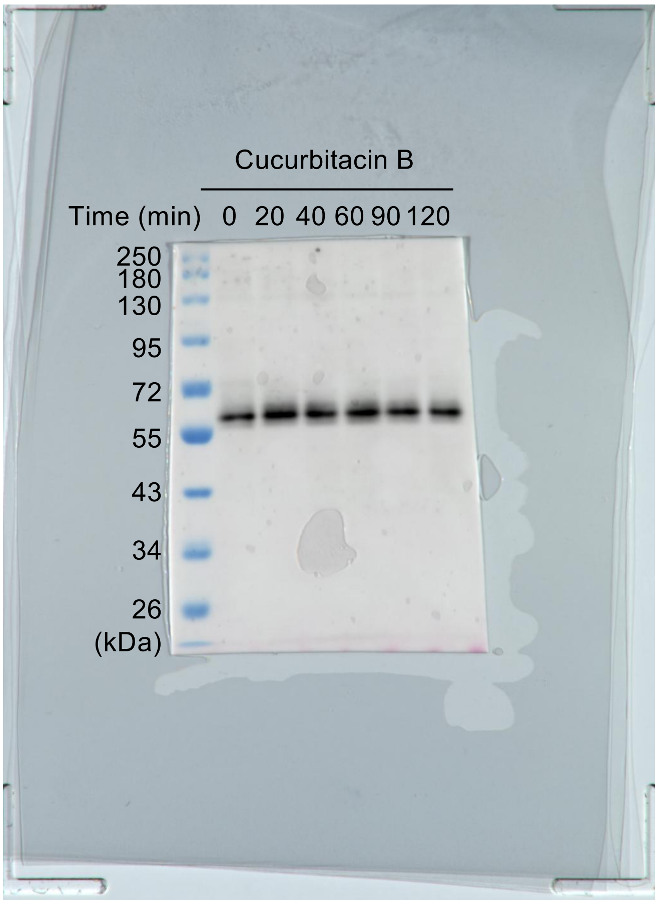


WB: TNF-R1

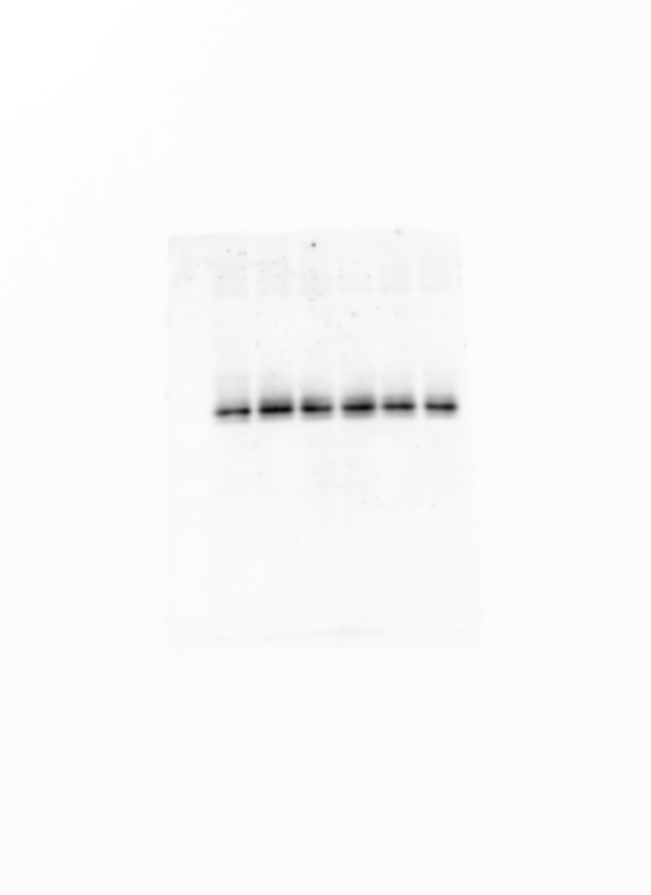

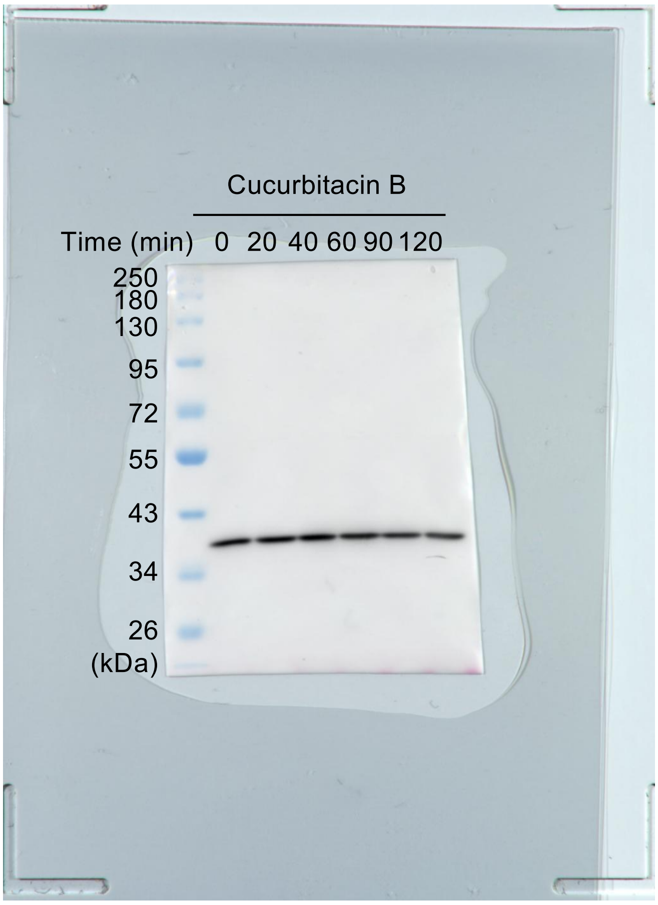


WB: GAPDH (reprobed)

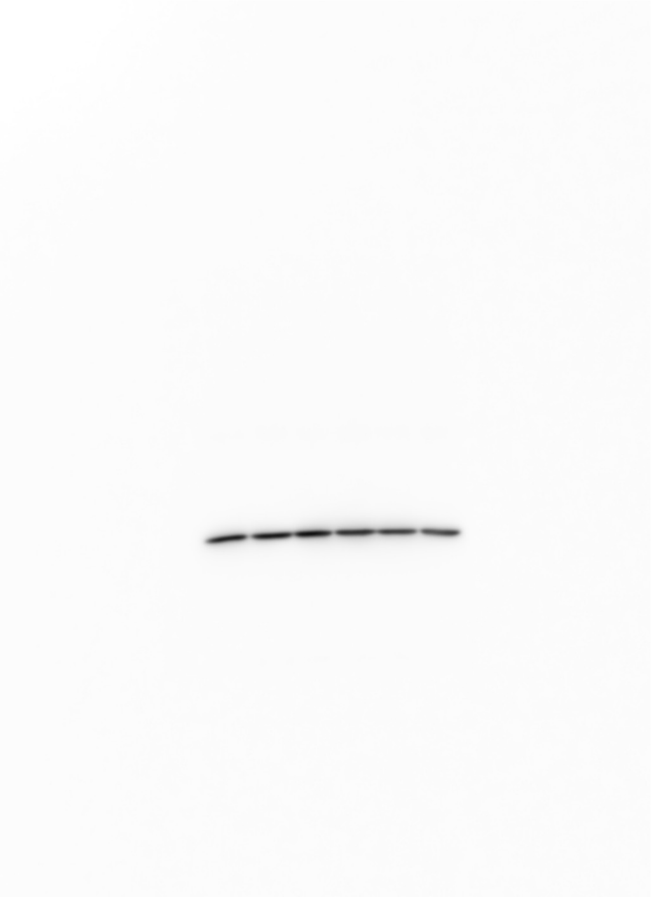

# Figure S29: Original blots in Figure 6A (medium)

WB: TNF-R1

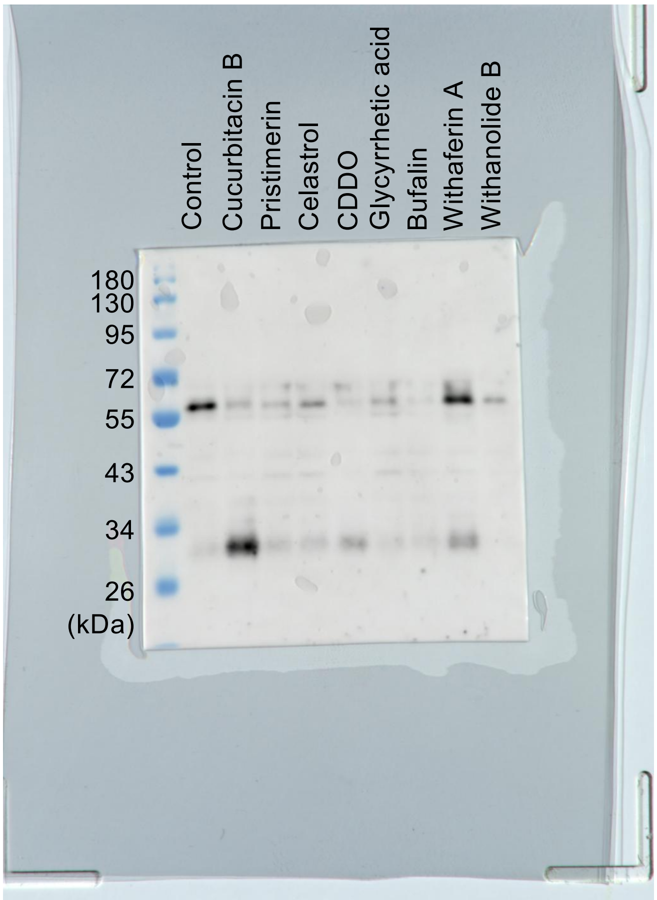


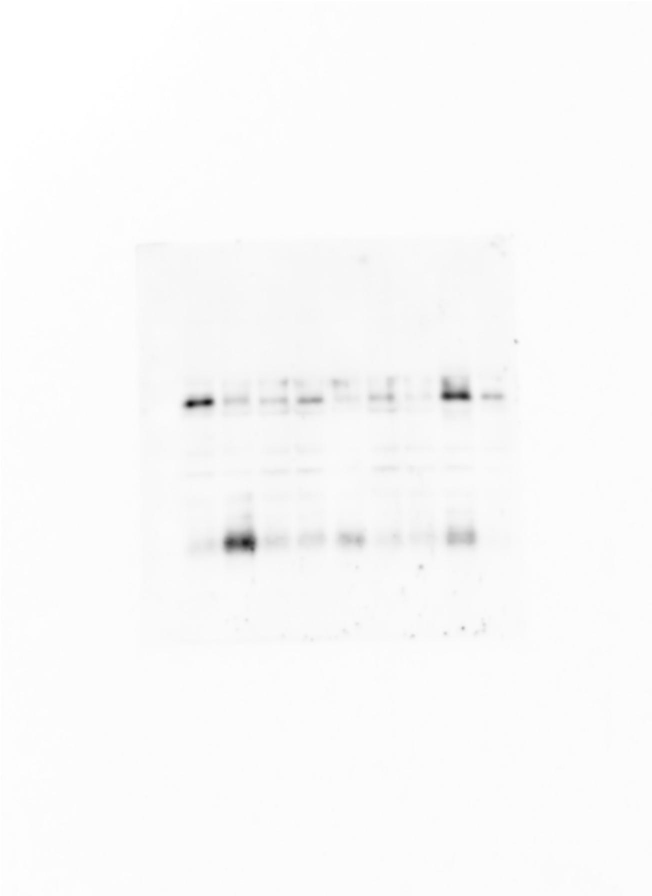

# Figure S30: Original blots in Figure 6A (cell lysate)

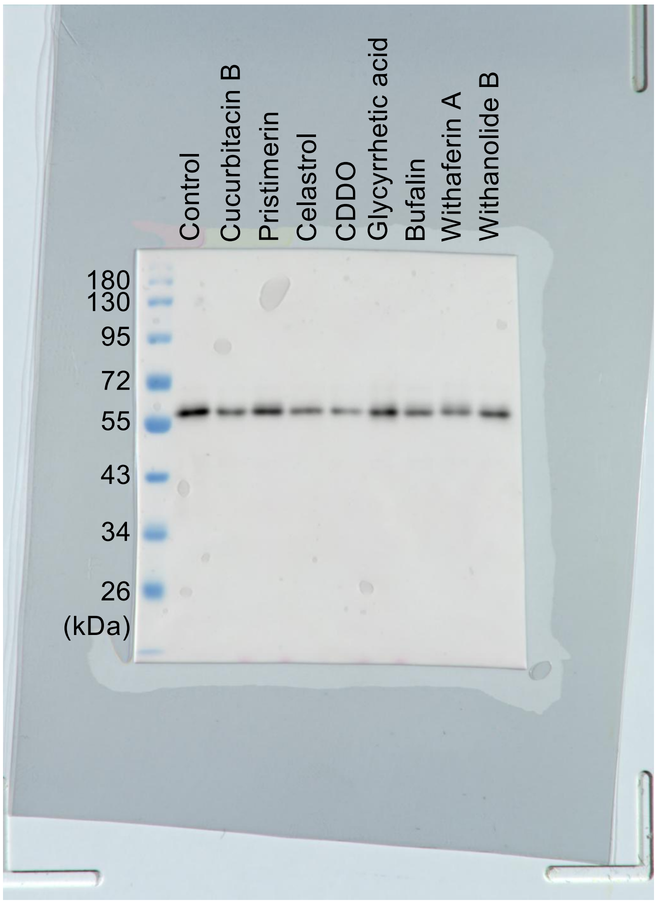


WB: TNF-R1

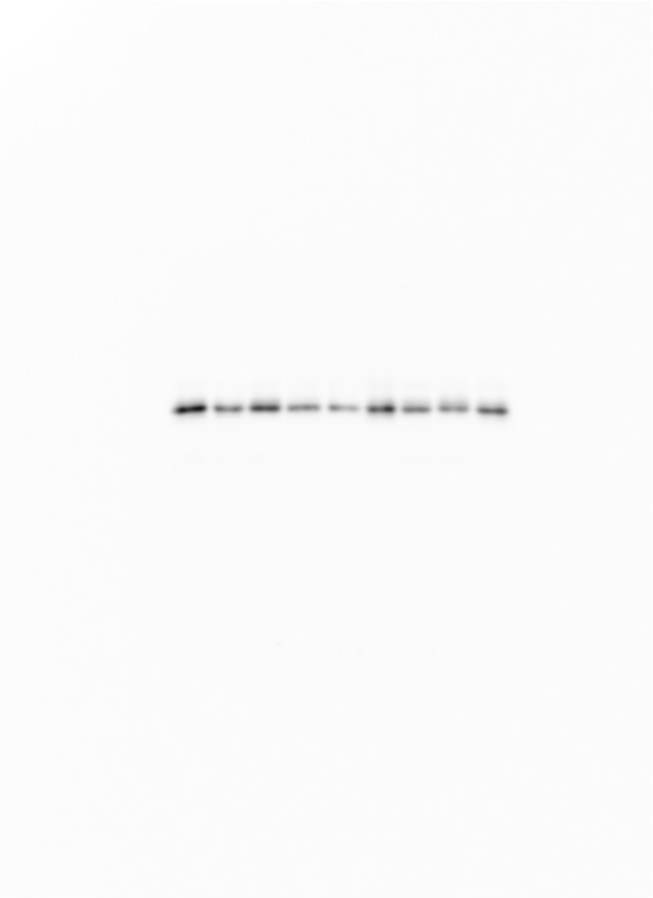

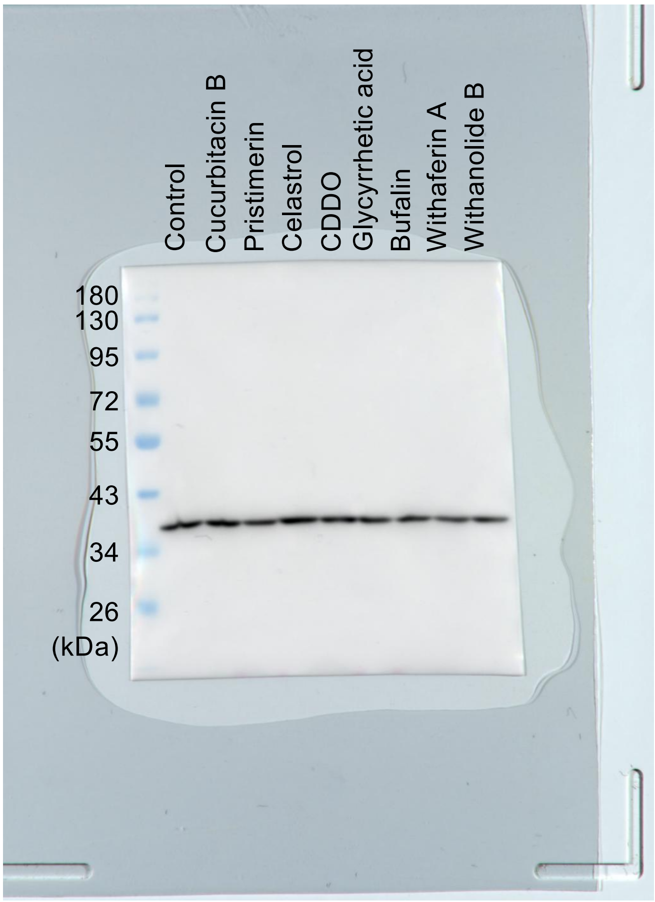


WB: GAPDH (reprobed)

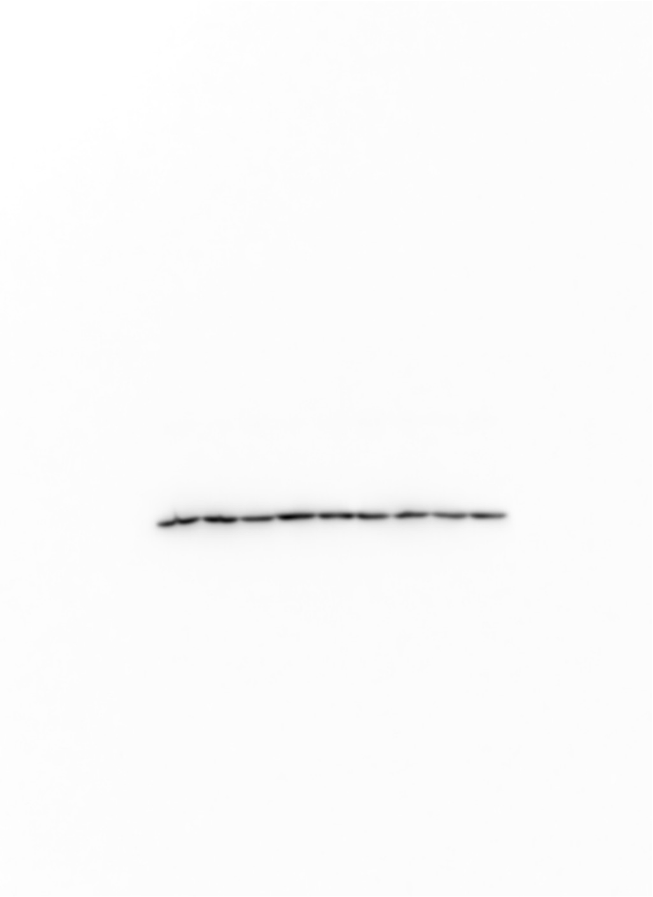

# Figure S31: Original blots (1) in Figure 6B

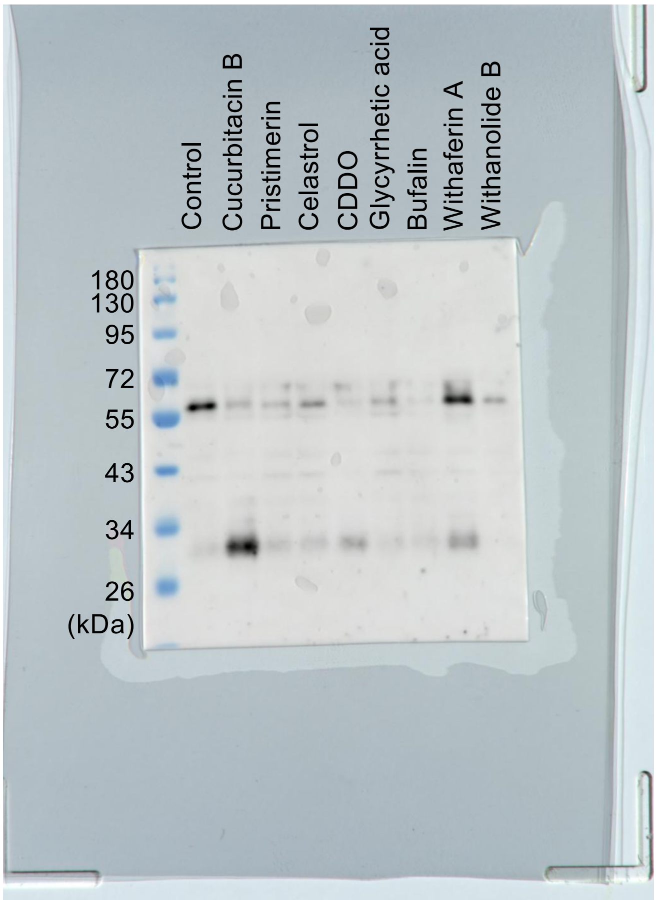


WB: TNF-R1

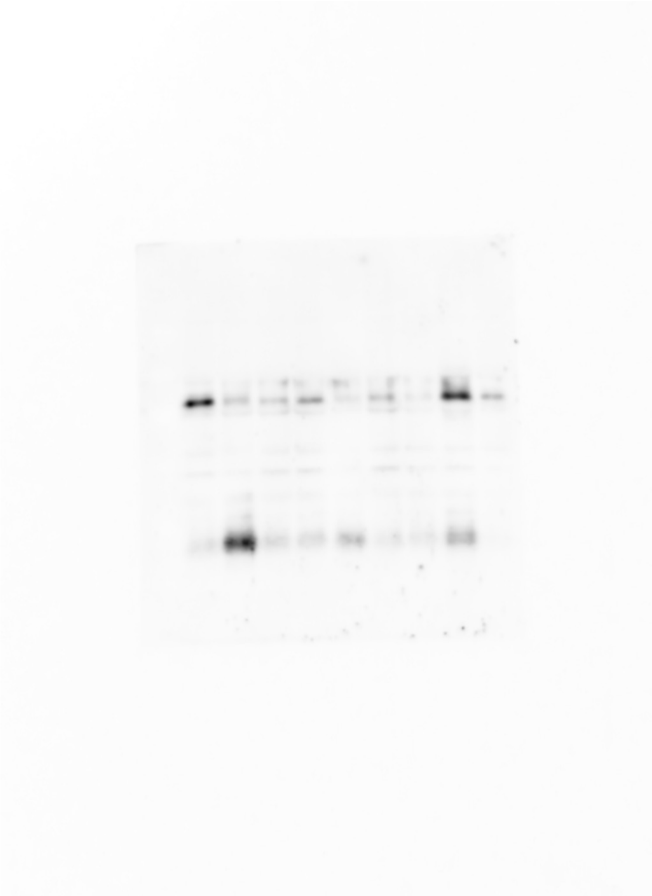

# Figure S32: Original blots (2) in Figure 6B

WB: TNF-R1

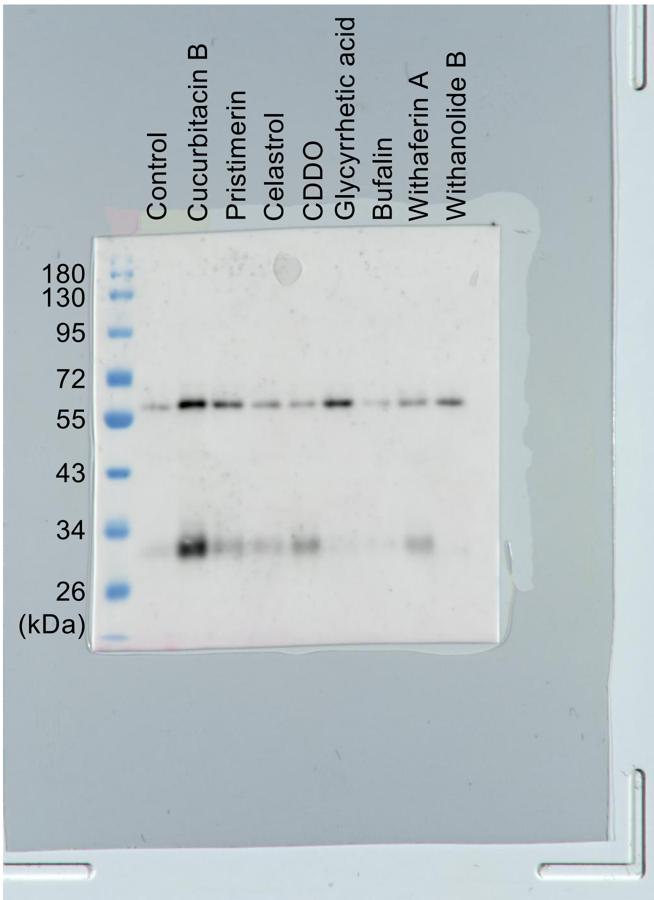


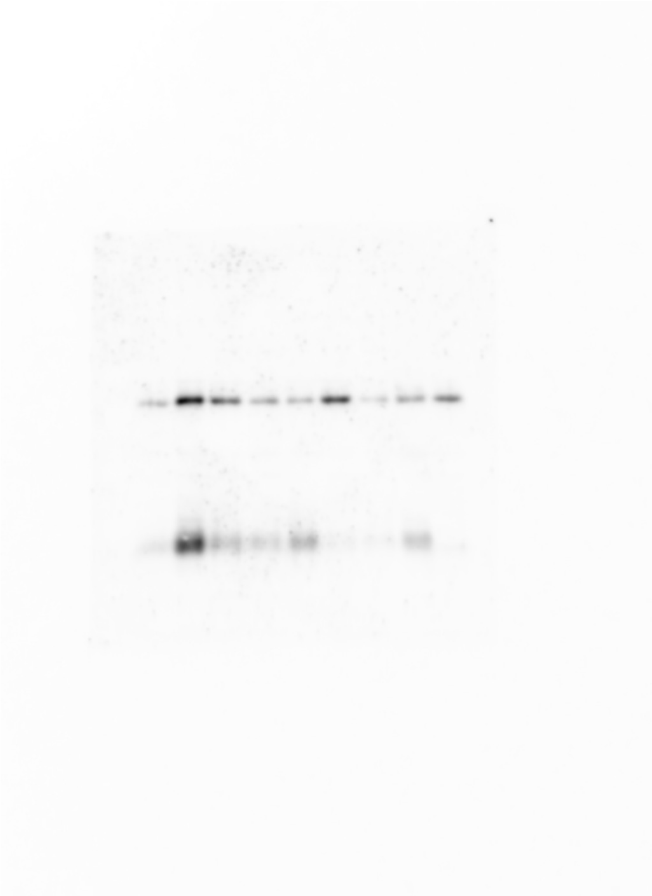

# Figure S33: Original blots (3) in Figure 6B

WB: TNF-R1

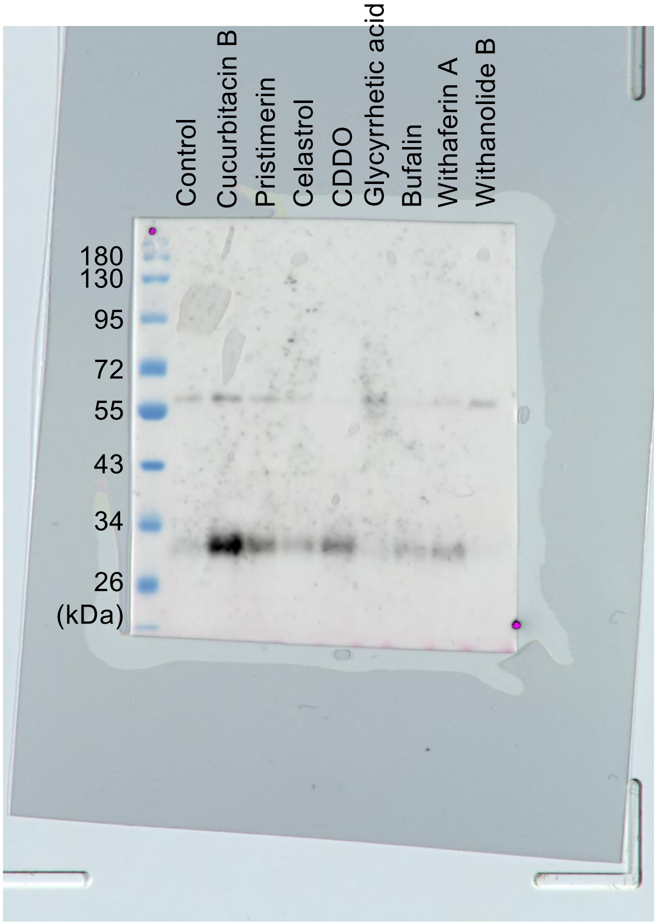


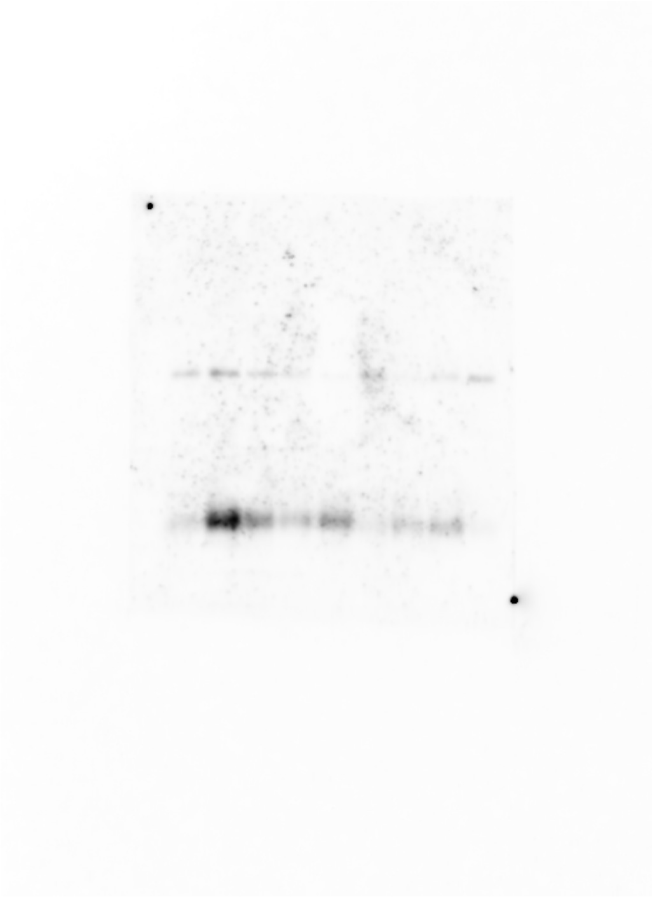

# Figure S34: Original blots (1) in Figure 6C

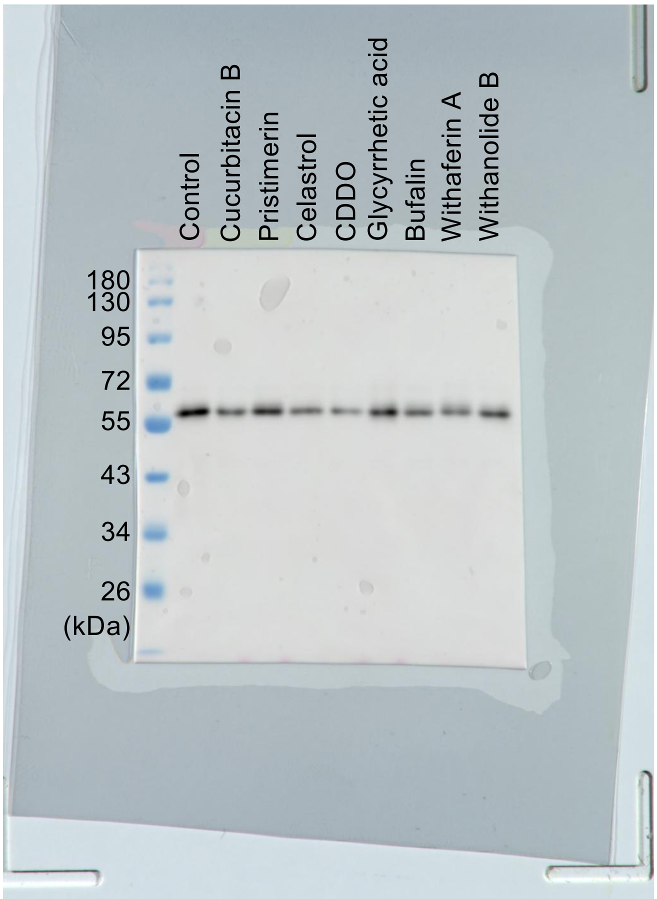


WB: TNF-R1

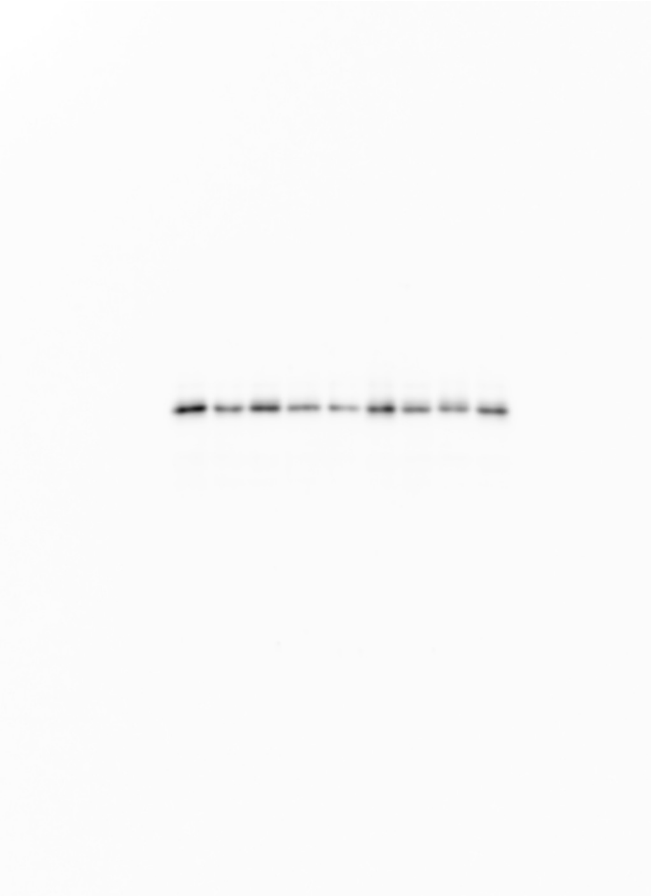

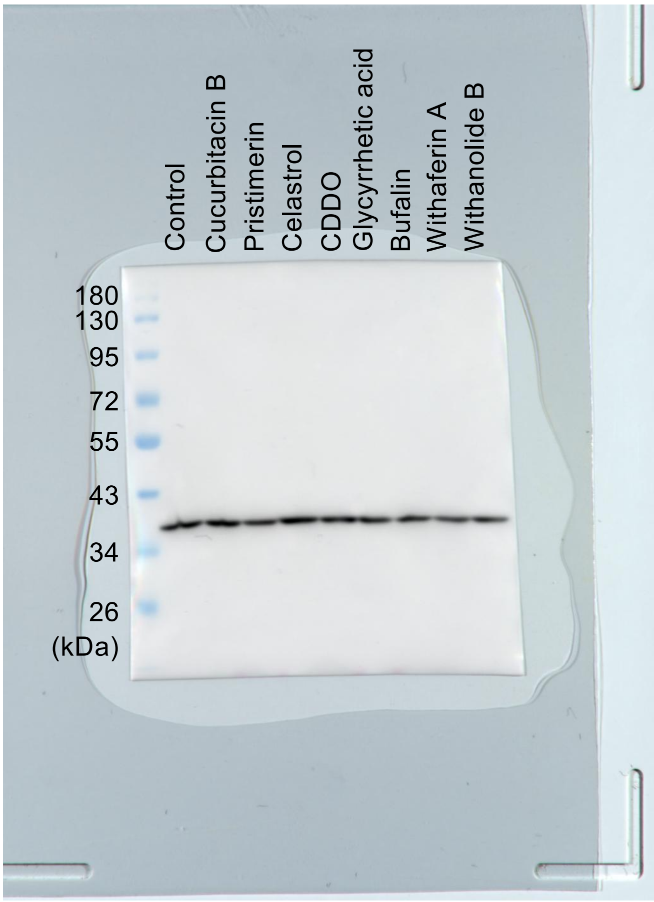


WB: GAPDH (reprobed)

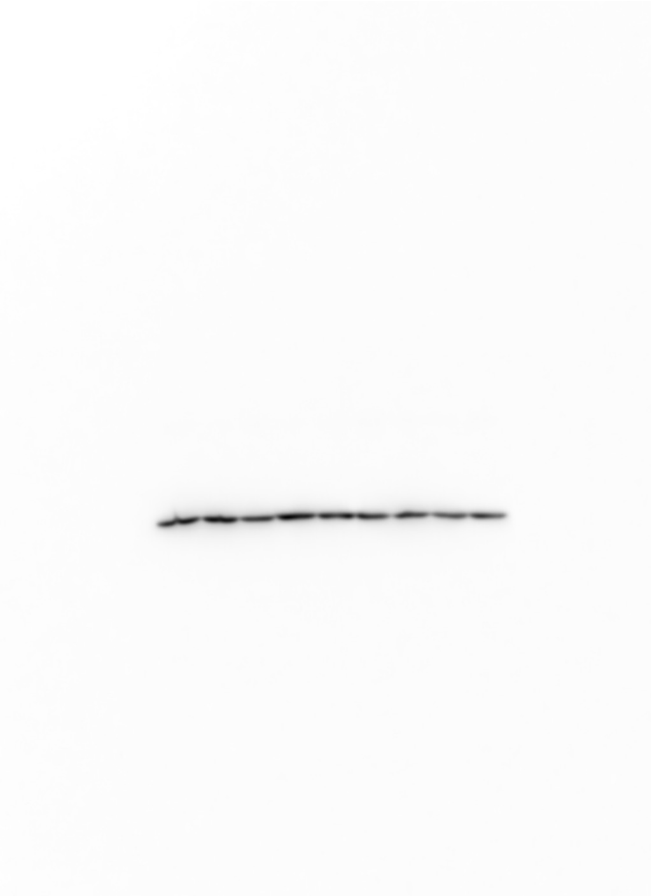

# Figure S35: Original blots (2) in Figure 6C

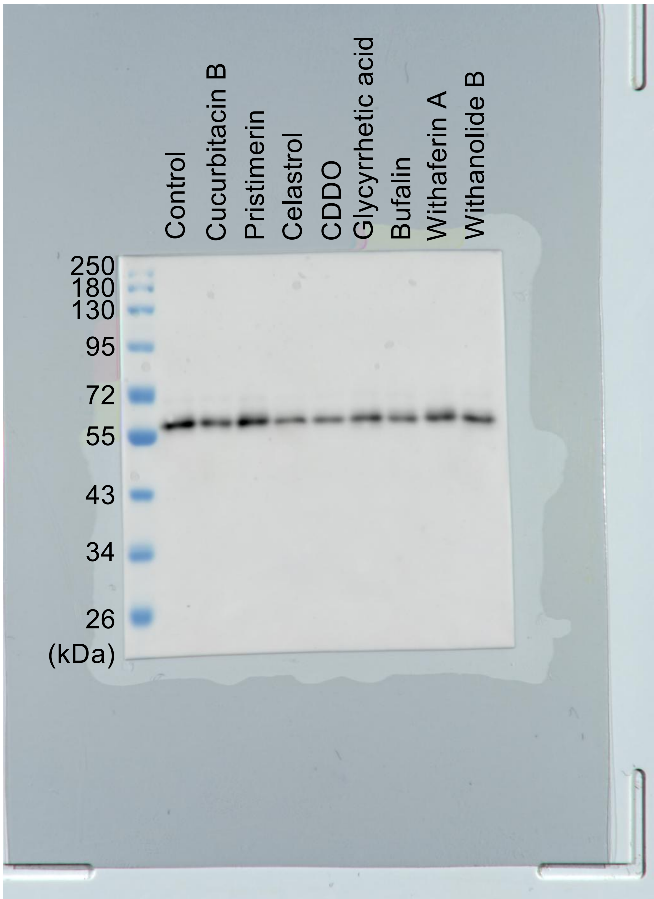


WB: TNF-R1

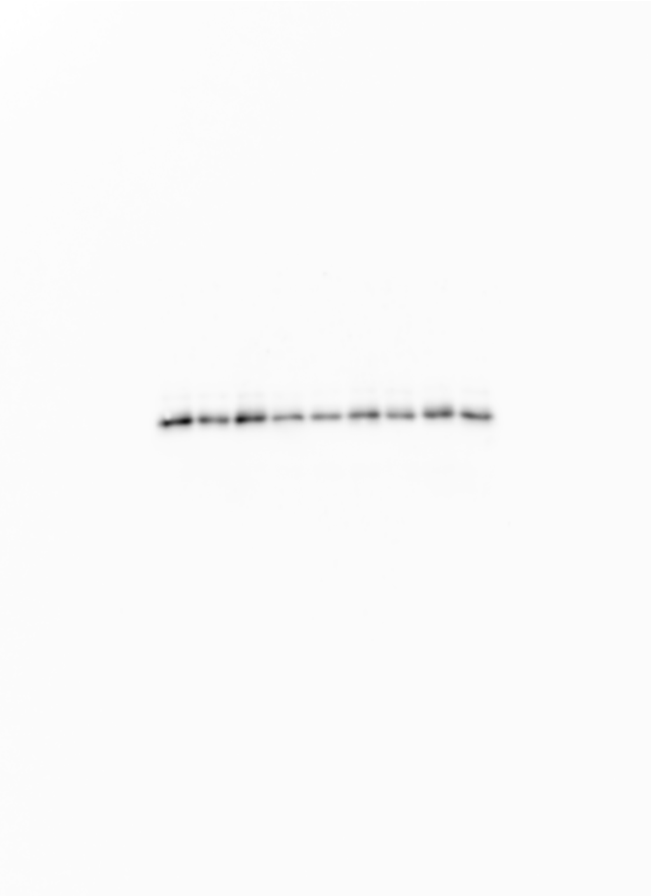

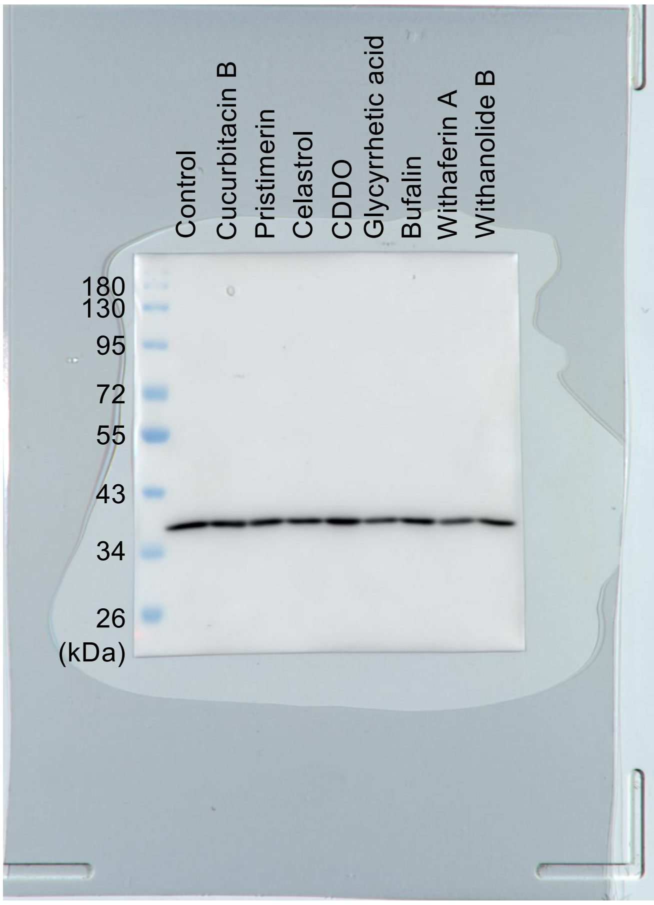


WB: GAPDH (reprobed)

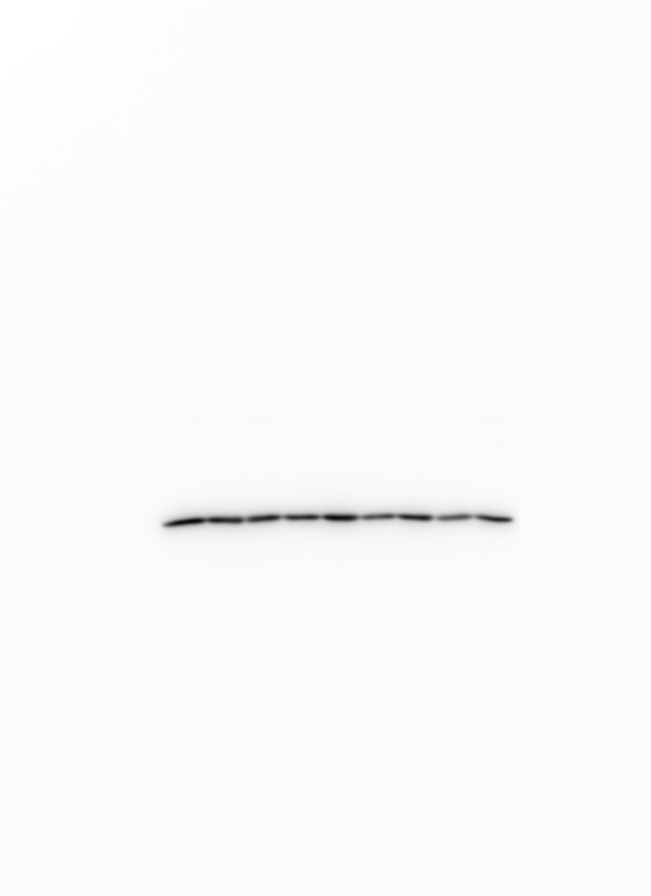

# Figure S36: Original blots (3) in Figure 6C

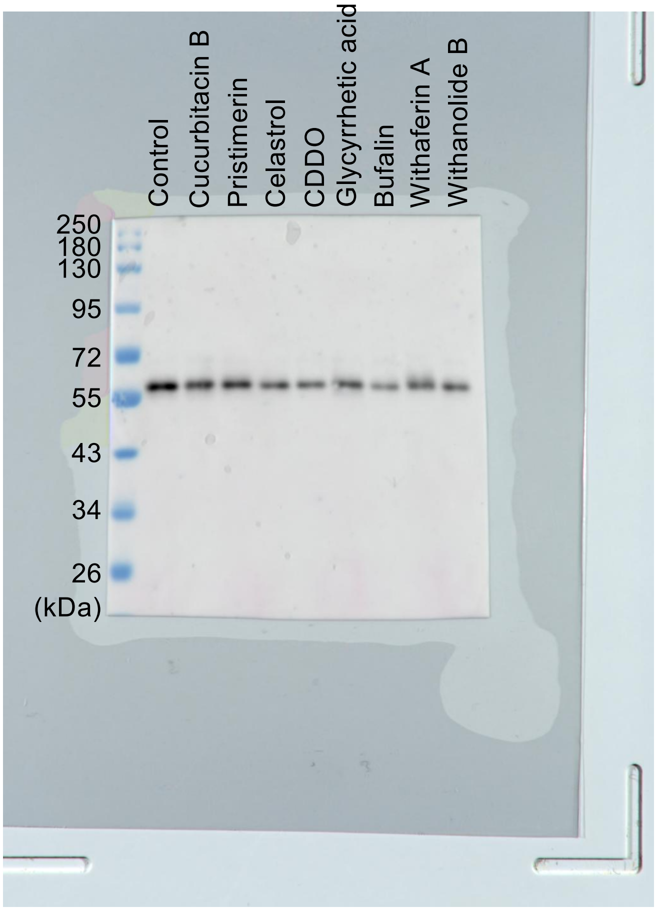


WB: TNF-R1

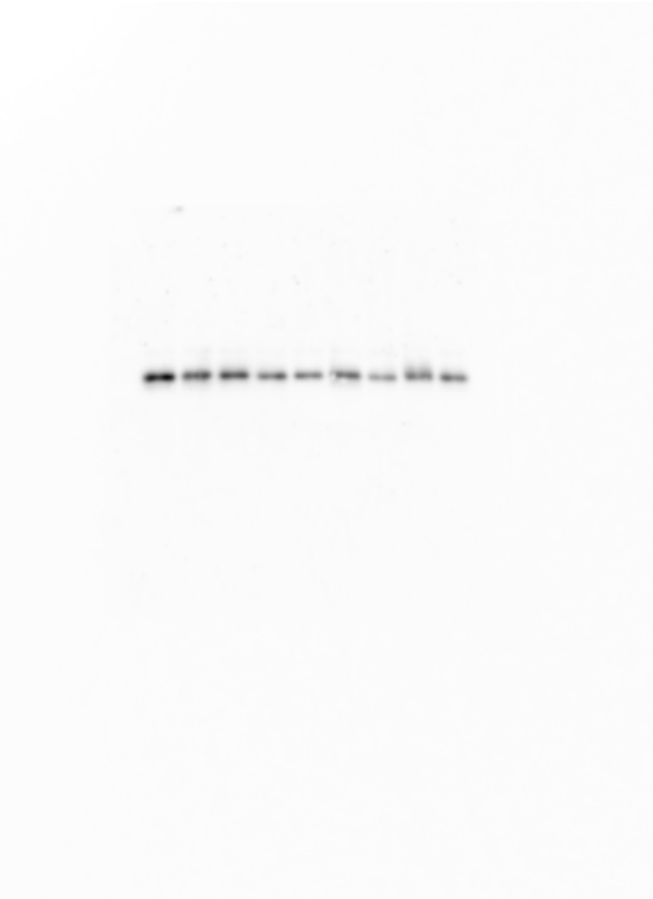

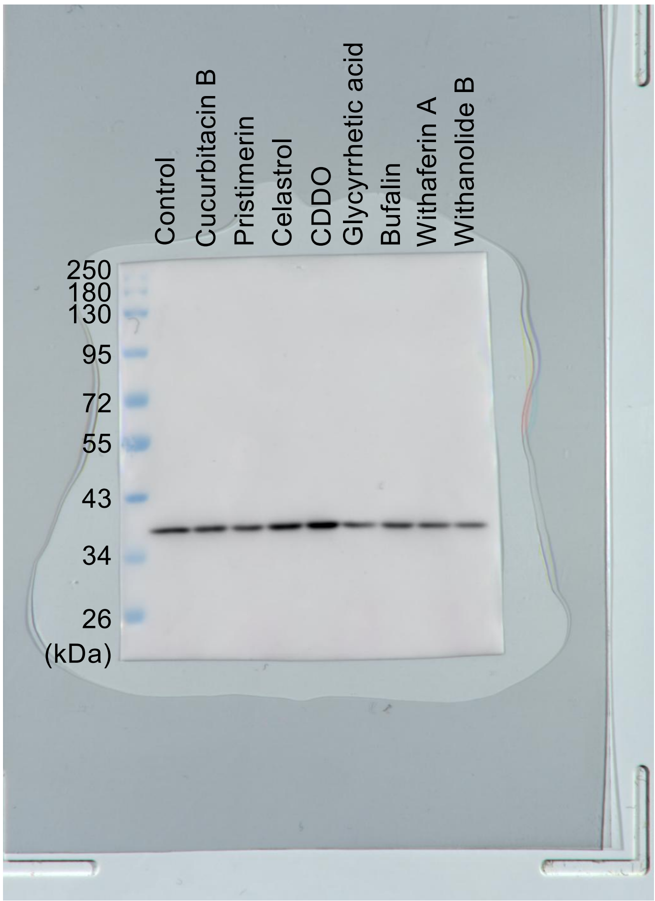


WB: GAPDH (reprobed)

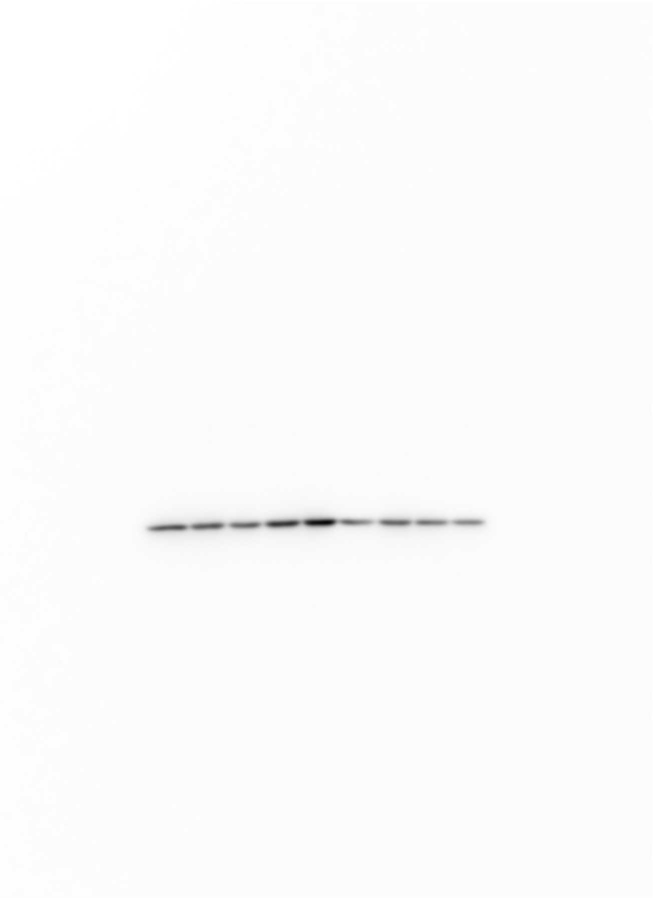

# Figure S37: Original blots in Figure 8A (medium)

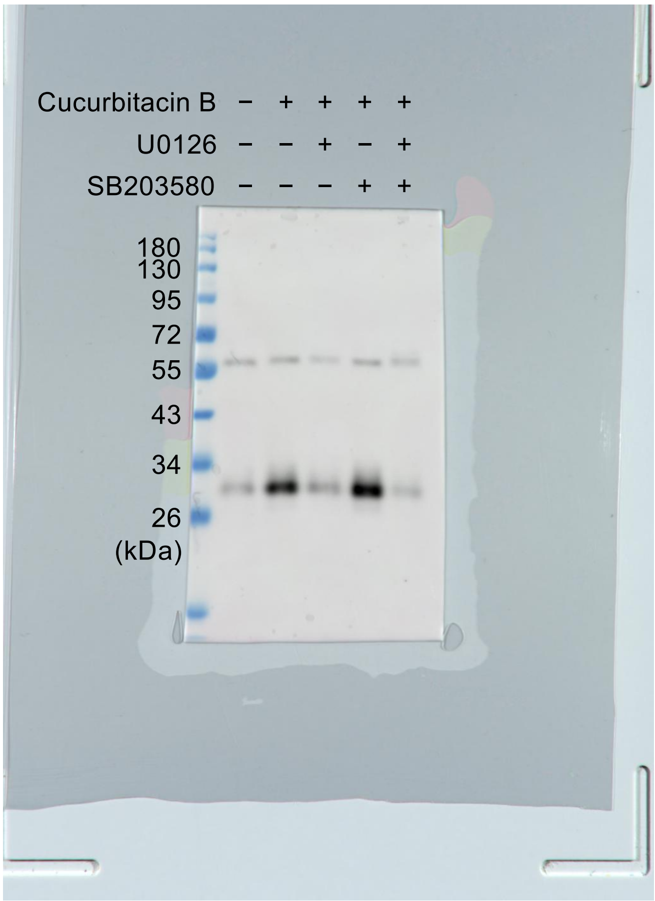


WB: TNF-R1

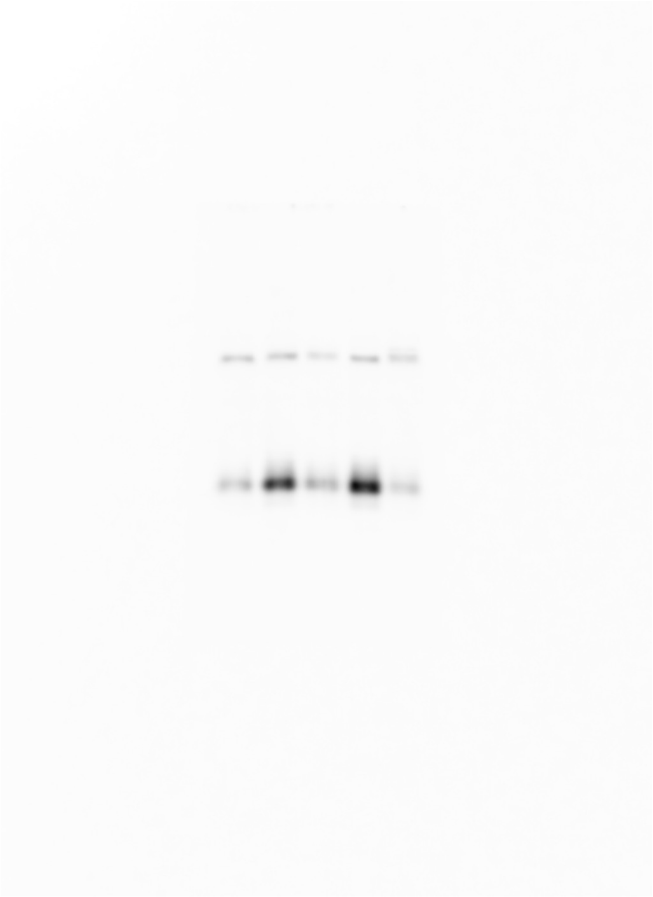

# Figure S38: Original blots in Figure 8A (cell lysate)

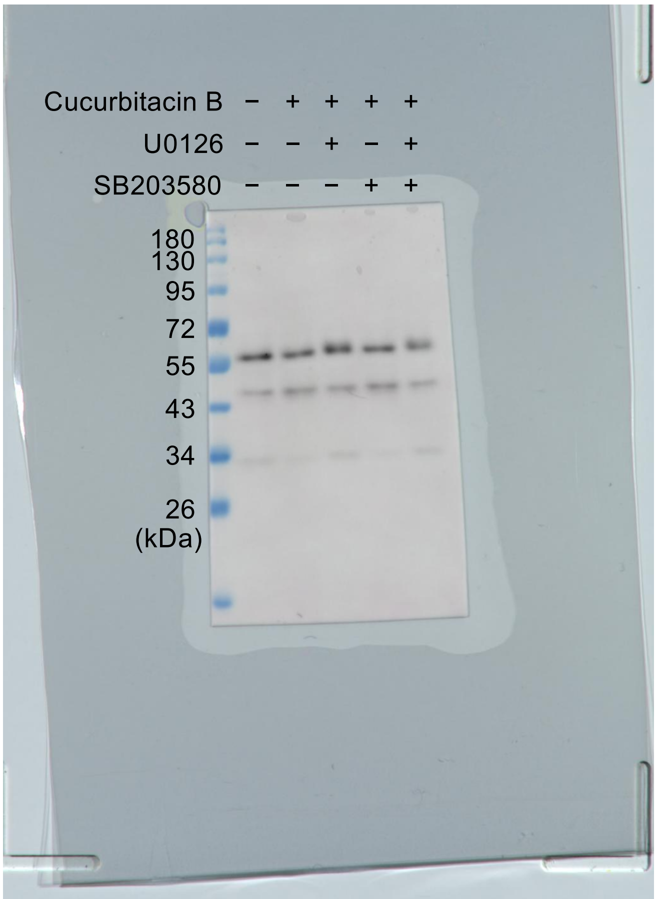


WB: TNF-R1

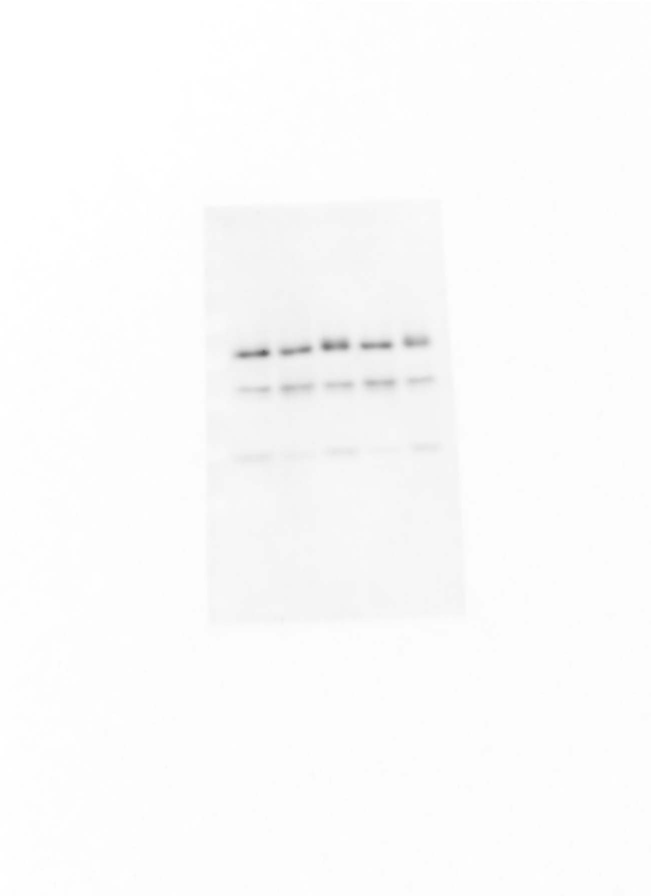

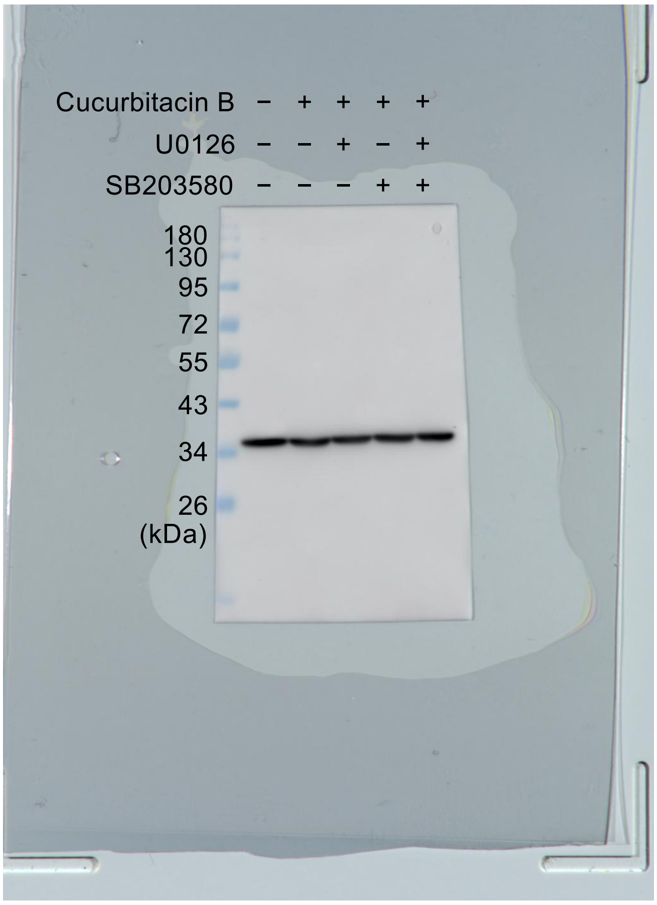


WB: GAPDH (reprobed)

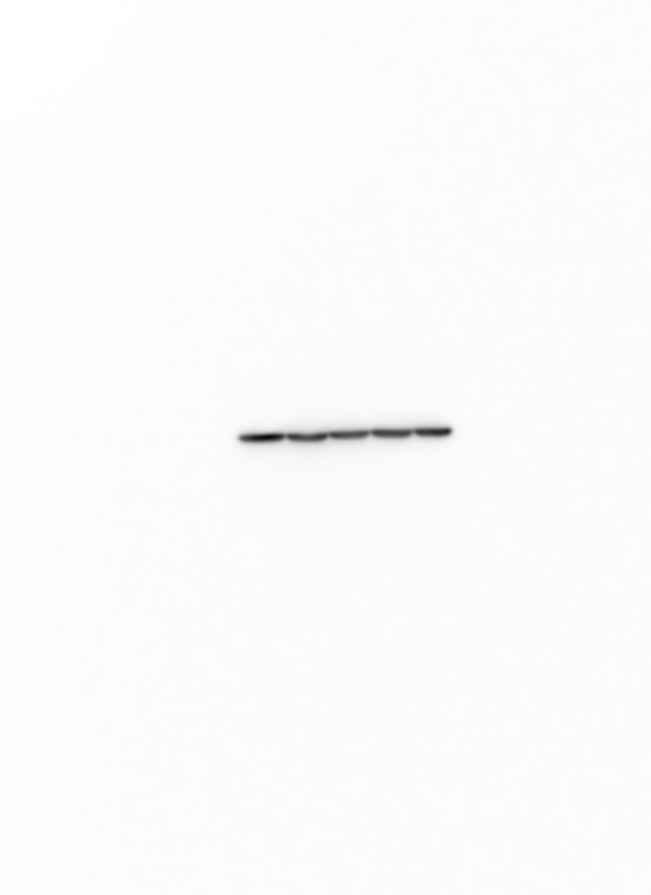

# Figure S39: Original blots (1) in Figure 8B

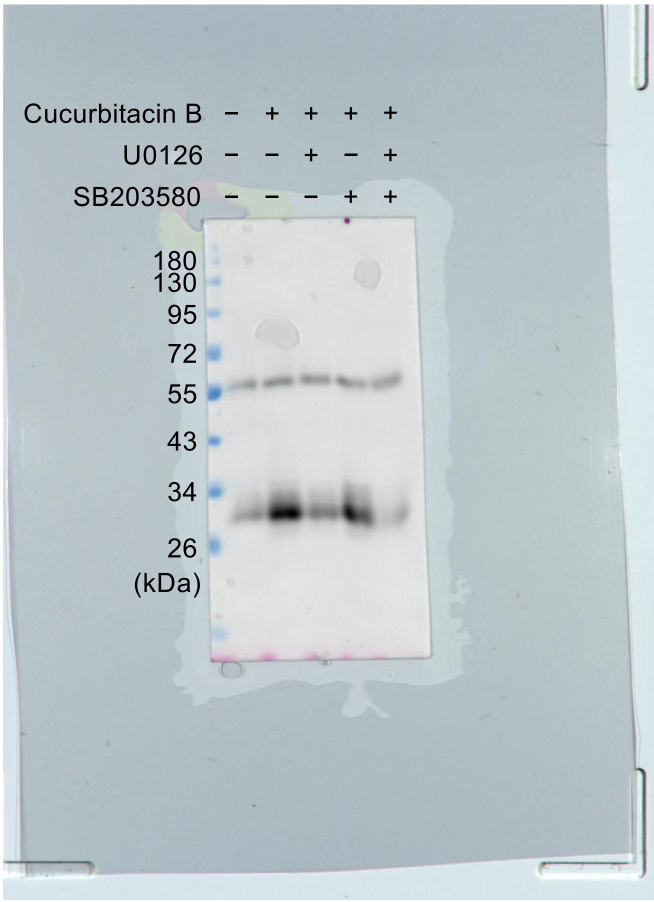


WB: TNF-R1

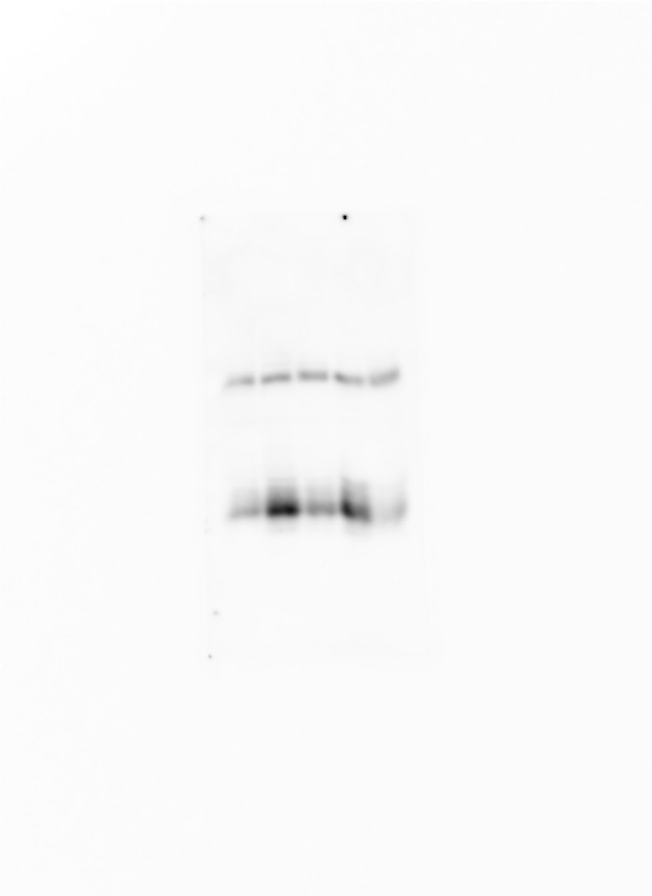

## Figure S40: Original blots (2) in Figure 8B

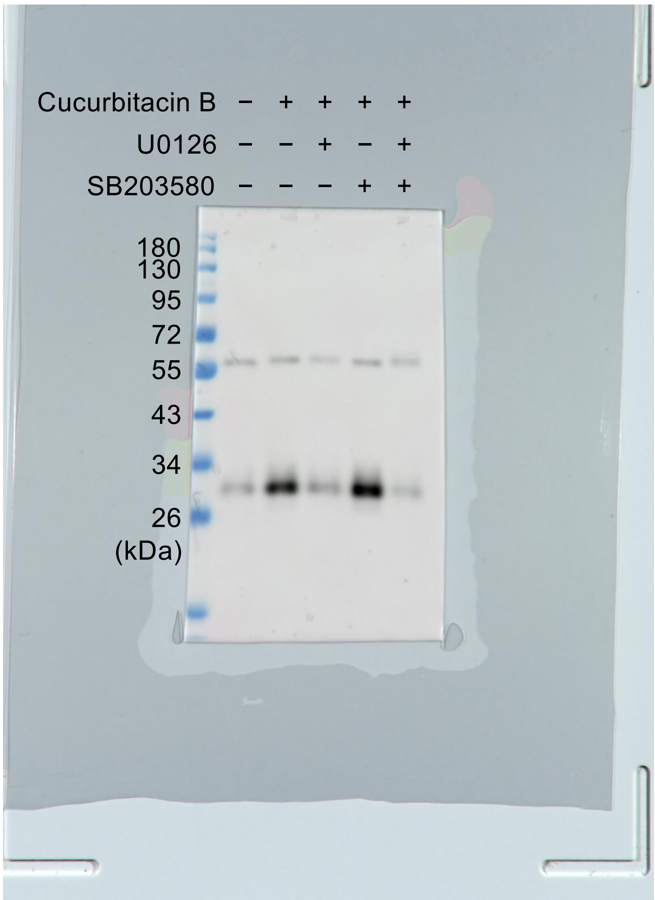


WB: TNF-R1

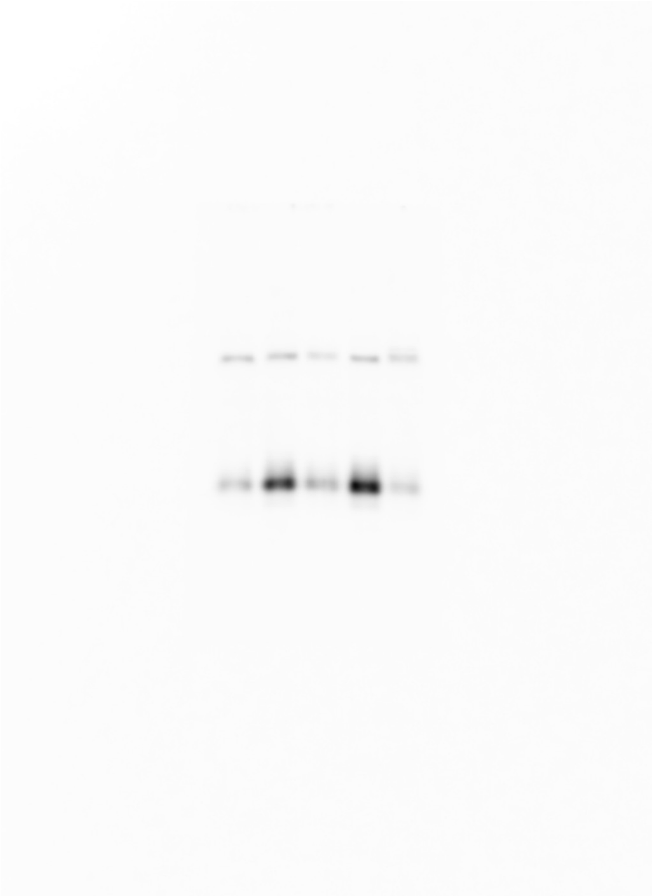

# Figure S41: Original blots (3) in Figure 8B

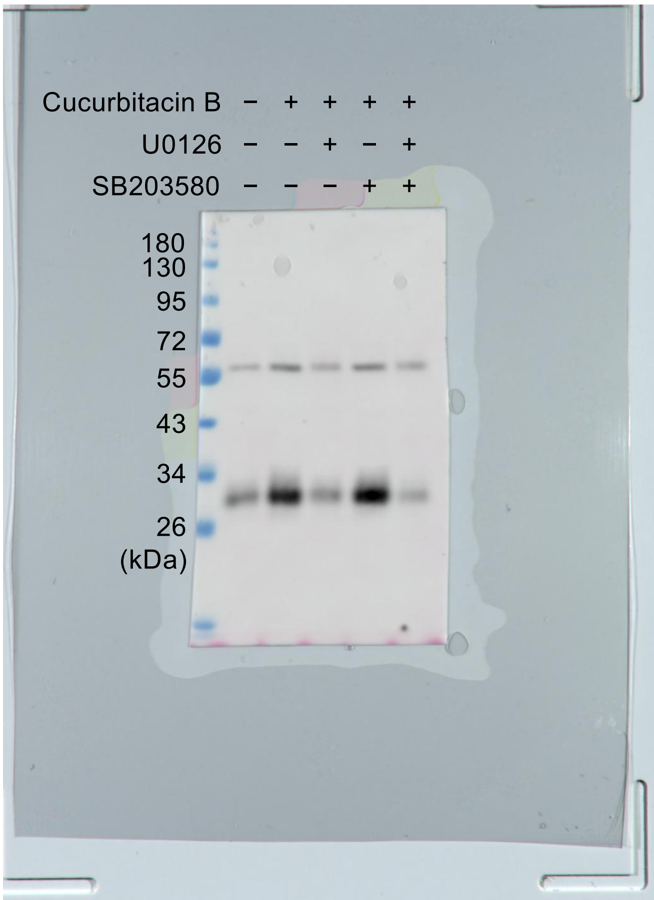


WB: TNF-R1

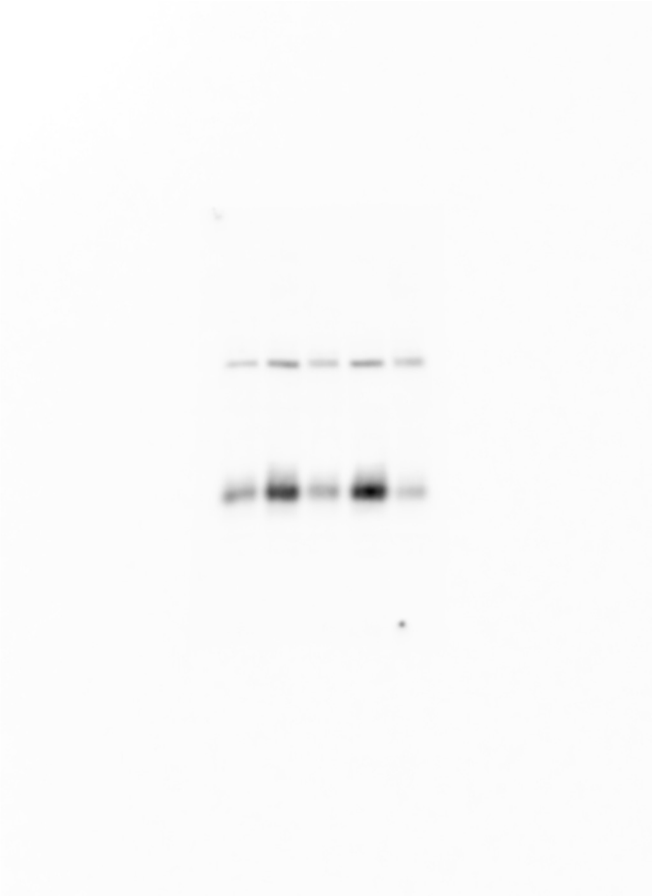

## Figure S42: Original blots (1) in Figure 8C

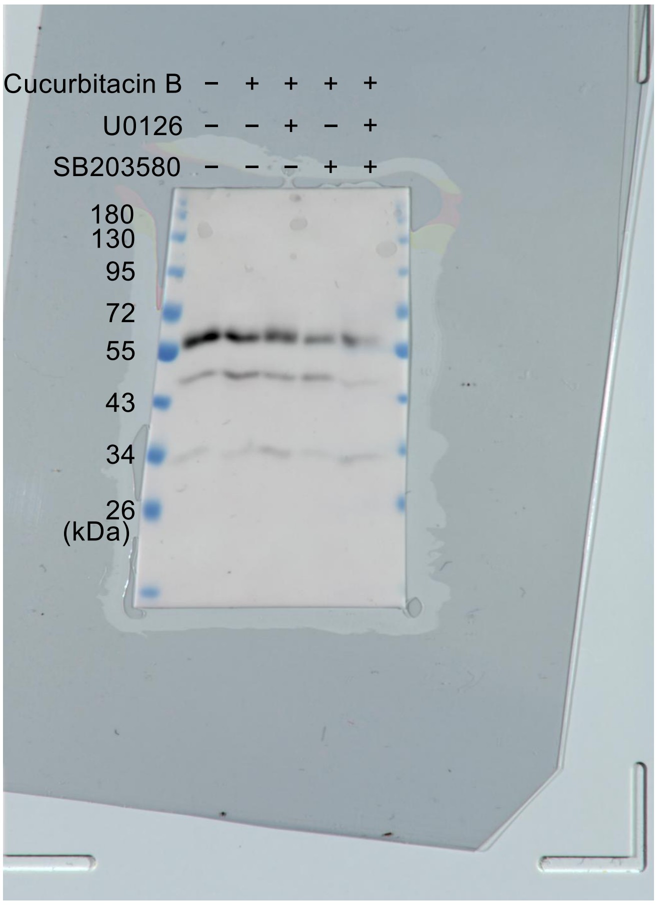


WB: TNF-R1

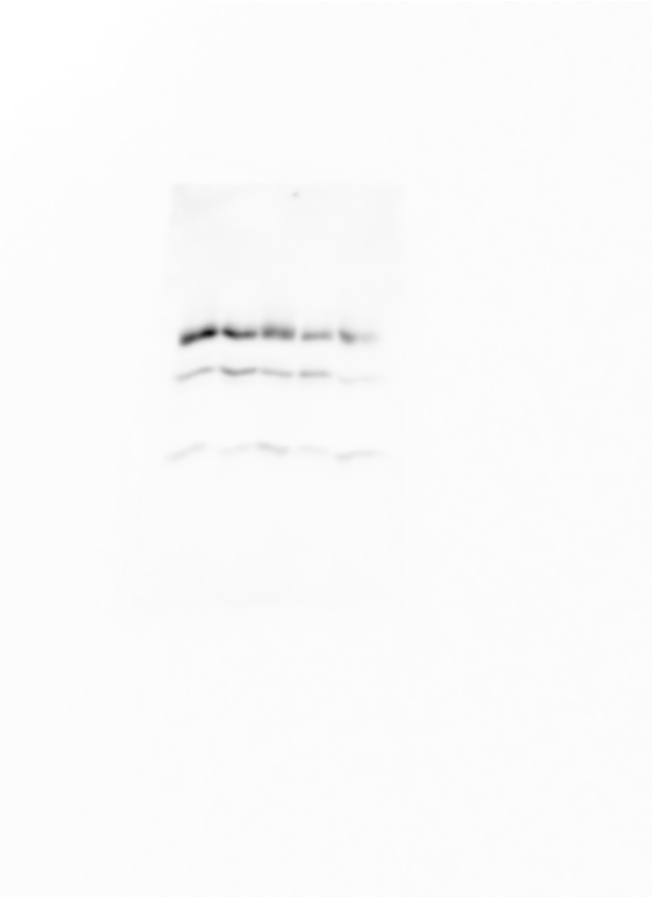

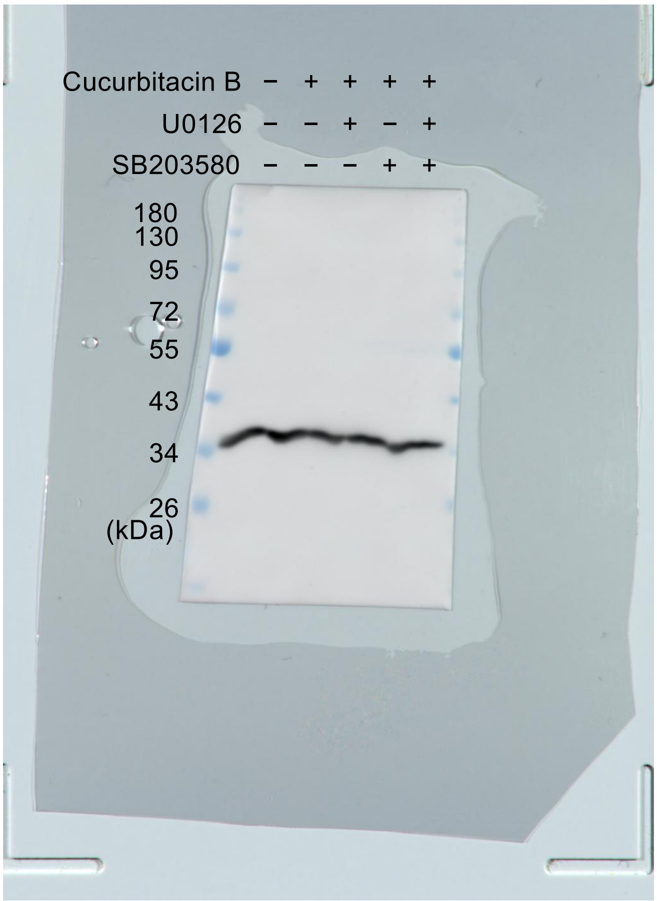


WB: GAPDH (reprobed)

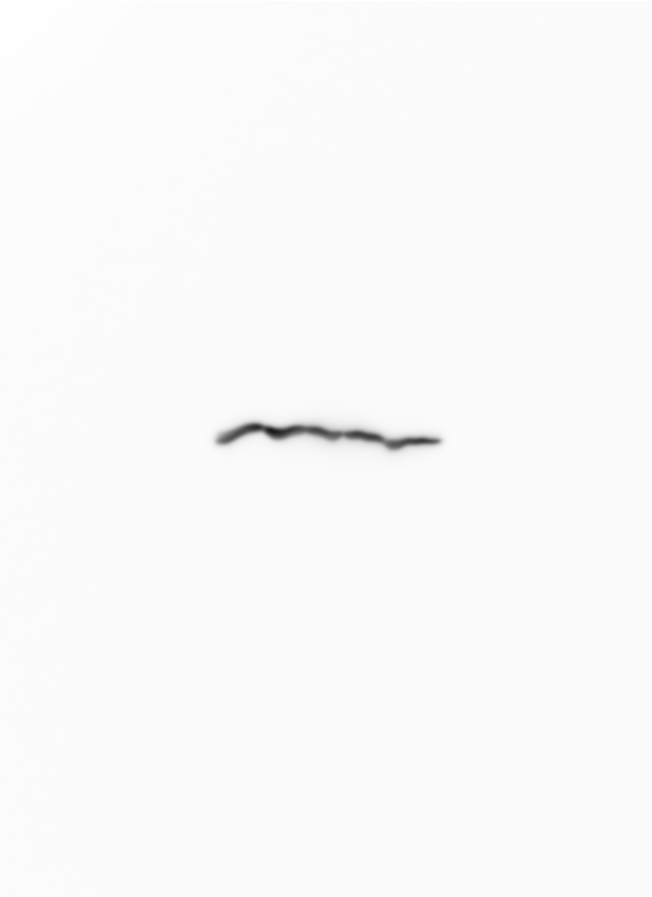

# Figure S43: Original blots (2) in Figure 8C

WB: TNF-R1

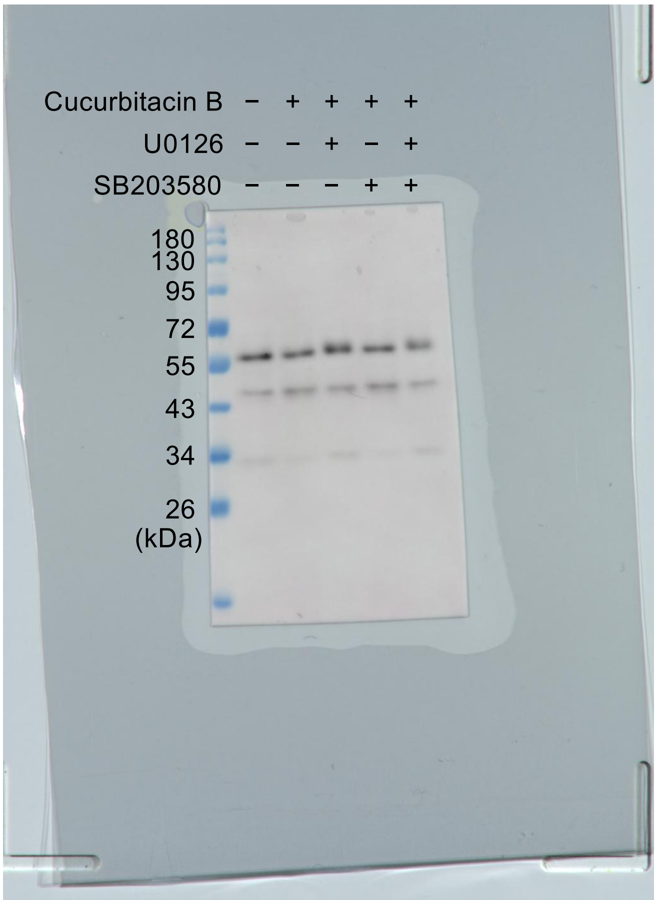


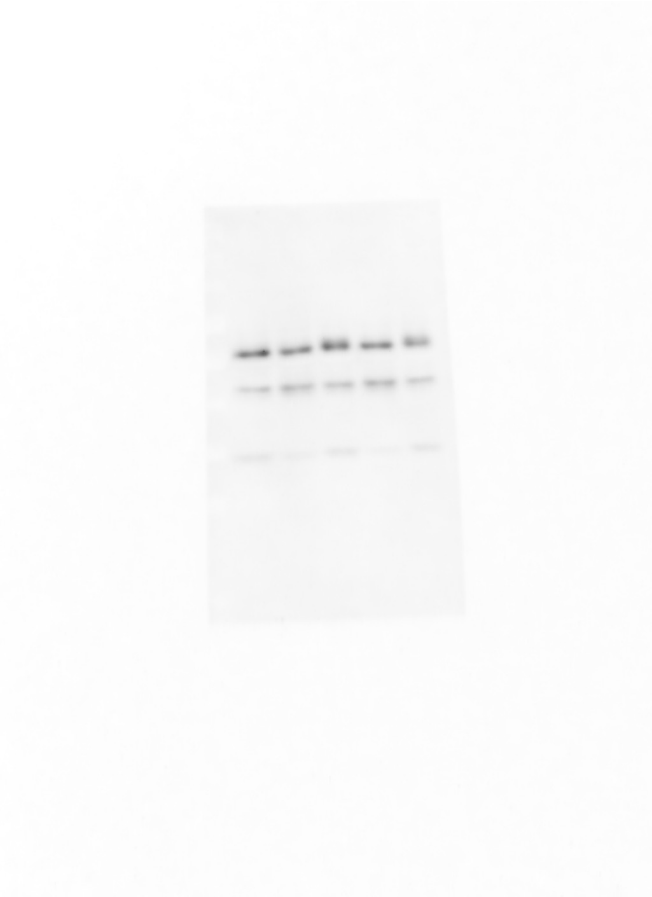

WB: GAPDH (reprobed)

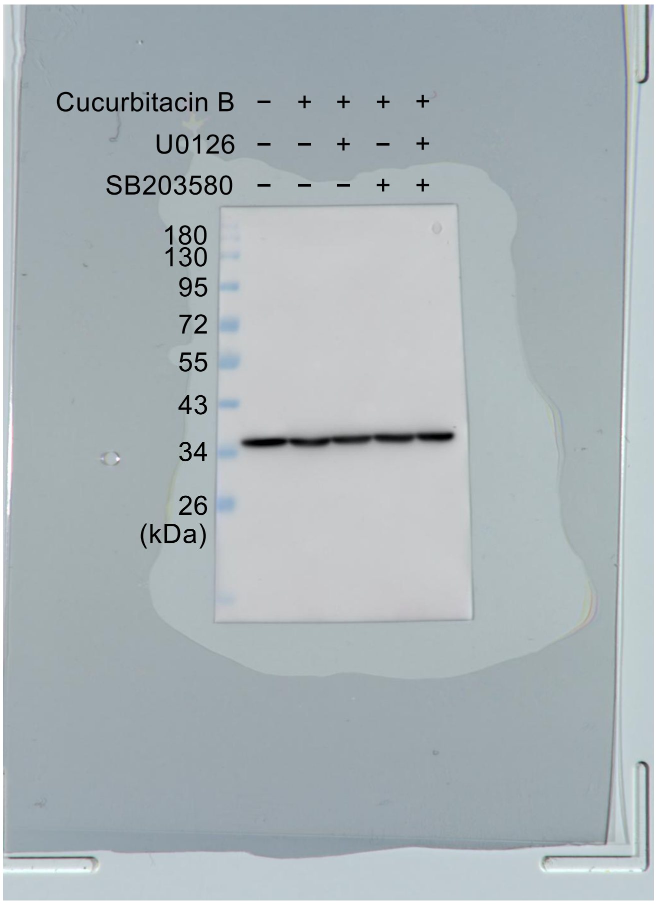


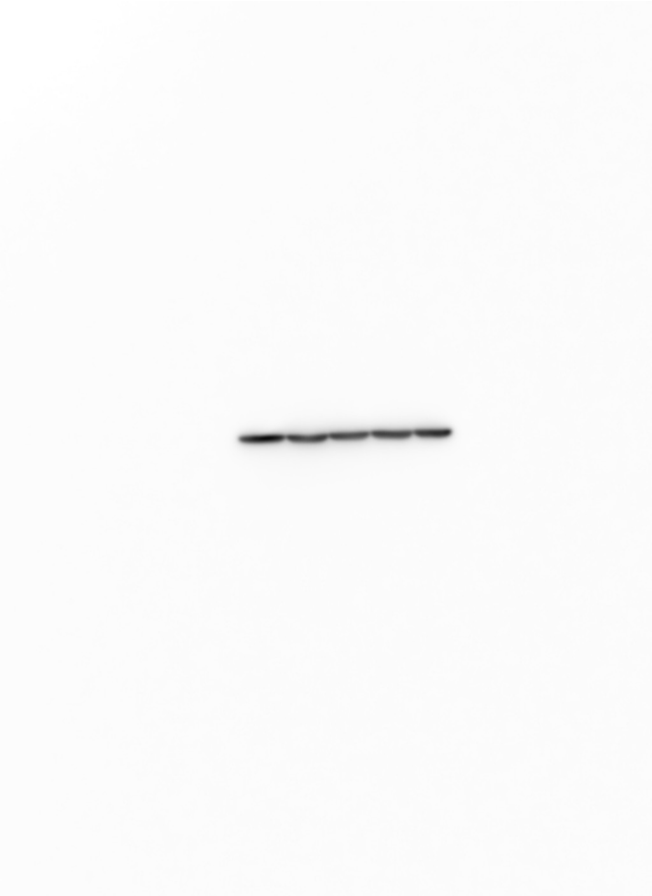

## Figure S44: Original blots (3) in Figure 8C

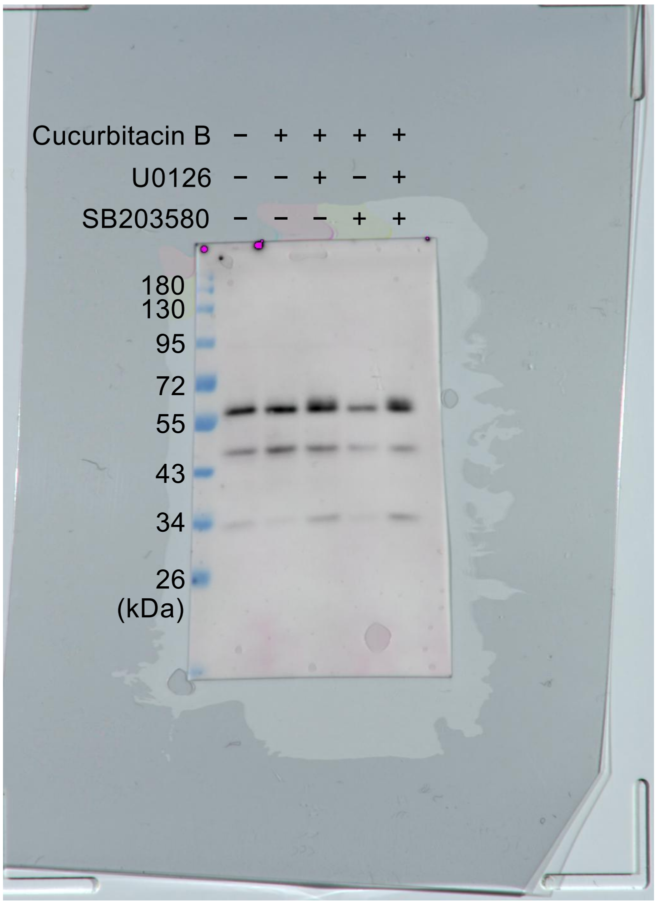


WB: TNF-R1

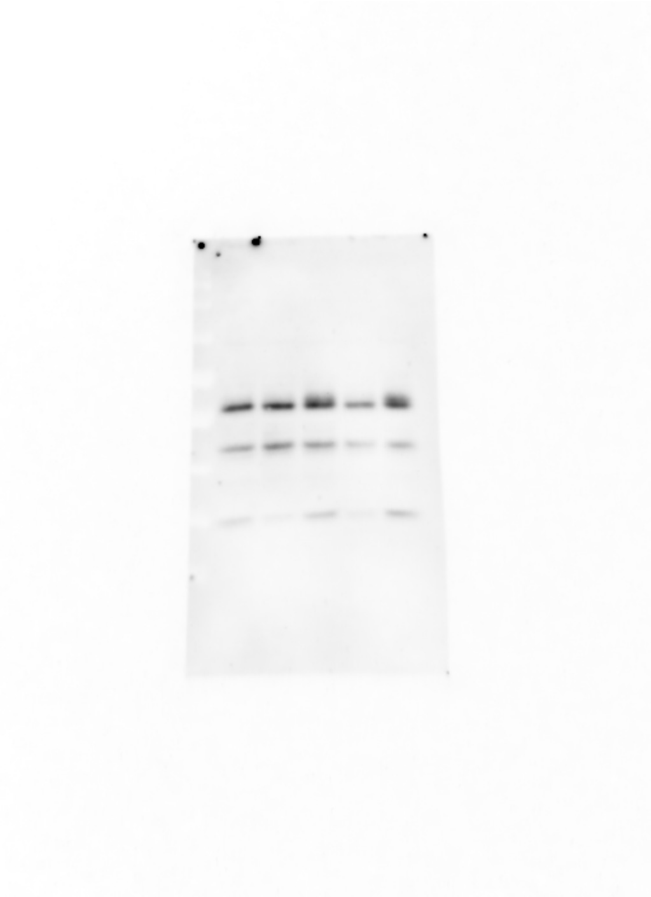

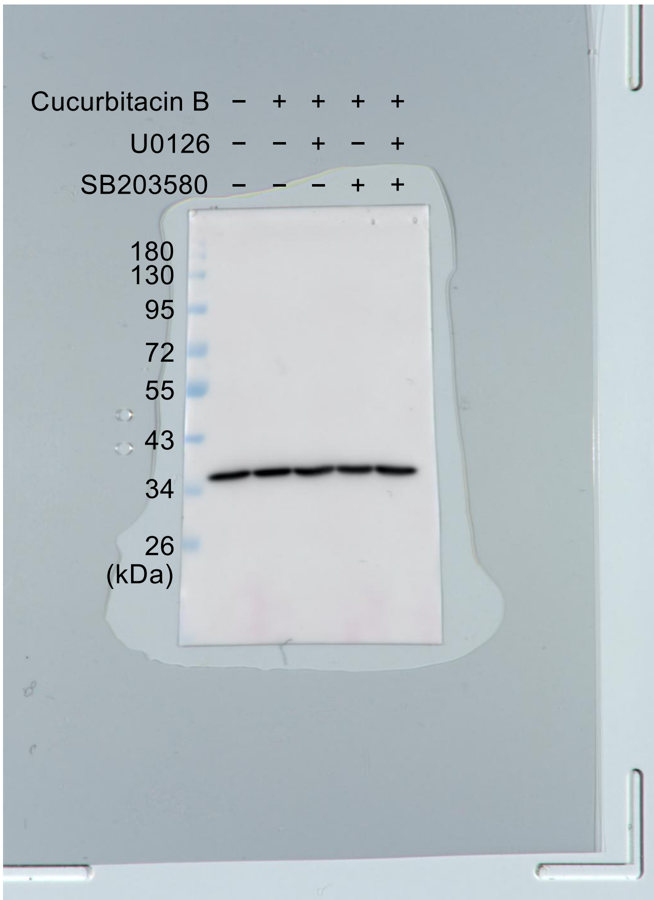


WB: GAPDH (reprobed)

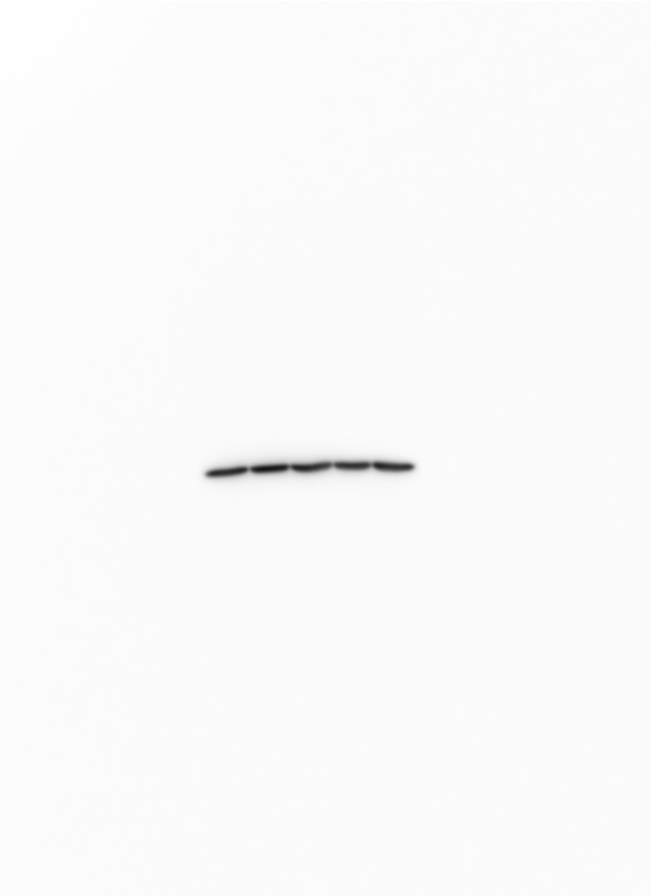

# Figure S45: Original blots in Figure 8D (medium)

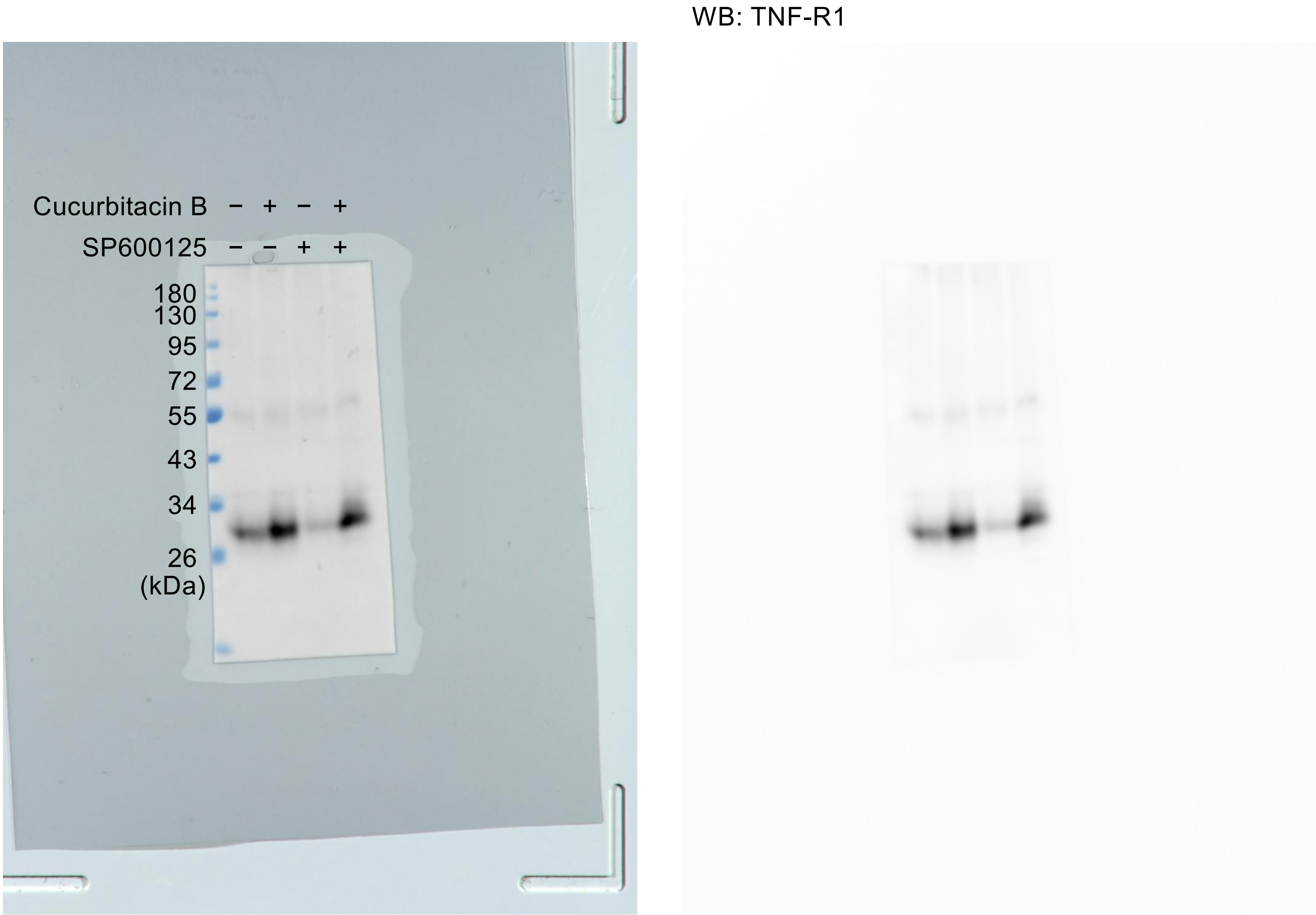

**Figure S46: Original blots in Figure 8D (cell lysate)**

WB: TNF-R1

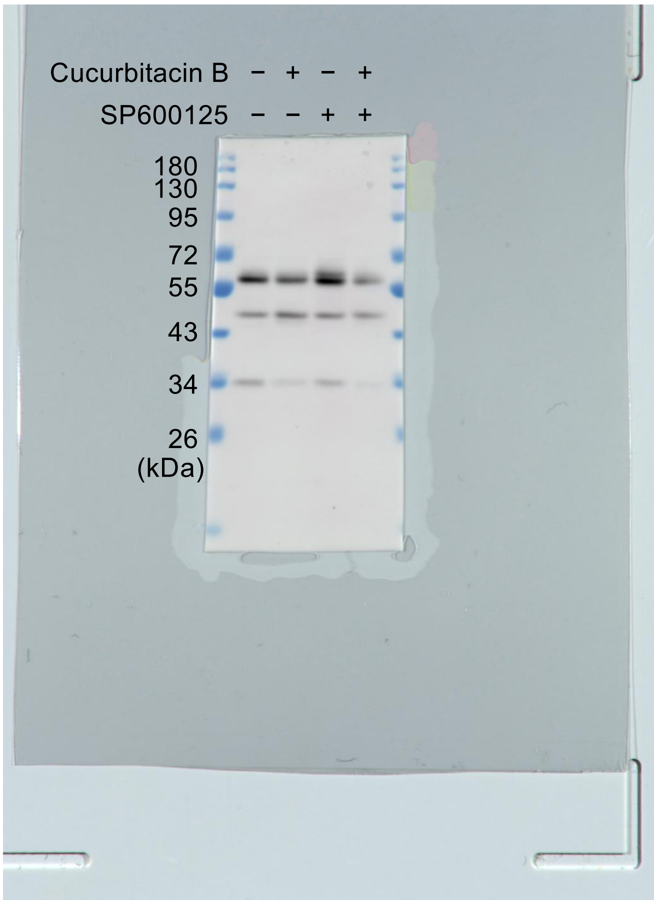


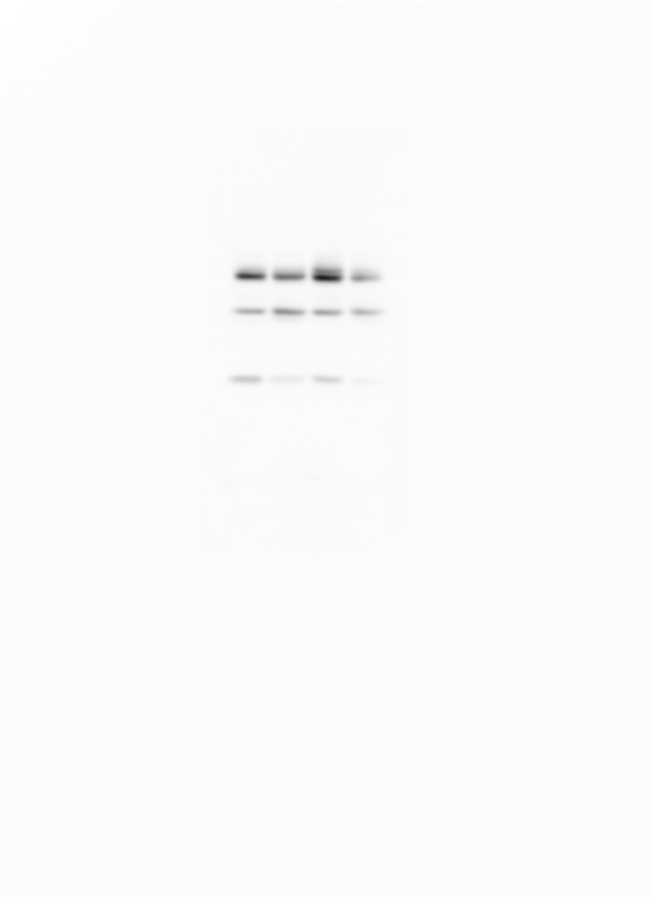

WB: GAPDH (reprobed)

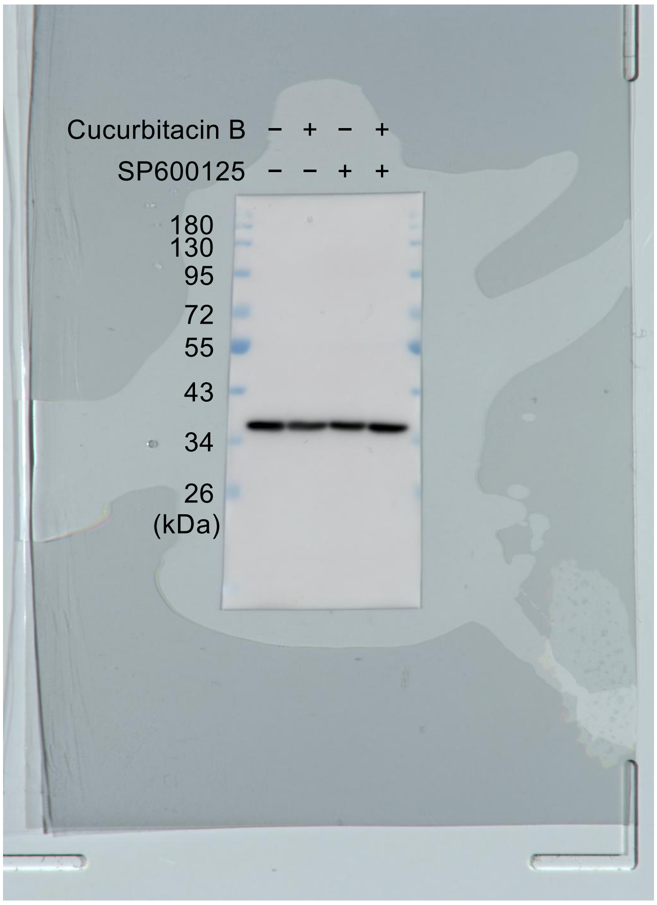


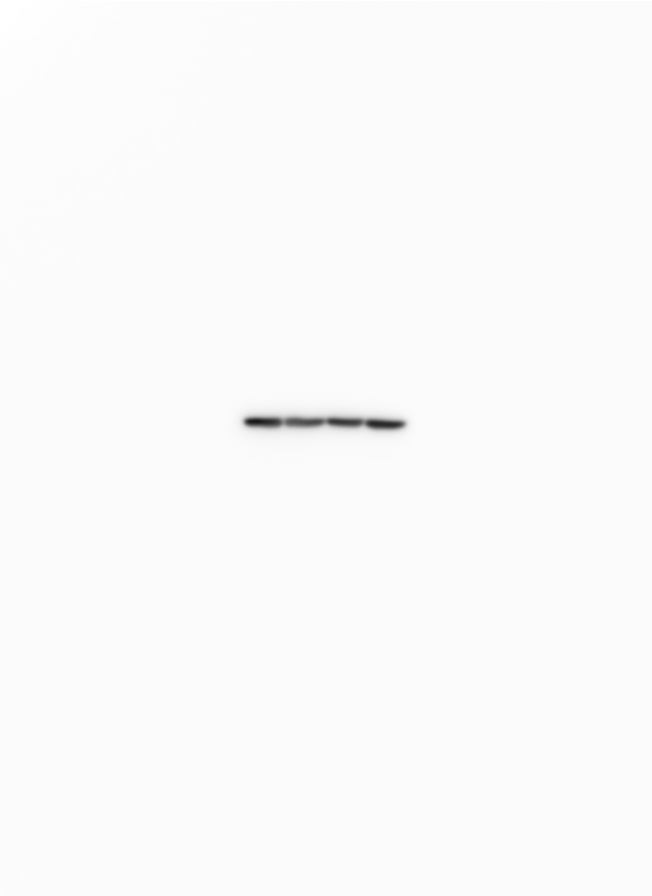

# Figure S47: Original blots (1) in Figure 8E

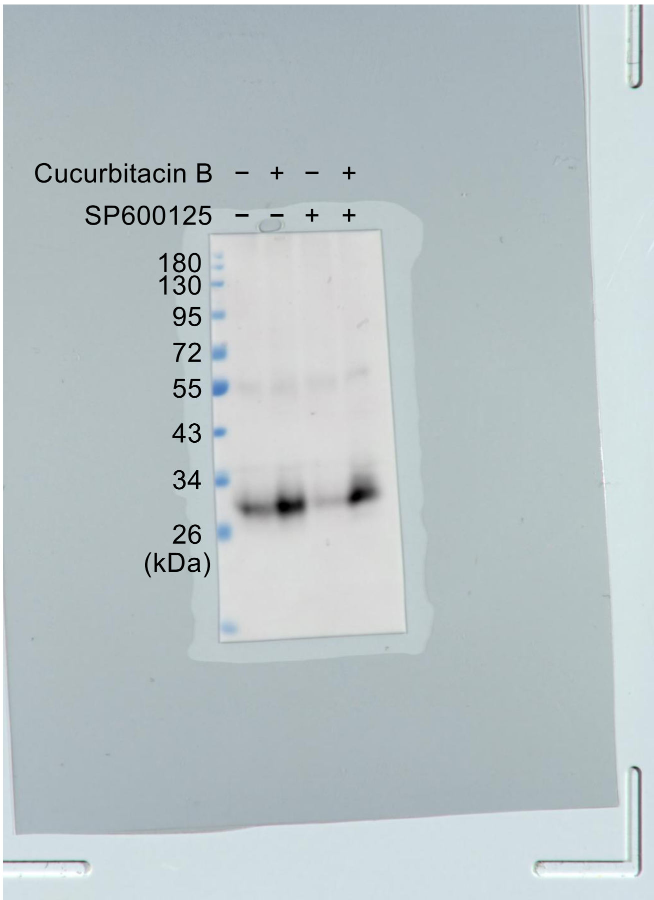


WB: TNF-R1

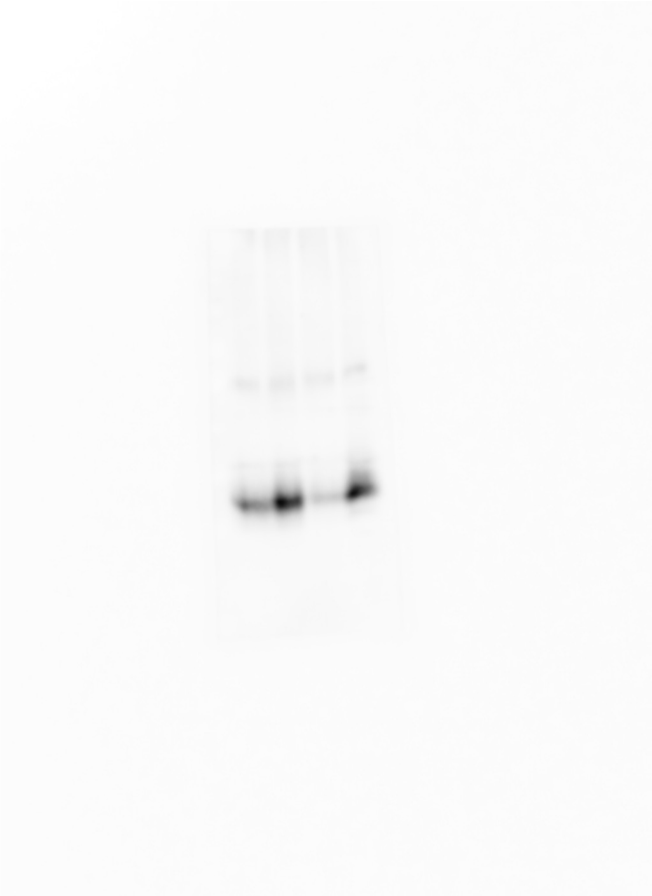

# Figure S48: Original blots (2) in Figure 8E

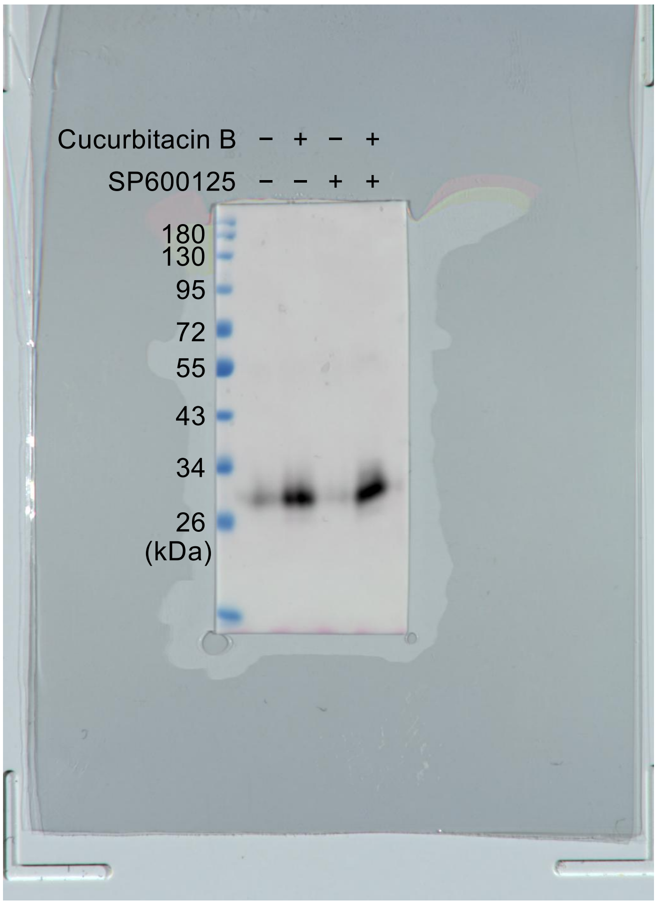


WB: TNF-R1

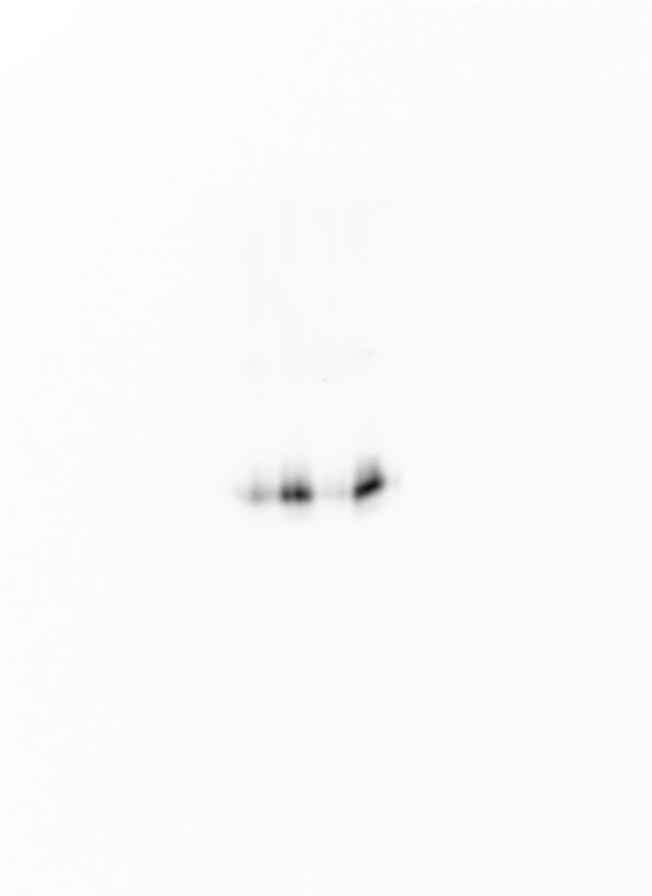

# Figure S49: Original blots (3) in Figure 8E

WB: TNF-R1

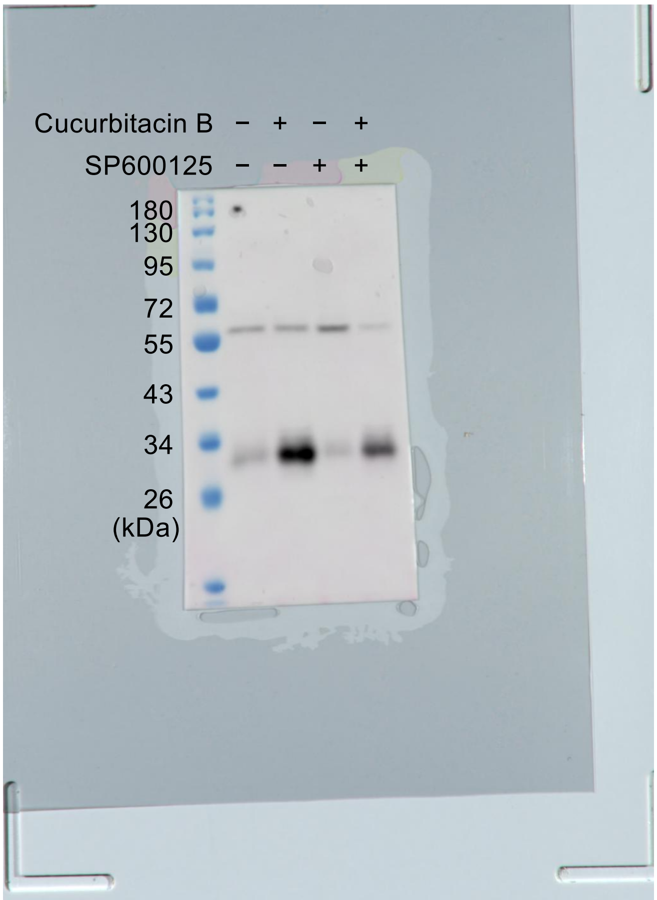


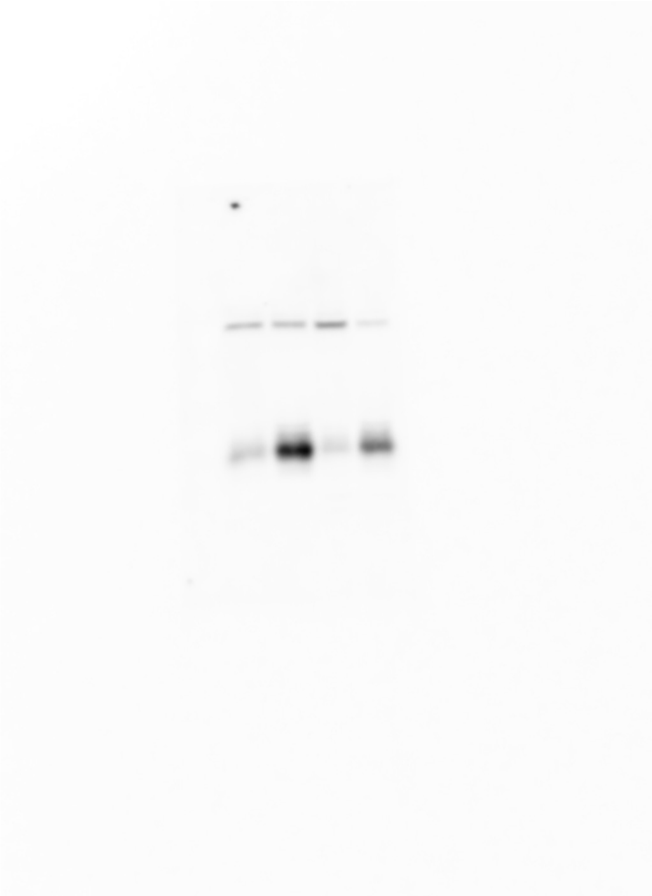

# Figure S50: Original blots (1) in Figure 8F

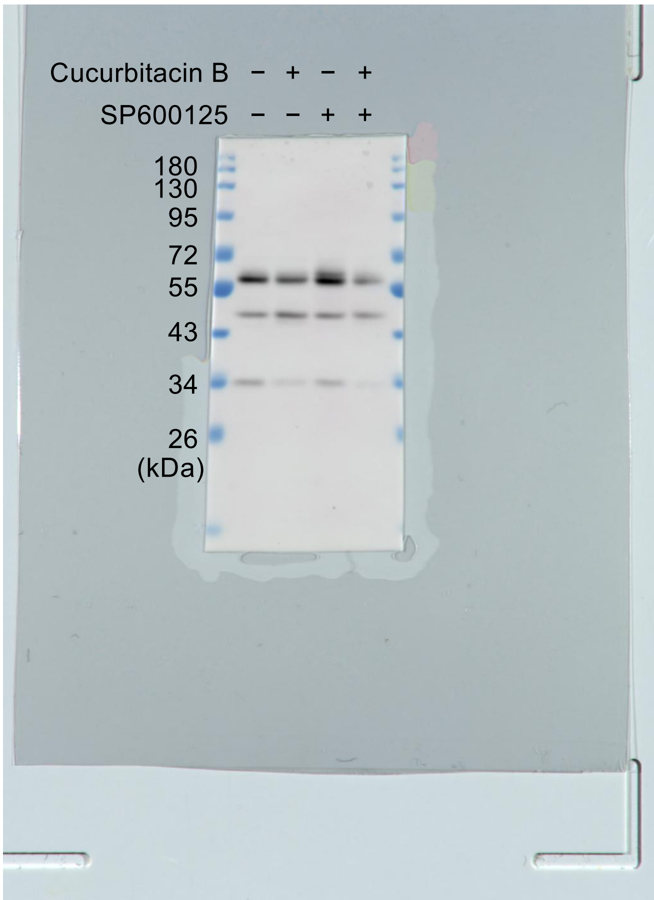


WB: TNF-R1

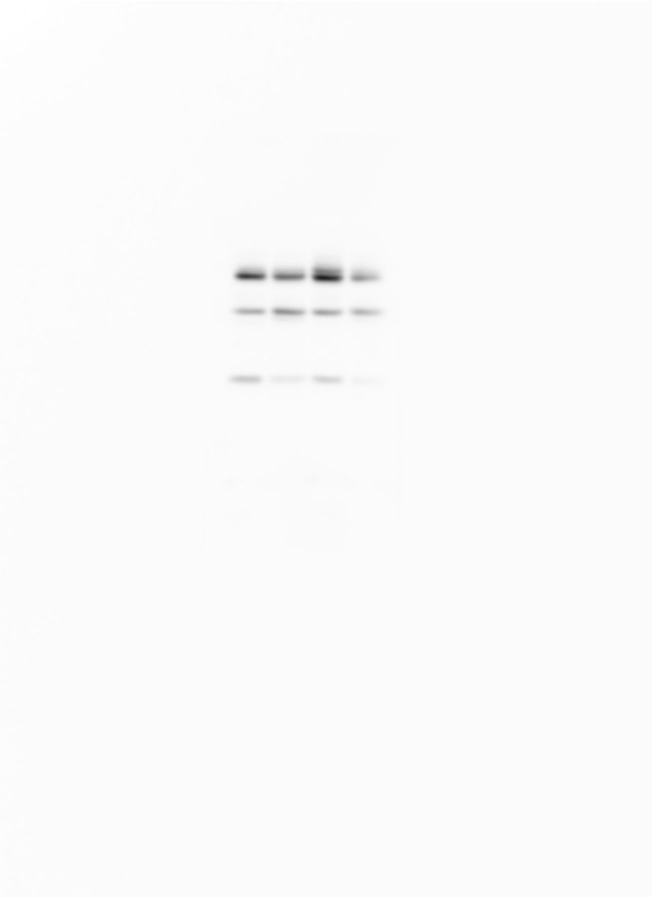

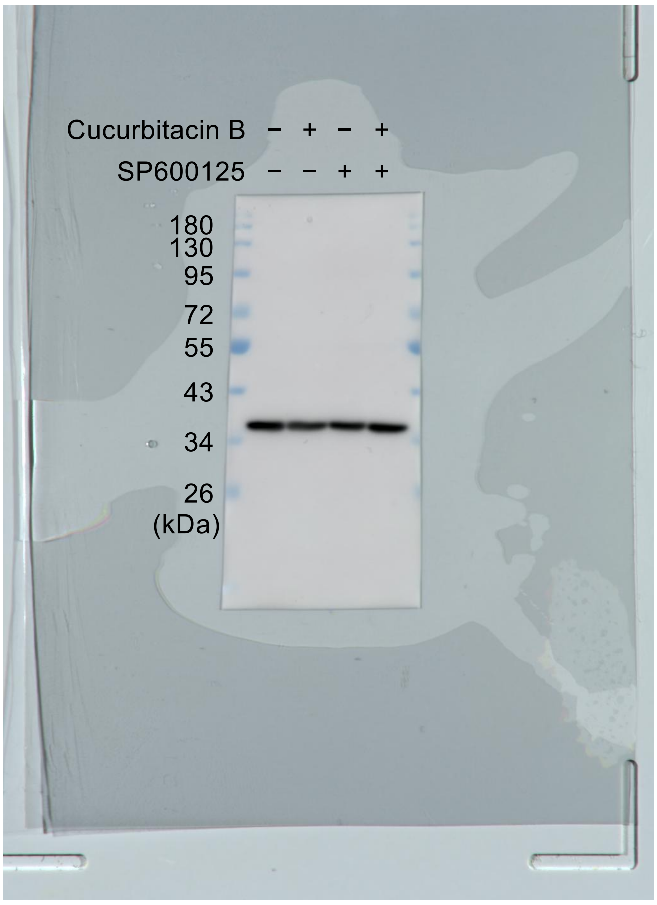


WB: GAPDH (reprobed)

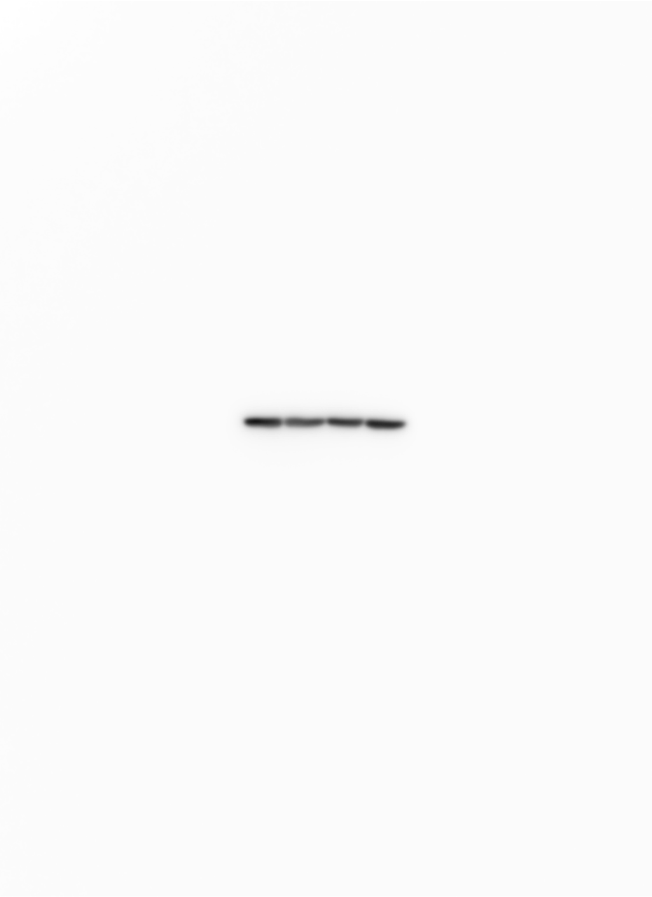

# Figure S51: Original blots (2) in Figure 8F

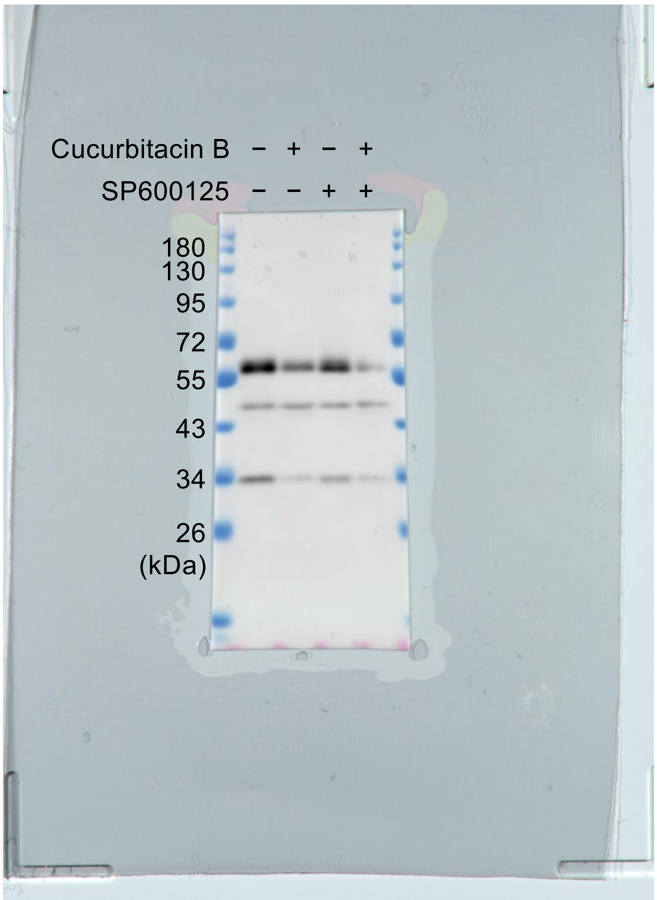


WB: TNF-R1

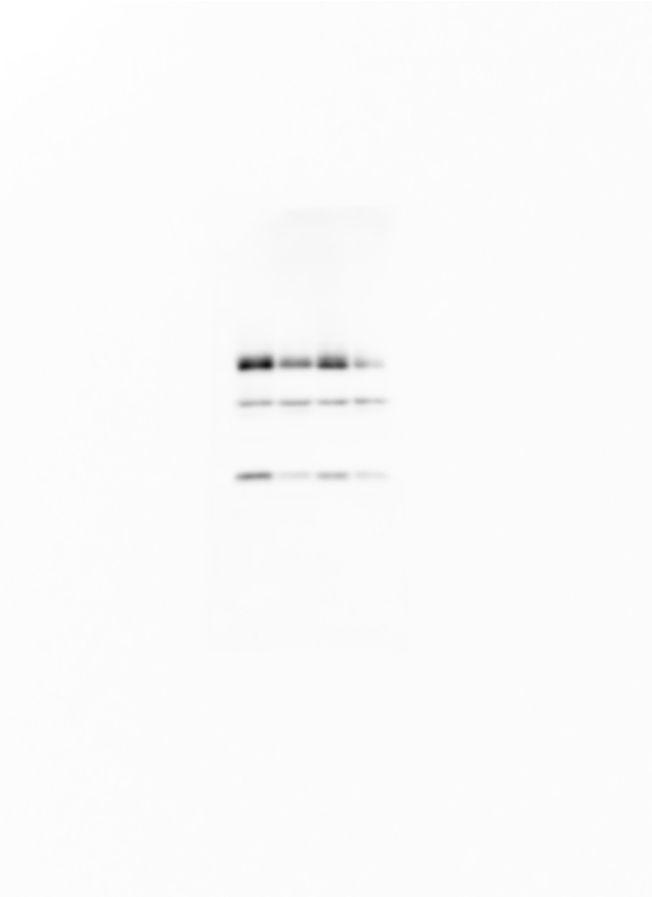

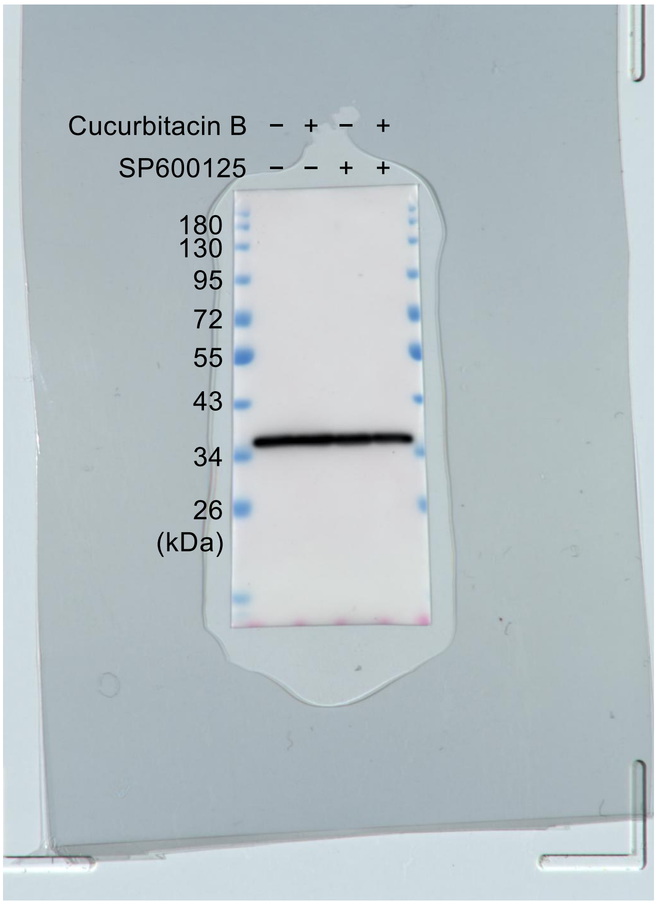


WB: GAPDH (reprobed)

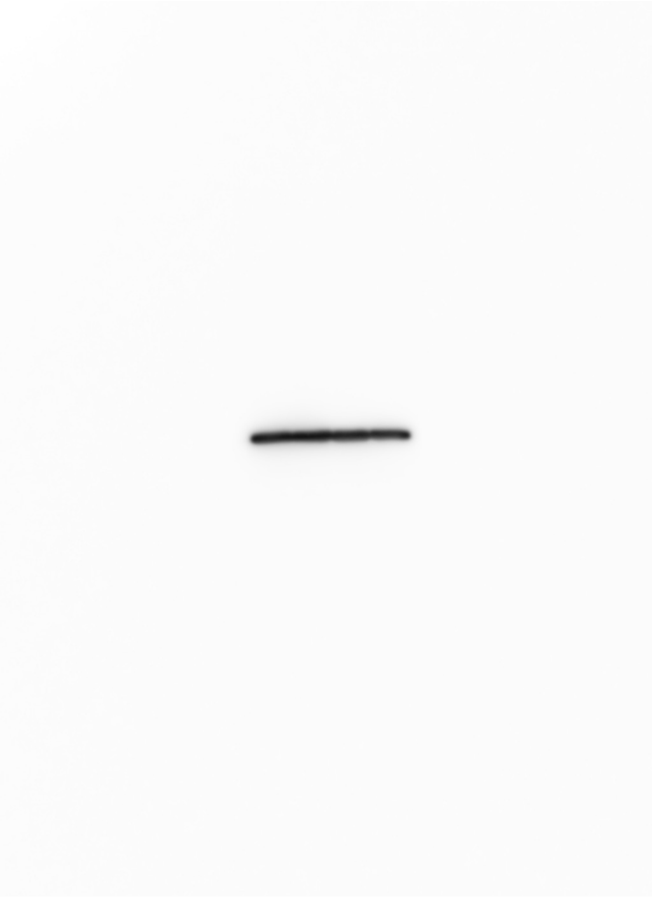

# Figure S52: Original blots (3) in Figure 8F

WB: TNF-R1

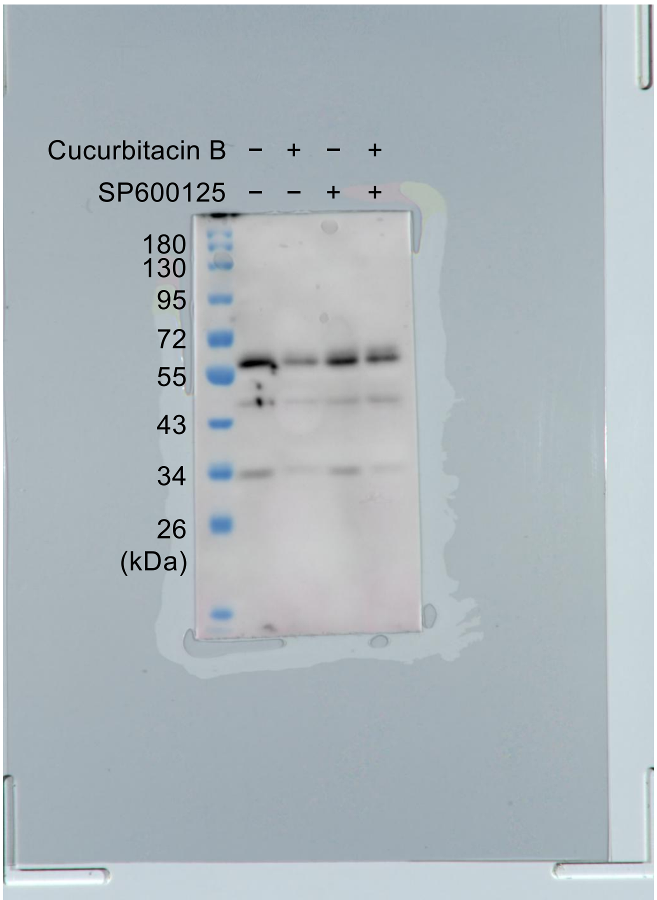


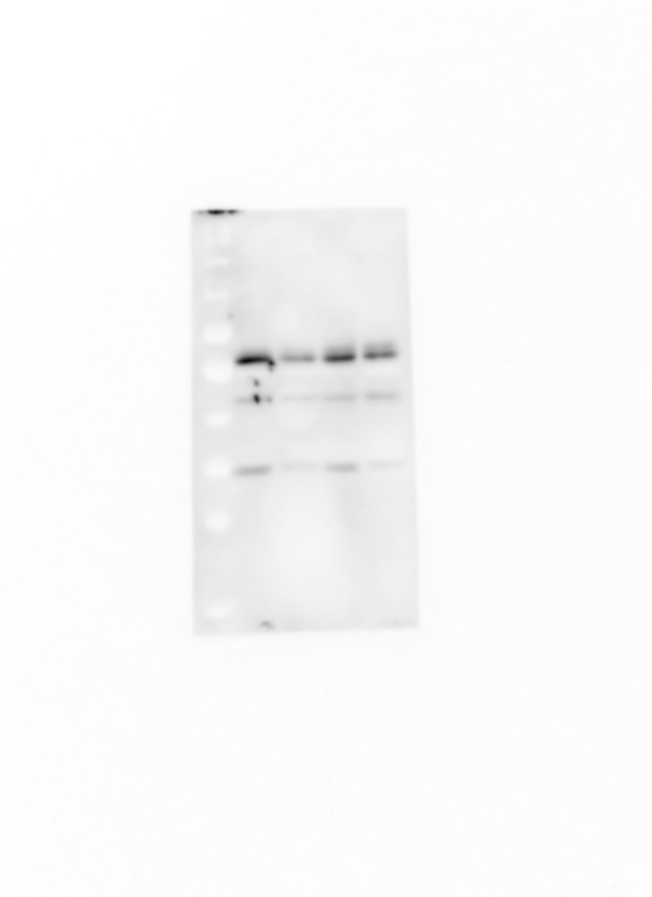

WB: GAPDH (reprobed)

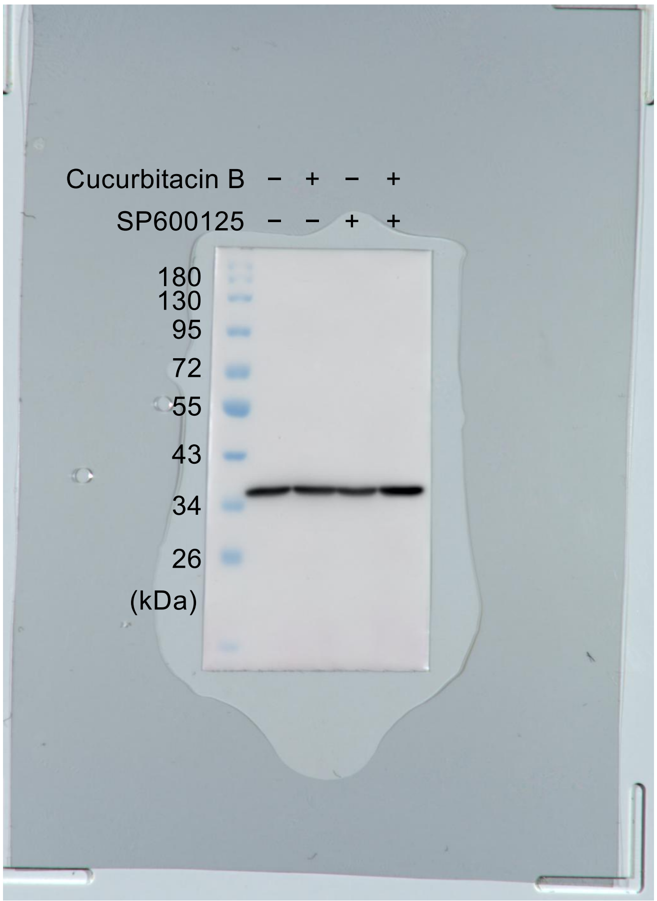


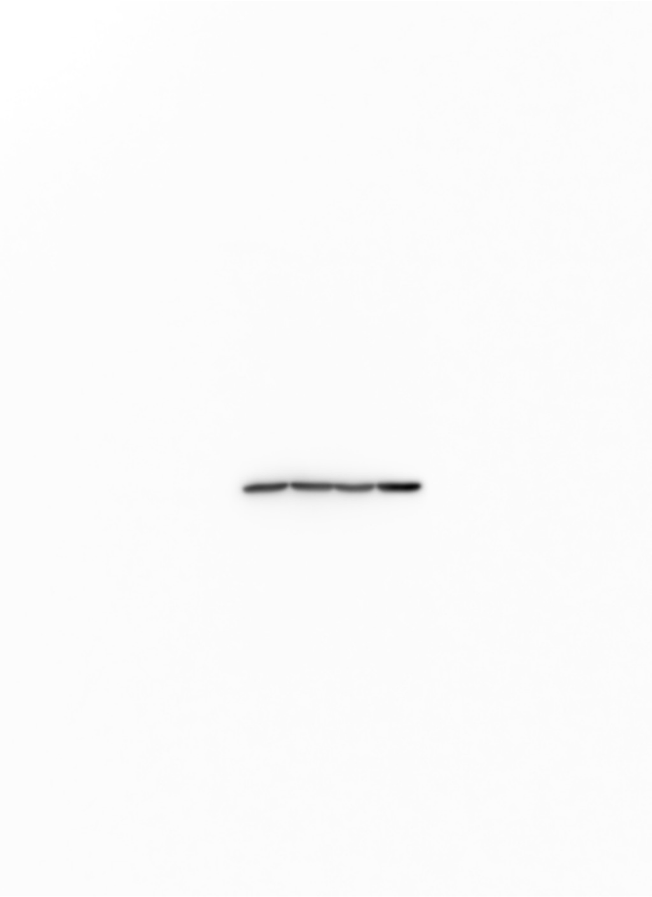

# Figure S53: Original blots in Figure 9A (phospho-ERK)

WB: Phospho-ERK

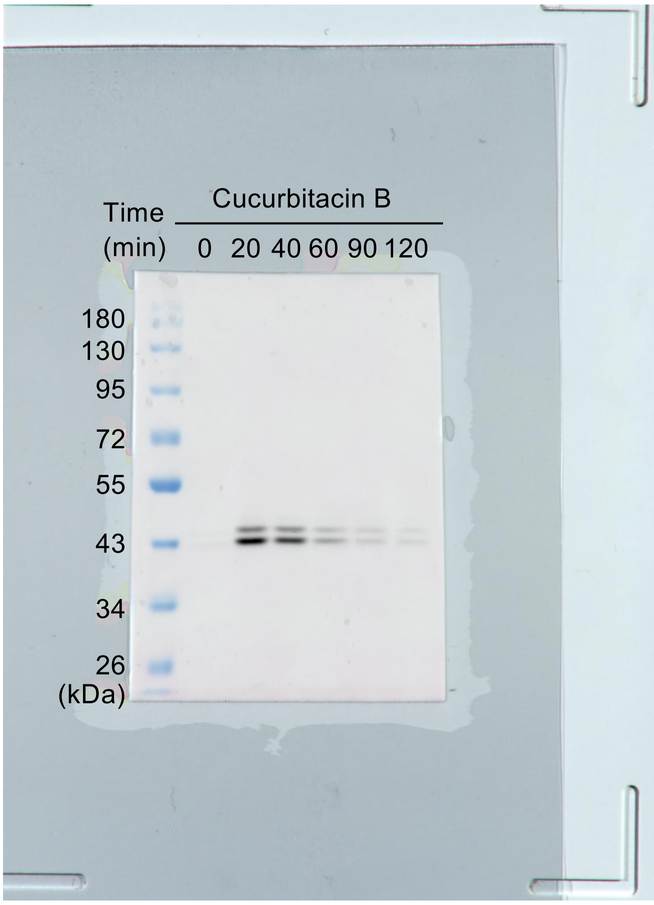


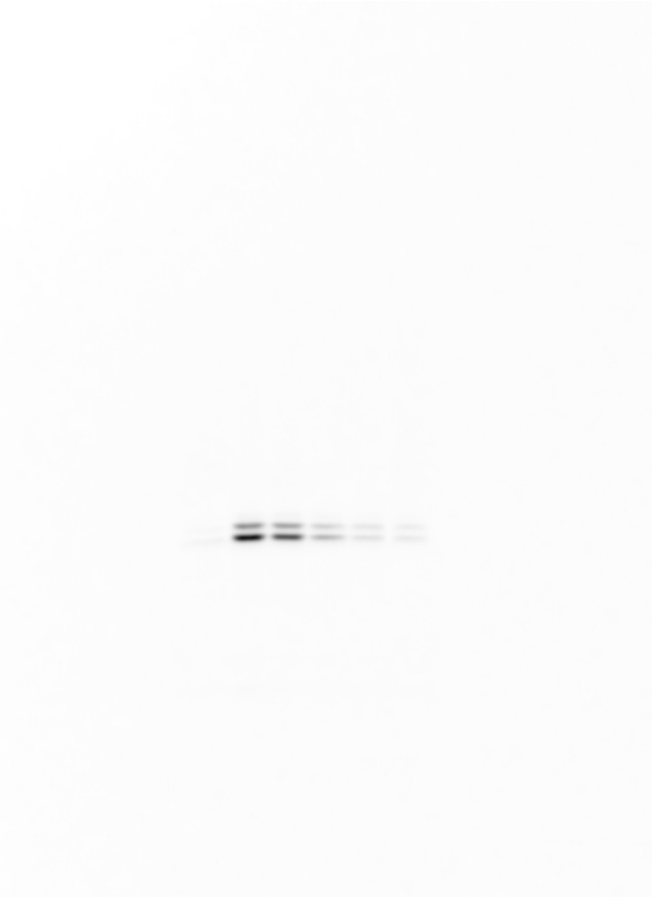

WB: GAPDH (reprobed)

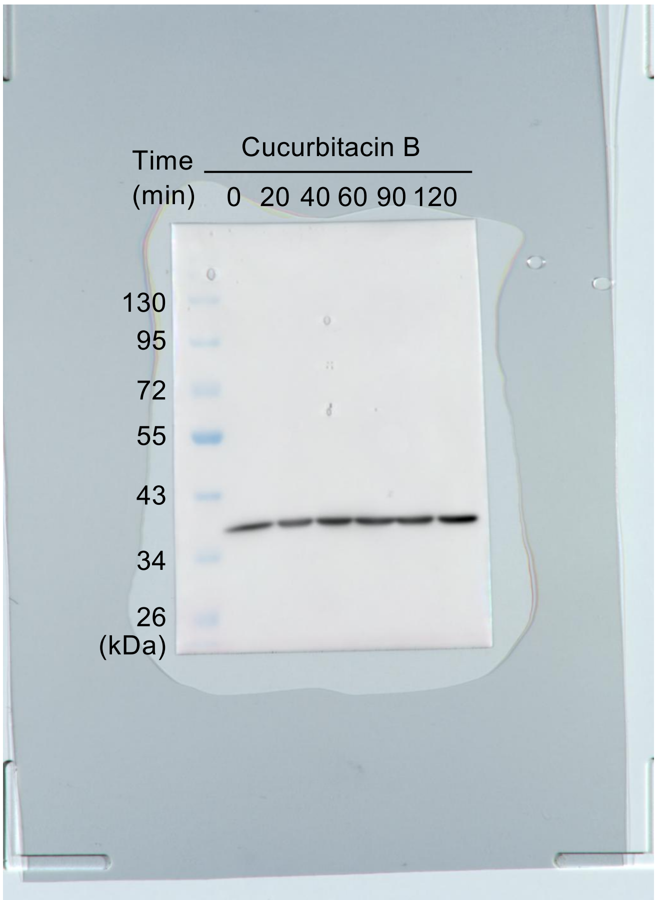


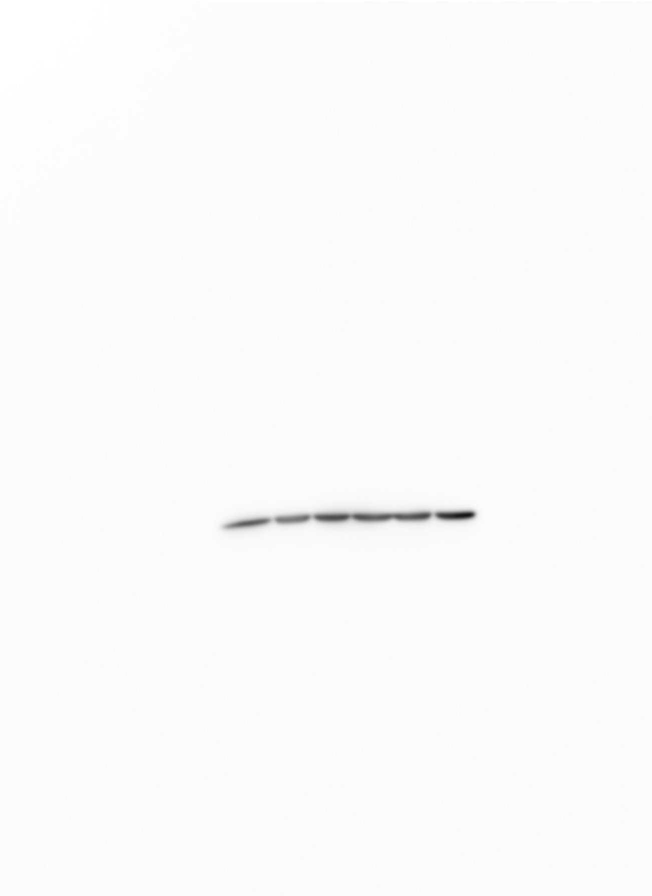

## Figure S54: Original blots in Figure 9A (ERK)

WB: ERK

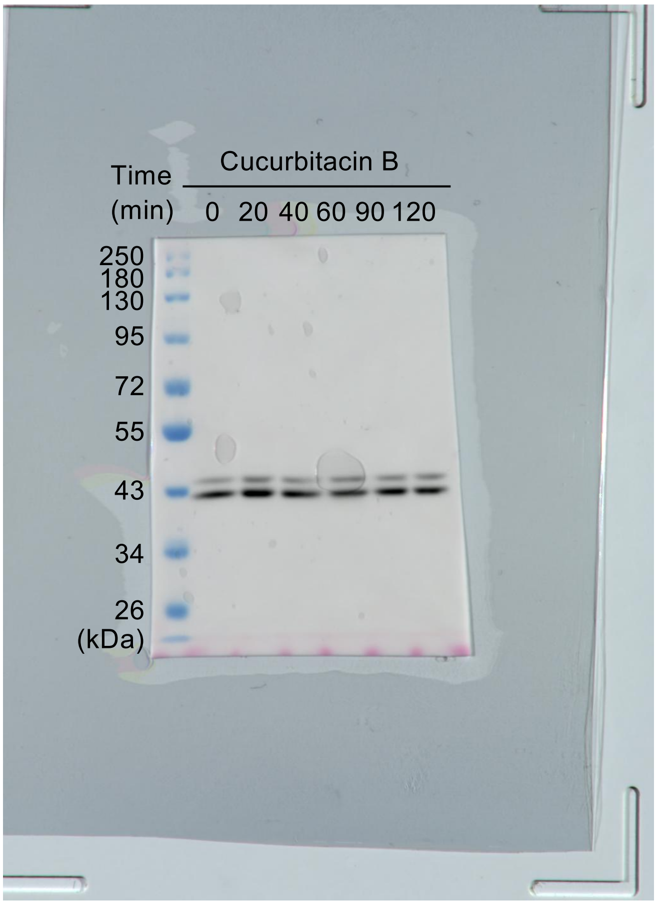


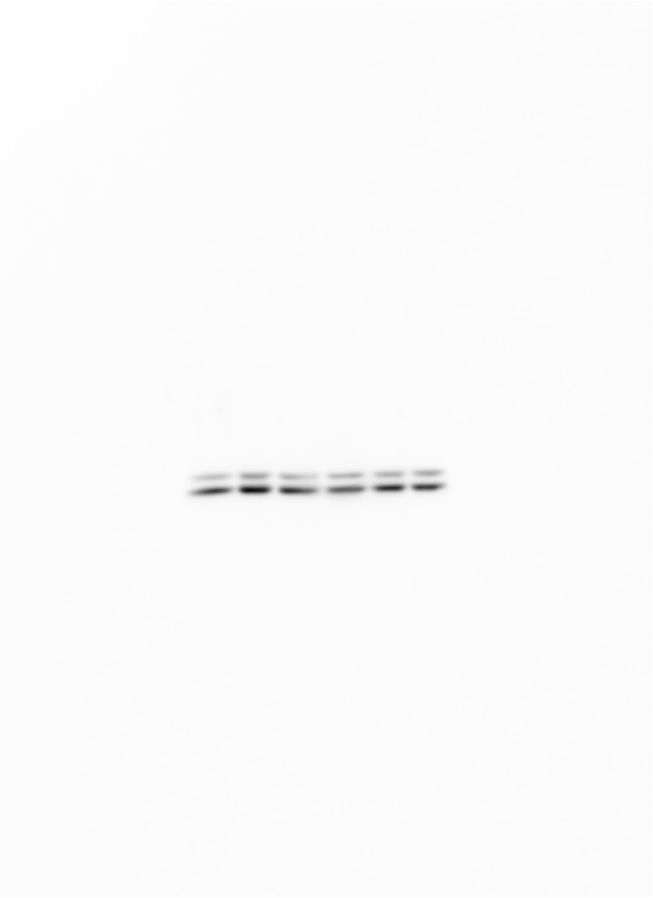

WB: GAPDH (reprobed)

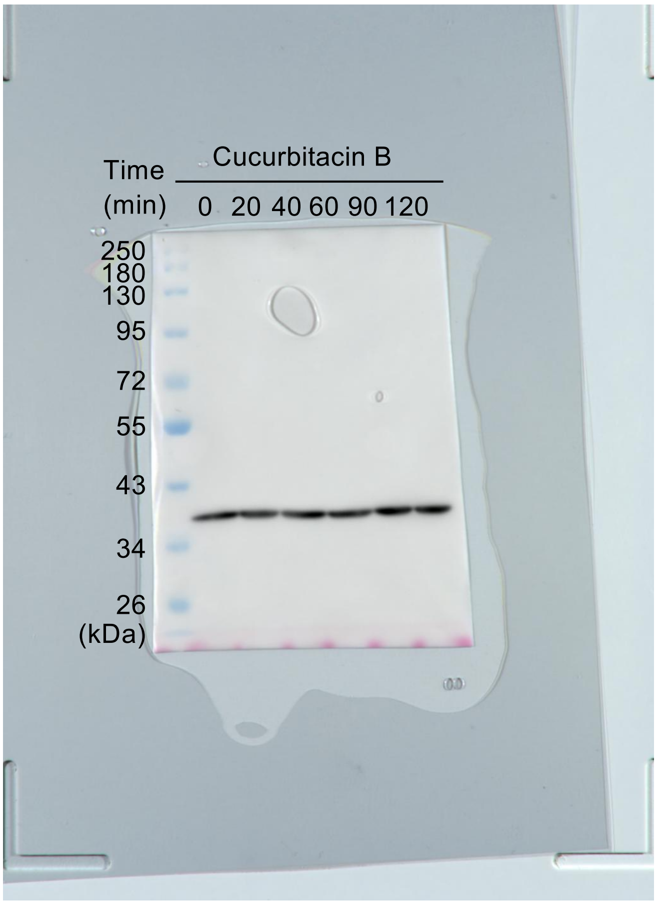


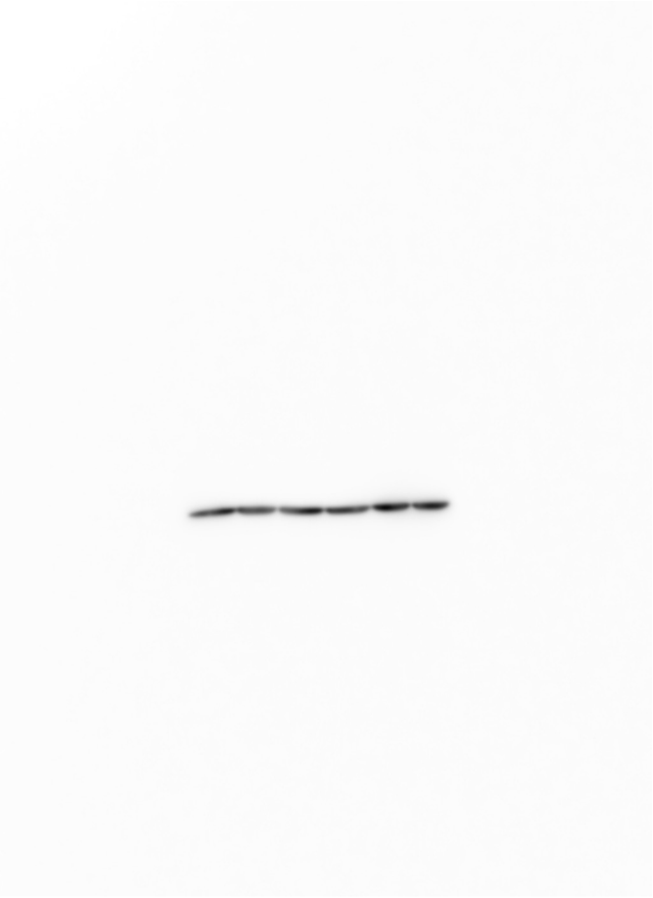

# Figure S55: Original blots (1) in Figures 9B and 9D

WB: Phospho-ERK

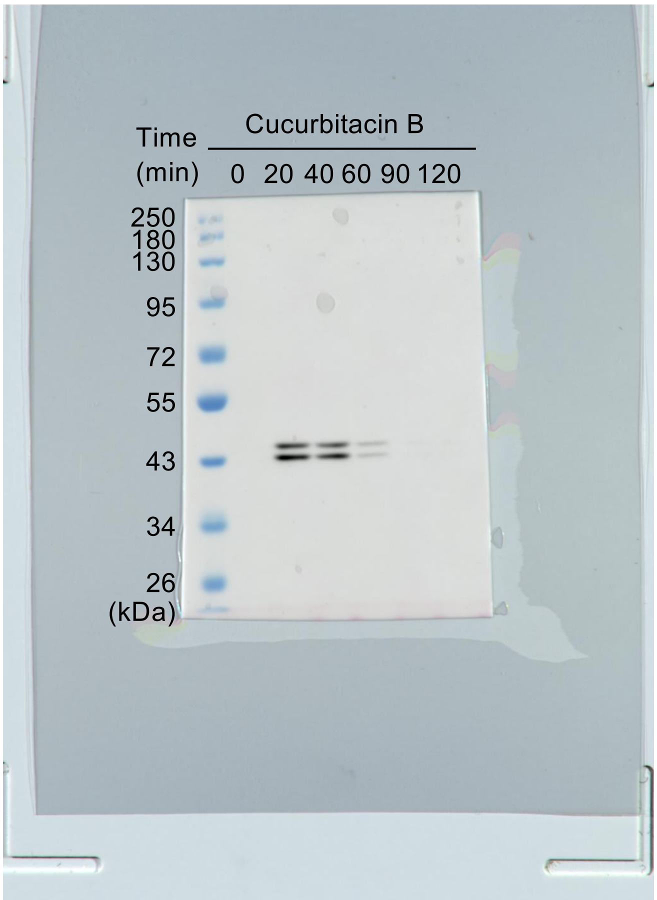


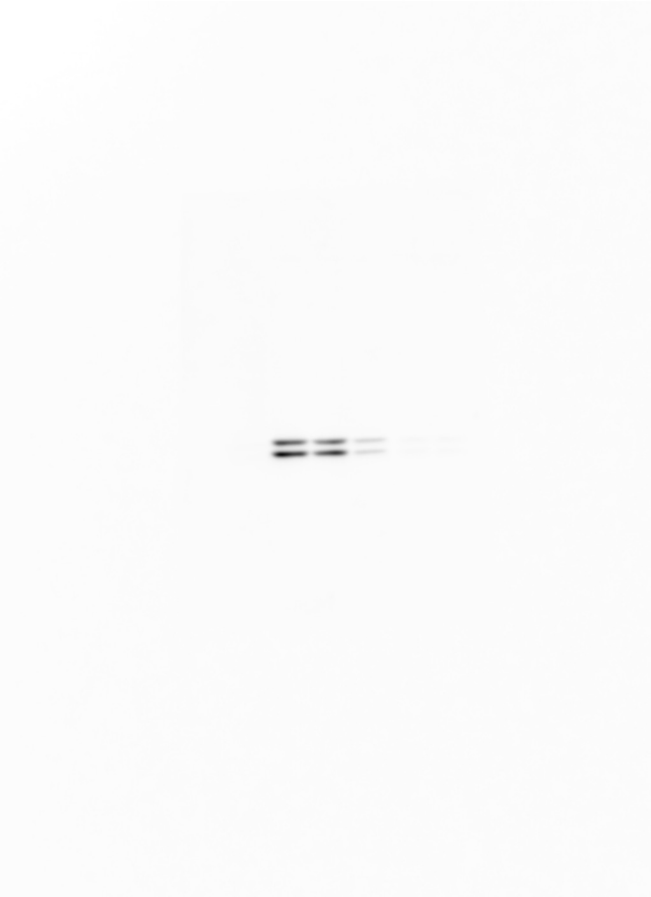

WB: GAPDH (reprobed)

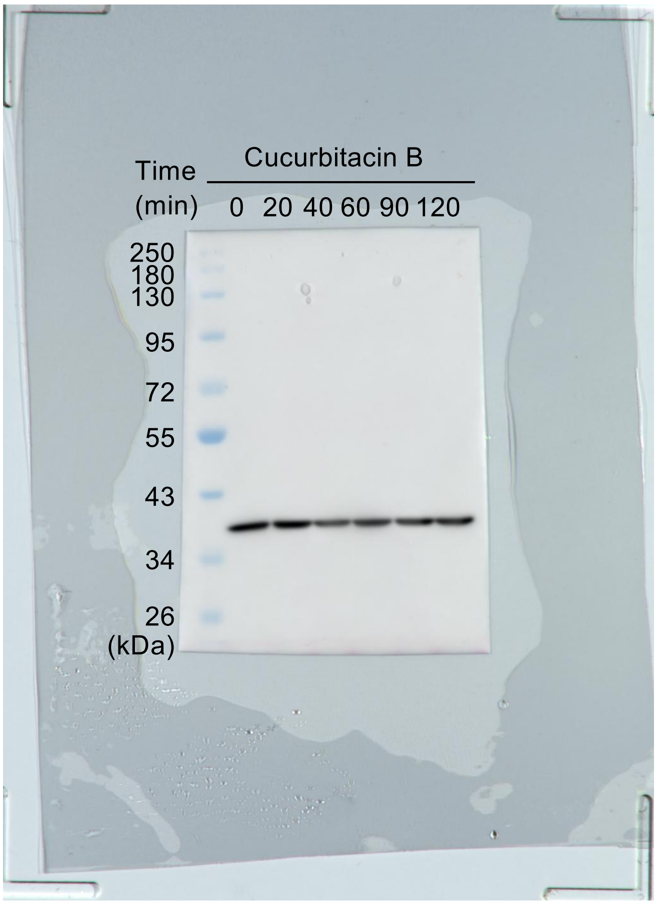


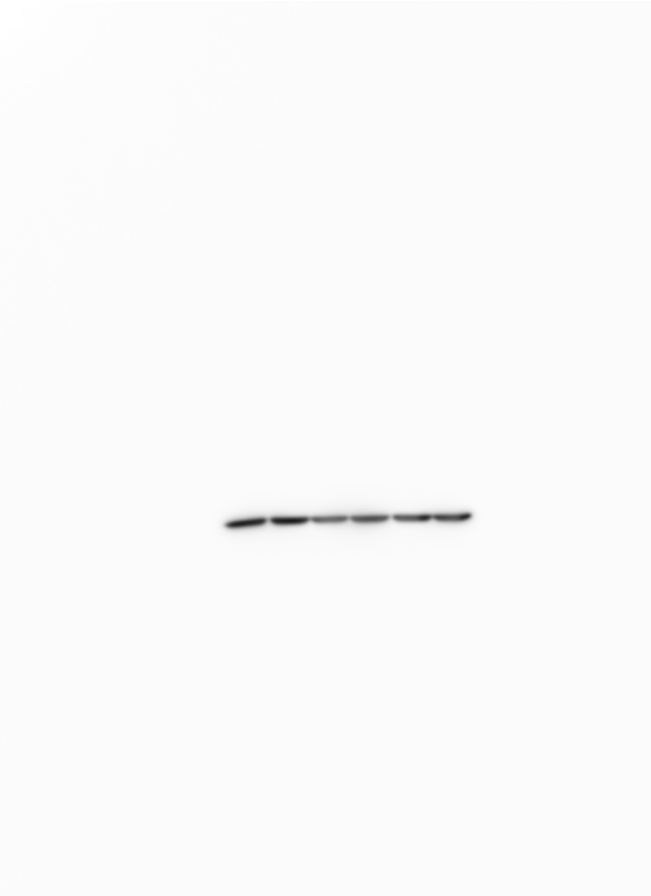

# Figure S56: Original blots (2) in Figures 9B and 9D

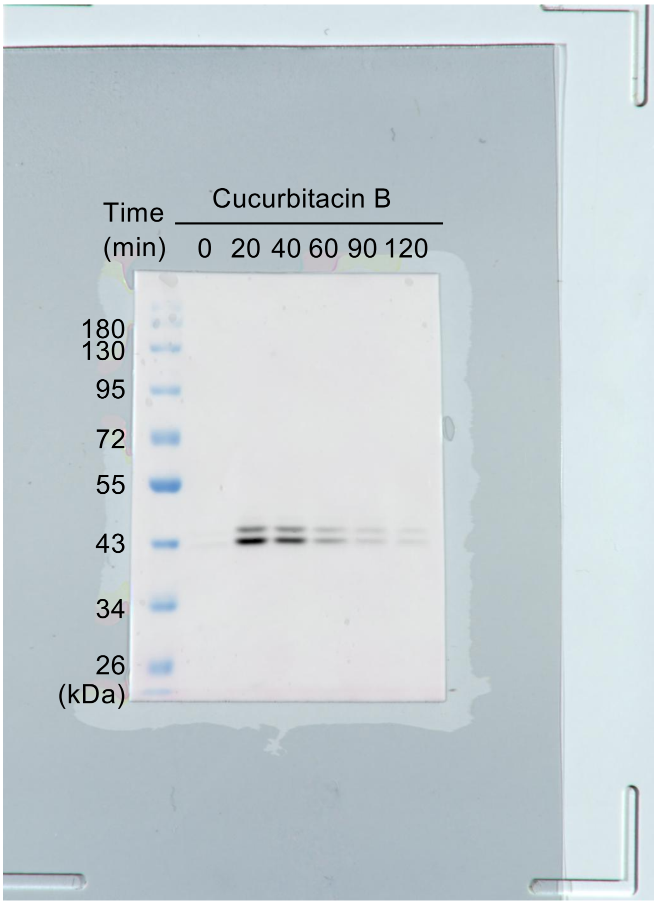


WB: Phospho-ERK

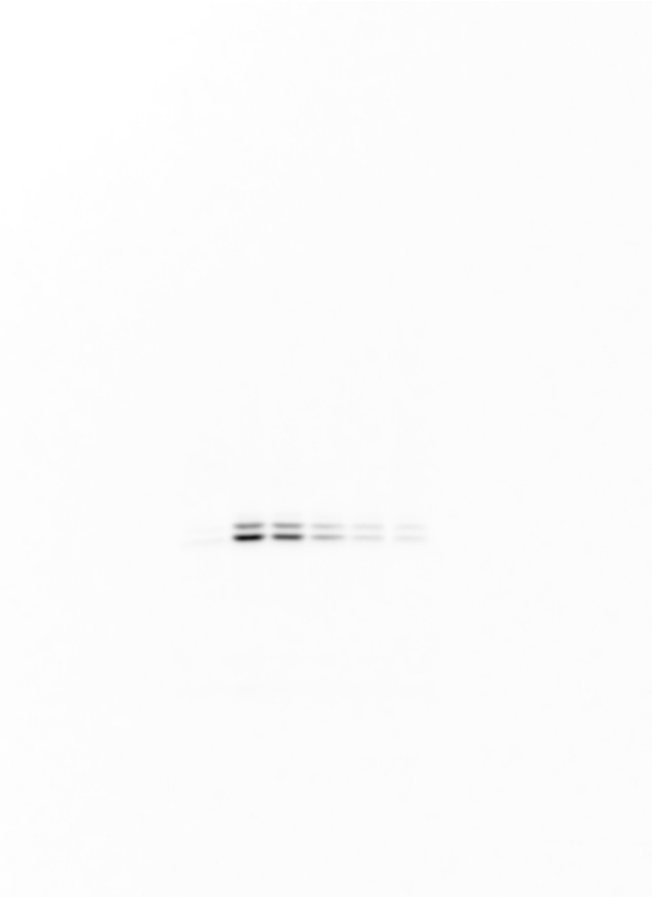

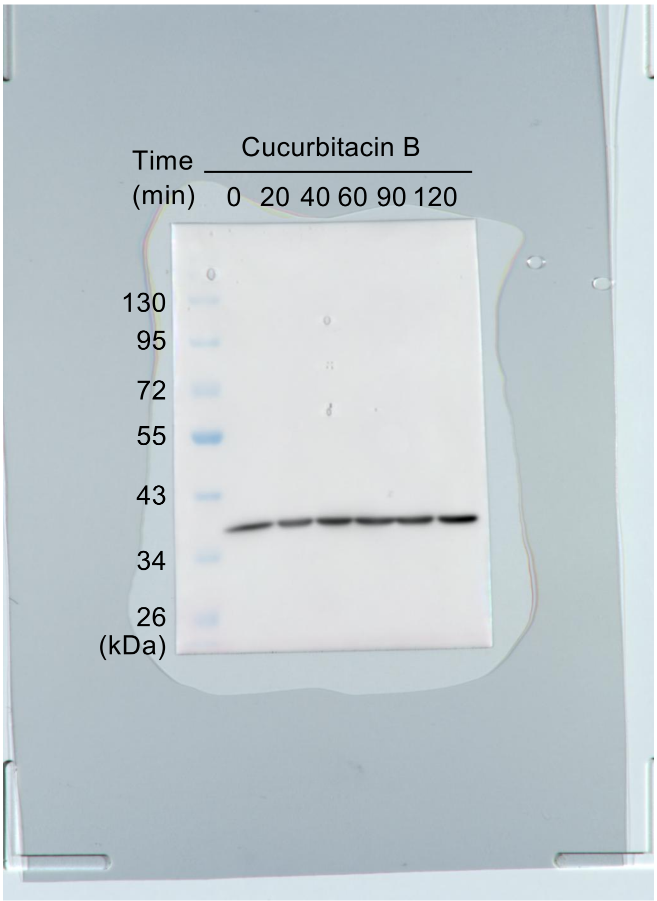


WB: GAPDH (reprobed)

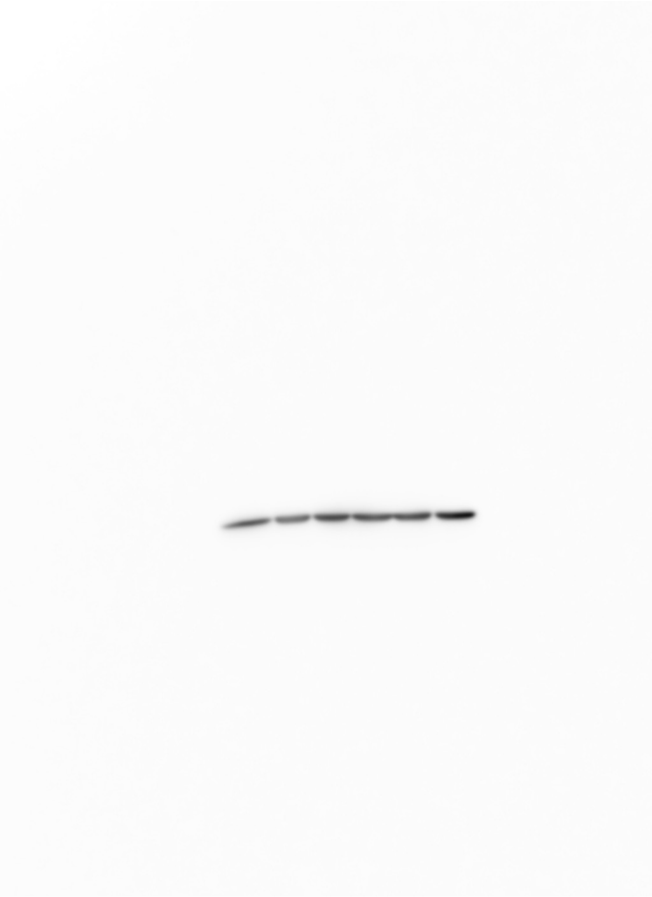

# Figure S57: Original blots (3) in Figures 9B and 9D

WB: Phospho-ERK

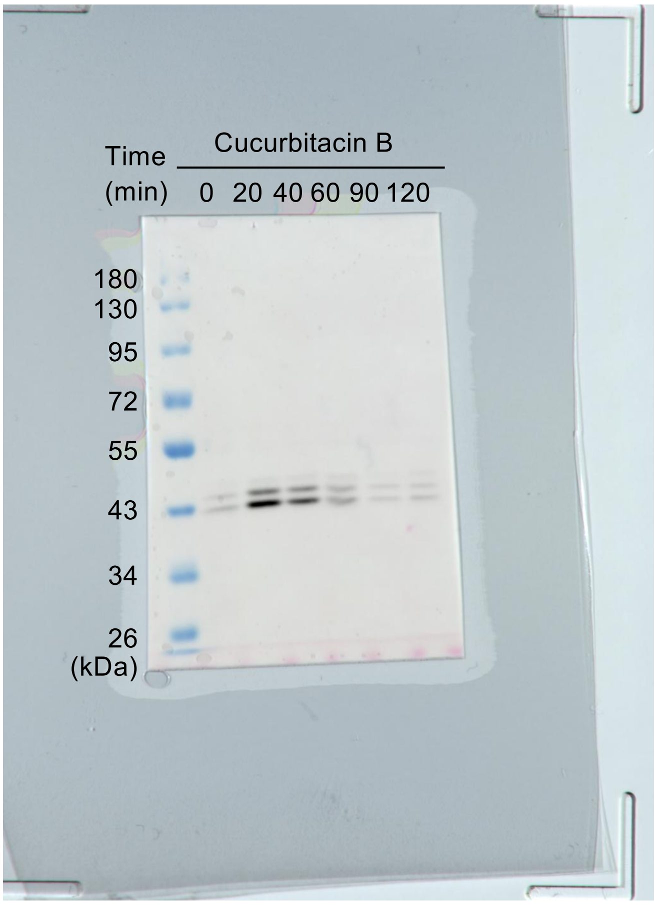


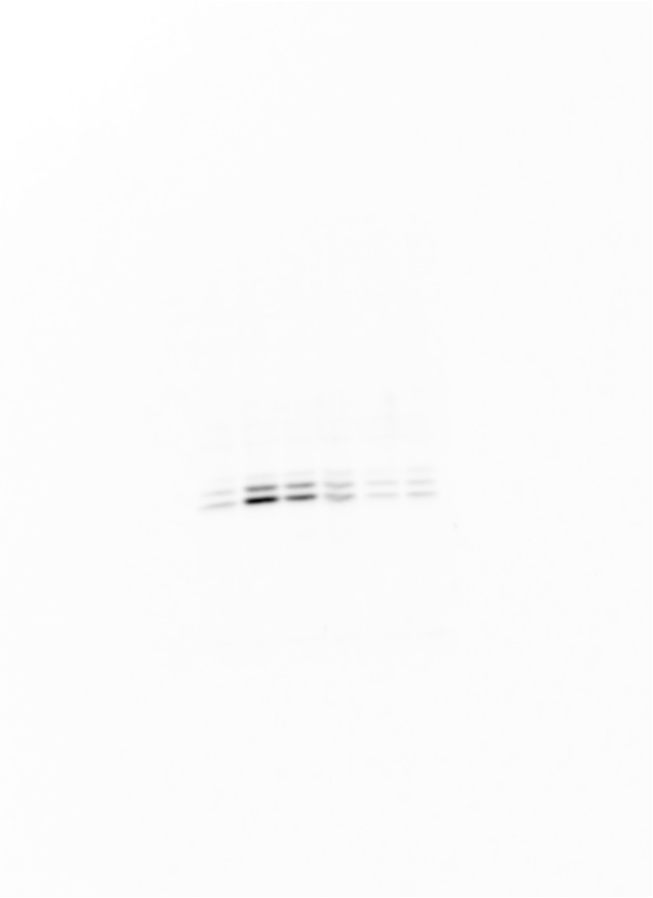

WB: GAPDH (reprobed)

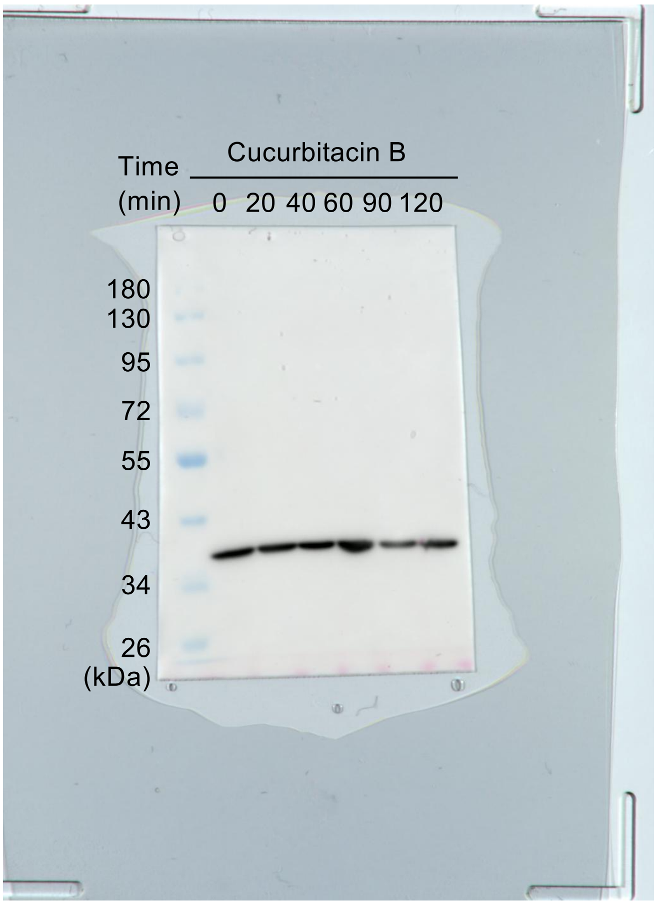


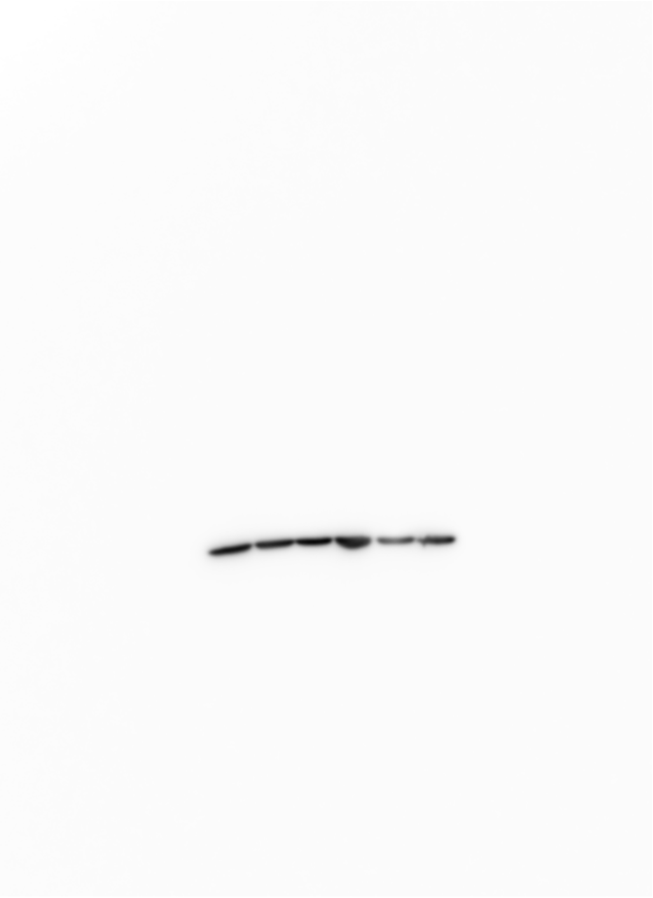

## Figure S58: Original blots (4) in Figures 9B and 9D

WB: Phospho-ERK

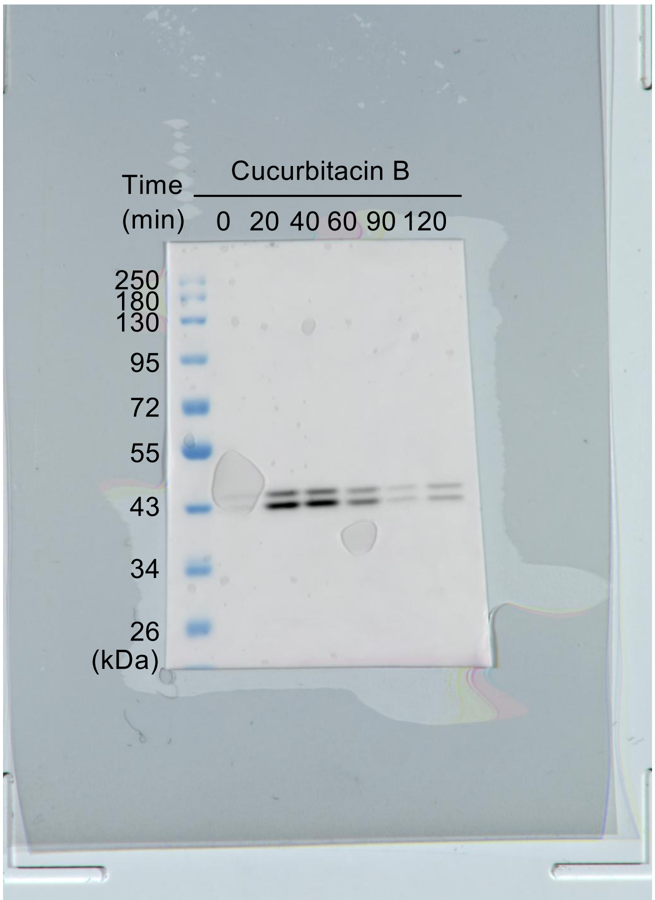


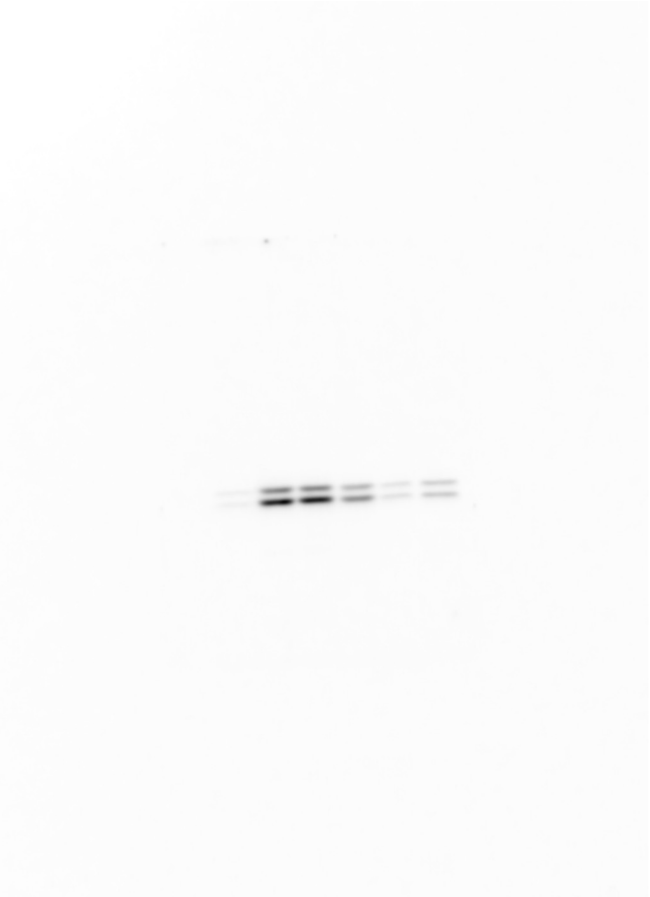

WB: GAPDH (reprobed)

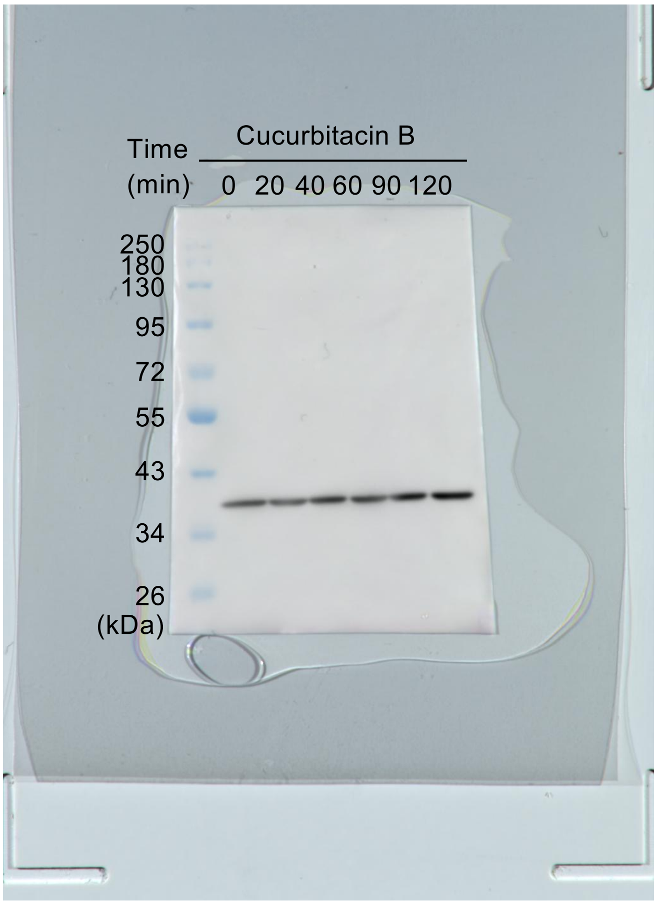


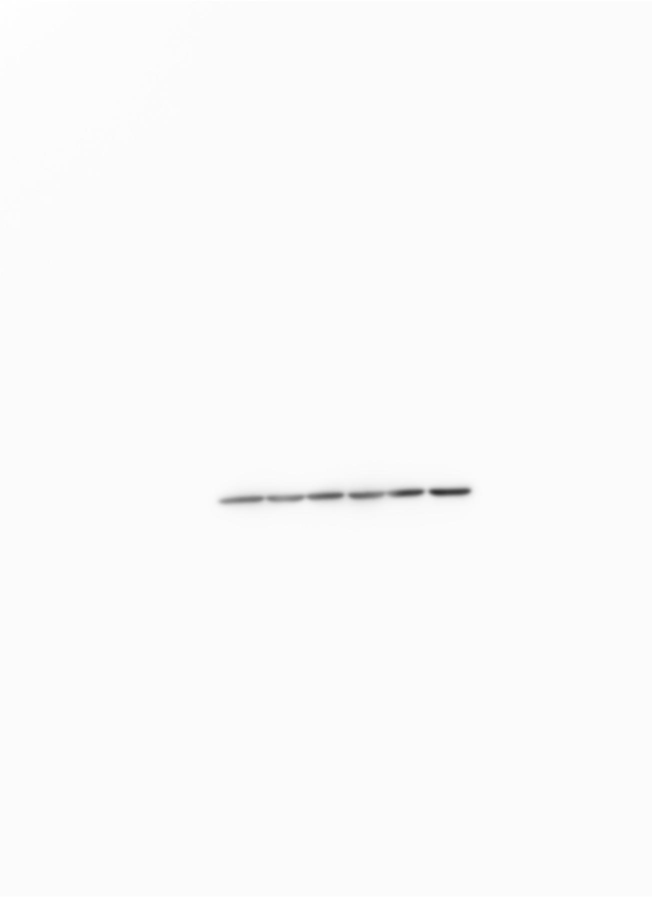

# Figure S59: Original blots (1) in Figures 9C and 9D

WB: ERK

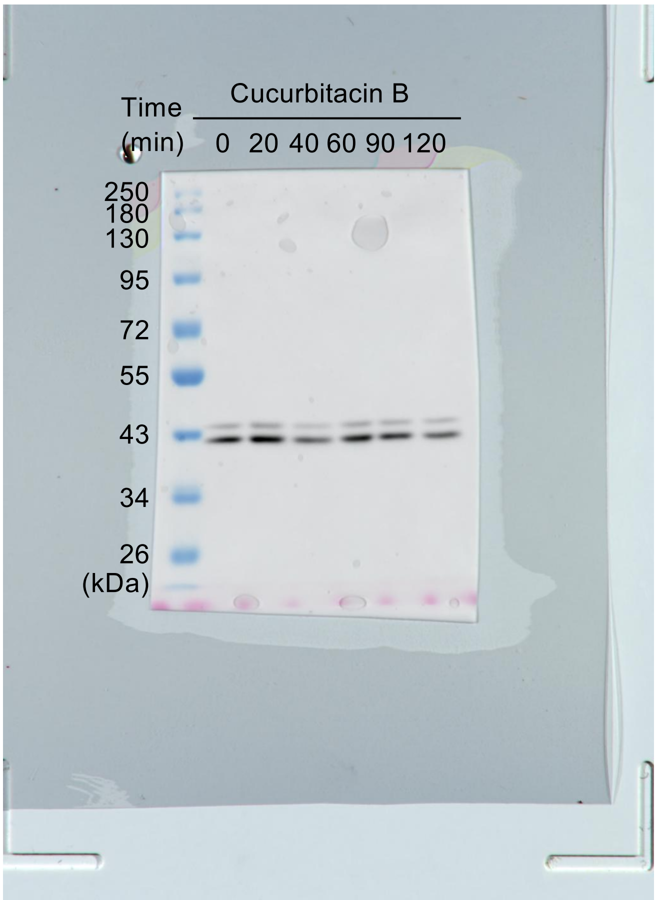


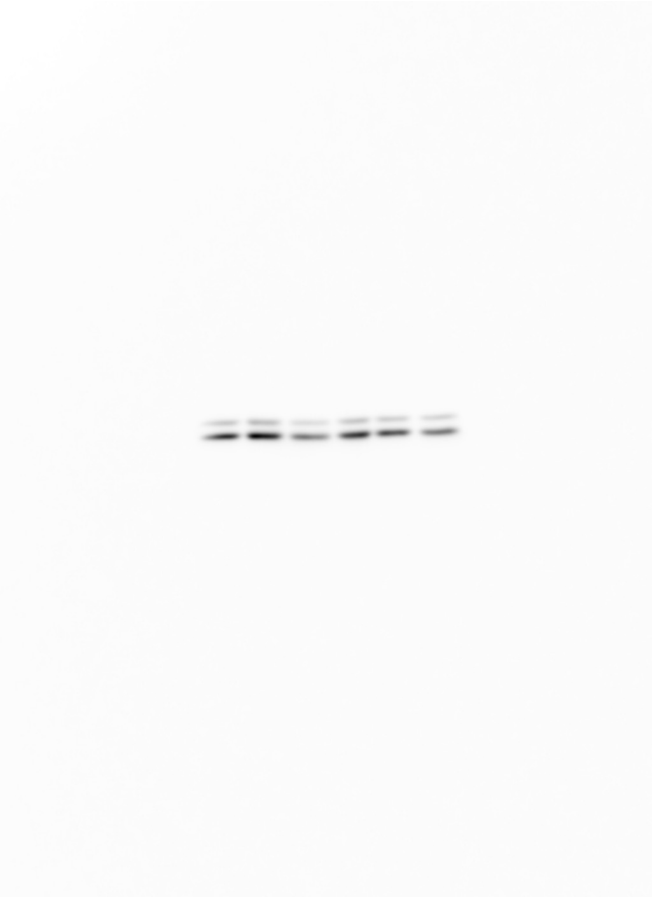

WB: GAPDH (reprobed)

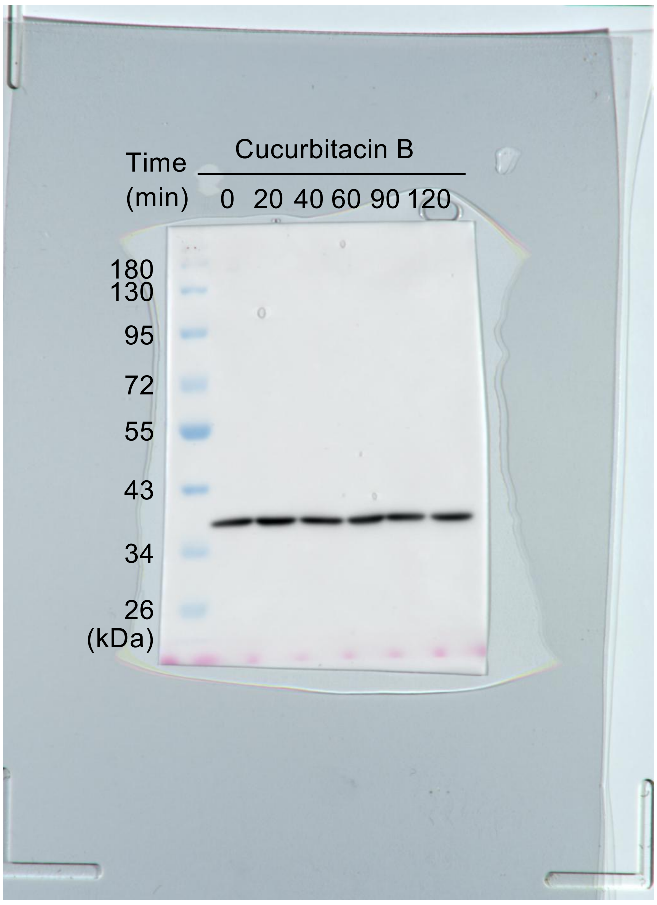


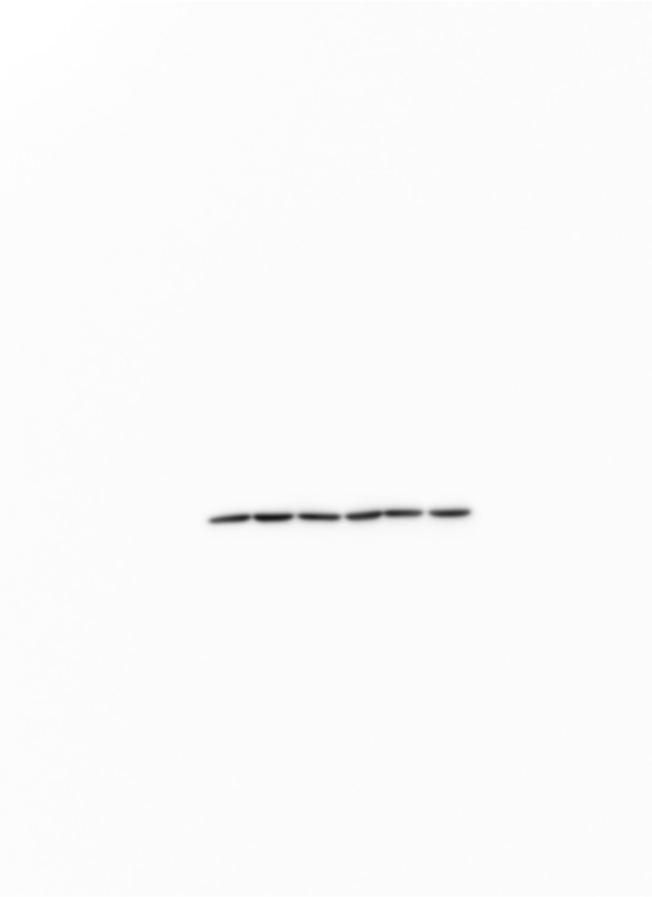

# Figure S60: Original blots (2) in Figures 9C and 9D

WB: ERK

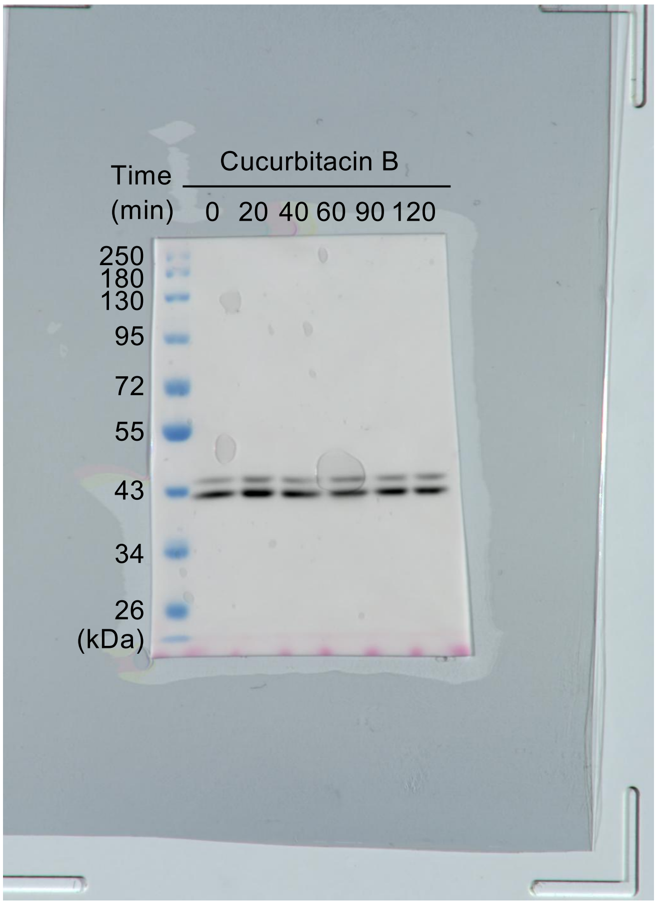


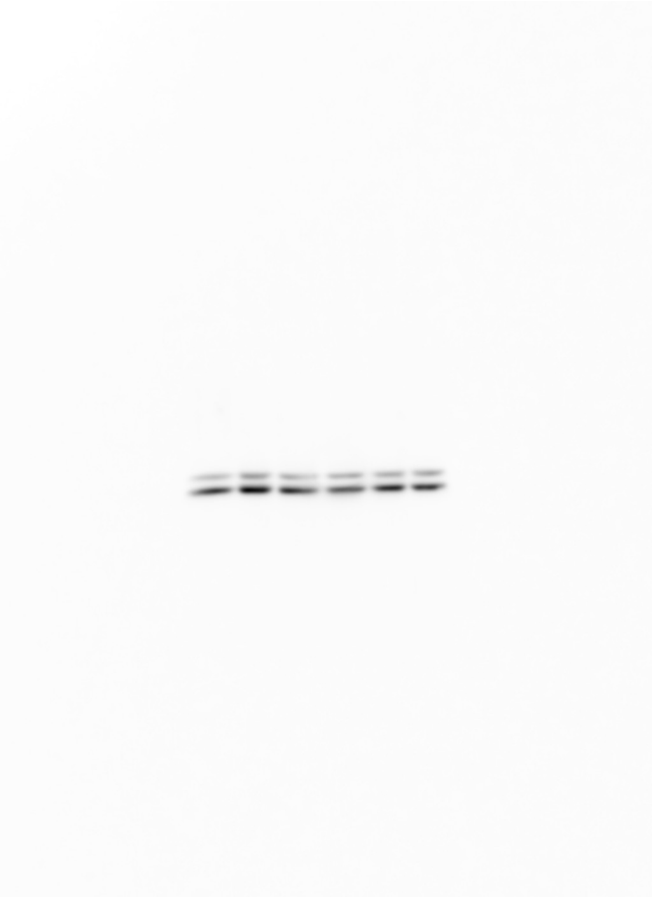

WB: GAPDH (reprobed)

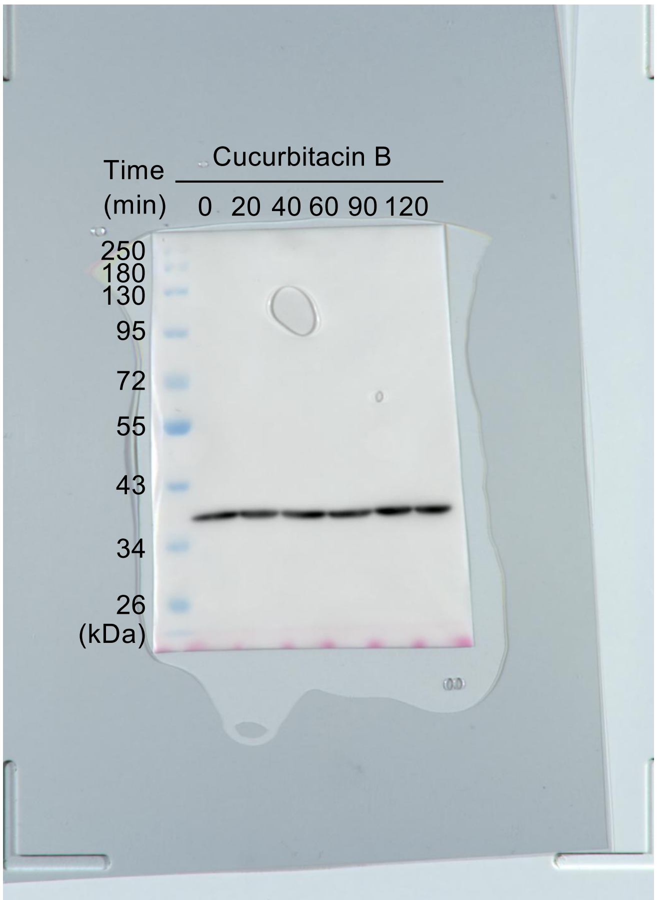


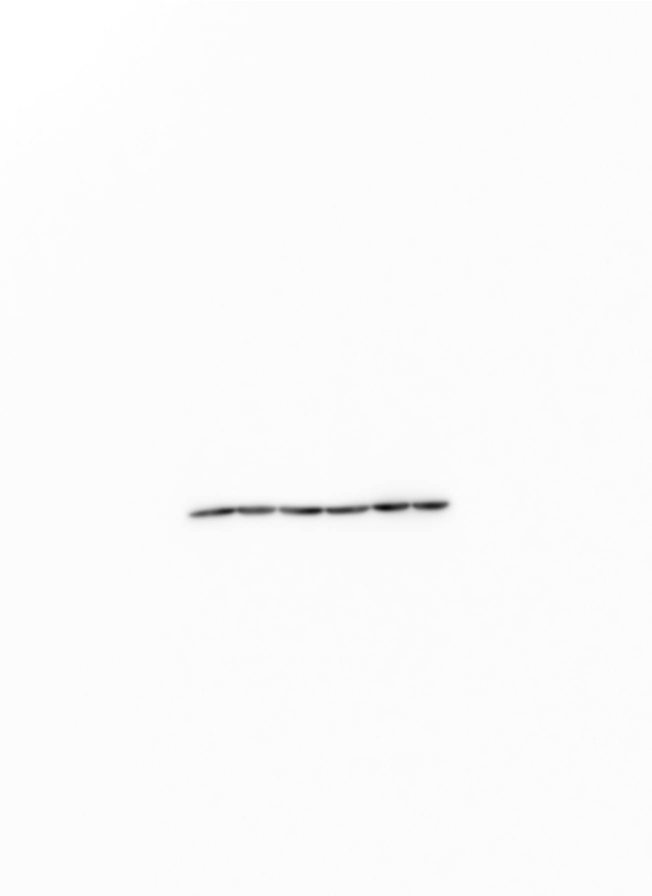

## Figure S61: Original blots (3) in Figures 9C and 9D

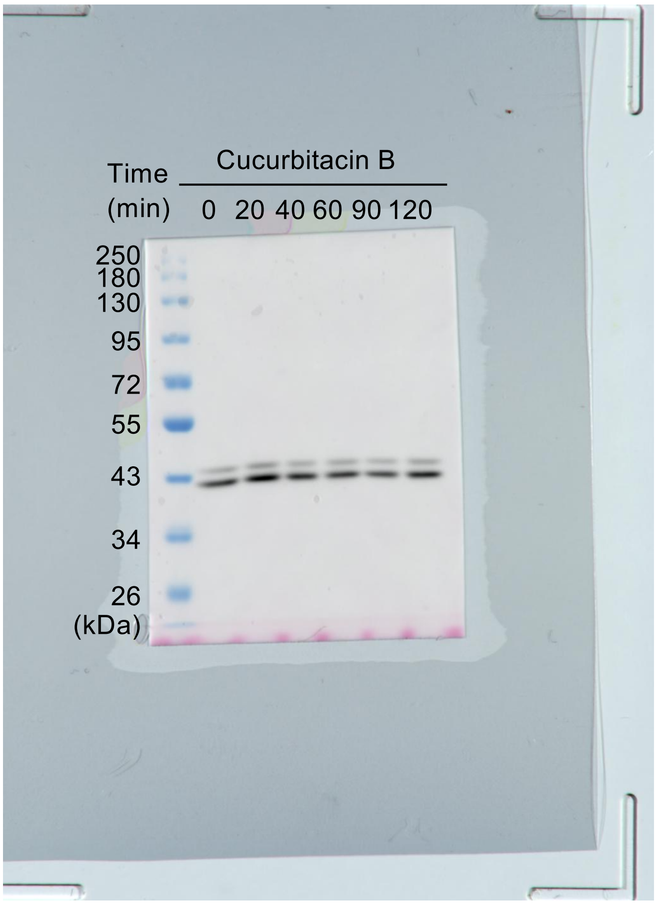


WB: ERK

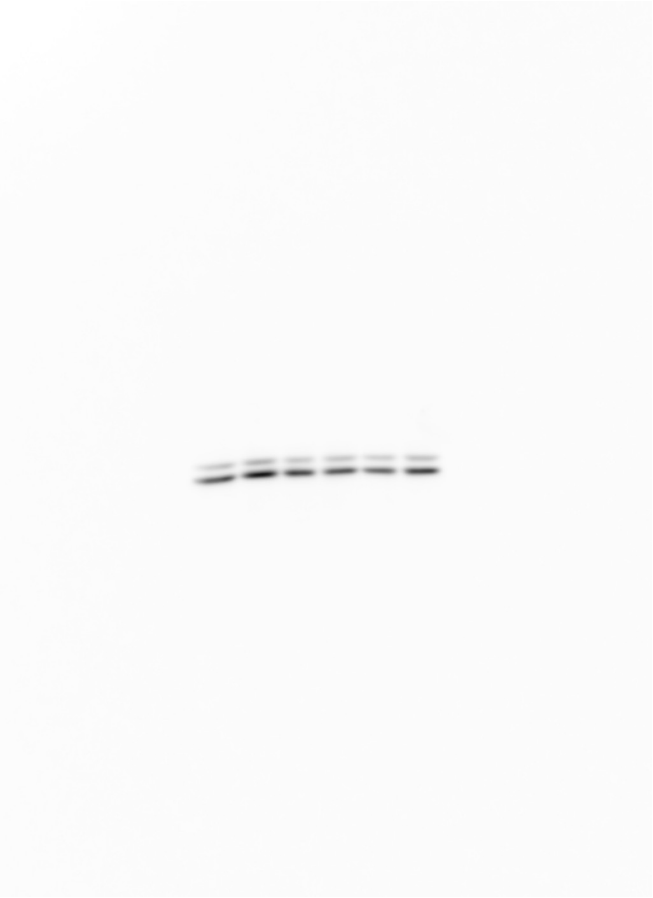

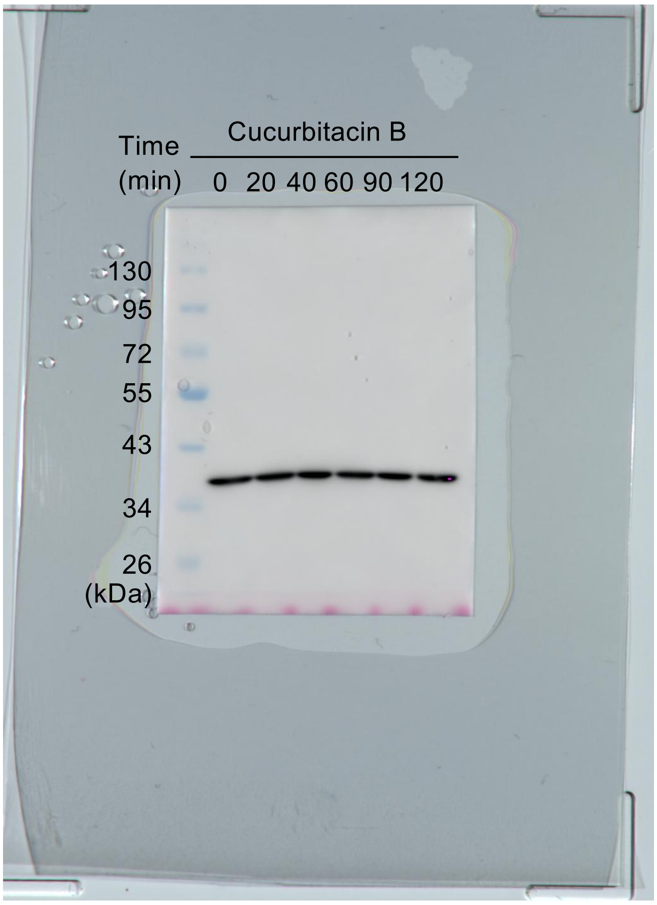


WB: GAPDH (reprobed)

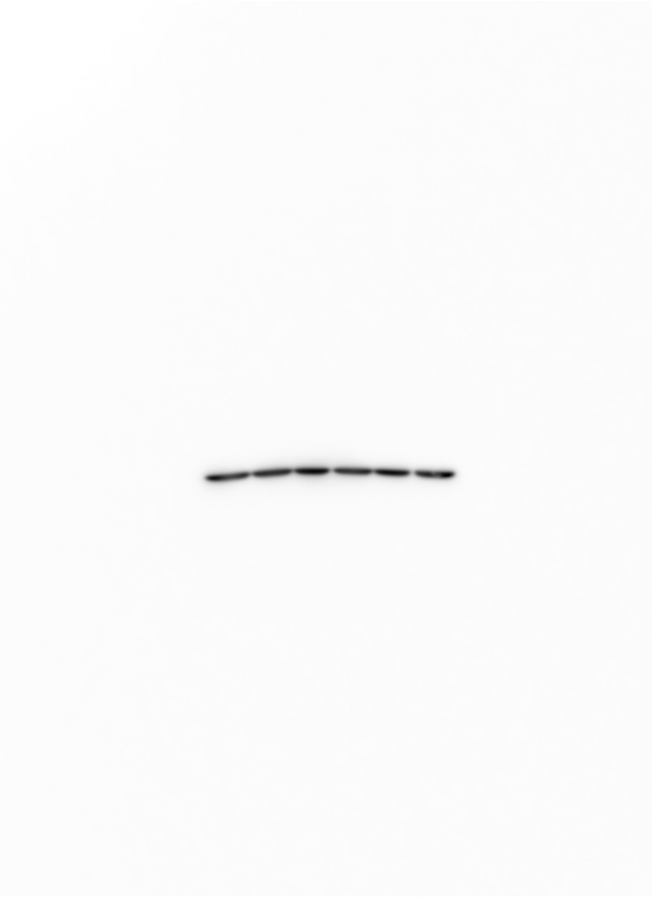

# Figure S62: Original blots (4) in Figures 9C and 9D

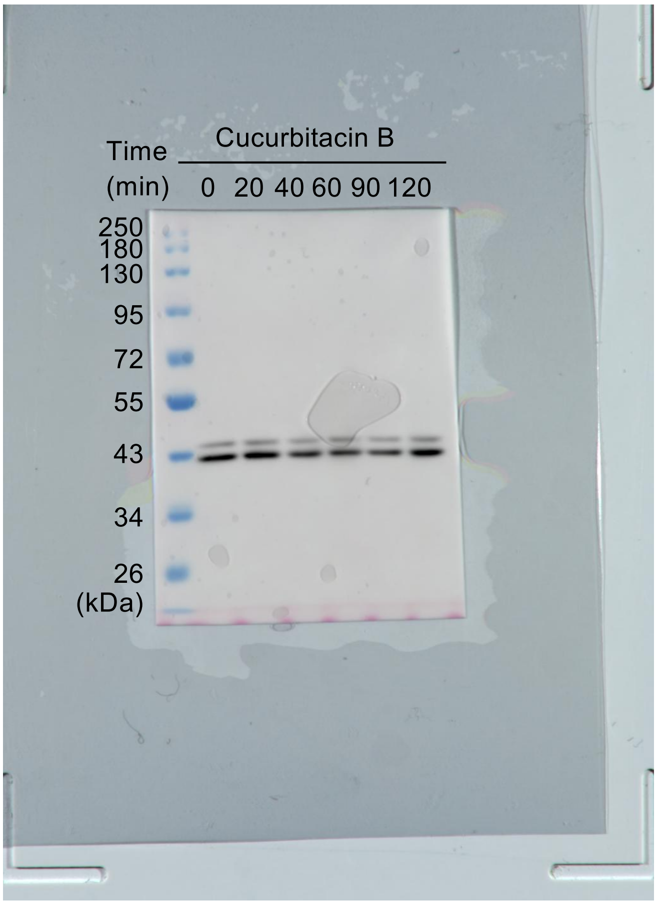


WB: ERK

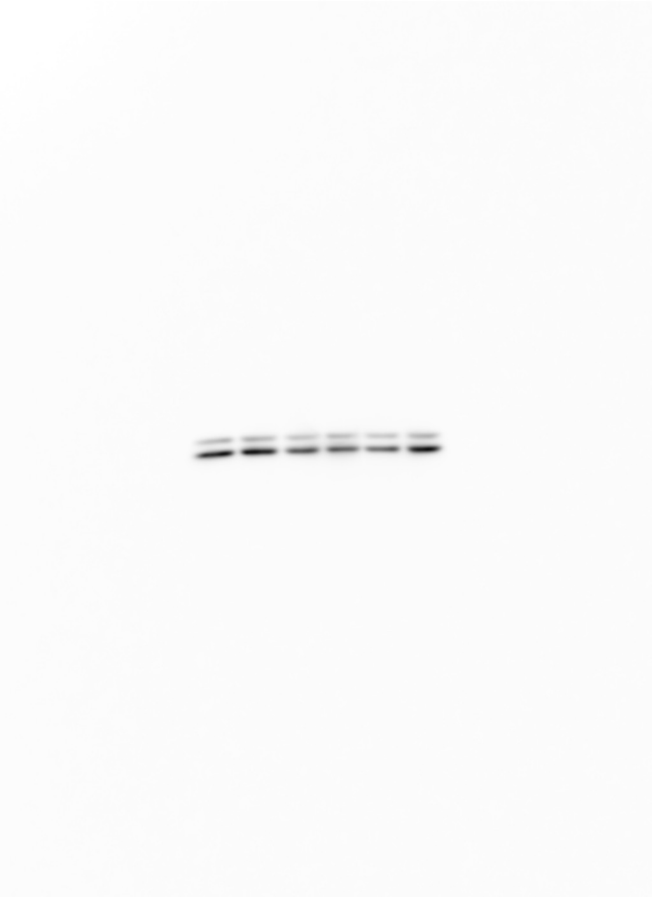

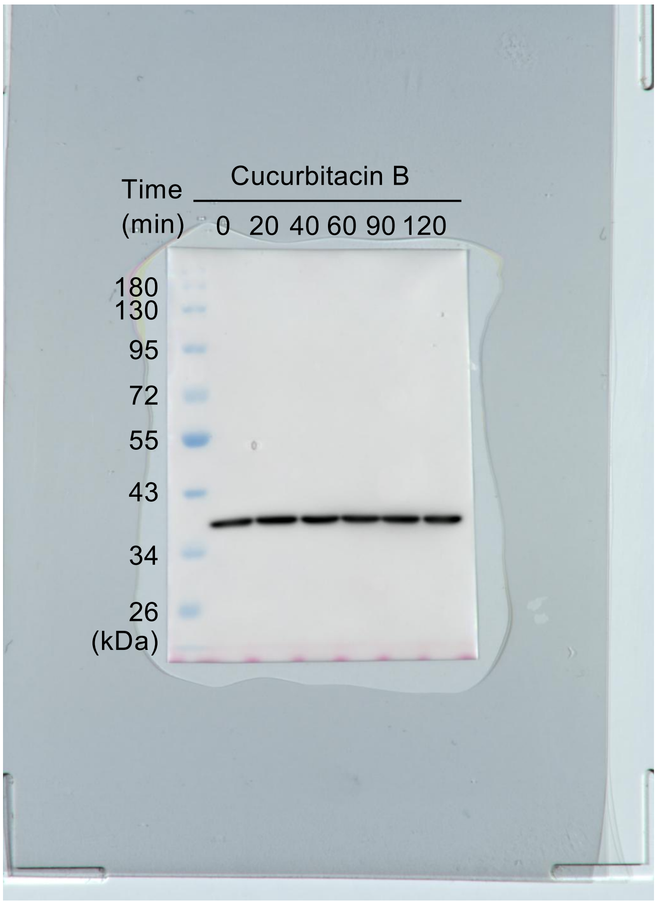


WB: GAPDH (reprobed)

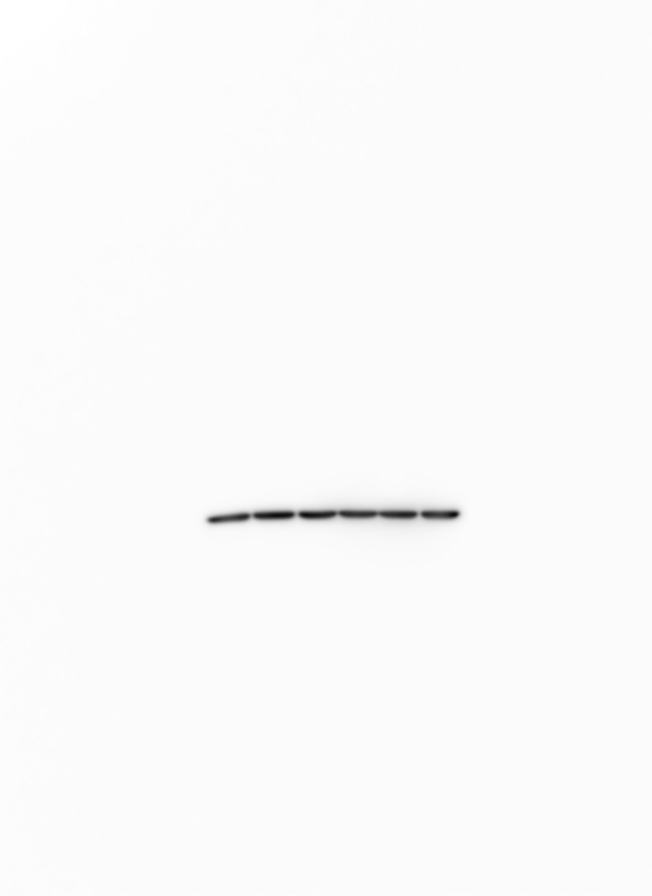

# Figure S63: Original blots in Figure 9E (phospho-p38 MAPK)

WB: Phospho-p38 MAPK

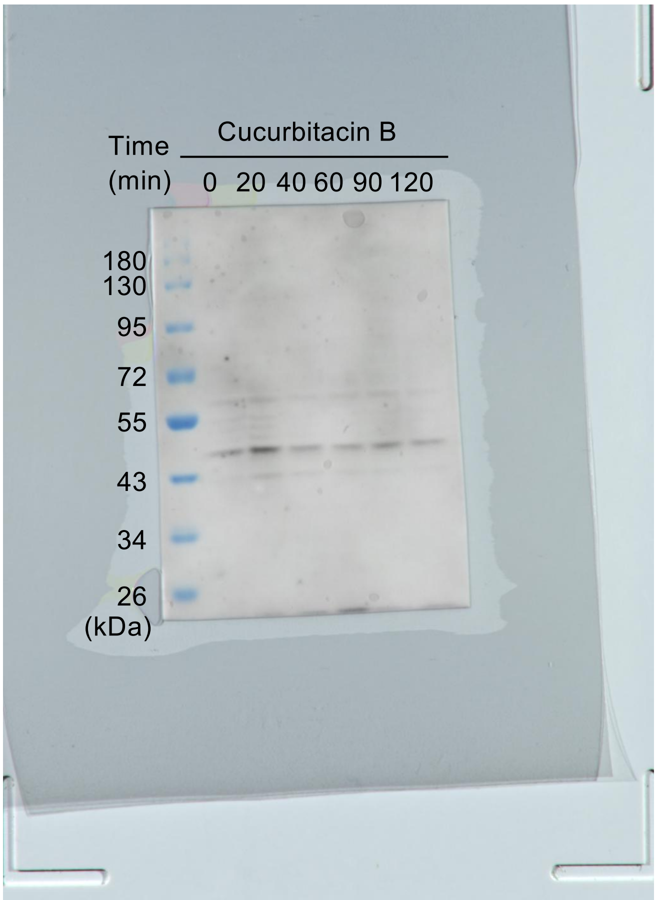


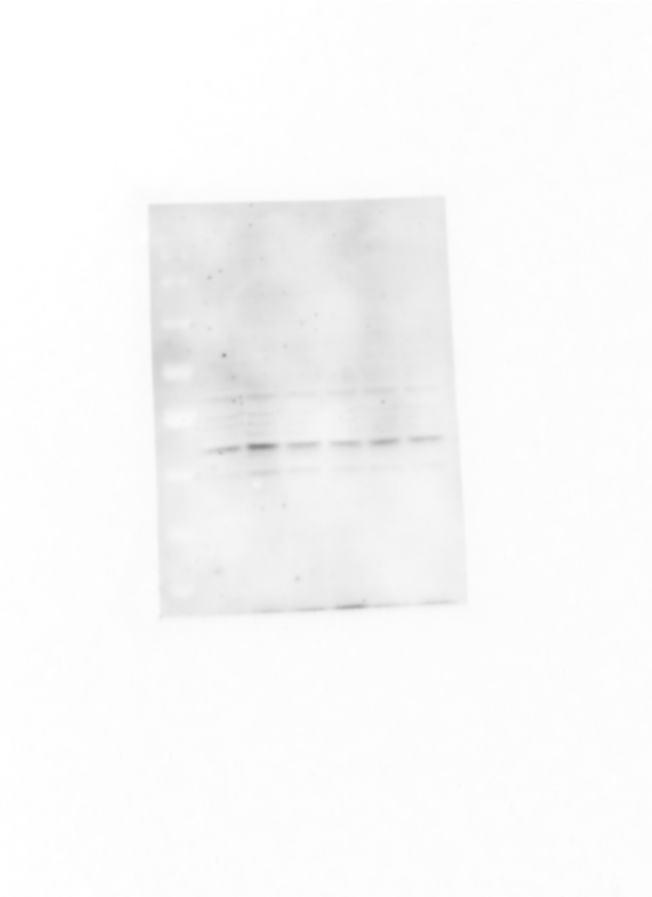

WB: GAPDH (reprobed)

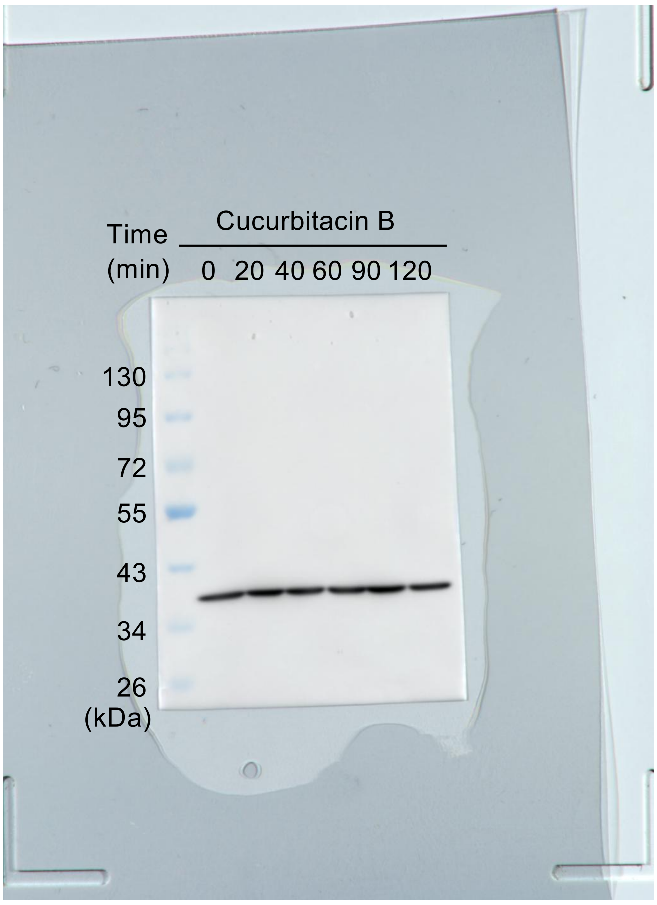


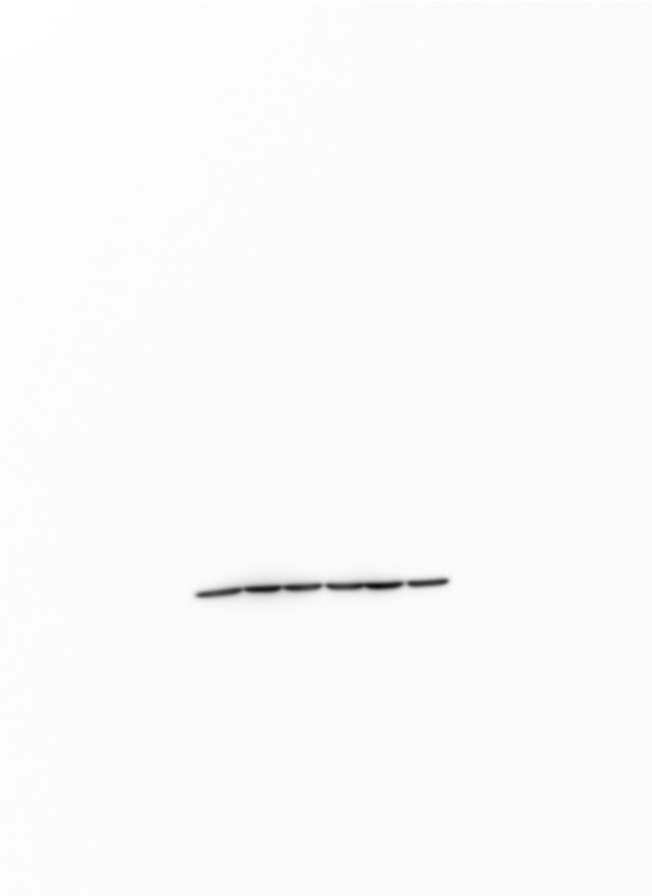

# Figure S64: Original blots in Figure 9E (p38 MAPK)

WB: p38 MAPK

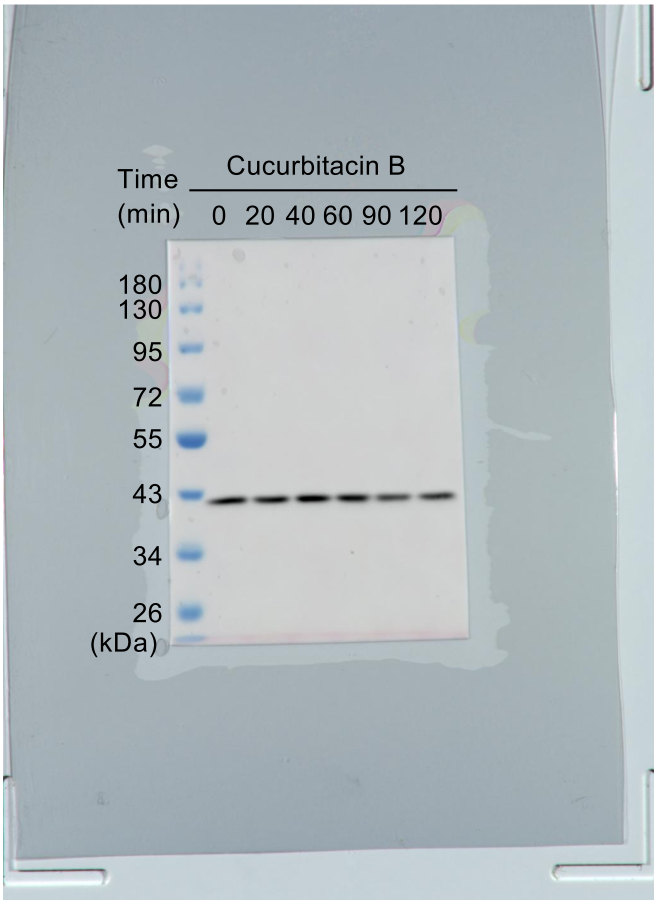


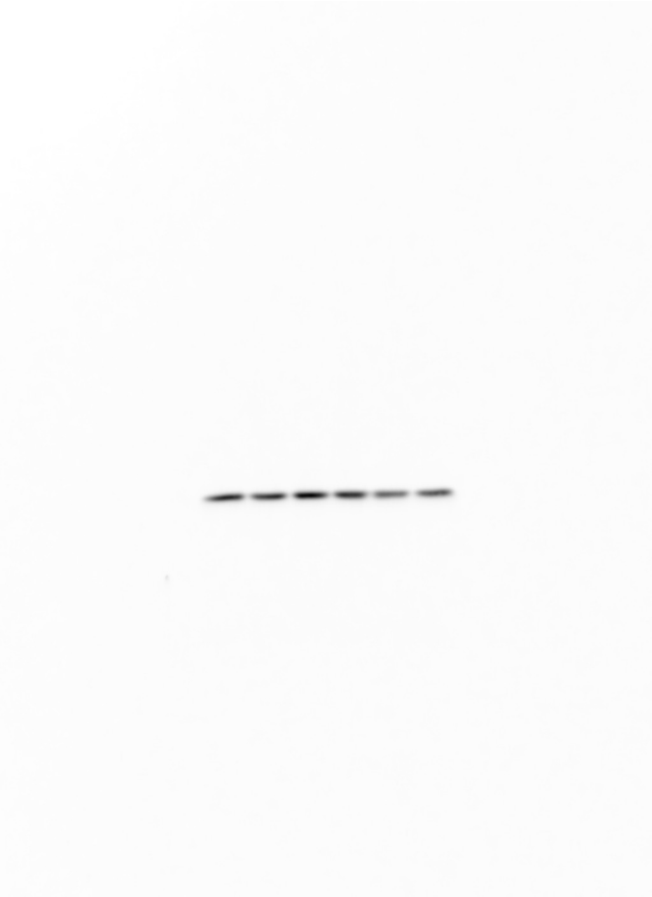

WB: GAPDH (reprobed)

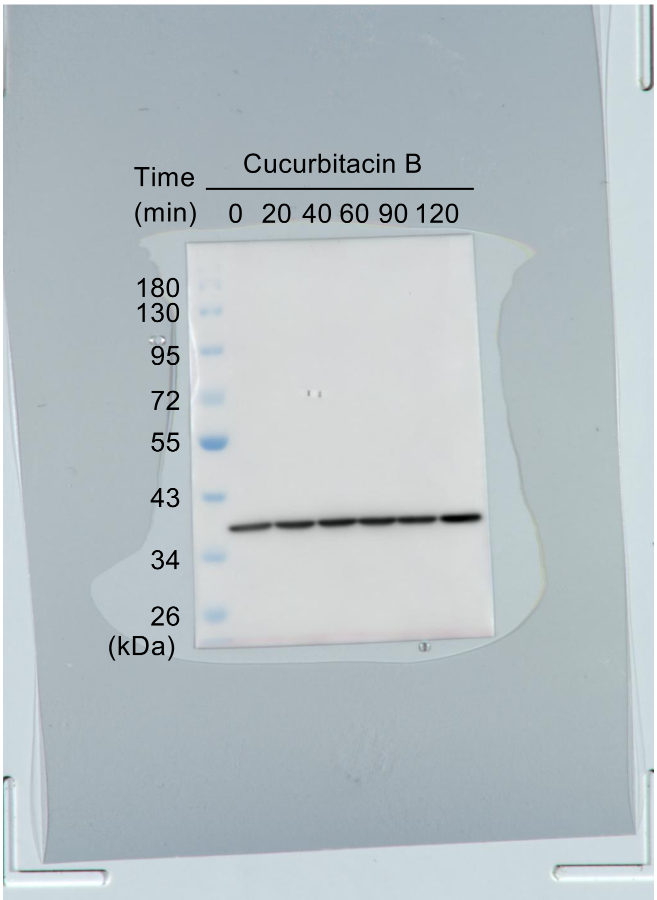


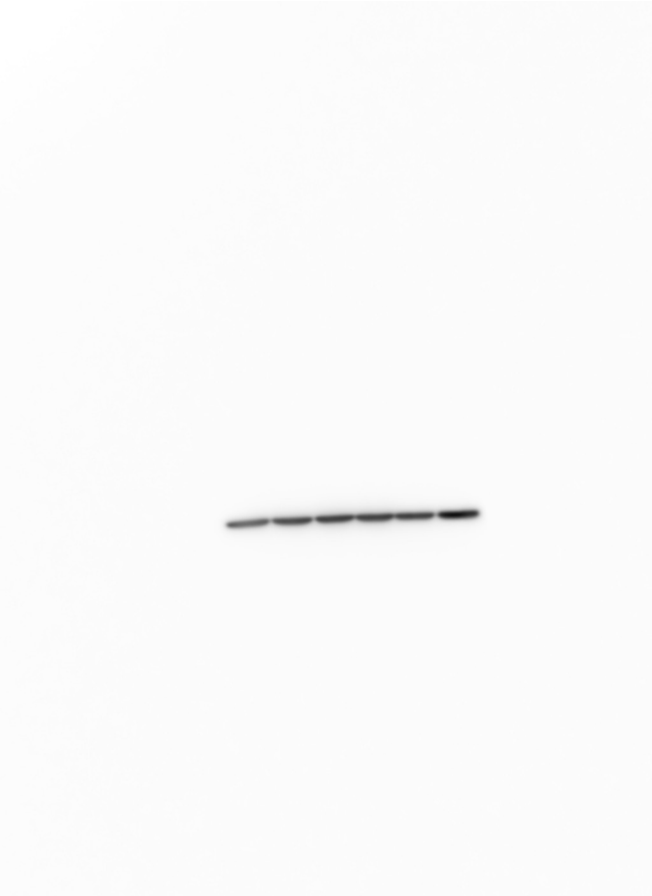

# Figure S65: Original blots (1) in Figures 9F and 9H

WB: Phospho-p38 MAPK

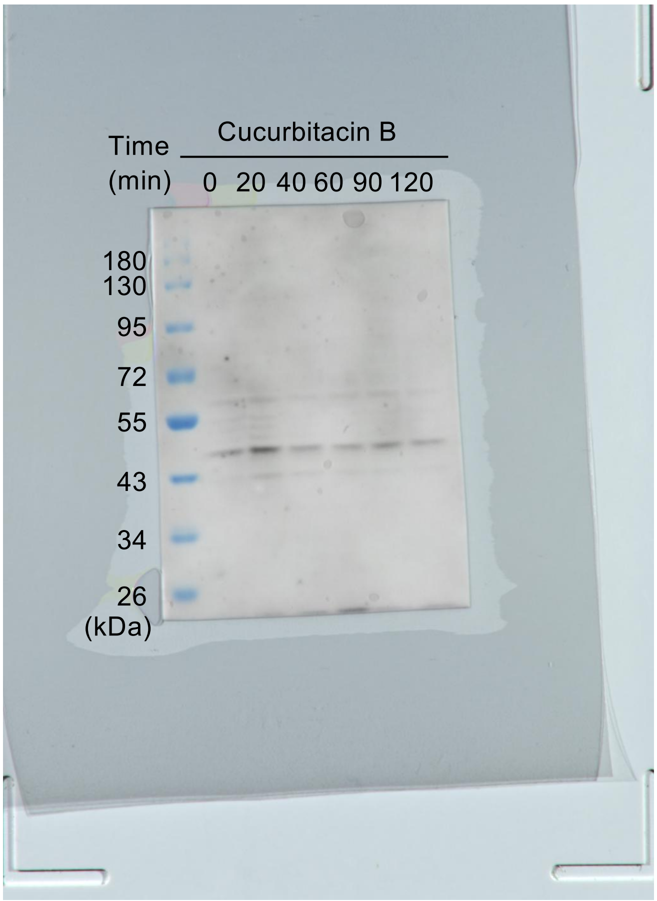


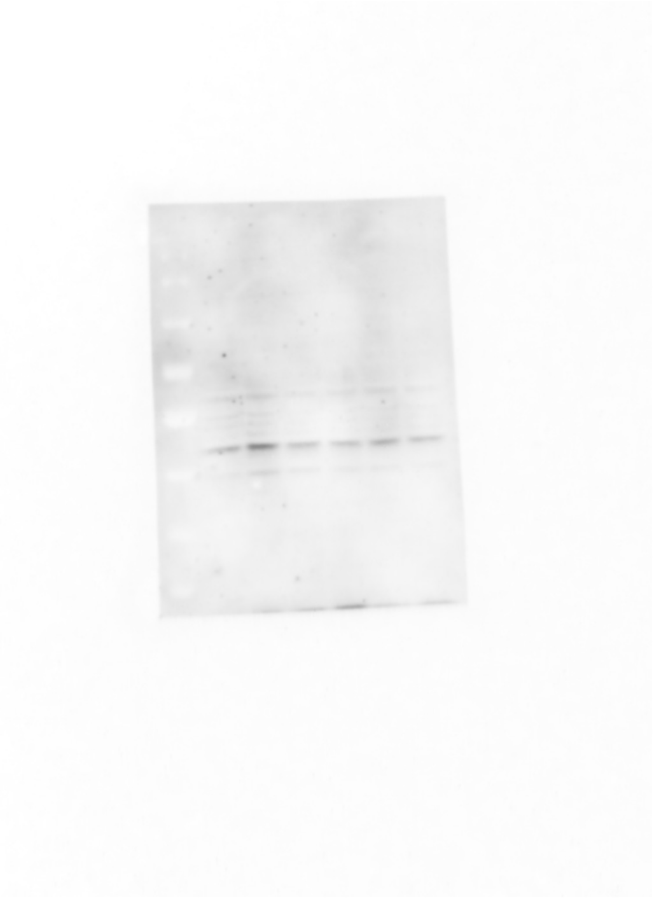

WB: GAPDH (reprobed)

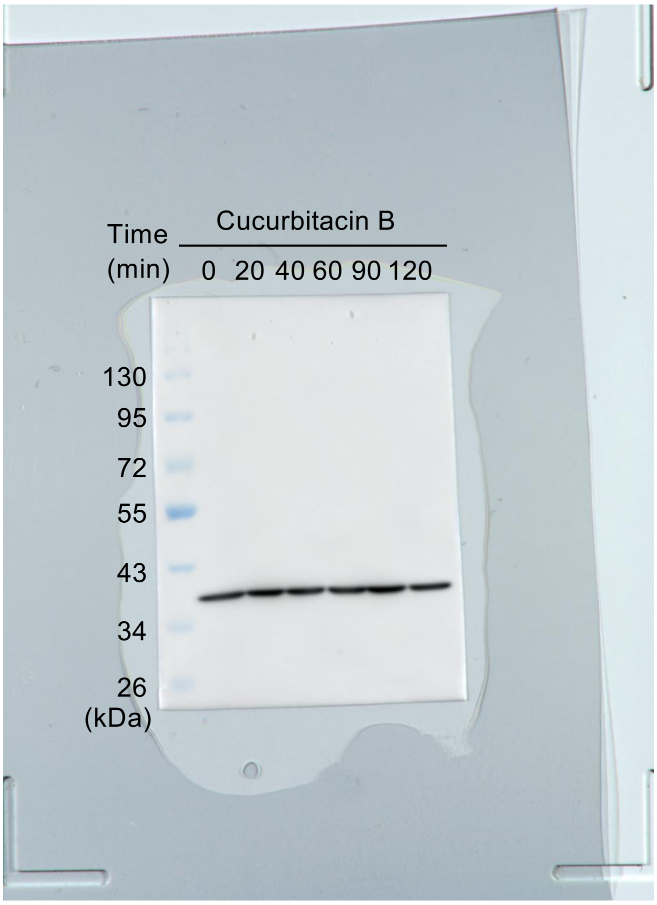


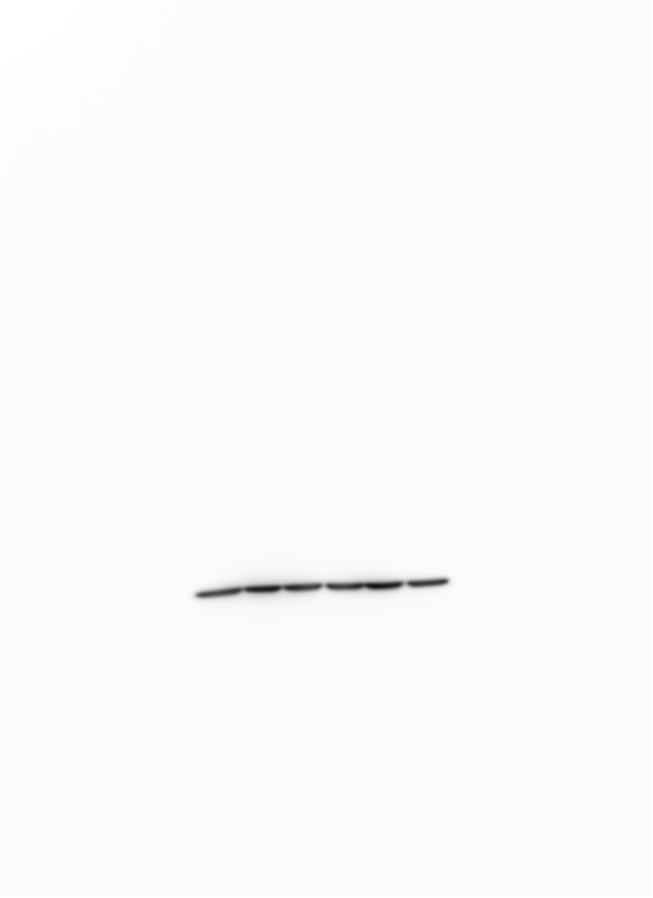

# Figure S66: Original blots (2) in Figures 9F and 9H

WB: Phospho-p38 MAPK

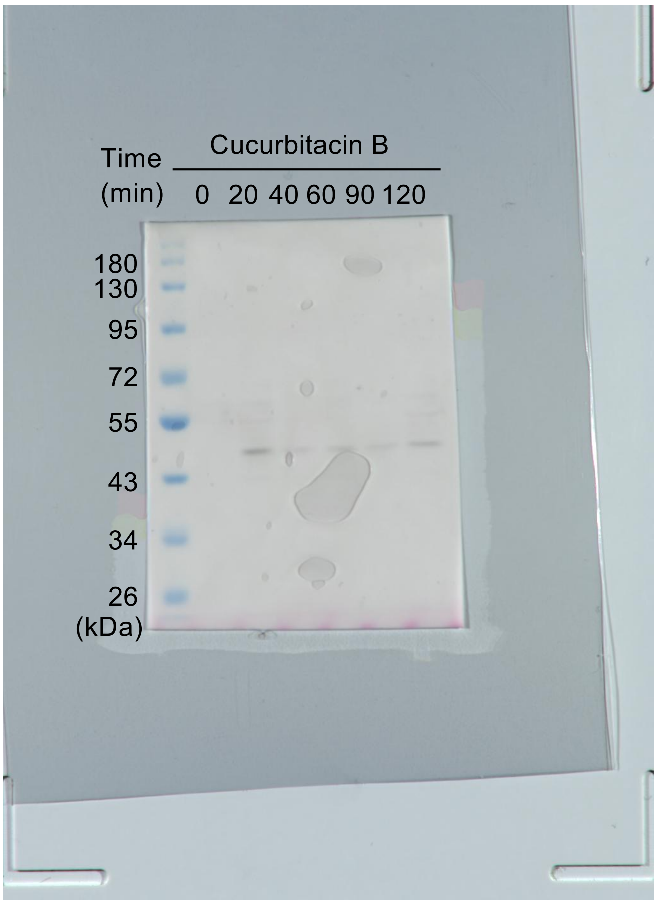




WB: GAPDH (reprobed)

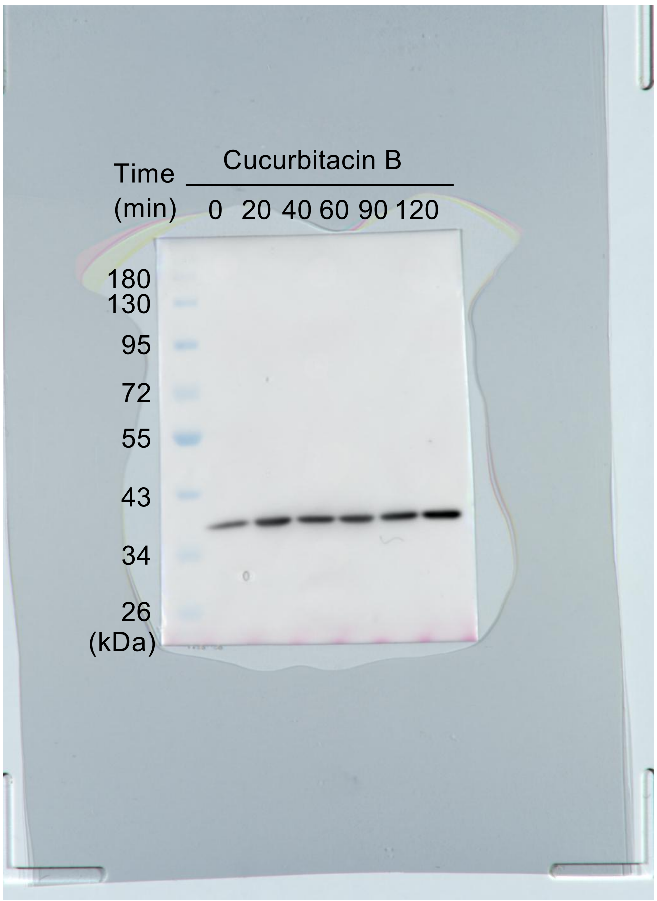


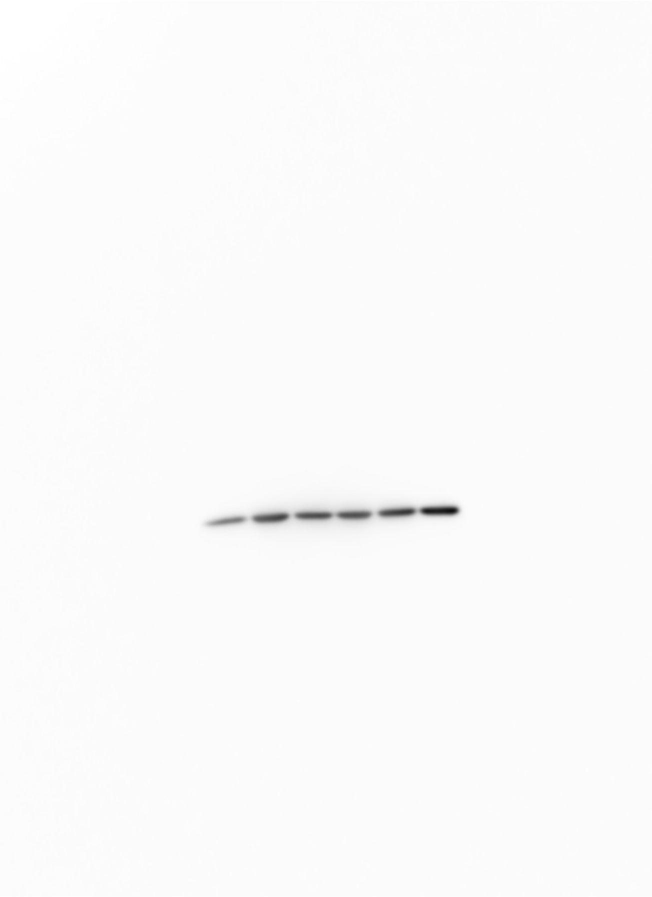

## Figure S67: Original blots (3) in Figures 9F and 9H

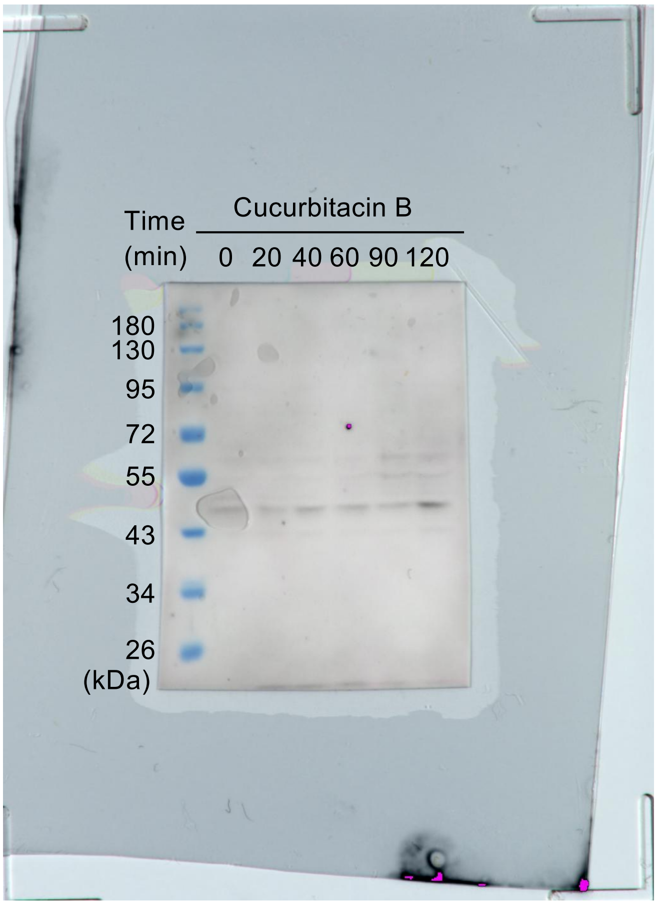


WB: Phospho-p38 MAPK

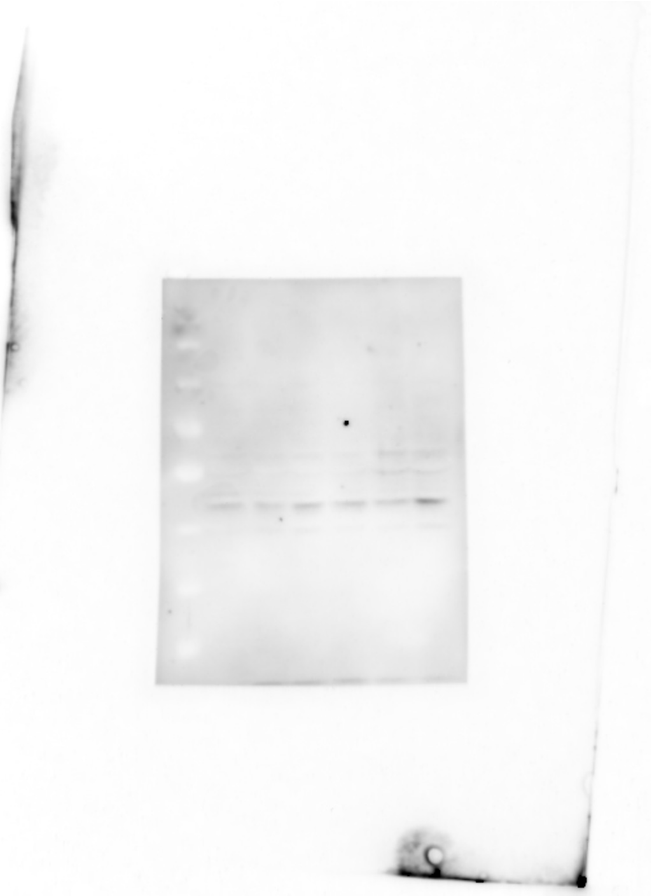

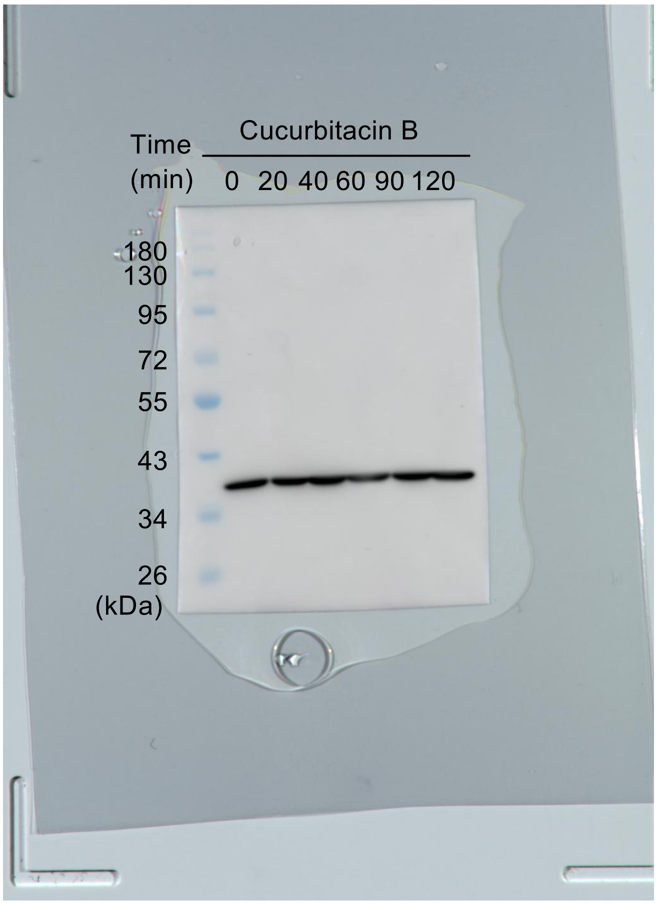


WB: GAPDH (reprobed)

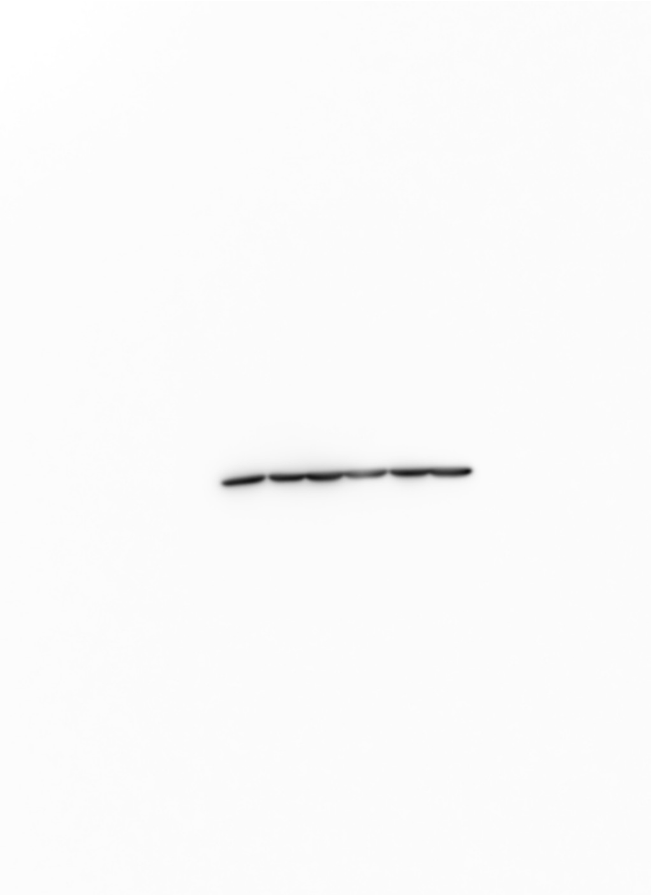

## Figure S68: Original blots (1) in Figures 9G and 9H

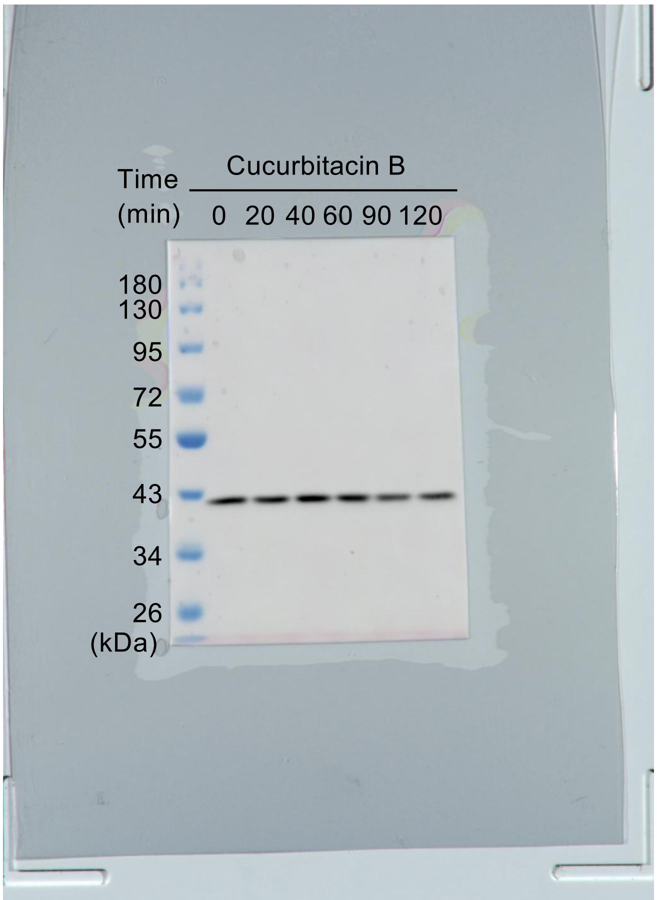


WB: p38 MAPK

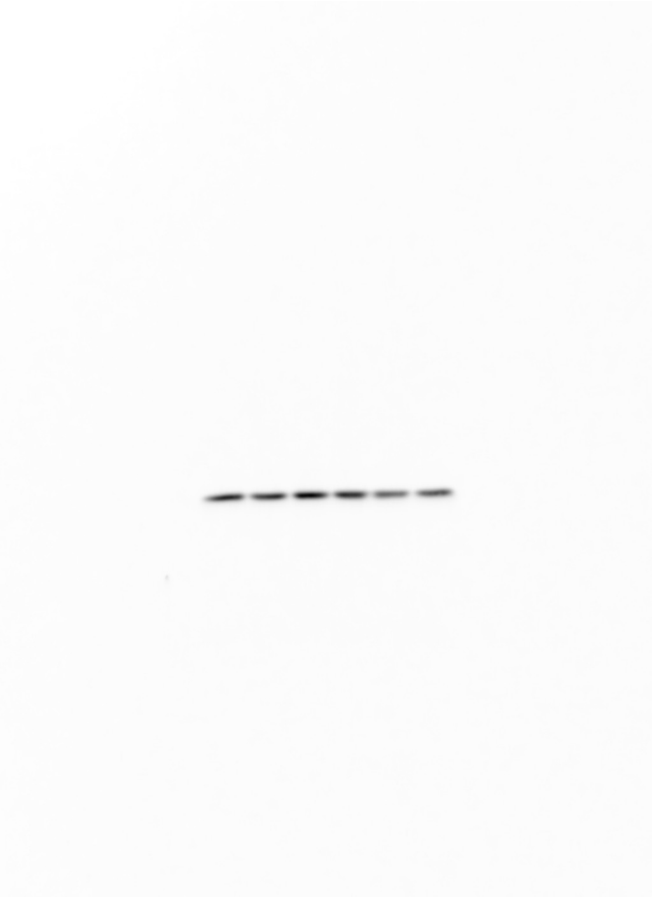

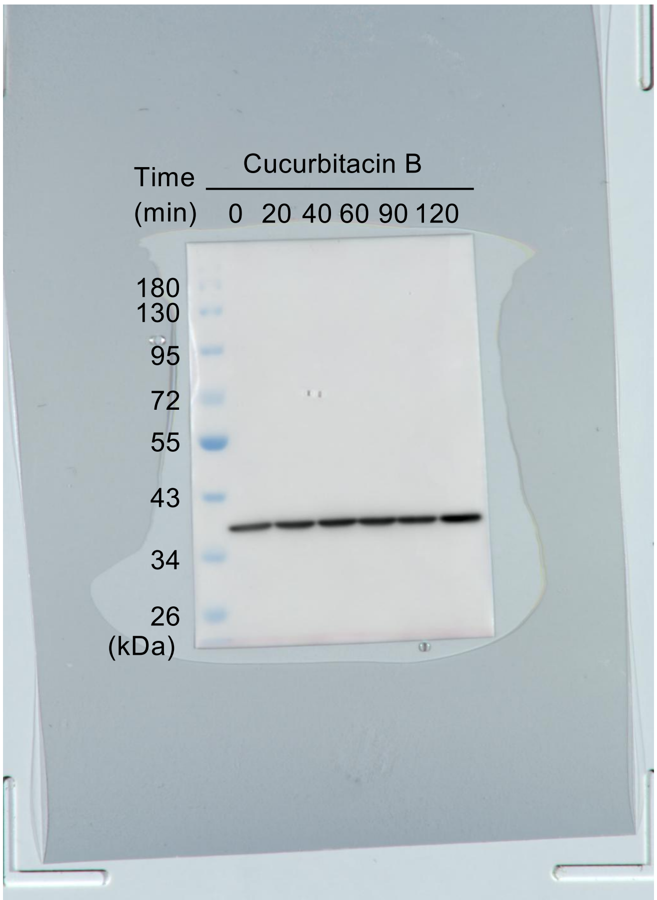


WB: GAPDH (reprobed)

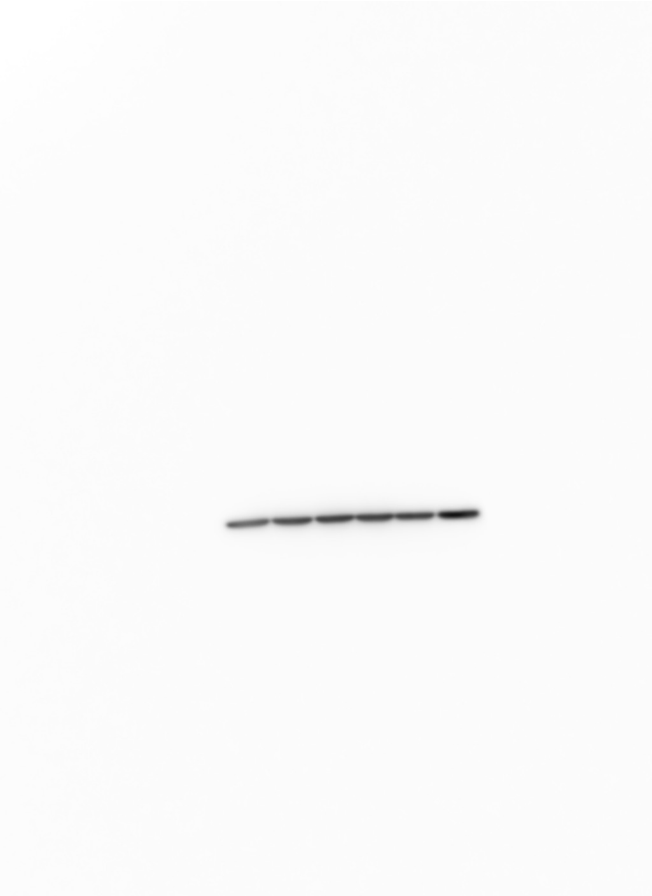

## Figure S69: Original blots (2) in Figures 9G and 9H

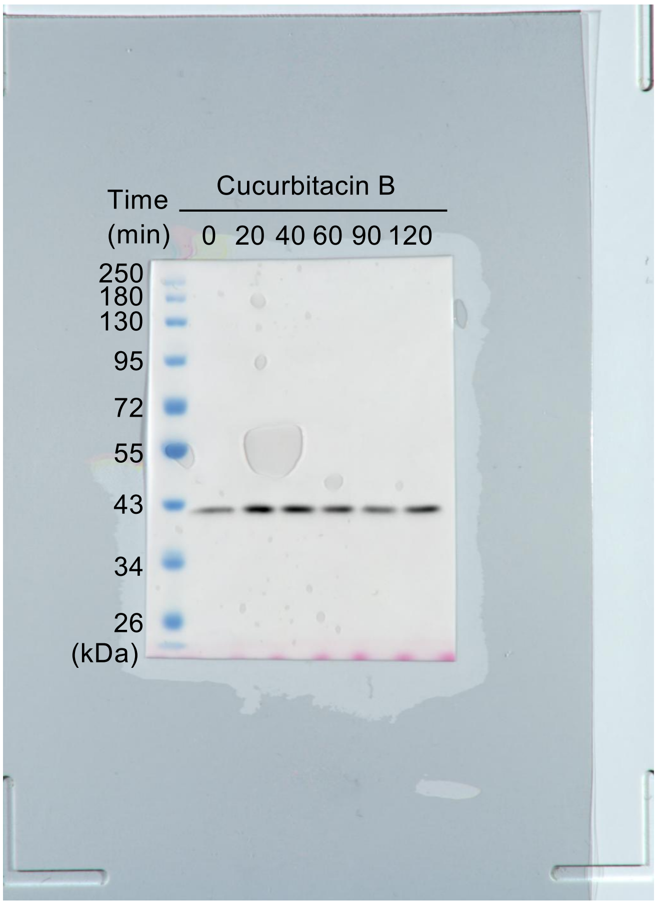


WB: p38 MAPK



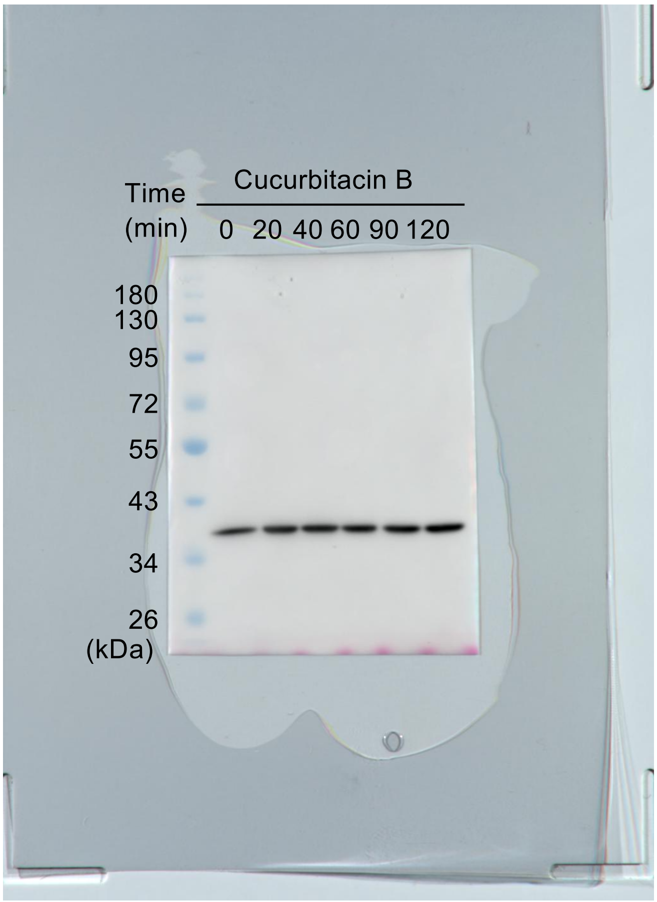


WB: GAPDH (reprobed)

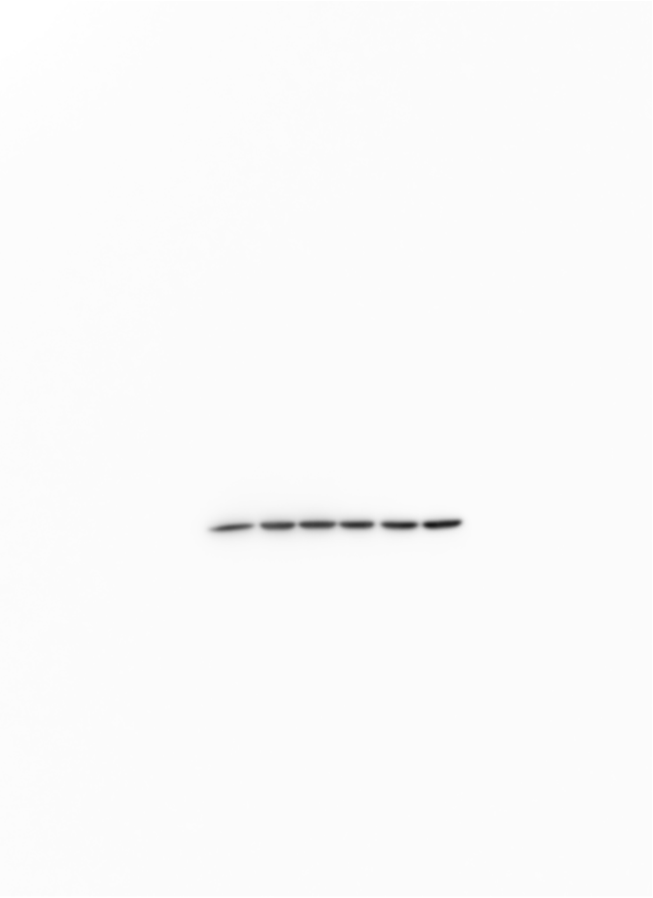

## Figure S70: Original blots (3) in Figures 9G and 9H

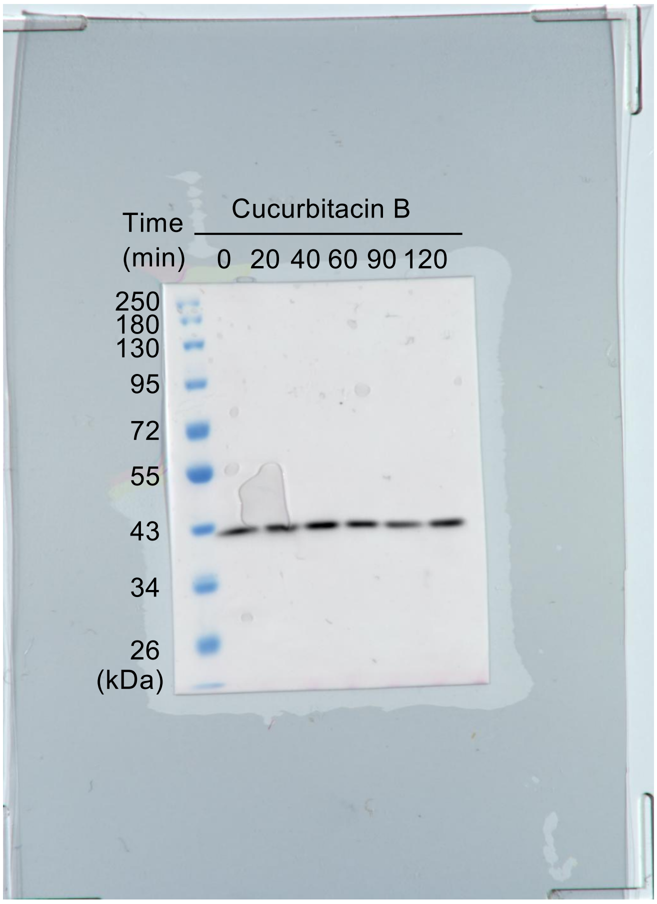


WB: p38 MAPK

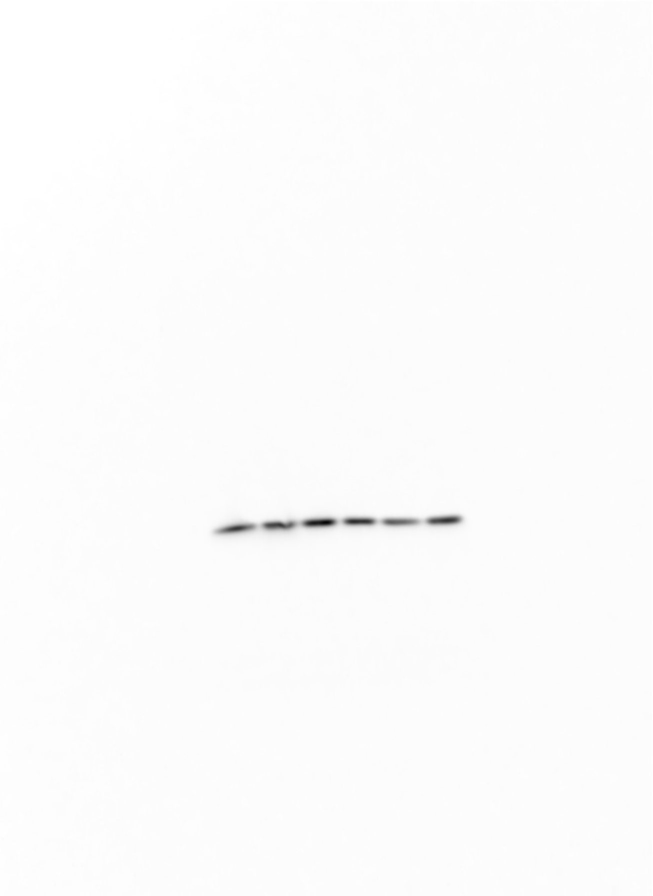

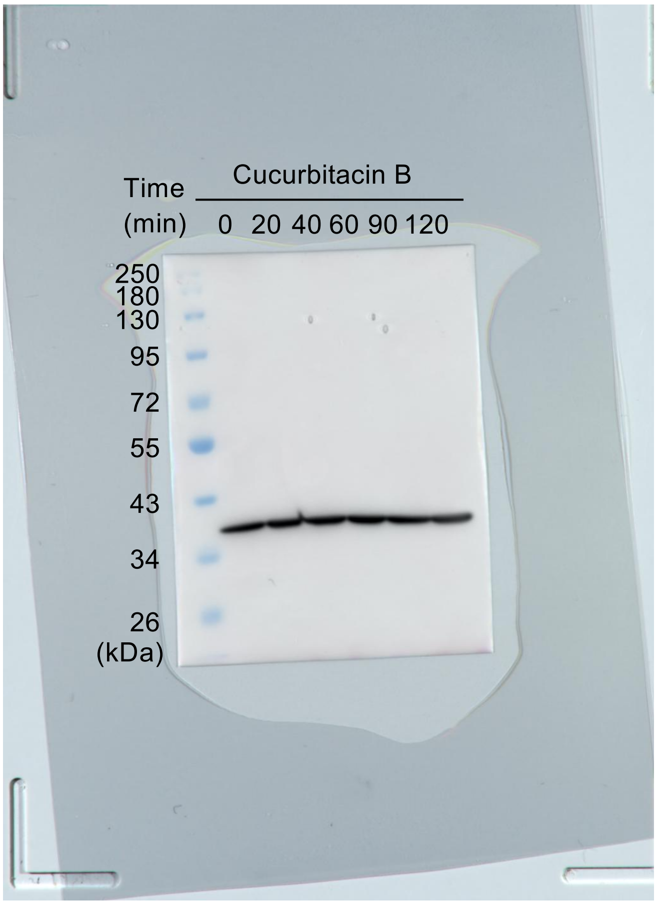


WB: GAPDH (reprobed)

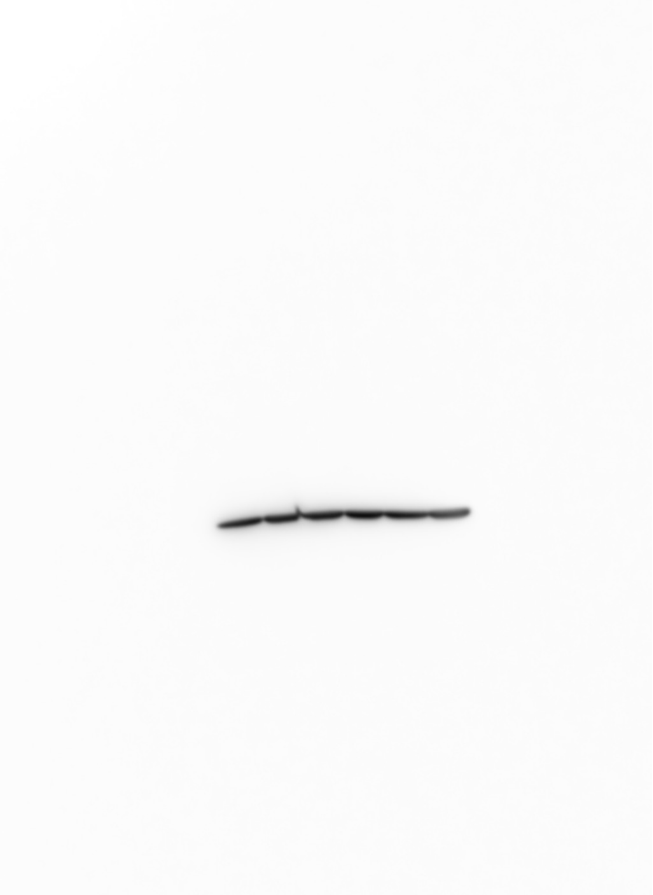

## Figure S71: Original blots in Figure 9I (phospho-JNK)

WB: Phospho-JNK

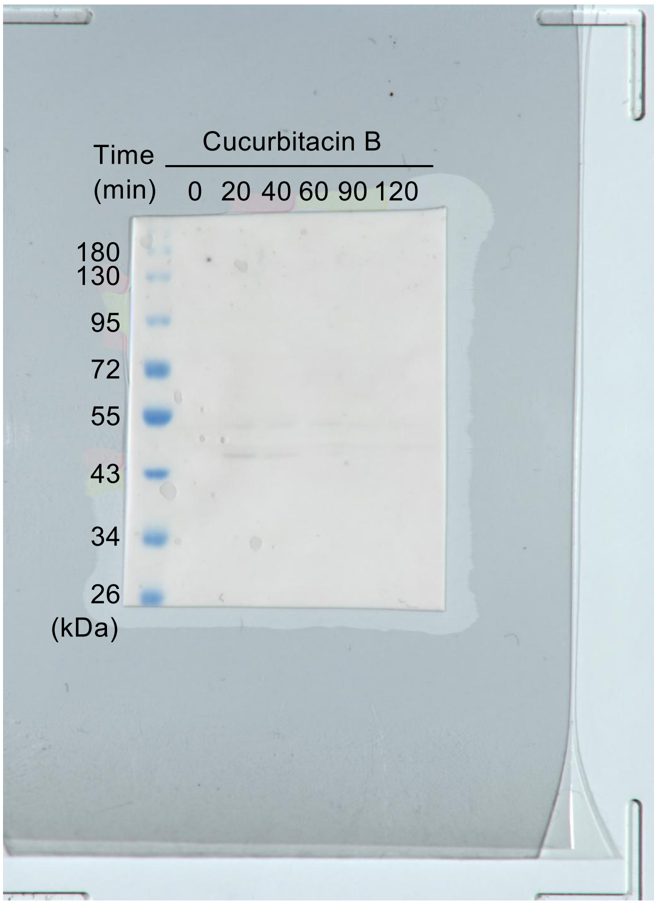




WB: GAPDH (reprobed)

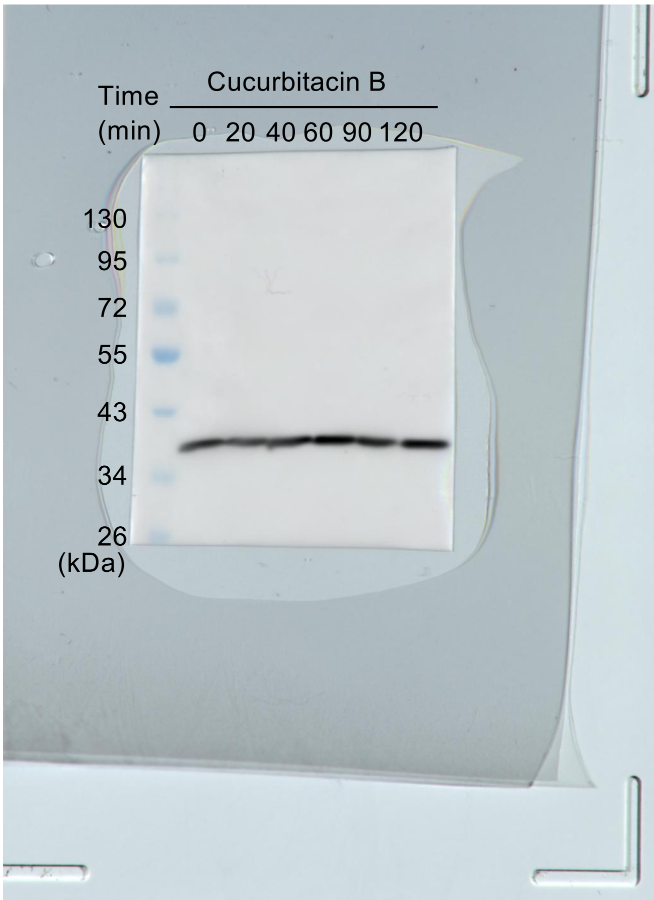


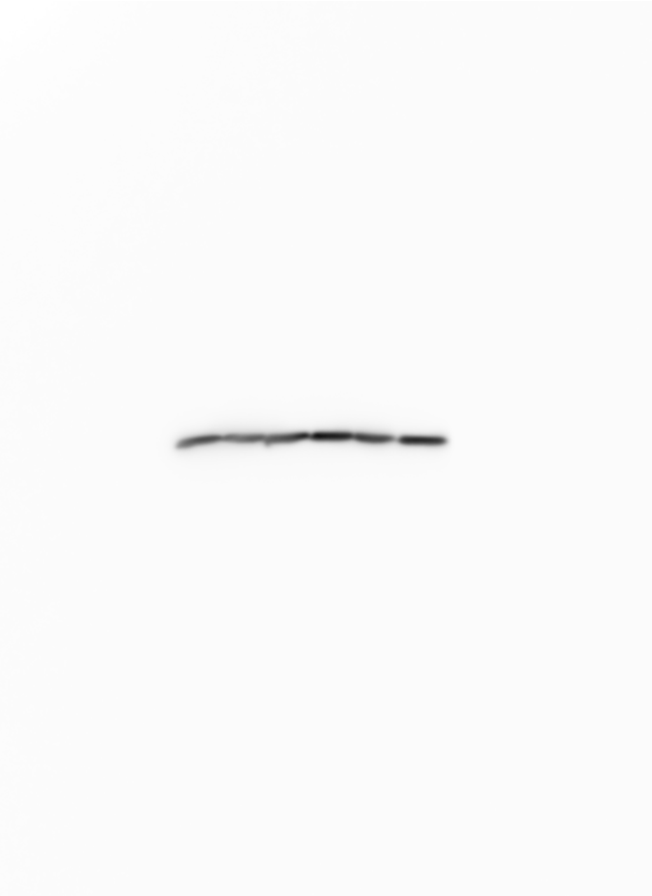

# Figure S72: Original blots in Figure 9I (JNK)

WB: JNK

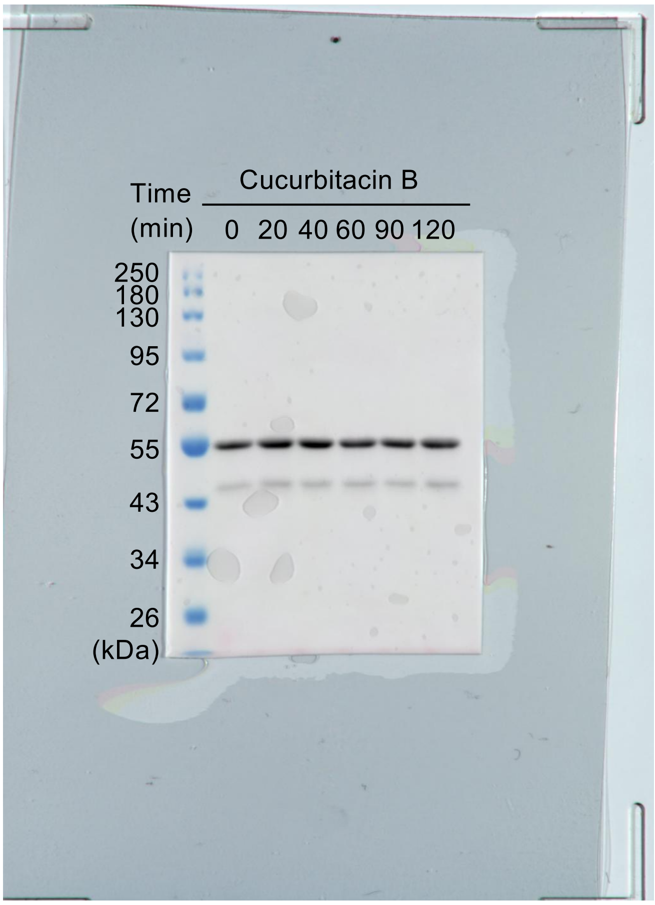


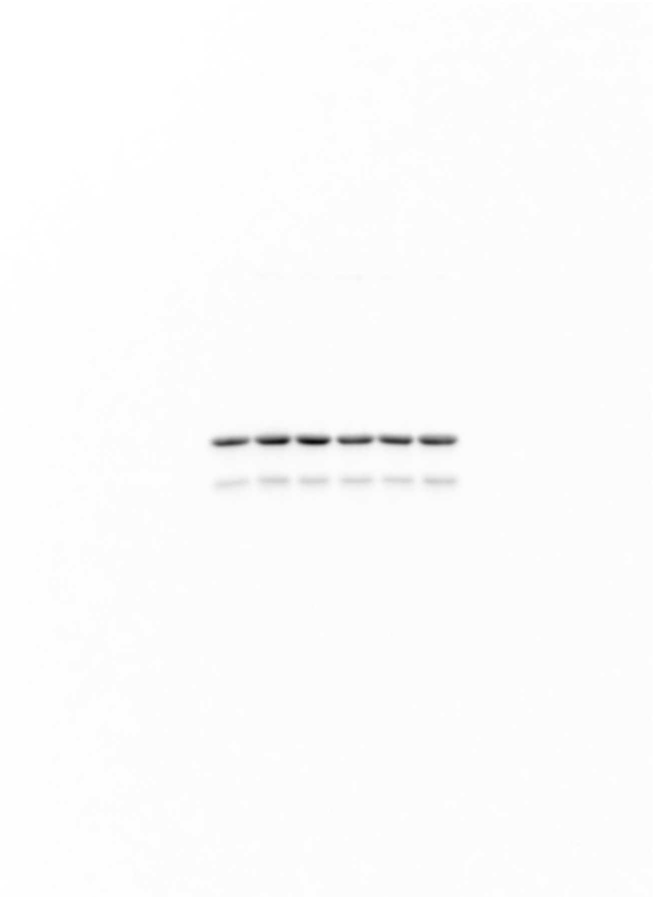

WB: GAPDH (reprobed)

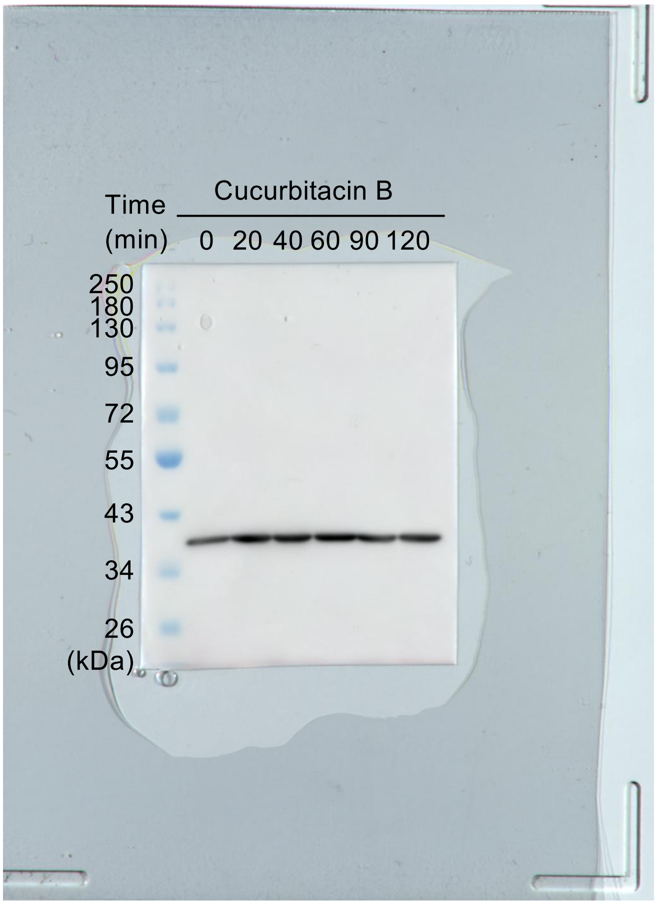


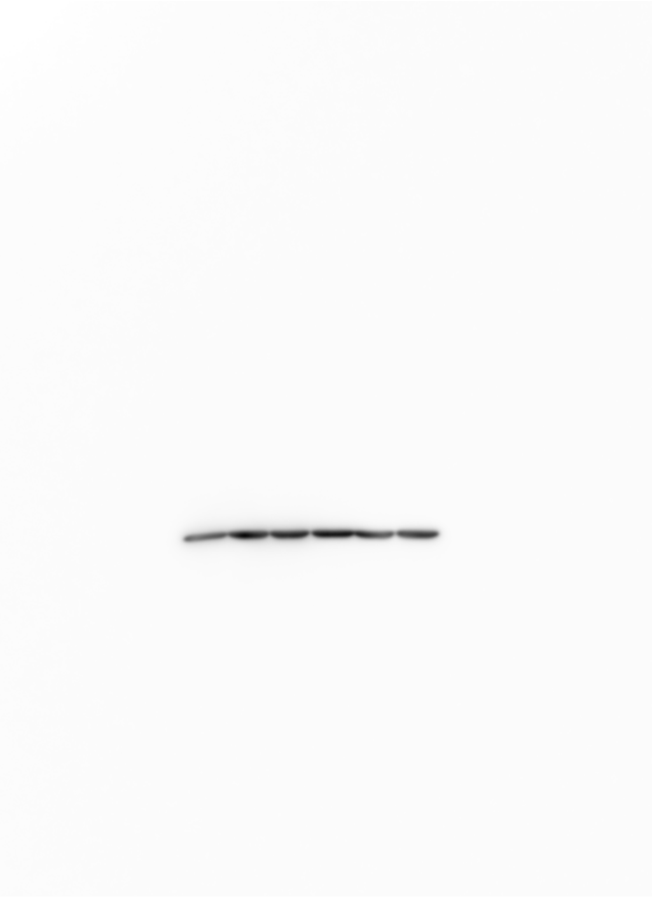

## Figure S73: Original blots (1) in Figures 9J, 9K, 9N, and 9O

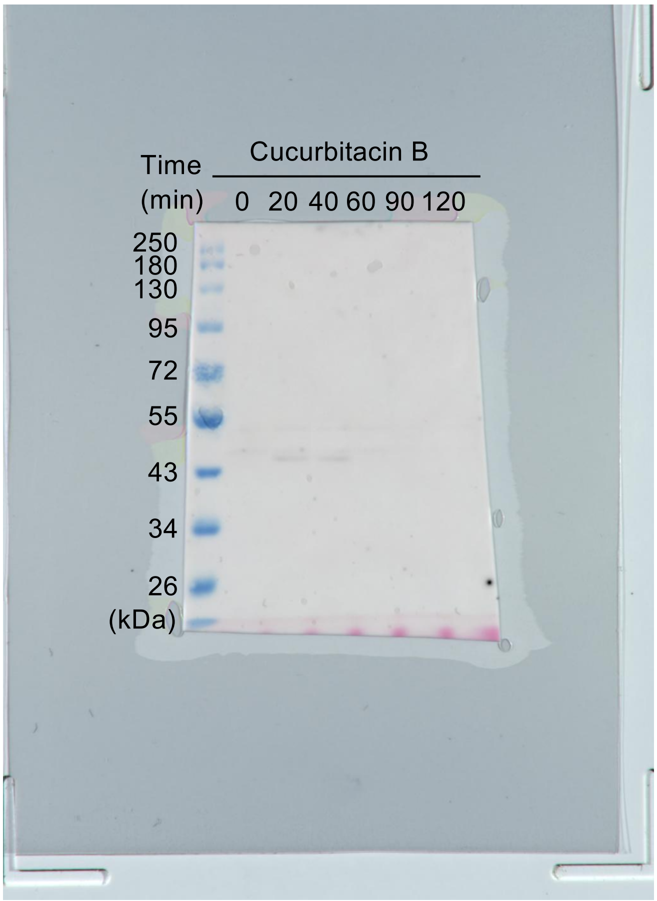


WB: Phospho-JNK

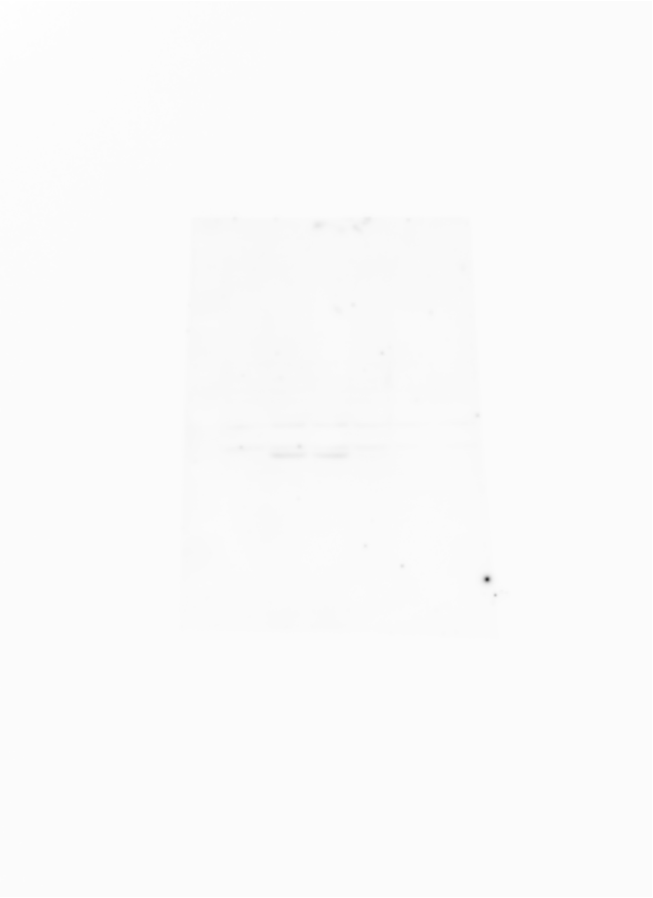

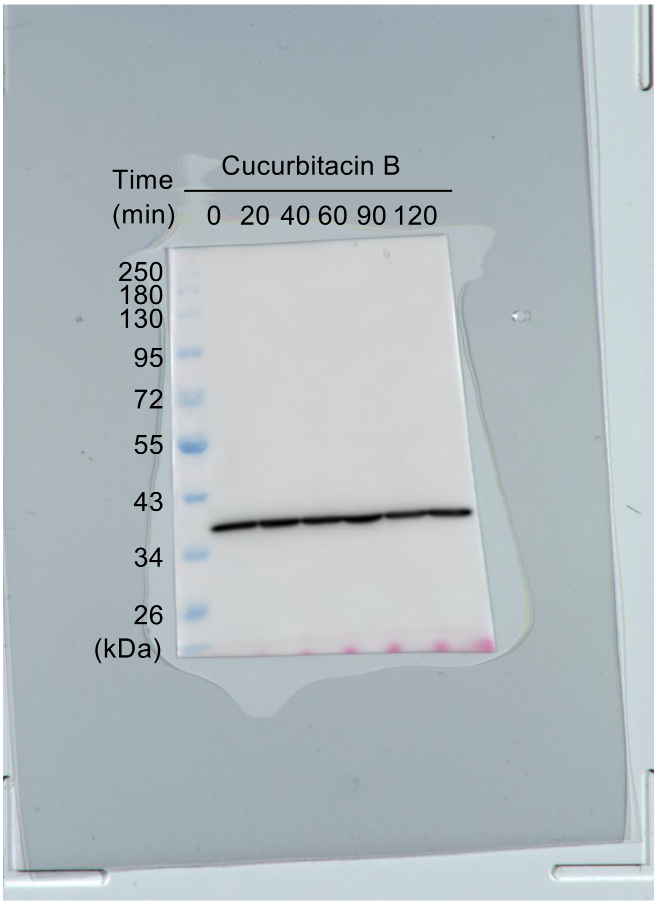


WB: GAPDH (reprobed)

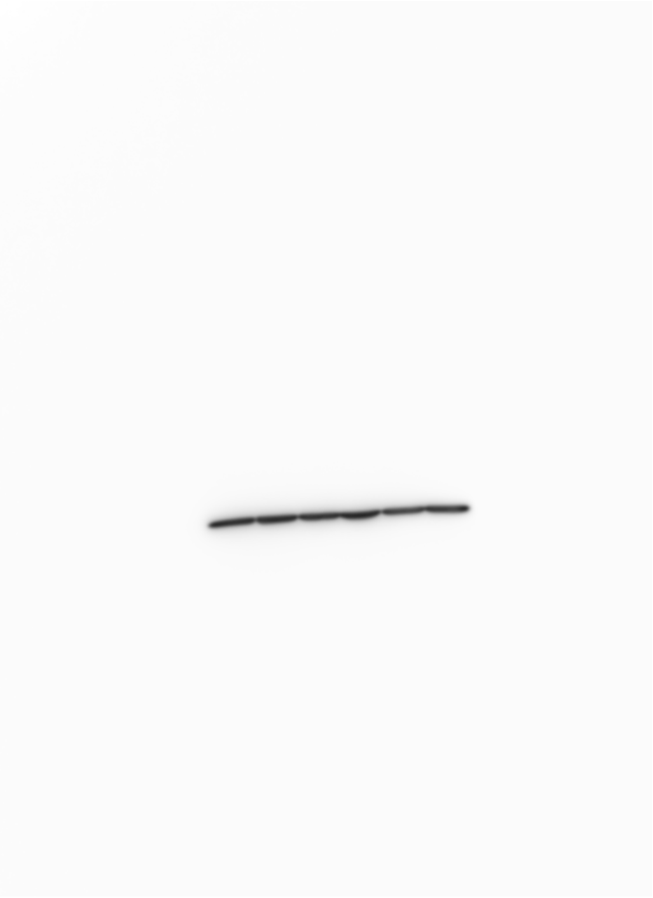

# Figure S74: Original blots (2) in Figures 9J, 9K, 9N, and 9O

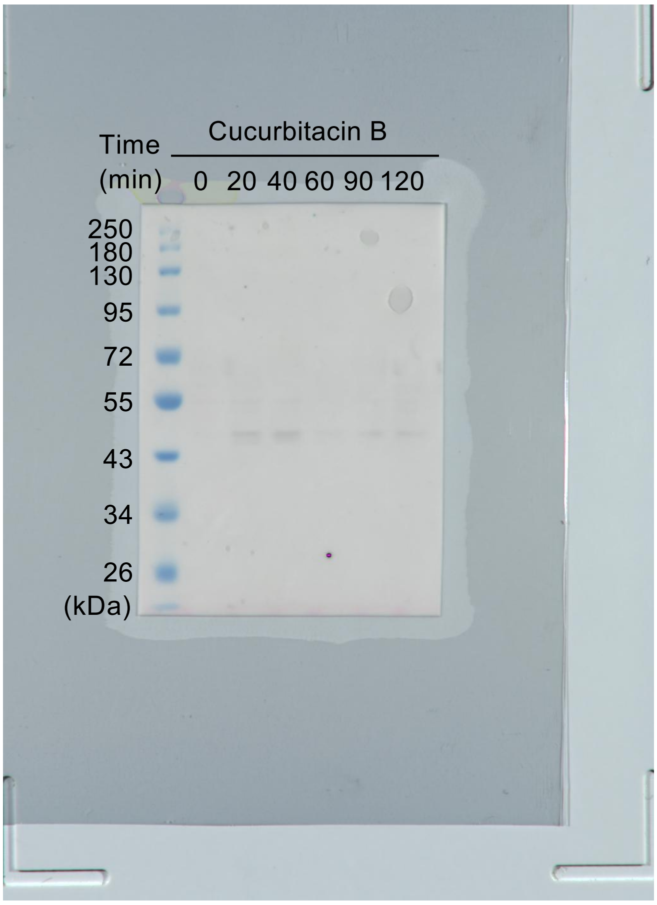


WB: Phospho-JNK



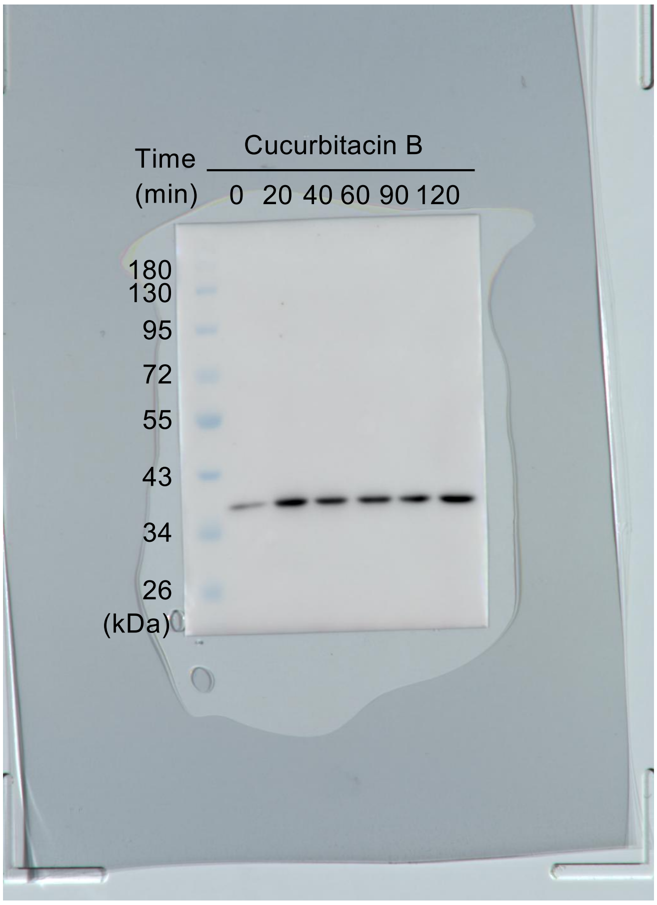


WB: GAPDH (reprobed)

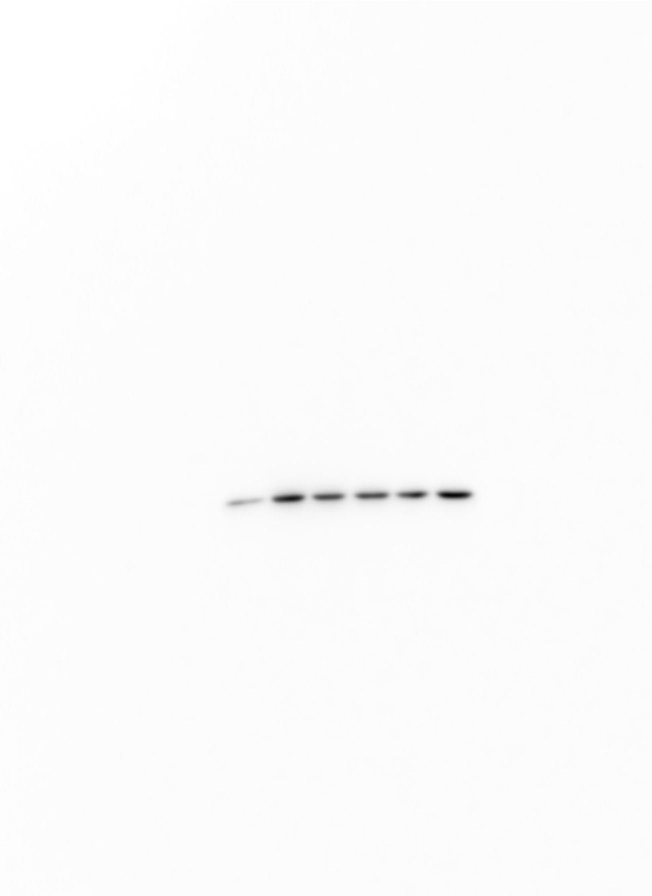

## Figure S75: Original blots (3) in Figures 9J, 9K, 9N, and 9O

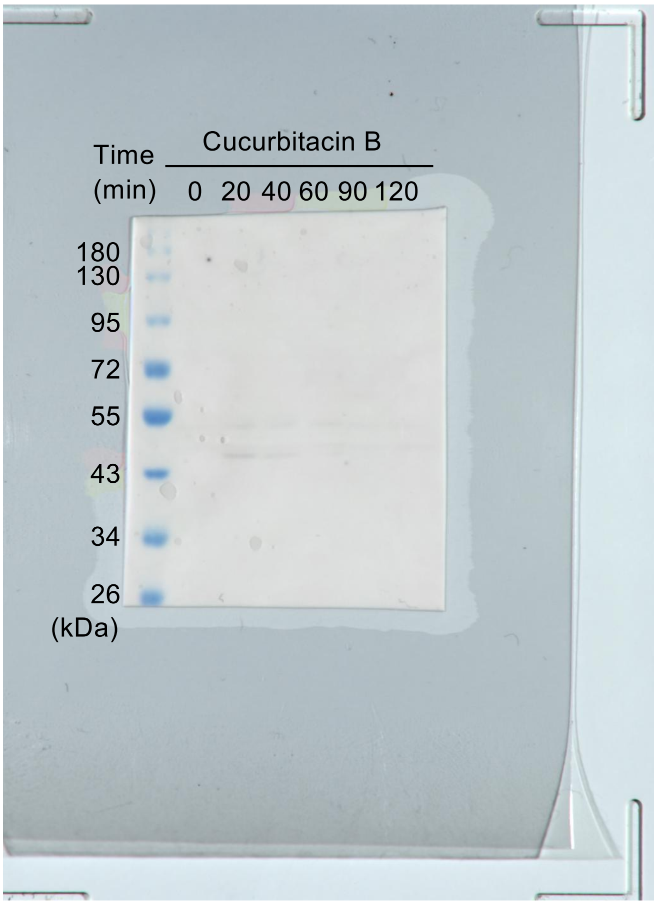


WB: Phospho-JNK



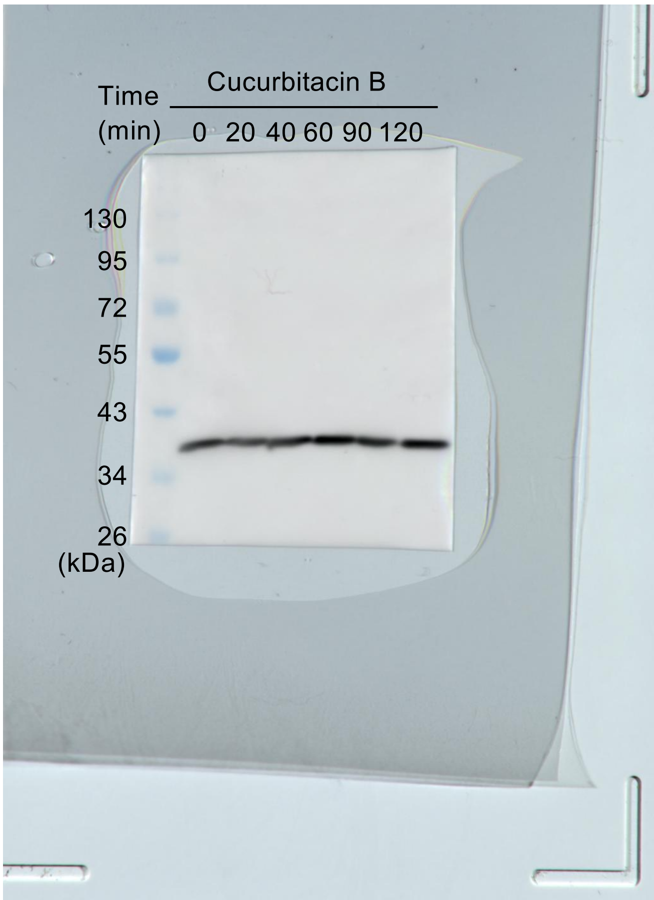


WB: GAPDH (reprobed)

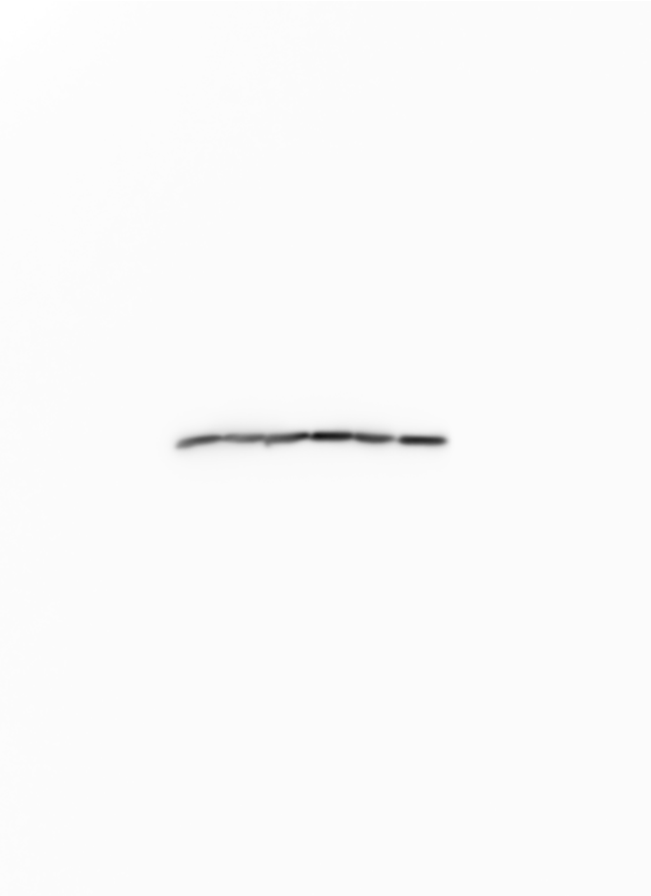

# Figure S76: Original blots (1) in Figures 9L–9O

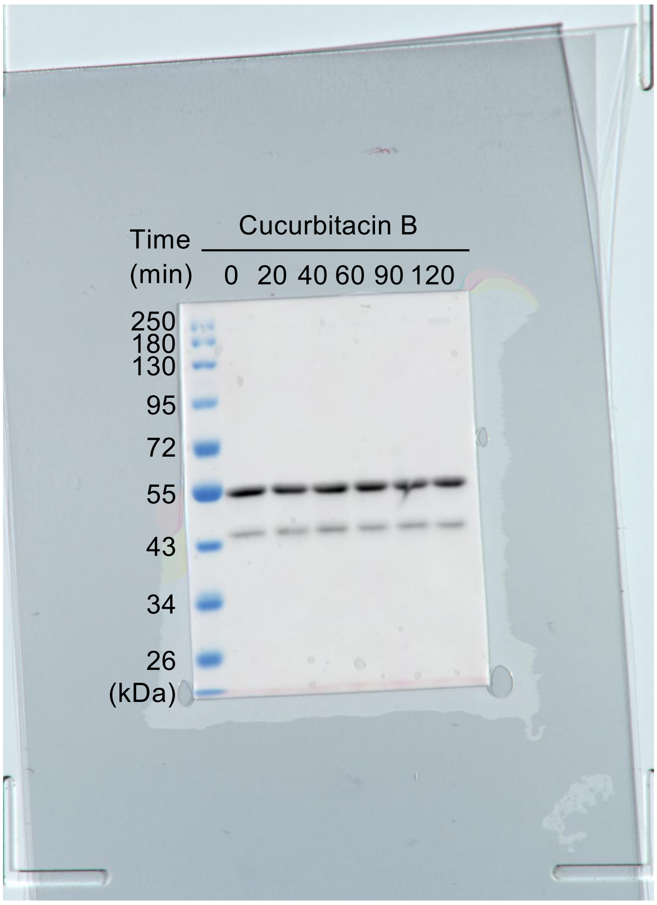


WB:JNK

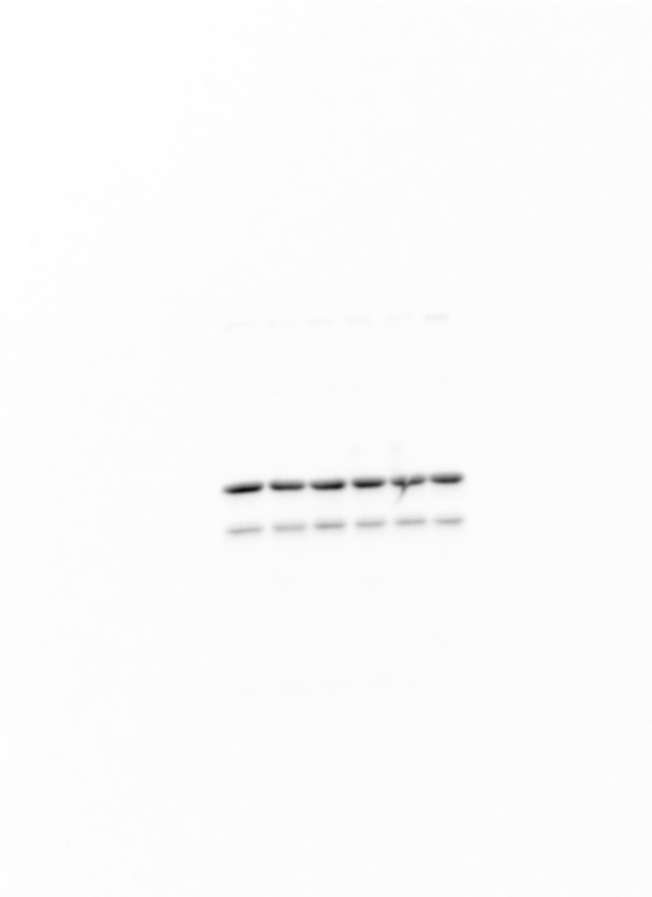

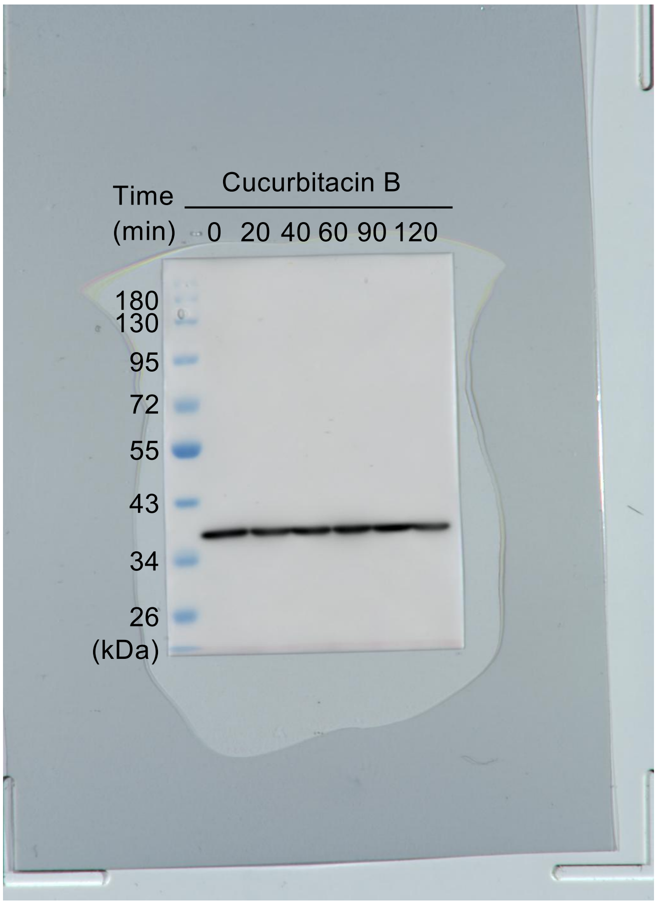


WB: GAPDH (reprobed)

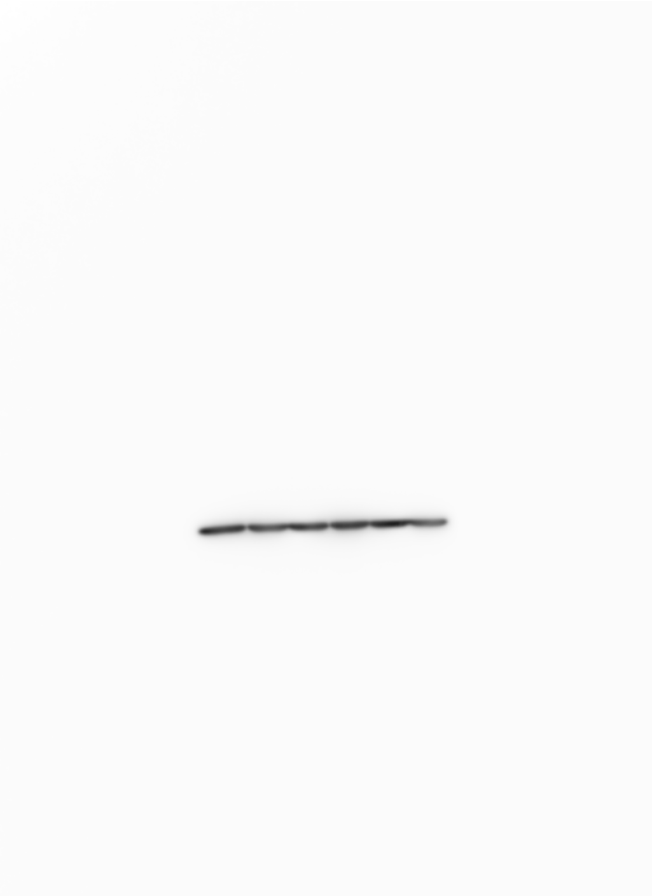

# Figure S77: Original blots (2) in Figures 9L–9O

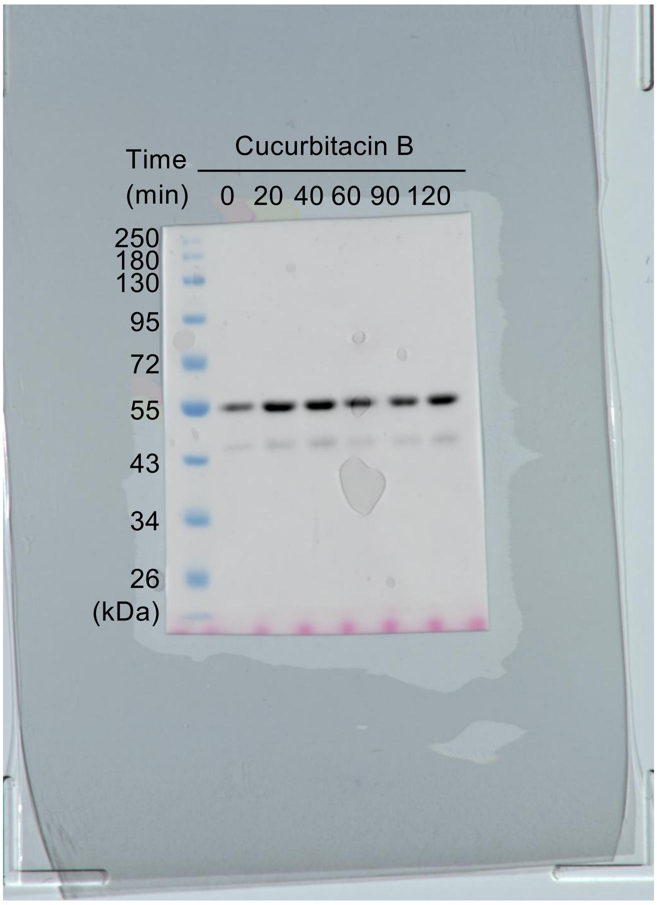


WB: JNK

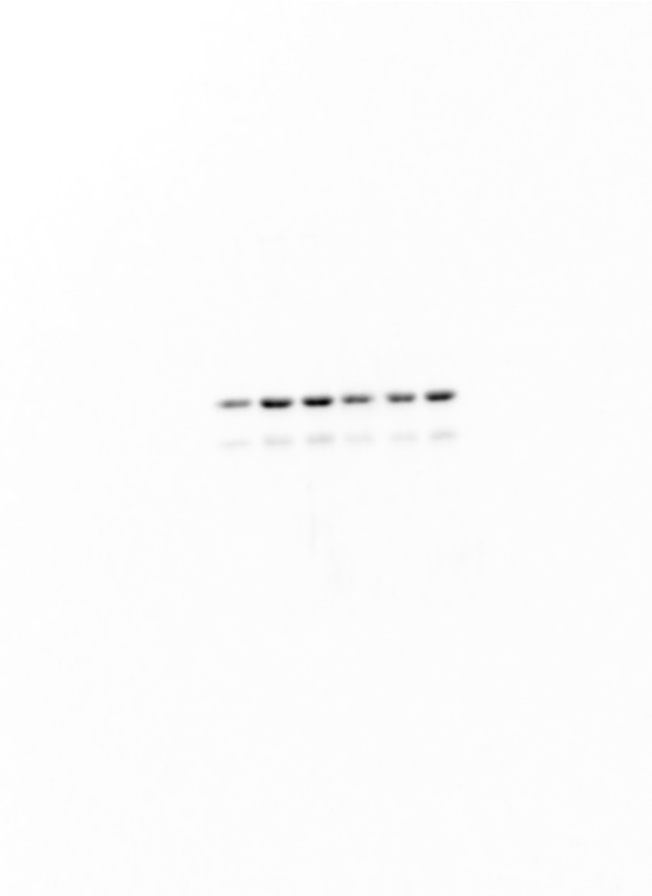

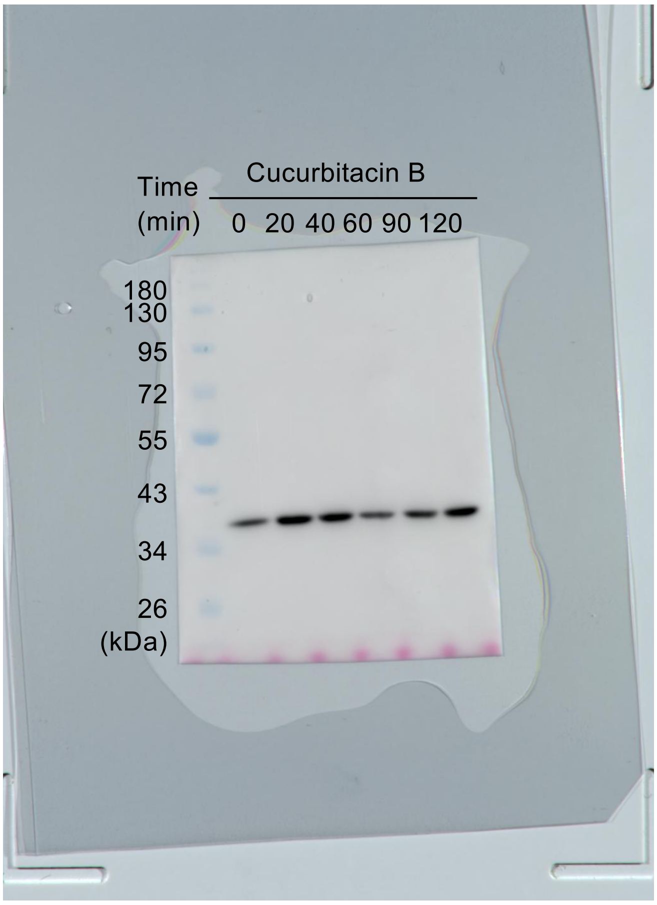


WB: GAPDH (reprobed)

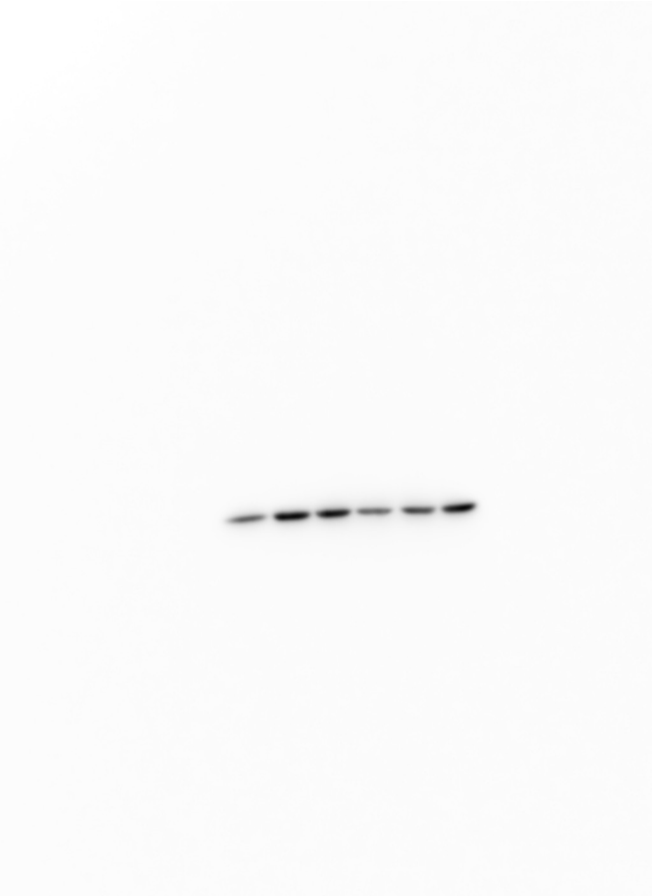

# Figure S78: Original blots (3) in Figures 9L–9O

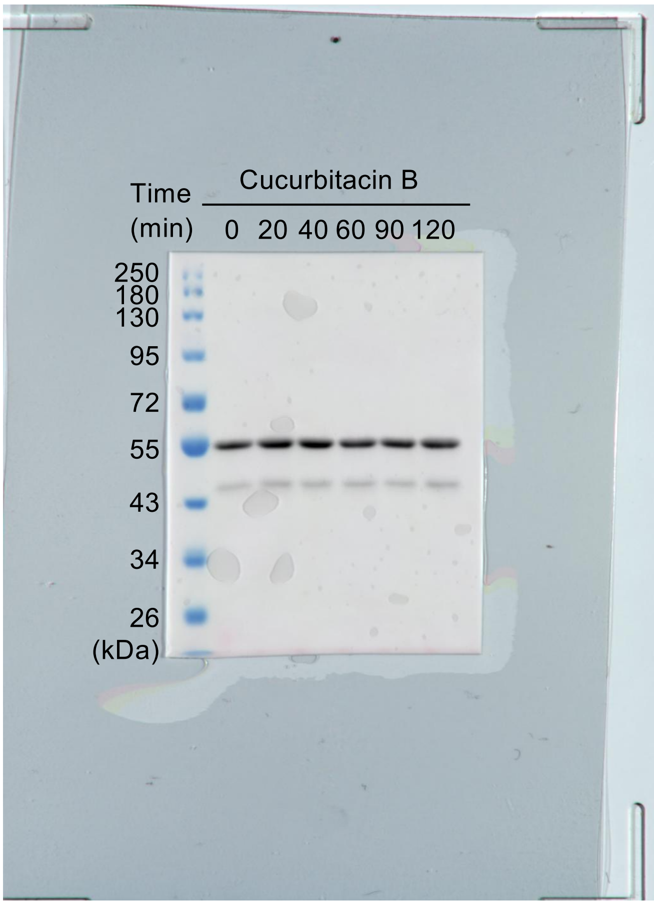


WB: JNK

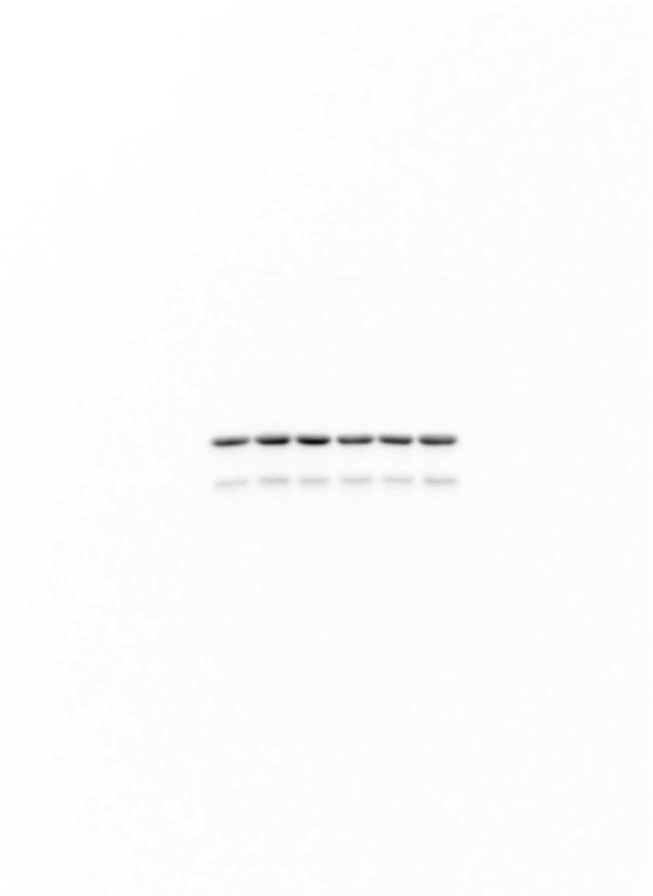

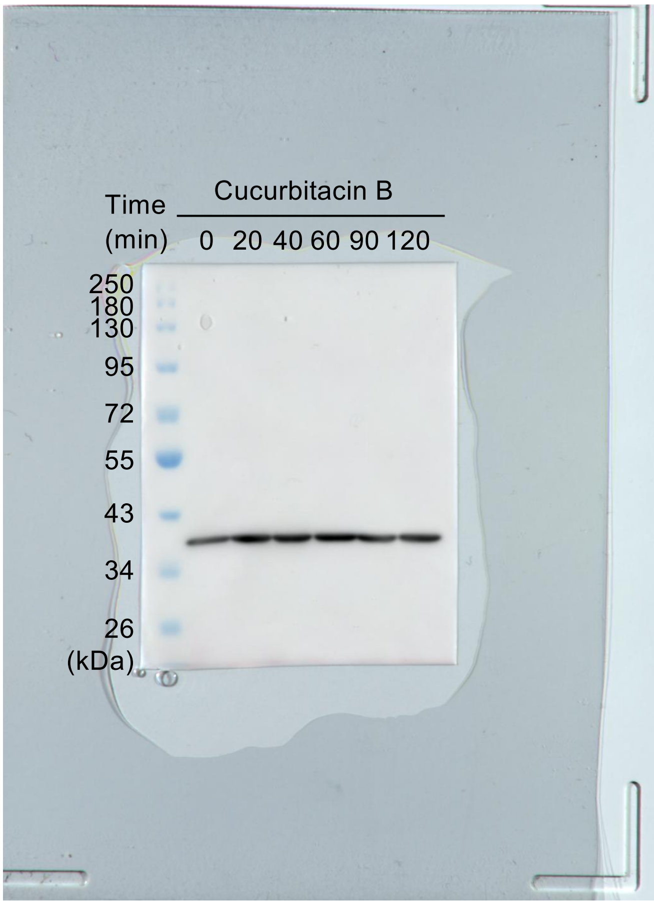


WB: GAPDH (reprobed)

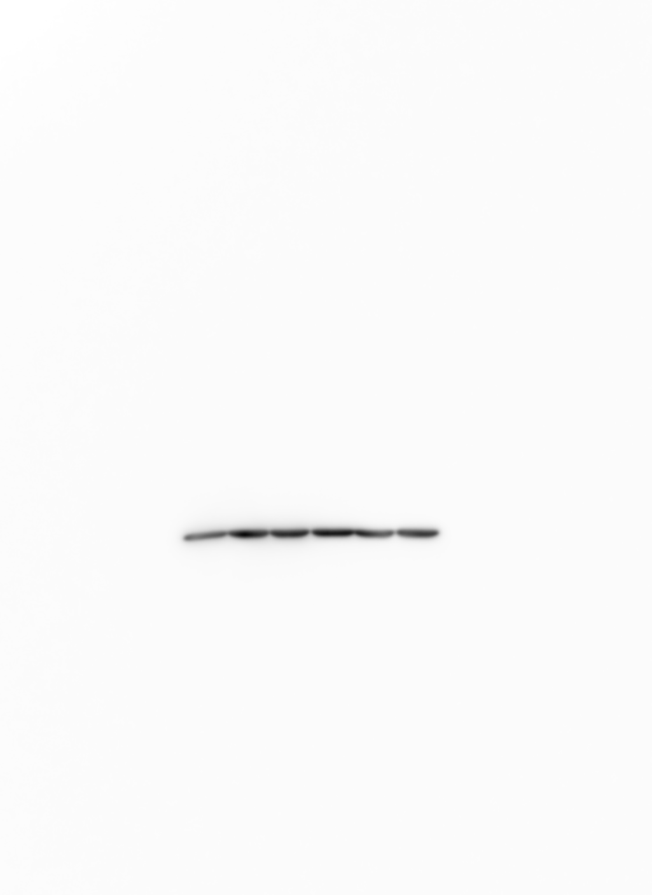

## Figure S79: Original blots in Figure 10A (phospho-RAF1)

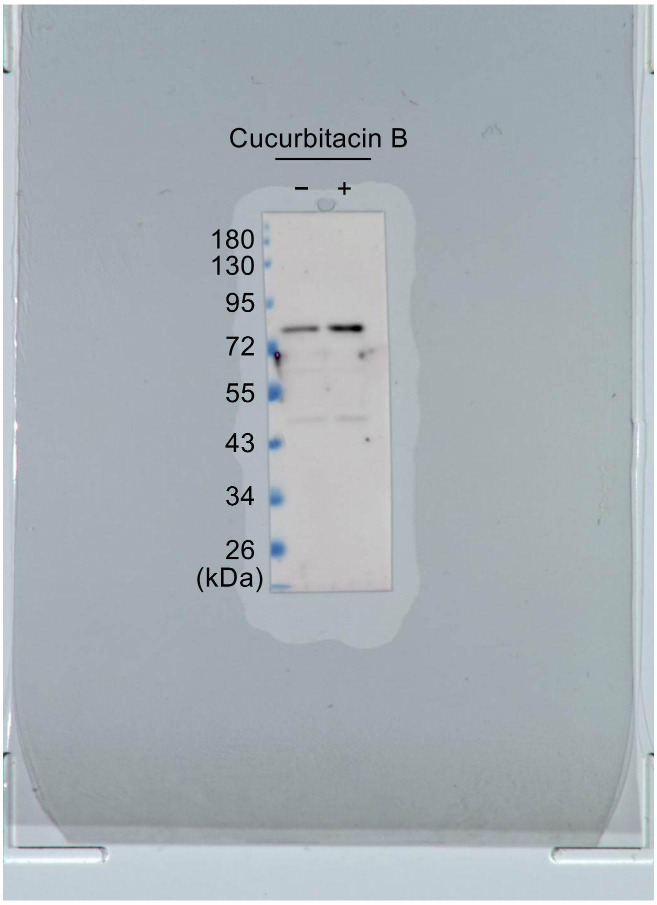


WB: Phospho-RAF1

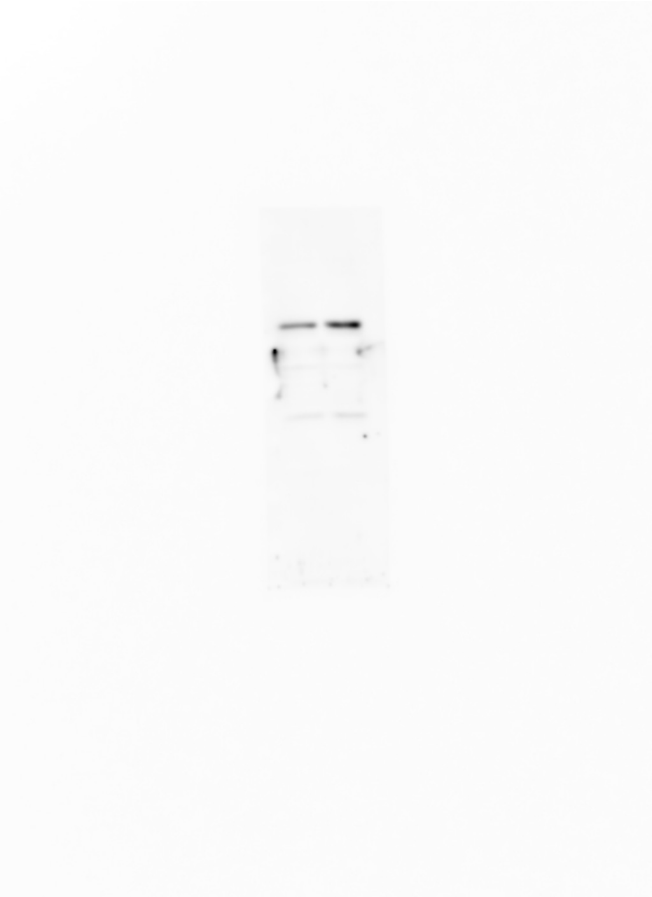

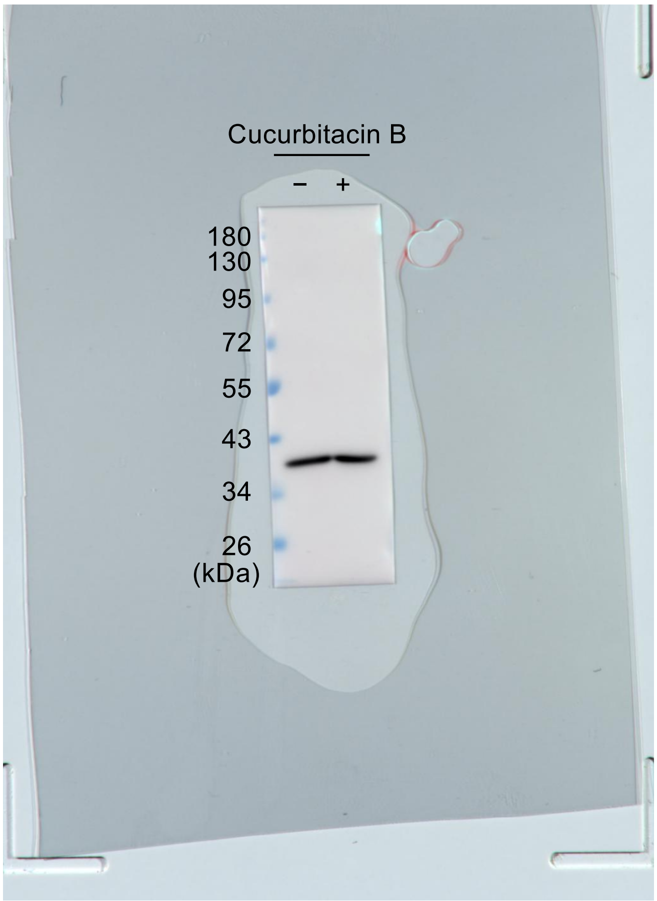


WB: GAPDH (reprobed)

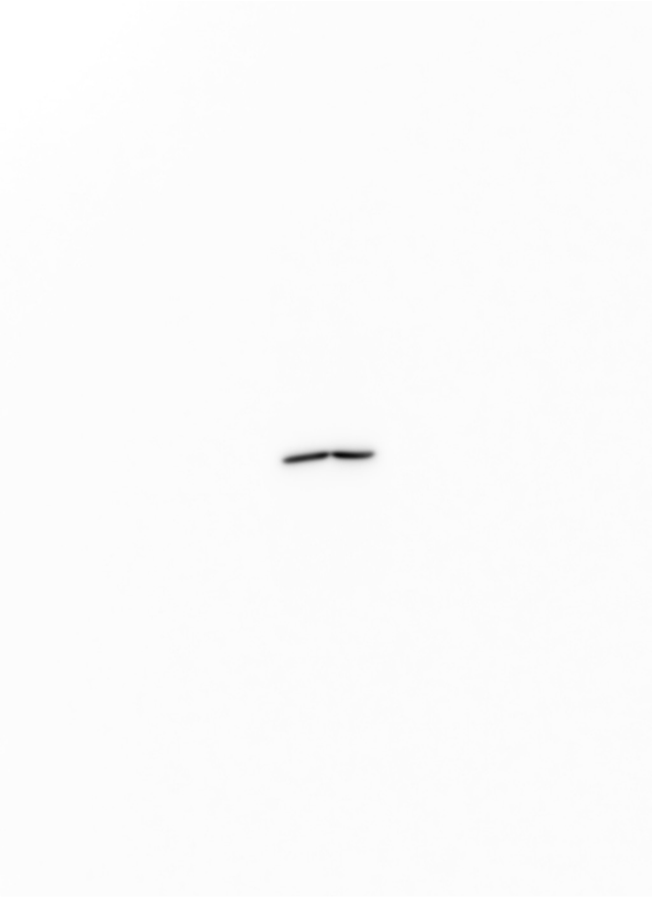

# Figure S80: Original blots in Figure 10A (RAF1)

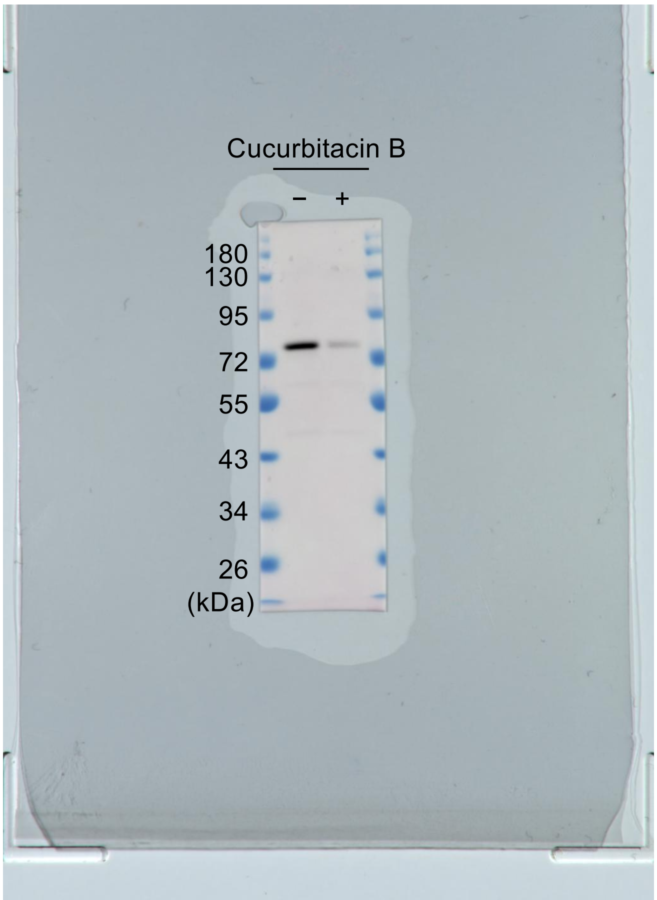


WB: RAF1



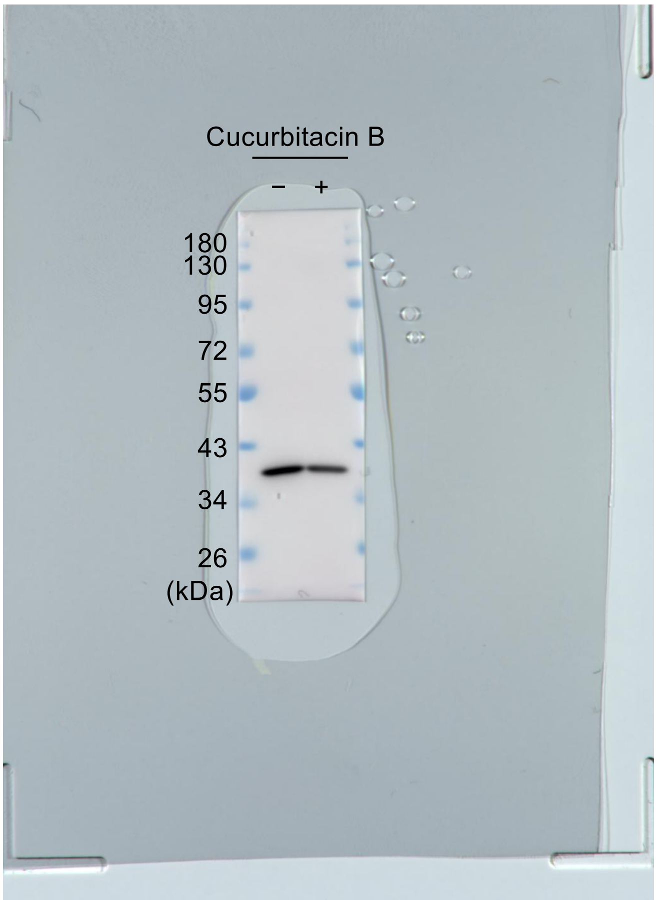


WB: GAPDH (reprobed)

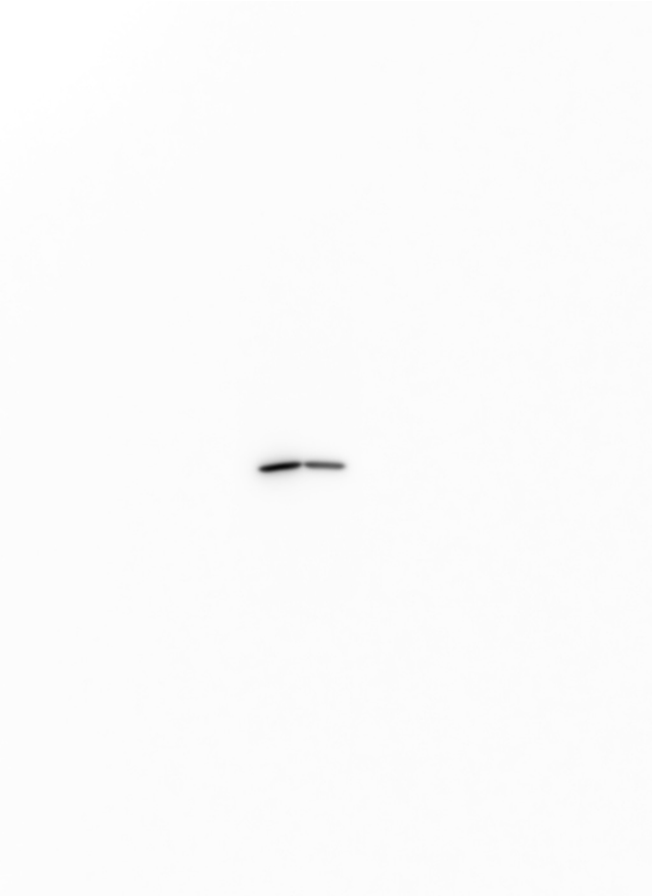

# Figure S81: Original blots (1) in Figures 10B and 10D

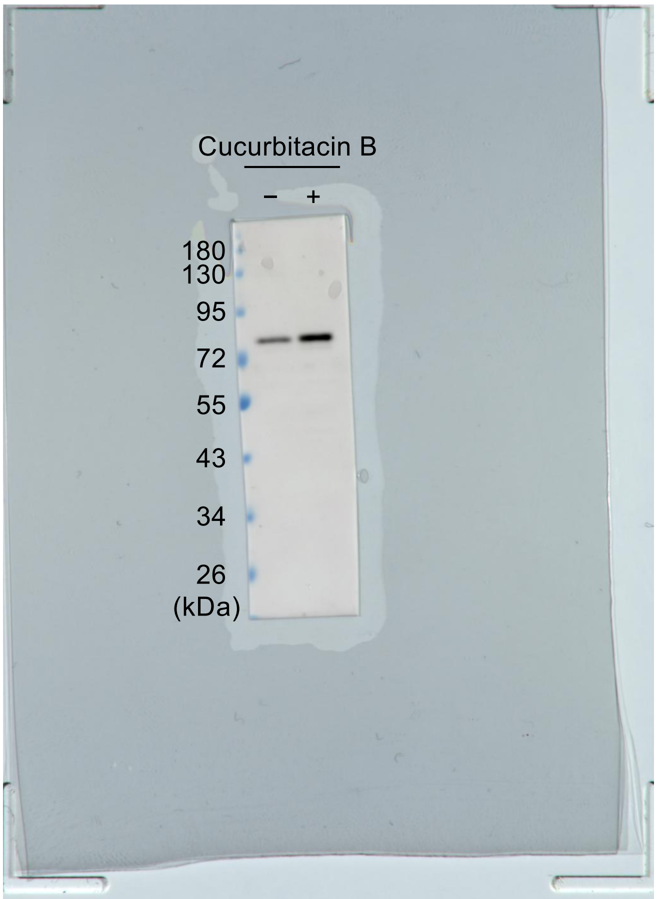


WB: Phospho-RAF1

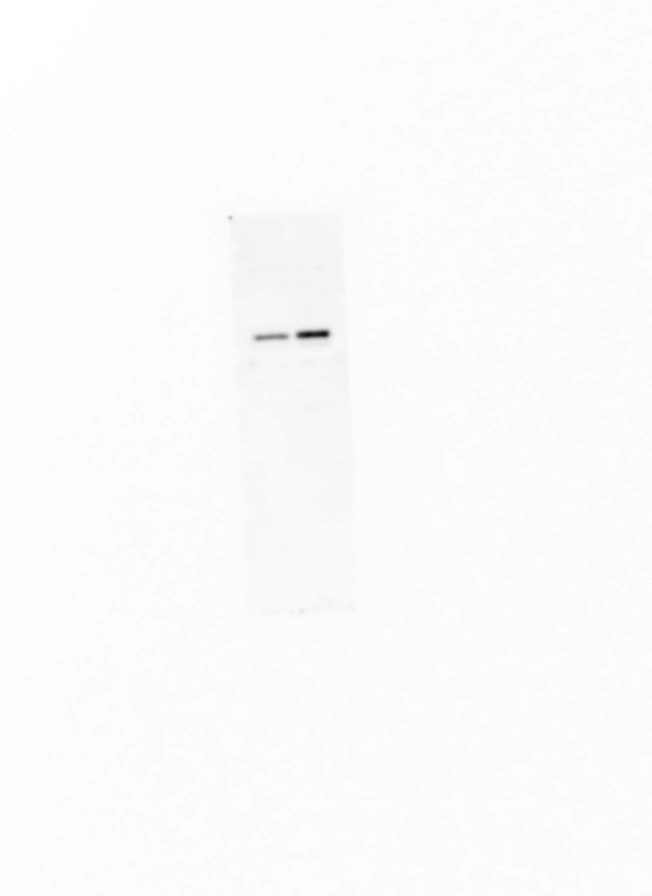

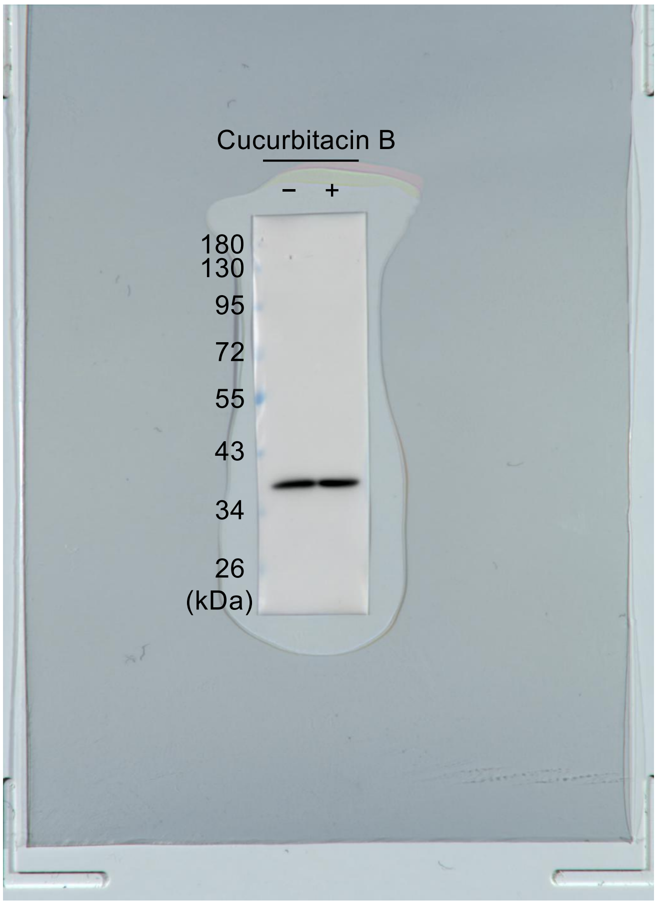


WB: GAPDH (reprobed)

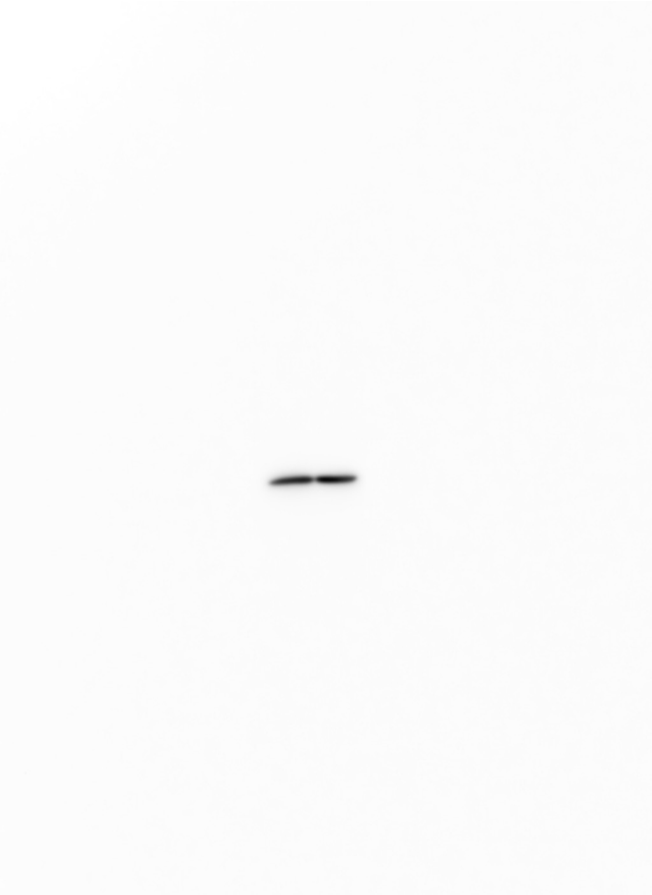

## Figure S82: Original blots (2) in Figures 10B and 10D

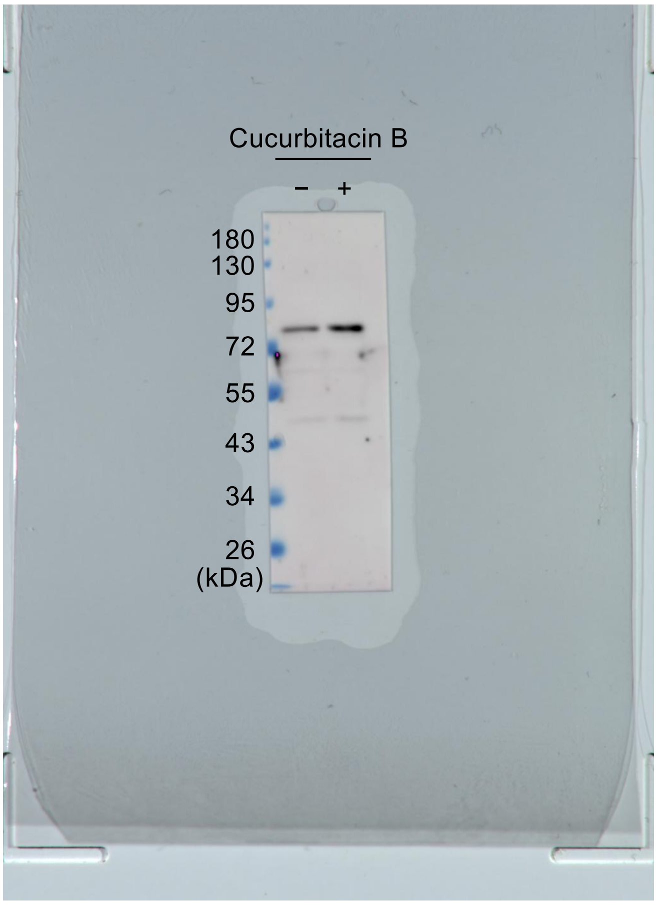


WB: Phospho-RAF1

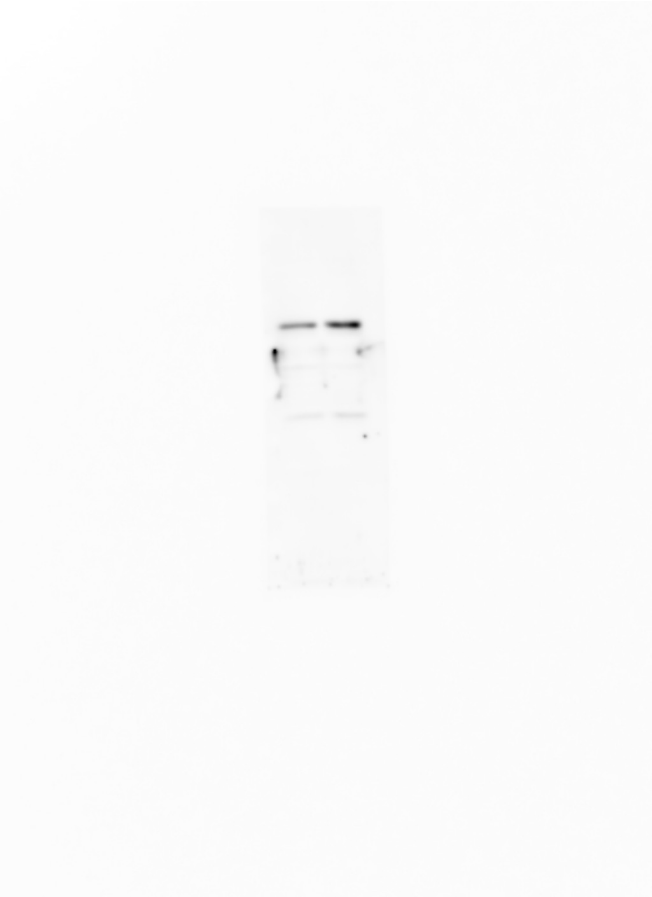

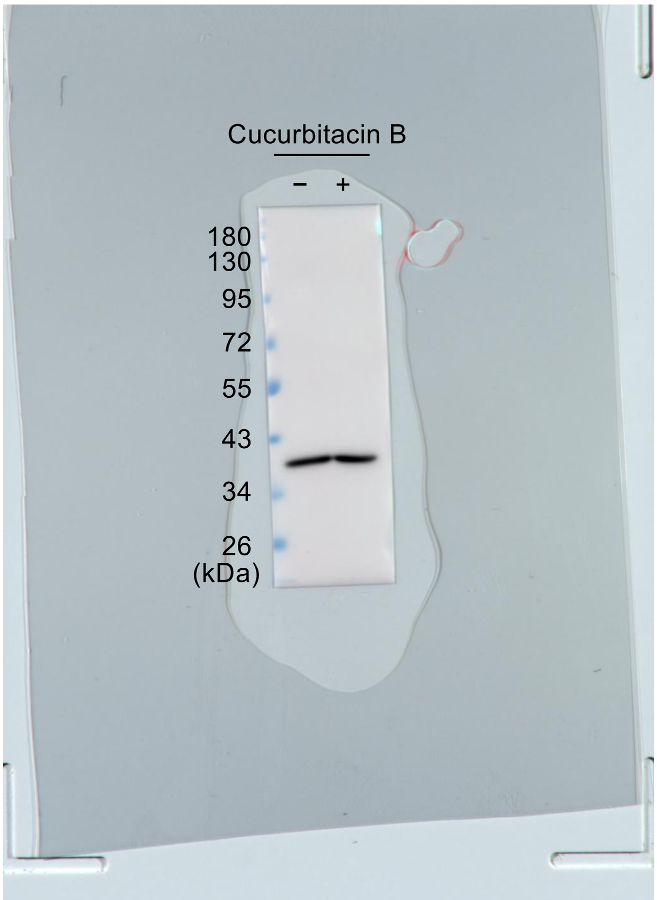


WB: GAPDH (reprobed)

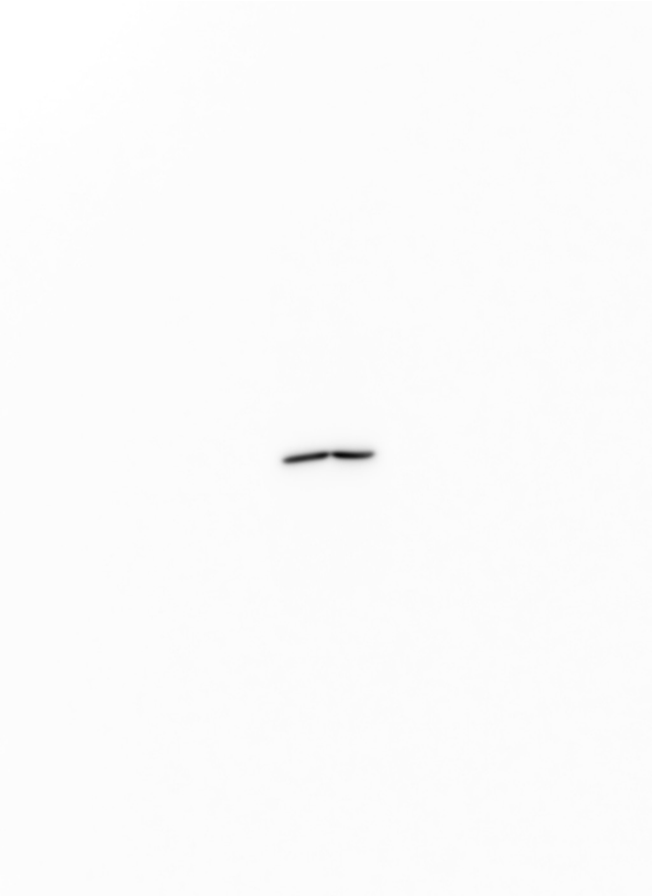

## Figure S83: Original blots (3) in Figures 10B and 10D

WB: Phospho-RAF1

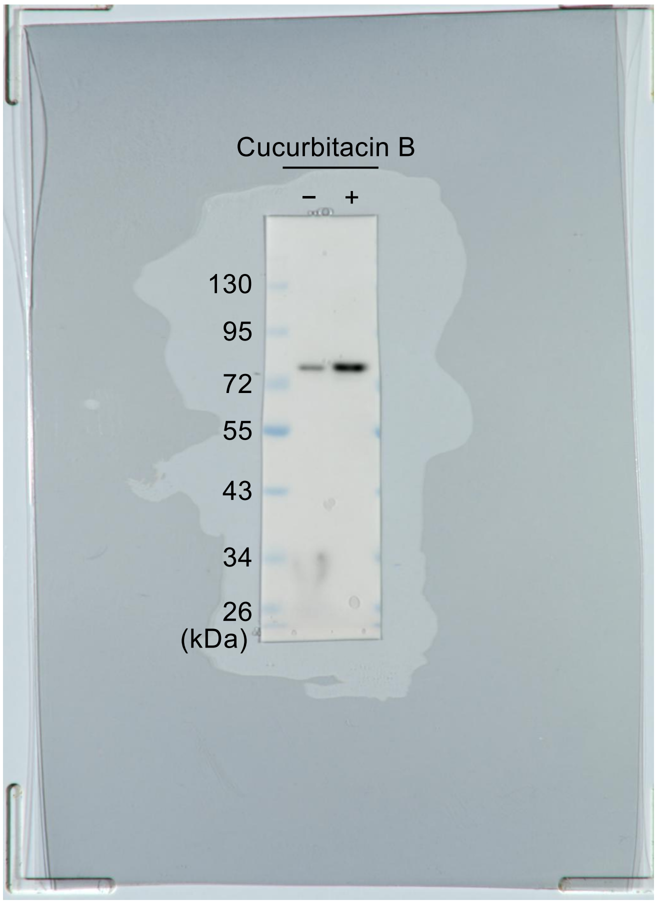




WB: GAPDH (reprobed)

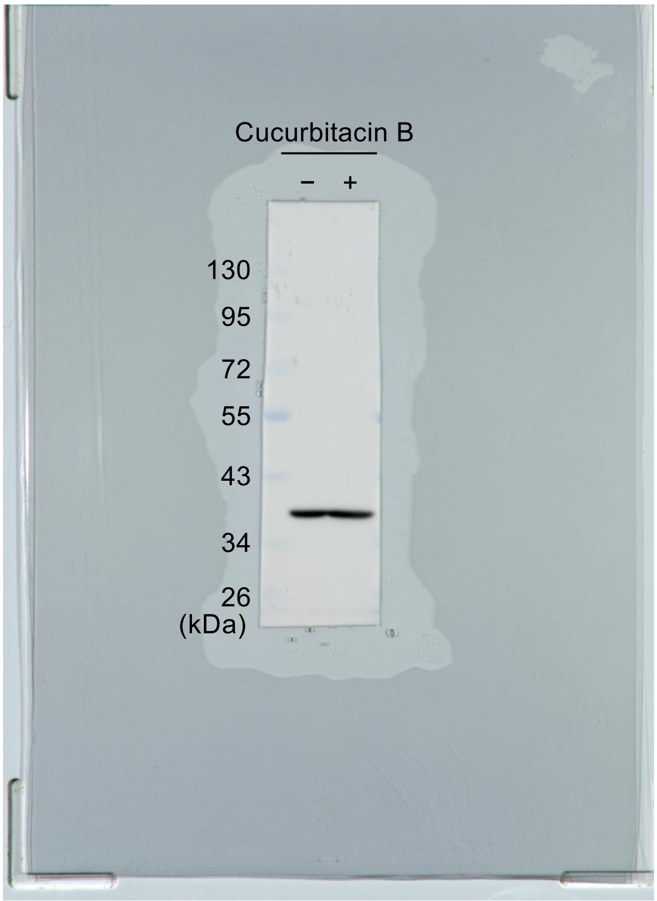

# Figure S84: Original blots (1) in Figures 10C and 10D

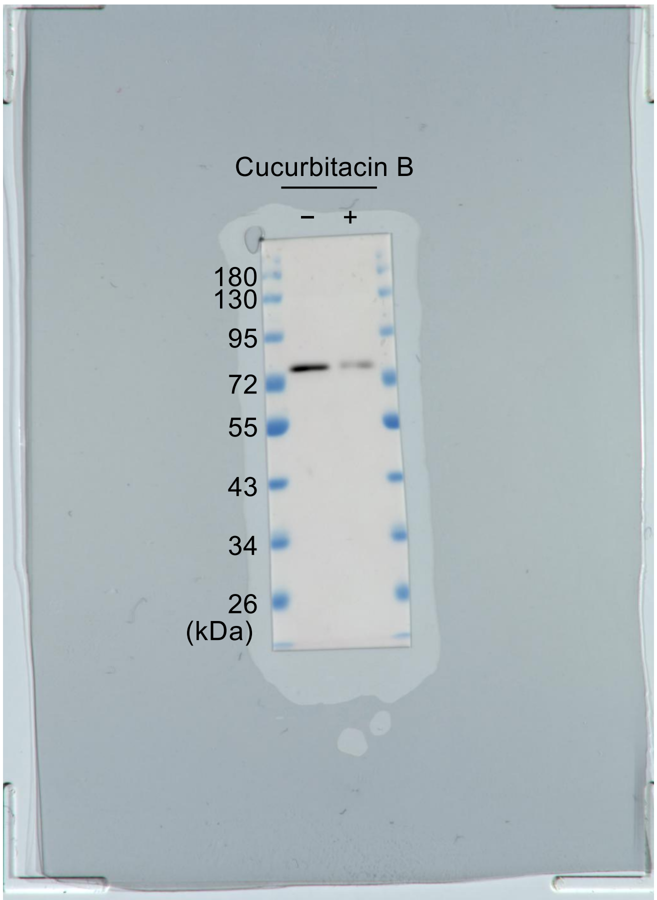


WB: RAF1

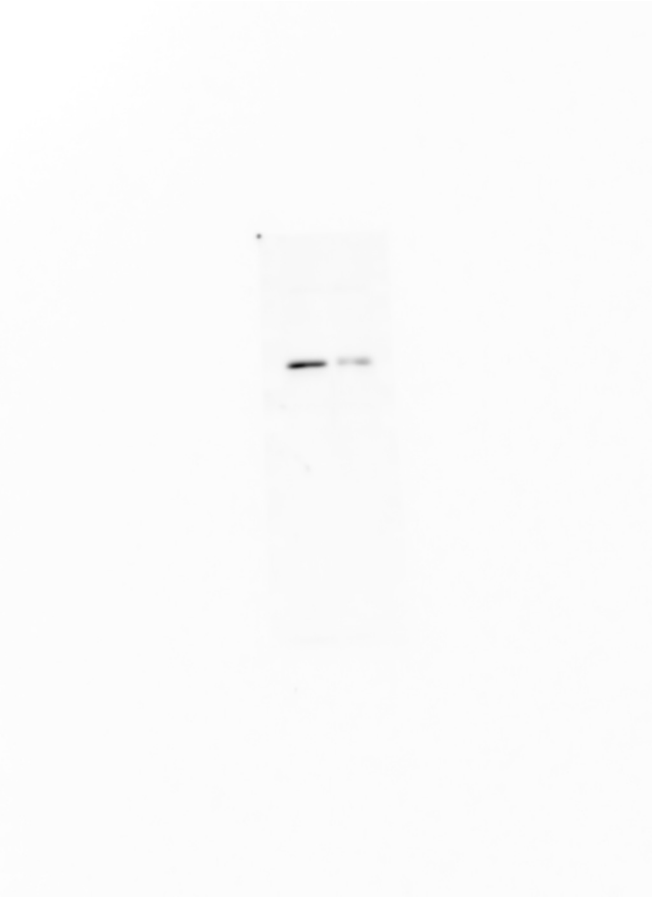

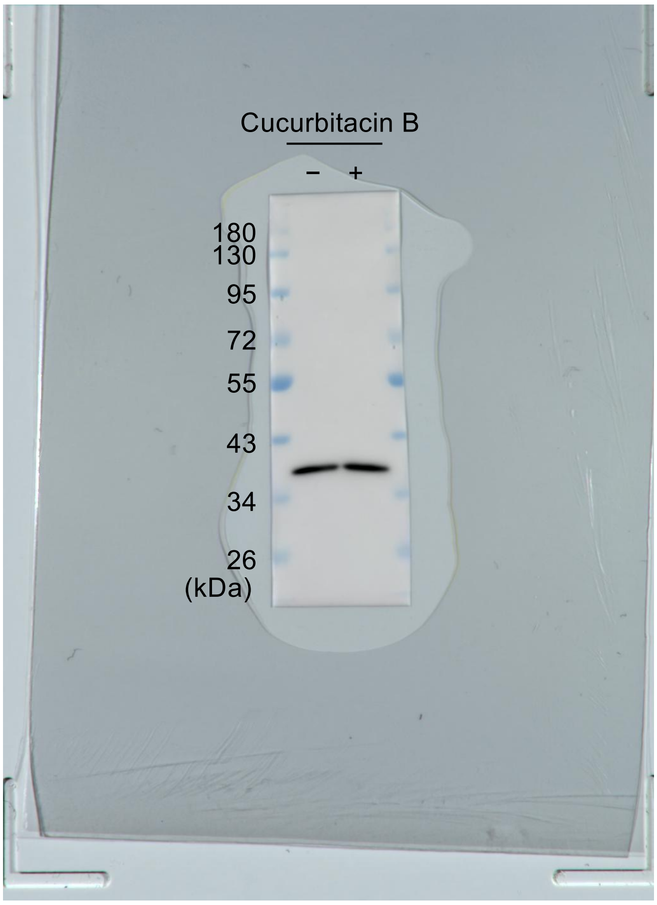


WB: GAPDH (reprobed)

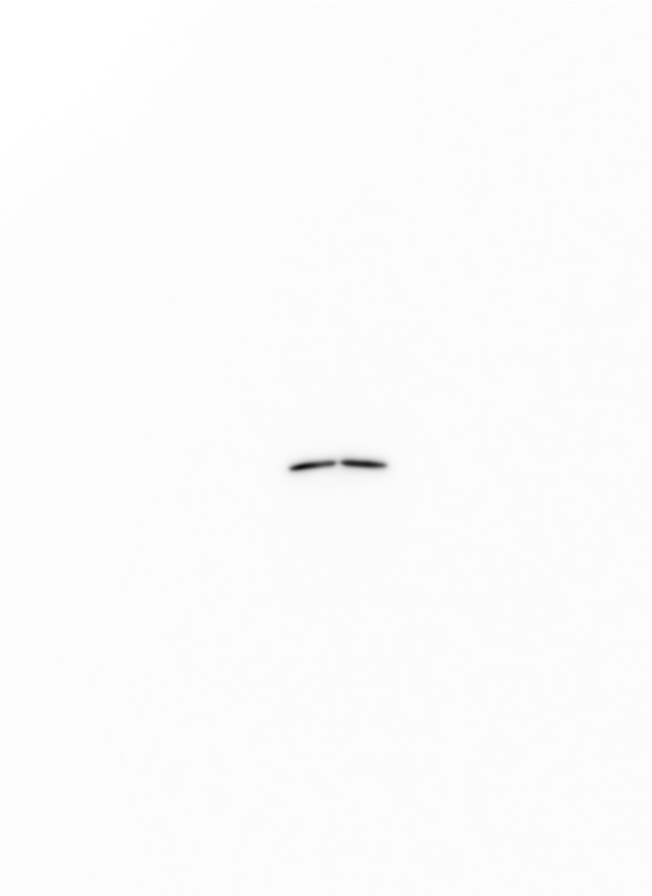

## Figure S85: Original blots (2) in Figures 10C and 10D

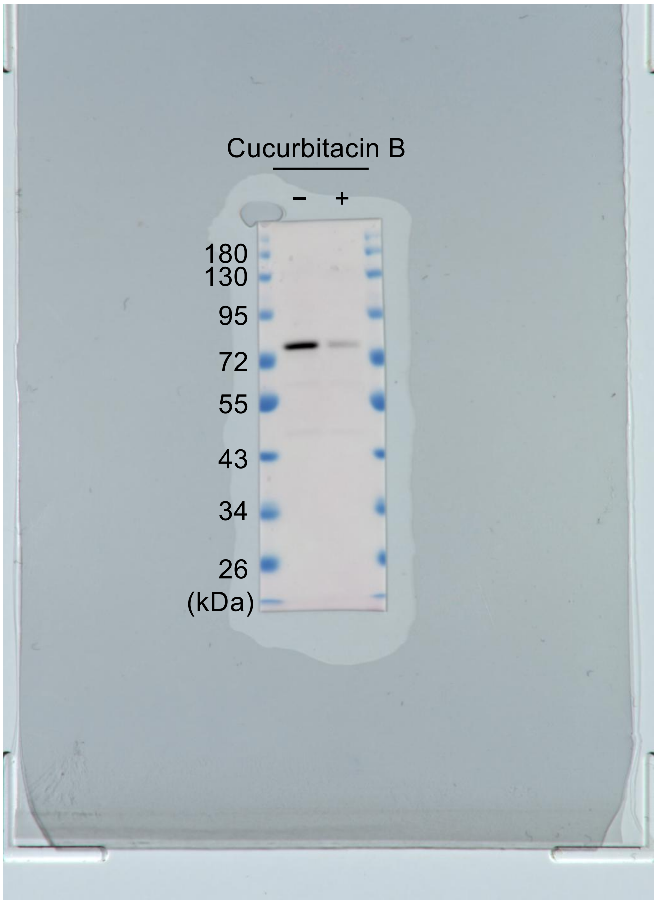


WB: RAF1



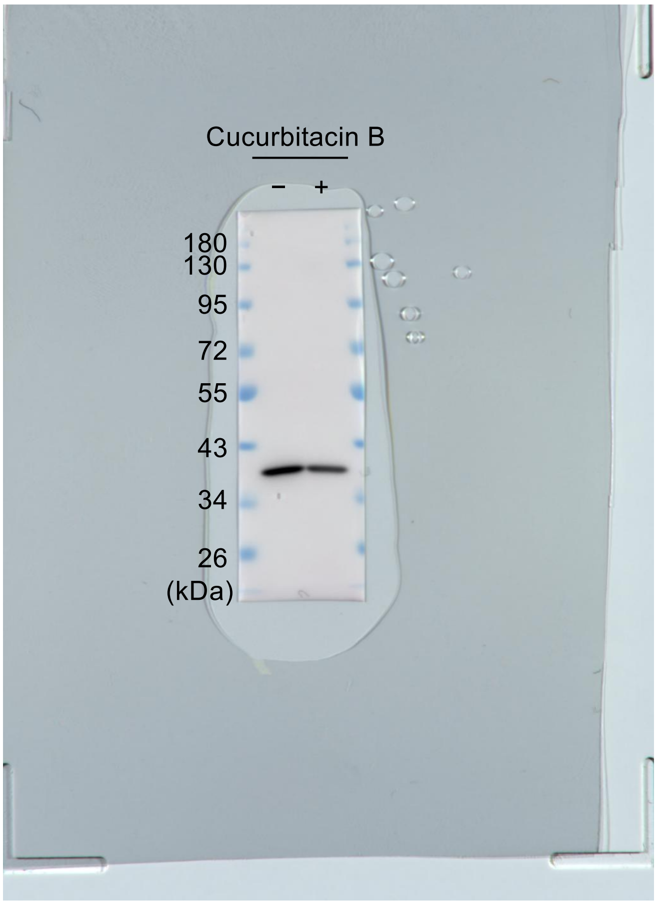


WB: GAPDH (reprobed)

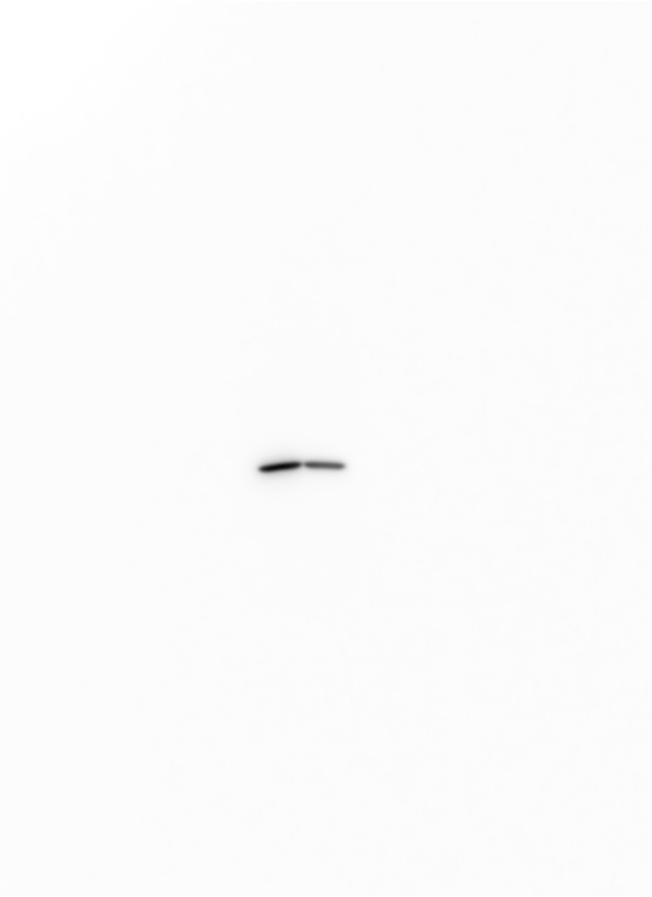

# Figure S86: Original blots (3) in Figures 10C and 10D

WB: RAF1

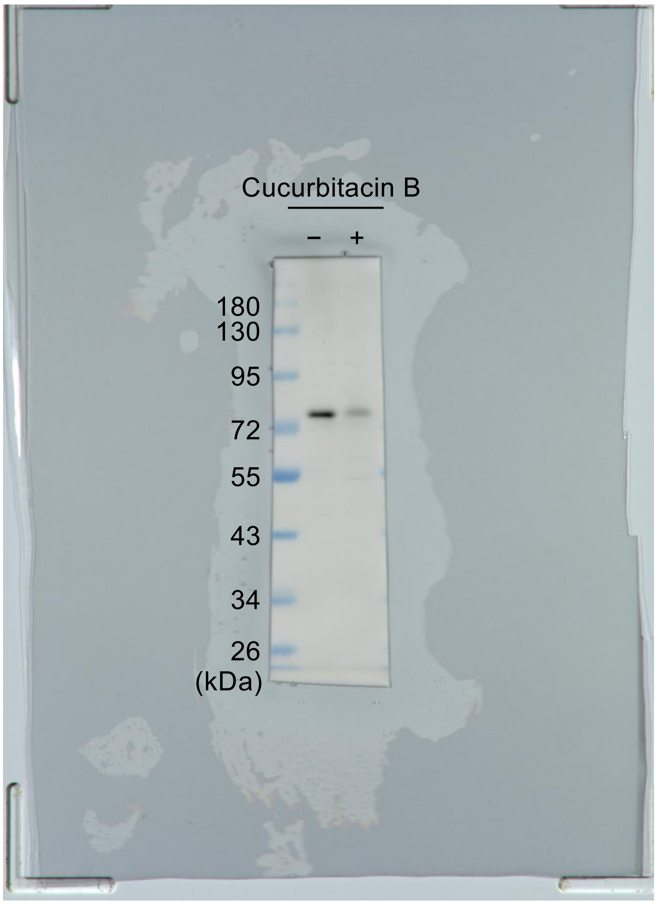




WB: GAPDH (reprobed)

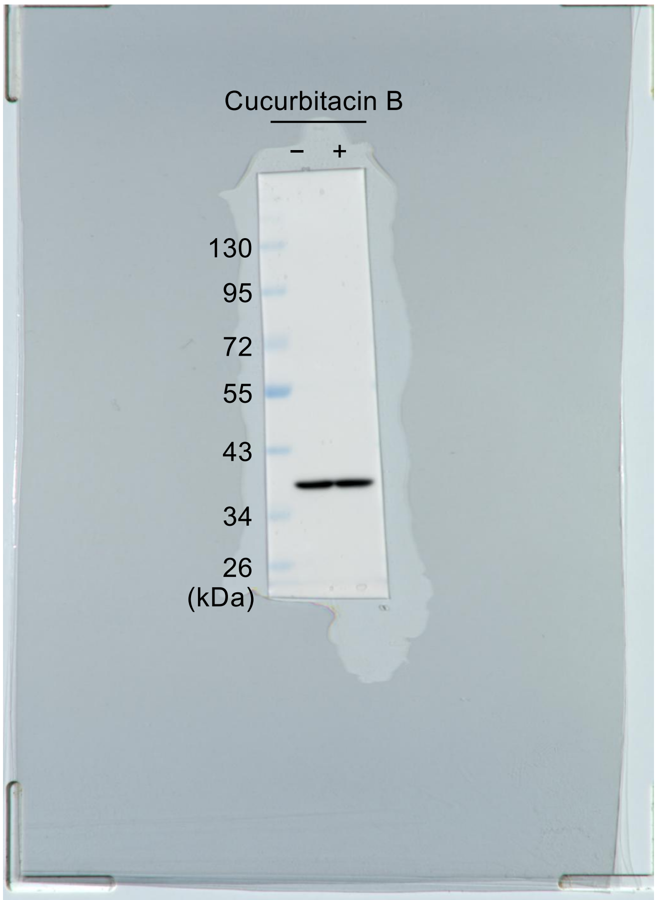

Supplement: Supplementary file 1 [file ijms-27-05011-s001.zip › ijms-4147720-supplementary.pdf]
